# Important Odorants of Four Brassicaceae Species, and Discrepancies between Glucosinolate Profiles and Observed Hydrolysis Products

**DOI:** 10.3390/foods10051055

**Published:** 2021-05-11

**Authors:** Luke Bell, Eva Kitsopanou, Omobolanle O. Oloyede, Stella Lignou

**Affiliations:** 1 School of Agriculture, Policy and Development, University of Reading, Whiteknights, Reading RG6 6AR, UK; luke.bell@reading.ac.uk; 2 Sensory Science Centre, Department of Food and Nutritional Sciences, Harry Nursten Building, University of Reading, Whiteknights, Reading RG6 6DZ, UK; evakitsopanou@gmail.com (E.K.); bola.oloyede@reading.ac.uk (O.O.O.)

**Keywords:** volatile compounds, odorants, glucosinolate, Brassicaceae, ‘salad’ rocket, wasabi, horseradish, watercress

## Abstract

It is widely accepted that the distinctive aroma and flavour traits of Brassicaceae crops are produced by glucosinolate (GSL) hydrolysis products (GHPs) with other non-GSL derived compounds also reported to contribute significantly to their aromas. This study investigated the flavour profile and glucosinolate content of four Brassicaceae species (salad rocket, horseradish, wasabi, and watercress). Solid-phase microextraction followed by gas chromatography-mass spectrometry and gas chromatography-olfactometry were used to determine the volatile compounds and odorants present in the four species. Liquid chromatography-mass spectrometry was used to determine the glucosinolate composition, respectively. A total of 113 compounds and 107 odour-active components were identified in the headspace of the four species. Of the compounds identified, 19 are newly reported for ‘salad’ rocket, 26 for watercress, 30 for wasabi, and 38 for horseradish, marking a significant step forward in understanding and characterising aroma generation in these species. There were several non-glucosinolate derived compounds contributing to the ‘pungent’ aroma profile of the species, indicating that the glucosinolate-derived compounds are not the only source of these sensations in Brassicaceae species. Several discrepancies between observed glucosinolates and hydrolysis products were observed, and we discuss the implications of this for future studies.

## 1. Introduction

Crops of the Brassicaceae family are grown all over the world, and they form an important part of many different cuisines and cultures [1]. Some species are noted for their distinctive, and often very strong, tastes and flavours. *Armoracia rusticana* (horseradish), *Eruca sativa* (‘salad’ rocket), *Eutrema japonicum* (wasabi), and *Nasturtium officinale* (watercress) are four such examples, which are noted for their pungent, peppery, and aromatic organoleptic properties [2,3,4].

Horseradish and wasabi produce large roots that are grated and used as a condiment in many cultures across the world, most notably in Eastern Europe and the United Kingdom (horseradish) and Japan (wasabi; [5]). Horseradish is a vegetative perennial that grows widely in temperate regions [6], whereas wasabi can only be grown in a very few locations, owing to its sensitivity to temperature change and root oxygen availability [7]. Wasabi is traditionally cultivated in damp river valleys of Japan, although commercial operations have been established elsewhere, such as in the UK.

Salad rocket originates from the Middle East and has spread throughout the Mediterranean basin [8]. It has become naturalised on every inhabited continent and is considered an invasive weed in some regions. Watercress has similarly become naturalised (for example in North America) and grows in shallow rivers and streams. It can be cultivated commercially on large scales using artificial growing ‘pools’ flooded with stream water. Its leaves and shoots are a popular salad and sandwich garnish, and they can also be made into soups [9].

**Figure 1:**
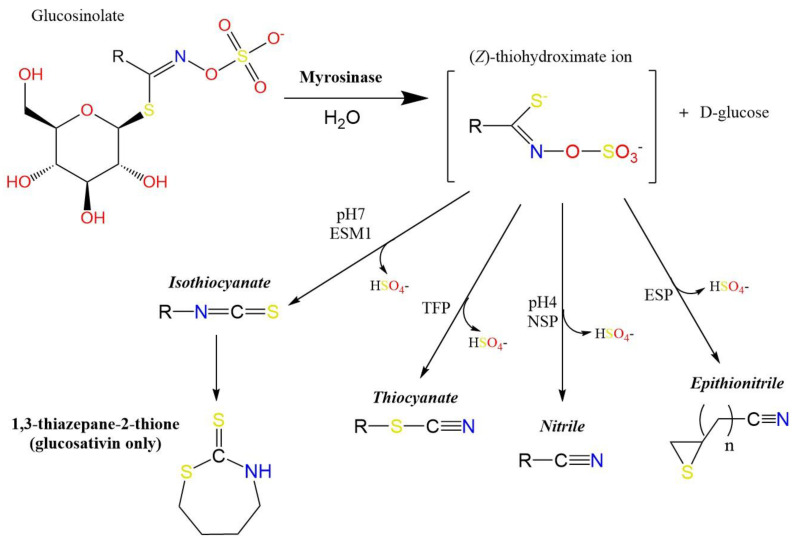
The glucosinolate–myrosinase reaction and hydrolysis products. Abbreviations: ESM1, epithiospecifier modifier protein 1; TFP, thiocyanate forming protein; NSP, nitrile specifier forming protein; ESP, epithiospecifier protein.

It is widely accepted that the distinctive aroma and flavour traits are produced by glucosinolate (GSL) hydrolysis products (GHPs [10]). GSLs are sulphur-containing secondary metabolites produced by Brassicales plants in response to biotic and abiotic stress [11]. Myrosinase enzymes are responsible for the hydrolysis of GSLs in water to form a plethora of GHPs, the conformation of which can be determined by the presence of enzyme cofactors, pH level, metallic ion concentration, and the precursor GSLs side-chain structure [8]. These products include: isothiocyanates (ITCs), nitriles, epithionitriles, indoles, oxazolidine-2-thiones, and other diverse products that result from tautomeric rearrangements (Figure 1).

Previous work has reported olfactometry data for each of these crops; however, data are generally very scarce. Only five studies of *E. sativa* volatile compounds have been published in the last twenty years [3,12,13,14,15]. Very little information regarding wasabi root volatile composition and aroma is available outside of Japanese language journals [7,16]. Similarly, very little information is available for watercress, with only four papers published in the last 40 years [17,18,19,20]. Horseradish is the most well characterised of these four species, but still, only six studies of note have been published in the last 50 years [6,21,22,23,24,25].

There is also an ‘elephant in the room’ regarding previous reports of GHPs present in aroma profiles of these Brassicaceae vegetables. Many of the reported GHP compounds are derived from GSL precursors that are not regularly reported as part of the profile for each respective species. In *E. sativa*, for example, GHPs such as methyl ITC, 3-butenyl ITC, 1-isothiocyanato-4-methylpentane, and 5-(methylsulfanyl)pentanenitrile have all been previously reported [13]. The GSL precursors to these compounds (glucocapparin, GCP; gluconapin, GNP; 4-methylpentyl GSL, 4MP; and glucoberteroin, GBT; respectively) have never been reliably or consistently reported as being part of the GSL profile in this species [26,27]. This begs the question whether these identifications are correct or if the reports of GSL components in these species are incomplete. This may be due to a lack of sensitivity in reported mass spectrometry methods or because there is a lack of analytical standards to confirm compound identities. Another possibility is that there is a post-hydrolysis modification of GHPs, either by enzymatic means or through reactions with other phytochemical components, or as part of thermolytic reactions during gas chromatography. Very few examples of such modifications have been reported within the literature [28], but this may explain the presence of some GHPs within profiles of species where the GSL precursor is absent.

**Figure 2:**
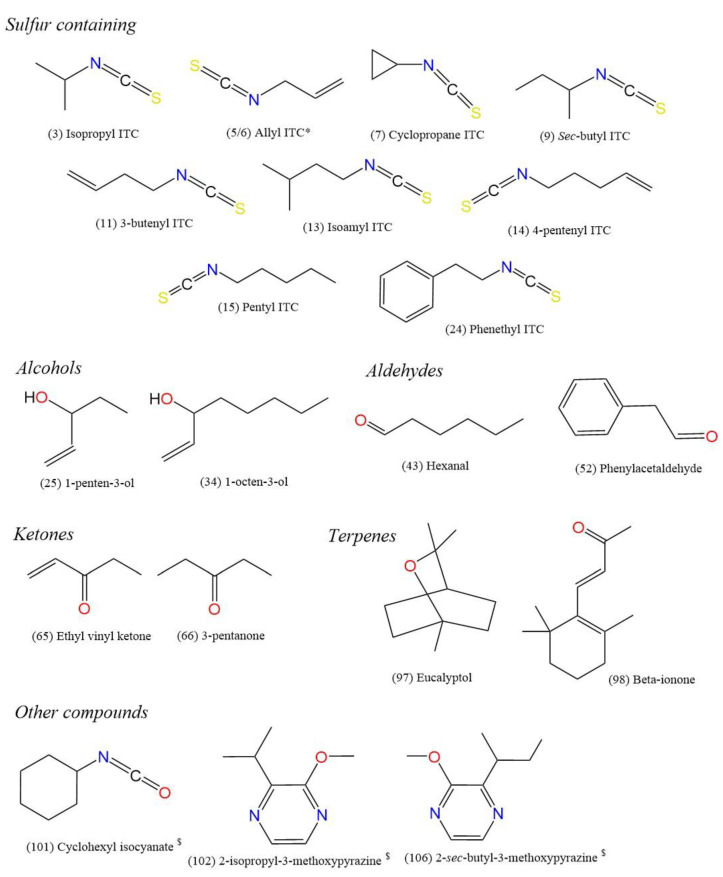
Chemical structures of volatile compounds found in horseradish, rocket, wasabi, and watercress samples (numbers in parentheses refer to compound codes in Table 2); * two separate peaks present for this compound; ^$^ tentatively identified.

Other non-GSL derived compounds have also been reported to contribute significantly to the aromas of Brassicaceae crops. 2-Isopropyl-3-methoxypyrazine has been found to produce a strong pea, or green, pepper-like aroma in horseradish, for example ([6] Figure 2). In rocket, ‘green-leaf’ volatiles such as 3-hexenal and 1-penten-3-one have also been highlighted as having high odour potency [12]. The role of these compounds in aroma generation in GSL-containing crops is often not fully appreciated, and there are many diverse compounds with equally high odour intensities to GHPs present within the volatile bouquet.

The aims of this study were to (i) identify and describe in detail the key odorants of four Brassicaceae species (salad rocket, horseradish, wasabi, and watercress) by gas chromatography-olfactometry (GC-O) and gas chromatography-mass spectrometry (GC-MS), and (ii) associate the observed GHPs with their respective GSL profiles by liquid chromatography-mass spectrometry (UPLC-MS/MS). Our goal was to improve upon existing compound characterisation and odour descriptors for compounds in salad rocket, horseradish, wasabi, and watercress. The contribution of pungency by GHPs to aroma profiles is well studied; however, other aroma traits generated by non-GHPs are not well described for these species, and they likely create distinctive and subtle sensory characteristics. Additionally, detailed GSL compositions and MS/MS spectra for rocket, wasabi, and watercress is presented, highlighting discrepancies with observed hydrolysis products.

## 2. Materials and Methods

### 2.1. Samples

Individual horseradish and wasabi roots were purchased from Morrison’s supermarket (Reading, UK) and The Wasabi Company (Dorchester, UK) respectively. Watercress was purchased as whole bags of leaves from ASDA supermarket (Reading, UK). Salad rocket was grown in controlled environment conditions at the University of Reading using seeds donated by Elsoms Seeds Ltd. (Spalding, UK) and designated RS4 and RS8. Seeds of each cultivar were sown into module trays containing peat-based seedling compost and germinated at 30 °C (daytime; 25 °C night). Lighting conditions were set to a long-day cycle (16 h light, 8 h dark). Light intensity was set to 380 µmol m^−2^ s^−1^. Humidity was ambient. Plants were considered mature upon the development of 10 to 15 leaves. Harvested leaves were taken intact and placed inside a Zip-loc bag.

Roots and leaves were placed in a fridge upon either purchase or harvest (4 °C) until further analysis was performed.

### 2.2. Chemicals

For headspace solid-phase-microextraction (HS-SPME), calcium chloride and the alkane standards C_6_-C_25_ (100 μg/mL) in diethyl ether were obtained from Sigma-Aldrich (now Merck; Poole, UK). For ultra-high performance liquid chromatography mass spectrometry (UPLC-MS), authentic compounds of glucoiberin (GIB; 99.61%, HPLC), progoitrin (PRO; 99.07%, HPLC), sinigrin (SIN; 99%, HPLC), glucoraphanin (GRA; 99.86%, HPLC), glucoalyssin (GAL, 98.8%, HPLC), gluconapin (GNP, 98.66%, HPLC), 4-hydroxyglucobrassicin (4HGB; 96.19%, HPLC), glucobrassicanapin (GBN; 99.22%, HPLC), glucotropaeolin (GTP; 99.61%, HPLC), glucoerucin (GER; 99.68%, HPLC), glucobrassicin (GBR; 99.38%, HPLC), and gluconasturtiin (GNT; 98.38%, HPLC) were purchased from PhytoPlan (Heidelberg, Germany). Methanol (HPLC grade), formic acid (LC-MS grade), and acetonitrile (LC-MS grade) were purchased from VWR (Leicestershire, UK).

### 2.3. Volatile Compounds

#### 2.3.1. Headspace Solid Phase Microextraction (SPME)

Samples of respective leaf and root tissues were homogenised by means of a commercial blender for 30 s, and 2 g of each was weighed into a SPME vial of 15 mL fitted with a screw cap. Samples were left aside for 10 min for the enzymatic hydrolysis of GSLs to take place. After exactly 10 min, 2 mL of saturated CaCl_2_ was added in order to cease the enzymatic reactions. After equilibration at 40 °C for 10 min, a 50/30 μm DVB/CAR/PDMS fibre was exposed to the headspace above the sample for 20 min. Three biological replicates were prepared for GC-MS analysis, and two replicates for each of the three assessors were prepared for the GC-O analysis.

#### 2.3.2. GC-MS Analysis of SPME Extracts

After extraction, the SPME device was inserted into the injection port of an Agilent 7890A gas chromatography system coupled to an Agilent 5975C detection system equipped with an automated injection system (CTC-CombiPAL). A capillary column HP-5MS (30 m × 0.25 mm × 0.25 μm film thickness) (Agilent, Santa Clara, CA, USA) coated with (5% Phenyl Methyl Silox) was used for the chromatographic separation of volatile compounds. The oven temperature program used was 2 min at 40 °C isothermal and an increase of 4 °C/min to 250 °C. Helium was used at 3 mL/min as carrier gas. The sample injection mode was splitless. Mass spectra were measured in electron ionisation mode with an ionisation energy of 70 eV, the scan range from 20 to 280 *m/z* and the scan rate of 5.3 scans/s. The data were controlled and stored by the HP G1034 Chemstation system. Identities were confirmed by running the samples on a Stabilwax-DA (30 m × 0.25 mm × 0.25 μm film thickness) polar column from Restek (Bellefonte, PA, USA). Volatile compounds were identified or tentatively identified by comparison of each mass spectrum with spectra from authentic compounds analysed in our laboratory, or from the NIST mass spectral database (NIST/EPA/NIH Mass Spectral database, 2014), or spectra published elsewhere (see Supplementary Data S1 for GC-MS chromatograms and compound fragmentation spectra). A spectral quality value >80 was used alongside linear retention index to support the identification of compounds where no authentic standards were available. LRI was calculated for each volatile compound using the retention times of a homologous series of C_6_-C_25_ *n*-alkanes and by comparing the LRI with those of authentic compounds analysed under similar conditions. The compound peak areas were normalised and converted to the relevant abundance of each component as a percentage of the total peak area.

#### 2.3.3. GC-O Analysis of SPME Extracts

After extraction, the SPME device was inserted into the injection port of an Agilent 7890B Series ODO 2 (SGE) GC-O system equipped with a non-polar HP-5MS column (30 m × 0.25 mm × 0.25 μm film thickness). The outlet was split between a flame ionisation detector and a sniffing port. The contents of the SPME fibre were desorbed for 3 min in a split/splitless injection port, in splitless mode, onto five small loops of the column in a coil, which were cooled in solid carbon dioxide and contained within a 250 mL beaker. The injector and detector temperatures were maintained at 280 °C and 250 °C, respectively. During desorption, the oven was held at 40 °C. After desorption, the solid carbon dioxide was removed from the oven. The oven was maintained at 40 °C for a further 2 min and then, the temperature was raised at 4 °C/min to 200 °C and at 8 °C/min to 300 °C. Helium was the carrier gas, and the flow rate was 2.0 mL/min. Three assessors were used for the detection and verbal description of the odour active components of the SPME extracts. Each assessor participated in three training sessions for each sample species prior to scoring sessions. Each assessor evaluated by sniffing each sample in duplicate and documented the odour description, retention time, and odour intensity (OI) on a seven-point scale (2–8), where <3 = weak, 5 = medium, and 7 = strong. Only those odours that were detected by all three assessors were recorded in the results. *n*-Alkanes C_6_-C_25_ were analysed under the same conditions to obtain LRI values for comparison with the GC-MS data.

### 2.4. Non-Volatile Compounds

#### 2.4.1. Glucosinolate (GSL) Extraction

GSL extraction was performed as per the protocol presented by [29] with modifications. Briefly, 40 mg of dried leaf powder was placed into Eppendorf tubes and put into a heat block (80 °C for ten minutes). Afterwards, 1 mL of preheated methanol water (70% *v/v*) was added to dried powder, vortexed vigorously, and placed in a water bath (75 °C) for 20 min. Samples were cooled and centrifuged at full speed for five minutes at room temperature (≈22 °C); the supernatant was collected and filtered (0.22 µm PVDF Acrodisc syringe filters; VWR, Lutterworth, UK). Crude extracts were dried using a centrifugal evaporator and re-suspended in 1 mL of LC-MS-grade H_2_O and stored at −80 °C until analysis. Immediately before analysis by UPLC-MS, samples were diluted five-fold with LC-MS-grade H_2_O.

#### 2.4.2. UPLC-MS Analysis of GSL Extracts

UPLC-MS was performed on a Shimadzu Nexera X2 series UHPLC, coupled with an 8050 triple quadrupole mass spectrometer system (Shimadzu UK Ltd., Milton Keynes, UK). Separation of standards and samples was achieved using a Waters BEH C_18_ Acquity column (100 × 2.1 mm, 1.7 µm; Waters Corp., Wilmslow, UK) with an Acquity in-line filter. Mobile phases consisted of 0.1% formic acid in LC-MS grade H_2_O (A), and 0.1% formic acid in LC-MS grade acetonitrile (B) and GSLs were separated during a five minute run with the following gradient timetable: (i) 0–50 s (A-B, 98:2, *v/v*), (ii) 50 s–3 min (A-B, 70:30, *v/v*), (iii) 3–3 min 10 s (A-B, 5:95, *v/v*), (iv) 3 min 10 s–4 min (A-B, 5:95, *v/v*), (v) 4–4 min 10 s (A-B, 98:2, *v/v*), (vi) 4 min 10 s–5 min (A-B, 98:2, *v/v*). The flow rate was 0.4 mL per min and the column oven temperature was 35 °C.

Two MS methods were used for the identification and quantification of GSLs. First, a Product Ion Scan (PIS) method was established to identify GSLs based on known primary ion masses ([M-H]-) characteristic fragment ions (357, 258, and 97 *m/z*; Table 1). Then, MS/MS spectra were compared to authentic standards and available literature sources [30,31,32,33,34,35,36,37,38]. Pentyl GSL (PEN), isobutyl GSL (ISO), glucoputranjivin (GPJ), and butyl GSL (BUT) were tentatively identified due to the possible presence of isomers [38], and/or no reliable reference MS spectra could be found in the literature. Total ion chromatograms of glucosinolates identified were included in the Supplementary Data (S2).

MS/MS settings for the PIS method were as follows: samples were analysed in the negative ion mode with a scan range of 70–820 *m/z*. A collision energy of 25 eV and a scan speed of 30,000 u per s^−1^. For the quantification of GSLs, a Multiple Reaction Monitoring (MRM) method was established. Based on the fragmentation observed in the PIS method, confirmation and quantification transitions were established (Table 1). Dwell times for each precursor and product ion were set to 5 s.

Authentic GSL compounds were run as external standards. Limits of detection (LOD) and limits of quantification (LOQ) were established for each and are presented in Table 1. As standard compounds are not available for all GSLs, SIN was used to semi-quantify glucorucolamine (GRM), glucoputranjivin (GPJ), diglucothiobeinin (DGTB), glucoberteroin (GBT), glucocochlearin (GCL), glucosativin (GSV), dimeric 4-mercaptobutyl GSL (DMB), glucobarbarin (GBB), and tentatively identified GSL compounds. Similarly, GBR was used to semi-quantify the indolic GSLs 4-methoxyglucobrassicin (4MGB) and neoglucobrassicin (NGB).

## 3. Results and Discussion

### 3.1. Volatile Compounds

The volatile compounds identified in the headspace of the four Brassicales species are listed in Table 2, detailing their PubChem compound identification (PubChem CID) as well as their linear retention indices (LRI) in a polar and non-polar column. Semiquantitative characterisation results are also shown in Table 2 as relative area. ITCs and alcohols were the chemical classes of compounds dominating the volatile profile of the samples with other compounds such as aldehydes, esters, and terpenes also present.

GC-olfactometry analysis of the samples yielded a total of 107 odorants across the four species, which are presented in Table 3. Qualitative differences were observed between the samples with horseradish and watercress yielding a total of 52 and 51 odorants, respectively. Green/grassy, radish, sulphury, and horseradish were some of the terms that were mostly used by the assessors to describe the odours. Additionally, a total of 46 odorants of unknown identity were detected within the headspace of the four Brassicales analysed that may contribute to the odour profiles of these crops. These compounds matched no corresponding peaks and LRI values within the GC-MS data. This suggests that the compounds responsible for generating the perceived aromas were present at levels below the detection threshold of the instrumentation used. The number and diversity of unidentified compounds and aromas is indicative of the fact that characterisation of the species’ volatile profiles is far from complete, and it is likely that many more will be discovered in future studies.

**Table 1:** Glucosinolate compounds identified in salad rocket, wasabi, and horseradish by UPLC-MS/MS.

Glucosinolate	Common Name	Abbreviation	Rt	LOD (nmol g^−1^)	LOQ (nmol g^−1^)	R^2^	Precursor Ion ([M-H]-)	Quantification Transition	Confirmation Transition	MS/MS of [M-H]- Ion m/z (Relative Intensity)	References
3-(methylsulfinyl)propyl	glucoiberin *	GIB	1.061	0.053	0.161	0.969	422	422 > 357	422 > 97	**357**(57), **97**(100), 95(61)	[30,31]
pentyl ^a,$^	-	PEN	1.08	-	-	-	388	388 > 75	388 > 97	273(15), 258(13), 194(14), 192(13), 97(100), 89(24), 79(18),75(71), 74(11)	-
(*R*)-2-hydroxy-3-butenyl	progoitrin *	PRO	1.336	0.066	0.199	0.933	388	388 > 74	388 > 97	**97**(100), 74(75)	[32]
allyl	sinigrin *	SIN	1.523	0.105	0.319	0.982	358	358 > 258	358 > 97	**258**(10), **97**(100), **74**(25)	[30,32,33]
isobutyl ^a,$^	-	ISO	1.56	-	-	-	374	374 > 240	374 > 115	277(11), 274(20), 258(86), 257(62), 240(95), 115(100), 97(26), 95(38)	-
4-(methylsulfinyl)butyl	glucoraphanin *	GRA	1.598	0.059	0.179	0.989	436	436 > 371	436 > 97	**371**(80), **97**(100)	[30,32]
4-(cystein-*S*-yl)butyl	glucorucolamine ^a,$^	GRM	1.654	-	-	-	494	494 > 406	494 > 217	413(32), 406(66), 404(38), 295(32), 291(17), 275(38), 250(66), 217(100), 209(33), 195(20), 171(38), 145(38), 129(17), 114(17), 112(40), 97(47), 96(32), 75(16)	-
5-(methylsulfinyl)pentyl	glucoalyssin *	GAL	2.421	0.064	0.194	0.971	450	450 > 206	450 > 97	**386**(42), **275**(49), 263(21), **208**(21), 206(100), **190**(71), **97**(51), 75(49)	[31,32,34]
1-methylethyl	glucoputranjivin ^a,$^	GPJ	2.447	-	-	-	360	360 > 75	360 > 97	359(24), 119(13), 97(100), 94(34), 75(54)	-
3-butenyl	gluconapin *	GNP	2.489	0.151	0.459	0.953	372	372 > 258	372 > 97	**258**(6), **97**(100)	[30,31]
4-(β-D-glucopyrano syldisulfanyl)butyl	diglucothiobeinin ^a,$^	DGTB	2.501	-	-	-	600	600 > 290	600 > 97	290(7), **97**(100)	[32]
5-(methylthio)pentyl	glucoberteroin ^a,$^	GBT	2.593	-	-	-	434	434 > 95	434 > 97	146(60), **97**(73), 95(100)	[31,35]
4-hydroxy-3-indolylmethyl	4-hydroxygluco brassicin *	4HGB	2.619	0.15	0.454	0.943	463	463 > 285	463 > 97	**285**(17), **97**(100)	[30,32]
1-methylpropyl	glucocochlearin ^a,$^	GCL	2.682	-	-	-	374	374 > 75	374 > 97	**293**(10), **275**(19), 98(58), 97(100), 95(31), 84(10), **75**(81)	[33]
4-mercaptobutyl	glucosativin ^a,$^	GSV	2.684	-	-	-	406	406 > 74	406 > 97	**259**(12), 97(100), 76(19), 74(23)	[32]
7-(methylsulfinyl)heptyl ^a,$^	-	7MSH	2.737	-	-	-	478	478 > 413	478 > 97	413(46), 259(12), 219(11), 98(68), **97**(100), 75(12)	[36]
4-pentenyl	glucobrassicanapin ^a^	GBN	2.819	0.081	0.245	0.945	386	386 > 75	386 > 96	**97**(100), **96**(44), **75**(50)	[33]
dimeric 4-mercaptobutyl ^a,$^	-	DMB	2.839	-	-	-	811	405 > 80	405 > 97	208(11), 97(100), 81(15), 80(16), 75(15)	[32]
2(*S*)-hydroxy-2-phenylethyl	glucobarbarin ^a,$^	GBB	2.847	-	-	-	438	438 > 98	438 > 96	437(16), **332**(20), **274**(17), 195(11), 137(29), **135**(18), 98(48), **96**(100), **74**(30)	[37]
benzyl	glucotropaeolin *	GTP	2.879	0.129	0.39	0.962	408	408 > 259	408 > 97	**259**(10), **97**(100)	[30,31]
4-(methylthio)butyl	glucoerucin *	GER	2.919	0.04	0.121	0.948	420	420 > 74	420 > 96	**258**(16), **241**(17), **178**(15), **96**(100), 75(13), 74(27)	[30,35]
indolyl-3-methyl	glucobrassicin *	GBC	3.102	0.096	0.29	0.955	447	447 > 259	447 > 97	**259**(10), **97**(100)	[31,32]
4-methoxyindolyl-3-methyl	4-methoxygluco brassicin ^b,^^$^	4MGB	3.173	-	-	-	477	477 > 75	477 > 97	**259**(12), **258**(13), 127(10), 119(13), 98(53), **97**(100), 84(15), **75**(32), 74(15)	[30,31,33,34]
2-phenethyl	gluconasturtiin *	GNT	3.419	0.062	0.189	0.968	422	422 > 259	422 > 97	**259**(10), **97**(100)	[32,33]
1-methoxyindolyl-3-methyl	neoglucobrassicin ^b,^^$^	NGB	3.526	-	-	-	477	477 > 75	477 > 97	**144**(13), **97**(100), 84(16), 82(11), 75(22)	[32]
4-methylpentyl ^a,$^	-	4MP	3.695	-	-	-	402	402 > 259	402 > 97	**275**(11), **259**(24), 195(16), 179(10), 159(20), 97(100), 85(12)	-
hexyl ^a,$^	-	HEX	3.726	-	-	-	402	402 > 119	402 > 97	401(22), **274**(15), **241**(59), **226**(32), 204(14), **198**(15), 197(14), 168(14), 161(56), **160**(32), **138**(32), 121(32), 119(64), 116(15), 114(14), 98(46), **97**(100), 96(65), 85(15), 79(15)	[31,32]
7-(methylthio)heptyl ^a,$^	-	7MTH	4.109	-	-	-	462	462 > 75	462 > 97	283(11), 275(11), 274(11), 220(17), **97**(100), 75(32)	[36]
butyl ^a,$^	-	BUT	4.176	-	-	-	375	375 > 256	375 > 180	328(48), 316(41), 307(41), 260(41), 256(58), 235(41), 207(41), 195(50), 185(20), 180(100), 143(20), 120(63), 97(41)	-

Ions in bold agree with previous studies; ions underlined indicate characteristic ions associated with glucosinolates. * Authentic standard; ^a^ quantified using sinigrin; ^b^ quantified using glucobrassicin; ^$^ tentative identification.

**Table 2:** Volatile compounds identified in the headspace of four Brassicales species analysed by HS-SPME GC-MS.

Compound Number	Compound	PubChem ID	LRI ^a^		ID ^b^	Peak Areas (% of total)					References
			HP-5MS	Stabilwax		Salad Rocket		Wasabi	Horseradish	Watercress	
	*Sulphur-containing compounds*										
1	carbon disulphide	6348	<600	738	B	0.43 *		0.04 *	0.02	0.11 *	[21]
2	methyl thiocyanate	11168	711	1266	B	0.13		nd	nd	0.48	[12]
3	isopropyl ITC	75263	835	1176	B	nd		4.07	0.03	nd	[7,23]
4	allyl thiocyanate	69816	870	1358	B	nd		0.48 *	0.73	nd	[22]
5	allyl ITC ^	5971	881	1371	B	nd		8.61	7.35	nd	[7,14,18,24]
6	allyl ITC ^		890	1392	B	nd		52.11	39.34	nd	
7	cyclopropane ITC	92463	899	1223	B	nd		0.07 *	0.05 *	nd	-
8	cyclopentyl-1-thiaethane	138938	922	1068	B	nd		nd	nd	0.51 *	-
9	*sec*-butyl ITC	78151	933	1265	B	0.46 *		4.95	1.84	nd	[7,23]
10	isobutyl ITC	68960	955	1316	B	nd		1.80	0.45	nd	[7,21]
11	3-butenyl ITC	76922	982	1455	B	0.19		4.24	1.52	nd	[3,7,22]
12	butyl ITC	11613	998	1597	B	0.67		0.12	0.32	nd	[7,15]
13	isoamyl ITC	79086	1059	1431	B	0.10		0.41 *	0.55	0.07	[3,18,22]
14	4-pentenyl ITC	87436	1086	1543	B	nd		12.42	2.26	nd	[7,22]
15	pentyl ITC	69415	1098	1488	B	0.56 *		0.12 *	0.20	nd	[21]
16	1-isothiocyanato-4-methylpentane	519452	1162	1522	B	4.96		0.01 *	0.04	0.18	[3,18,21]
17	cyclohexyl ITC	14289	1177	1650	B	nd		0.01 *	nd	nd	-
18	<unidentified ITC>	-	1193			nd		8.57	nd	nd	-
19	ibervirin	62351	1315	1991	B	0.58		0.29	1.02	nd	[3,7,22]
20	sativin	85704368	1353	>2000	B	0.55		nd	nd	nd	[12]
21	octyl ITC	78161	1372	1760	B	nd		nd	nd	0.16 *	-
22	benzyl ITC	2346	1372	>2000	B	nd		0.03 *	1.14	nd	[12,22]
23	erucin	78160	1440	>2000	B	7.16		0.01	0.02	nd	[7,12,22]
24	phenethyl ITC	16741	1477	>2000	B	nd		0.68	32.47	30.72	[7,12,18,24]
	*Total sulphur-containing compounds*					15.79		99.07	89.78	32.92	
	*Alcohols*										
25	1-penten-3-ol	12020	678	1159	A	0.78		nd	0.10 *	8.06	[3,7,18]
26	pentan-1-ol	6276	763	1251	A	0.13 *		0.02 *	0.02 *	0.25 *	-
27	(*E*)-2-penten-1-ol	5364919	763	1312	A	0.17 *		nd	nd	0.31 *	-
28	(*Z*)-2-penten-1-ol	15306	767	1322	A	0.76		nd	0.06	4.78	[3,22]
29	1-propoxy-2-propanol	15286	840		B	nd		nd	nd	0.24 *	-
30	(*E*)-3-hexen-1-ol	5284503	850	1365	A	nd		nd	nd	0.20	[3,19,22]
31	(*Z*)-3-hexen-1-ol	5281167	856	1387	A	40.88		nd	1.88	35.99	[3,18,22]
32	2-hexen-1-ol	5318042	863	1405	A	1.25		nd	nd	0.35	[7,13,18,22]
33	hexan-1-ol	8103	866	1355	A	4.82 *		nd	0.22	2.61	[7,18]
34	1-octen-3-ol	18827	977		A	0.18		0.01 *	0.02 *	0.06 *	[14]
35	2-ethylhexanol	7720	1025	1501	B	0.12 *		0.01 *	nd	0.12 *	-
36	benzyl alcohol	244	1035		A	nd		nd	0.05 *	nd	-
37	2-phenylethanol	6054	1116	1930	A	0.16 *		nd	0.03 *	2.64 *	-
38	1-nonanol	8914	1167		A	nd		0.03 *	nd	nd	-
39	terpinen-4-ol	11230	1182		A	nd		0.01 *	nd	nd	-
*Total alcohols*						49.25		0.09	2.38	55.62	
	*Aldehydes*										
40	2-pentanal	7895	699		A	nd		0.02 *	nd	nd	-
41	2-pentenal	5364752	753	1136	A	0.31		nd	0.02 *	0.23 *	[3]
42	3-hexenal	643139	796	1178	A	1.72		nd	0.04	0.23	[3,18,22]
43	hexanal	6184	798	1094	A	8.64		<0.01 *	0.37	1.16 *	[12,21]
44	(*E*)-2-hexenal	5281168	852	1224	A	14.30		nd	0.31	2.10	[3,7,18,21]
45	4-heptenal	5283318	901		A	0.08 *		nd	nd	0.51	-
46	heptanal	8130	902		A	0.13		nd	0.02	0.10 *	[13,21]
47	2,4-hexadienal	637564	908		B	0.09		nd	0.01 *	nd	[3,18]
48	benzaldehyde	240	964	1654	A	0.17		0.02 *	nd	nd	[12,22]
49	2,4-heptadienal (isomer 1)	5283321	994		B	0.09		nd	nd	nd	[12]
50	octanal	454	1001		A	nd		0.02 *	nd	nd	-
51	2,4-heptadienal (isomer 2)	5283324	1009	1472	B	0.15		nd	0.25 *	0.10 *	[12]
52	phenylacetaldehyde	998	1047		A	nd		nd	0.02	0.24 *	[22]
53	2-octenal	5283324	1058		A	nd		nd	0.06 *	nd	-
54	nonanal	31289	1101		A	0.41		0.10 *	0.04	0.31 *	[13,21]
55	decanal	8175	1202		A	nd		0.12 *	0.04	0.19 *	[21]
56	vanillin	1183	1403		A	0.02		nd	0.01 *	nd	[13]
*Total aldehydes*						26.11		0.28	1.25	5.17	
	*Esters*										
57	(*Z*)-pent-2-en-1-yl acetate	5363400	910		B	nd		nd	nd	0.07 *	-
58	3-hexenyl acetate	5352557	1003	1284	A	2.54		nd	nd	0.50	[14,18]
59	(*Z*)-3-hexenyl butanoate	5352438	1181	1440	A	0.99		nd	nd	nd	[14]
60	methyl salicylate	4133	1201		A	nd		nd	0.04 *	nd	-
61	ethyl decanoate	8048	1389		A	0.06 *		nd	0.03 *	nd	-
62	methyl dodecanoate	8139	1521		B	0.04		0.01 *	0.02 *	0.08 *	[14]
63	diethyl phthalate	6781	1588		B	0.08 *		nd	nd	nd	-
64	ethyl laurate	7800	1591		B	nd		nd	0.06 *	nd	-
*Total esters*						3.71		0.01	0.15	0.65	
	*Ketones*										
65	ethyl vinyl ketone	15394	682	1035	A	0.43		nd	0.05 *	nd	[3]
66	3-pentanone	7288	697	993	A	0.66		nd	0.05 *	1.86	[3,19]
67	2,3-octanedione	11449	980		A	nd		nd	0.01	nd	[22]
68	6-methyl-5-hepten-2-one	9862	983		A	0.55		nd	nd	0.23 *	[12]
69	2,2,6-trimethylcyclohexanone	17000	1038		B	nd		nd	nd	0.11 *	-
70	(*E,E*)-3,5-octadien-2-one	5352876	1070		B	nd		nd	0.08 *	nd	-
71	3,5-octadien-2-one	5352876	1092		B	nd		nd	0.09 *	nd	-
72	dihydro-2H-thiopyran-3(4H)-one	140474	1160	1856	B	0.58		nd	nd	nd	-
73	geranylacetone	1549778	1451		A	0.10 *		0.02 *	nd	nd	-
*Total ketones*						2.32		0.02	0.27	2.21	
	*Nitriles*										
74	3-butenenitrile	8009	654		B	nd		0.15	0.37	nd	[7,21]
75	5-methylhexanenitrile	29593	943		B	0.27		nd	nd	nd	[13]
76	6-heptenenitrile	4140856	971		B	nd		0.08 *	nd	nd	-
77	thiiraneacetonitrile	148821	1004		B	nd		nd	3.58 *	nd	[39]
78	phenylacetonitrile	8794	1142		B	nd		nd	0.01 *	nd	-
79	5-(methylsulfanyl)pentanenitrile	93320	1200		B	1.13		nd	nd	nd	[12]
80	4-(methylthio)-butanenitrile	100962	1213		B	nd		nd	0.11 *	nd	-
81	benzenepropanenitrile	12581	1243	>2000	B	nd		nd	0.75	1.47	[17,22]
82	<unidentified nitrile>	-	1559		B	nd		0.22	nd	nd	-
*Total nitriles*						1.39		0.45	4.83	1.47	
	*Hydrocarbons*										
83	2,2,4,6,6-pentamethylheptane	26058	991	947	A	nd		nd	nd	0.69 *	-
84	undecane	14257	1096		A	0.23		nd	0.01 *	nd	[15]
85	1-dodecene	8183	1187		A	nd		0.06 *	0.12 *	0.64 *	-
86	dodecane	8182	1196		A	nd		nd	0.01	nd	[22]
87	tridecane	12388	1296		A	nd		nd	0.03	nd	
88	tetradecane	12389	1396		A	0.10		0.01 *	0.06 *	nd	[15]
89	pentadecane	12391	1497		A	nd		nd	0.09 *	nd	-
90	hexadecane	11006	1599		A	nd		nd	0.10 *	nd	-
91	heptadecane	12398	1699		A	nd		nd	0.06 *	nd	-
92	octadecane	11635	1799		A	nd		nd	0.03 *	nd	-
93	nonadecane	12401	1899		A	nd		nd	0.01 *	nd	-
*Total hydrocarbons*						0.39		0.06	0.62	1.33	
	*Terpenes*										
94	*p*-cymene	7463	1023	1250	A	nd		<0.01 *	0.01 *	0.06	[18]
95	*o*-cymene	10703	1027		A	0.12 *		0.01 *	nd	nd	-
96	d-limonene	440917	1033	1169	A	nd		<0.01 *	0.07 *	nd	-
97	eucalyptol	2758	1037		A	nd		<0.01 *	nd	nd	-
98	β-ionone	638014	1494	1952	A	0.16		nd	nd	0.43	[12,20]
*Total terpenes*						0.28		0.01	0.08	0.49	
	*Other compounds*										
99	allyl isocyanate	15123	645		B	nd		nd	0.02 *	nd	-
100	2-ethylfuran	18554	701		A	0.16		nd	0.02	0.14 *	[3,21]
101	cyclohexyl isocyanate	18502	961		B	nd		0.01 *	nd	nd	-
102	1-isopropyl-3-methoxypyrazine	33166	1093		B	0.25		nd	0.09	nd	[15,25]
103	1,2,3,5-tetramethylbenzene	10695	1124		B	0.06 *		nd	nd	nd	-
104	veratrole	7043	1144		B	nd		nd	0.09 *	nd	-
105	octanoic acid	379	1157		B	0.10		nd	0.06	nd	[14,22]
106	2-*sec*-butyl-3-methoxypyrazine	520098	1172		B	0.15 *		nd	0.43	nd	[25]
107	phenethyl isocyanate	160602	1226	1807	B	nd		nd	0.41 *	0.70 *	-
108	quinoline	7047	1247		B	nd		nd	0.02 *	nd	-
109	caprolactam	7768	1251		B	0.05 *		nd	nd	nd	-
110	4-bromophenol	7808	1283		B	0.02 *		nd	nd	nd	-
111	6-methylquinoline	7059	1331		B	nd		nd	0.01 *	nd	-
112	methyleugenol	7127	1400		B	0.02 *		nd	nd	nd	-
113	benzyl tiglate	250096	1504		B	nd		nd	0.01 *	nd	-
*Total other compounds*						0.77		0.01	0.63	0.14	

^a^ Linear retention index on a HP-5MS and Stabilwax columns. ^b^ A, mass spectrum and LRI agree with those of authentic compound; B, mass spectrum agrees with reference spectrum in the NIST/EPA/NIH mass spectra database and LRI agree with those in the literature, tentatively identified. ^ Based on library identification and spectra but two separate peaks were present; nd, not detected; * newly reported for species.

**Table 3:** Odorants identified by HS-SPME GC-O in the headspace of four Brassicales species.

Compound Number	Compound	LRI ^a^	Odour Description		Odour Intensity ^c^				References
			This Study	Previous Description(s) ^b^	Salad Rocket	Wasabi	Horseradish	Watercress	
114	<unknown>	<600	sulphury	-	nd	3	3	4	-
115	<unknown>	<600	cooked onions	-	nd	nd	nd	4	-
116	<unknown>	<600	buttery	-	3	5	5	5	-
117	<unknown>	<600	sulphury, horseradish, rancid	-	3	6	4	nd	-
118	<unknown>	602	horseradish	-	nd	nd	5	nd	-
119	<unknown>	609	mustard, horseradish	-	nd	6	nd	nd	-
120	<unknown>	615	rotten cabbage	-	nd	nd	5	4	-
121	<unknown>	622	onions	-	nd	6	nd	nd	-
99	allyl isocyanate ^$^	648	musty, burnt plastic †	-	nd	nd	2 *	nd	-
122	<unknown>	655	sulphury, cabbage-like	-	6	nd	nd	nd	-
74	3-butenenitrile ^$^	657	sulphury, green, pungent †	-	nd	3	4	nd	[21]
25	1-penten-3-ol	676	cabbage, sulphury	pungent, horseradish-like	3	nd	nd	7	[40]
65	ethyl vinyl ketone	680	pungent, rotten, green	pungent	5	nd	nd	nd	[41]
66	3-pentanone	687	green, grassy, floral	acetone-like	nd	nd	nd	6	[42]
123	<unknown>	713	sulphury, garlic	-	nd	2	3	nd	-
2	methyl thiocyanate ^$^	719	sulphury, oniony	sulphur	4	nd	nd	4 *	-
124	<unknown>	737	sulphury, rotten onion	-	3	nd	nd	3	-
40	2-pentenal	750	apple, green	green, apple-like	3	nd	3 *	4 *	[43]
125	<unknown>	758	sulphury, cooked onion, pungent	-	nd	nd	nd	6	-
126	<unknown>	775	sulphury, oniony	-	nd	nd	nd	4	-
42	3-hexenal	796	grassy, green, floral	leafy, green	4	nd	3	4	[25]
43	hexanal	799	green, grassy, pungent	green, grass-like, leafy	5 *	nd	4	4 *	[44]
127	<unknown>	811	green, parsley	-	nd	nd	nd	4	-
128	<unknown>	812	mustard, pungent, oniony	-	5	6	3	6	-
3	isopropyl ITC ^$^	834	pungent, grassy notes	pungent	nd	5	nd	nd	[12]
44	(*E*)-2-hexenal	852	green, fresh, apples (weak)	green	3	nd	2	4	[45]
30	(*Z*)-3-hexen-1-ol	856	green, radishy	green	2	nd	nd	4	[46]
32	2-hexen-1-ol	864	green, leafy	green, leafy	3	nd	nd	nd	[47]
129	<unknown>	866	cooked, roasted chicken, chicken soup	-	nd	3	3	5	-
130	<unknown>	870	nutty, spicy	-	nd	nd	3	nd	-
4	allyl thiocyanate ^$^	879	peppery, horseradish, pungent	strong, pungent, mustard-like	nd	nd	4	nd	[48]
131	<unknown>	892	green, sour apples	-	5	nd	nd	6	-
5	allyl ITC ^$^	895	pungent, horseradish	strong, pungent, mustard-like	nd	nd	4	nd	[48]
6	allyl ITC ^$^	898	garlic, mustard, horseradish, very pungent		nd	7	7	nd	
7	cyclopropane ITC ^$^	900	horseradish, garlic, onion, sulphur †	-	nd	5 *	7 *	nd	[49]
45	4-heptenal	901	grassy, green †	mushroom-like, fatty, fishy, cooked, potato	4 *	nd	nd	3 *	[50]
46	heptanal	902	fatty, green	fatty, green	4	nd	nd	5 *	[13]
132	<unknown>	907	apples, grass	-	nd	nd	nd	4	-
47	2,4-hexadienal ^$^	907	green, rotten	green	3	nd	3 *	nd	[51]
57	(*Z*)-2-pent-2-en-1-yl acetate ^$^	914	sulphury, rotten †	-	nd	nd	nd	3 *	-
8	cyclopentyl-1-thiaethane	918	sulphur, sweaty †	-	nd	nd	nd	3 *	-
133	<unknown>	921	potato	-	nd	5	nd	4	-
9	*sec*-butyl ITC ^$^	934	radish, vegetative	green	nd	5	3	nd	[52]
10	isobutyl ITC ^$^	956	cooked, pungent, sulphury †	-	nd	4	5	nd	[21]
101	cyclohexyl isocyanate ^$^	965	cooked, peppery, potato †	-	nd	5 *	nd	nd	[53]
34	1-octen-3-ol	978	mushroom	mushroom	5	5 *	3 *	5 *	-
11	3-butenyl ITC ^$^	983	green, pungent, aromatic	aromatic, pungent	5	6	4	nd	[54]
68	6-methyl-5-hepten-2-one	984	perfume, floral, citrus	citrus, lemongrass	4	nd	nd	5 *	[55]
12	butyl ITC ^$^	997	peppery, sulphurous, oniony	sulphury	nd	4	2	nd	[21]
77	thiiraneacetonitrile ^$^	1005	sweaty, gas-like †	-	nd	nd	4	nd	[56]
134	<unknown>	1010	grassy	-	nd	nd	nd	3	-
135	<unknown>	1020	earthy, musty, petrol, aromatic	-	nd	nd	nd	5	-
136	<unknown>	1025	bread-like	-	nd	nd	nd	5	-
137	<unknown>	1032	green	-	nd	nd	nd	3	-
96	*d*-limonene	1034	lemon, vegetable	citrus, herbal	nd	nd	3 *	nd	[57]
36	benzyl alcohol	1037	fruity, medicinal, wine	phenolic	nd	nd	4 *	nd	[58]
97	eucalyptol	1037	eucalyptus, mint	eucalyptus	nd	5 *	nd	nd	[42]
69	2,2,6-trimethylcyclohexanone ^$^	1038	floral, green with citrus notes	thujonic	nd	nd	nd	4 *	[59]
52	phenylacetaldehyde	1044	honey-sweet	honey, sweet	nd	nd	4	7 *	[21]
138	<unknown>	1051	oniony	-	5	nd	nd	nd	-
139	<unknown>	1053	soily, earthy	-	6	nd	nd	nd	-
13	isoamyl ITC ^$^	1057	pungent, grassy	green	nd	6 *	3	nd	[3]
140	<unknown>	1066	medicinal, floral	-	nd	nd	nd	5	-
141	<unknown>	1076	gas, sulphur, burnt, roasted	-	3	nd	5	4	-
142	<unknown>	1080	roasted, smoky	-	3	nd	nd	3	-
14	4-pentenyl ITC ^$^	1084	pungent, peppery, sulphurous, musty	mustard, horseradish-like	nd	5	3	nd	[48]
71	3,5-octadien-2-one ^$^	1090	green, pungent	fruity	nd	nd	4 *	nd	-
102	2-isopropyl-3-methoxypyrazine ^$^	1091	rotten, potato, vegetative	pea-like, earthy, bean-like	6	nd	6	nd	[60]
15	pentyl ITC ^$^	1095	cabbage, green, rotten	green	5 *	4 *	6 *	nd	-
143	<unknown>	1095	cucumber, floral, flowers	-	nd	nd	nd	3	-
54	nonanal	1101	fatty, green	green, fatty	nd	nd	4	5 *	[40]
37	2-phenylethanol	1114	floral	floral	3 *	nd	nd	4 *	[58]
144	<unknown>	1118	spicy, chemical	-	3	nd	nd	nd	-
145	<unknown>	1122	sulphury, gas-like	-	4	nd	2	nd	-
146	<unknown>	1133	sulphury, garlic	-	nd	3	2	nd	-
147	<unknown>	1147	petrol, aromatic	-	nd	nd	nd	6	-
148	<unknown>	1153	cucumber	-	nd	nd	nd	6	-
149	<unknown>	1153	spicy, cinnamon-like, nutty	-	nd	6	nd	nd	-
150	<unknown>	1155	fresh cucumber, rotten, vegetable	-	2	5	nd	nd	-
16	1-isothiocyanato-4-methylpentane ^$^	1162	musty †	-	2	4 *	2	nd	-
106	2-*sec*-butyl-3-methoxypyrazine ^$^	1173	earthy, rotten potatoes, vegetable-like	musty, green, pea-like, bell pepper-like	3 *	nd	7	nd	-
59	(*Z*)-3-hexenyl butanoate	1181	green, wine-like	green, fruity	4	nd	nd	nd	[61]
18	<unidentified ITC>	1192	radish, green	-	nd	4	nd	nd	-
151	<unknown>	1198	grassy, fruity, chemical, dried fruit	-	nd	nd	nd	6	-
79	5-(methylsulfanyl)pentanenitrile ^$^	1200	radish	broccoli-like, cabbage-like	2	nd	nd	nd	[62]
152	<unknown>	1200	sweet, floral, violets, perfume	-	-	3	4	-	-
60	methyl salicylate	1201	medicinal, camphorous	wintergreen, mint	nd	nd	4 *	nd	[40]
153	<unknown>	1203	peppery, green, earthy	-	nd	5	nd	5	-
107	phenethyl isocyanate	1224	ground pepper, pungent, horseradish †	-	nd	nd	4 *	nd	-
81	benzenepropanenitrile ^$^	1242	herbal, green, floral †	-	nd	3	3	4	[21]
154	<unknown>	1249	liquorice, medicinal	-	nd	4	nd	nd	-
155	<unknown>	1266	cooked, cabbage, sulphur	-	nd	nd	3	nd	-
156	<unknown>	1275	green, radish, potato	-	nd	nd	nd	4	-
157	<unknown>	1279	soapy, pungent	-	nd	nd	nd	5	-
158	<unknown>	1285	soapy, grassy, floral	-	nd	2	nd	6	-
19	ibervirin ^$^	1314	horseradish, radish, vegetative	vegetative, horseradish-like, gooseberry-like	nd	3	5	nd	[63]
159	<unknown>	1318	medicinal, soapy	-	nd	nd	nd	5	-
111	6-methylquinoline ^$^	1334	hydrogen sulphide, egg	tobacco, fecal	nd	nd	3 *	nd	-
20	sativin ^$^	1349	burnt, rubbery, soily †	rocket-like	3	nd	nd	nd	[12]
21	octyl ITC ^$^	1370	green, vegetative †	-	nd	nd	nd	5 *	[64]
22	benzyl ITC ^$^	1378	rotten grass, cooked	watercress-like	nd	3 *	4	-	[48]
61	ethyl decanoate	1391	green, waxy	waxy, apple	nd	nd	4 *	nd	[65]
23	erucin ^$^	1441	radishy	radish-like, cabbage-like	2	nd	nd	nd	[12]
160	<unknown>	1478	minty, cooling, fresh	-	nd	nd	4	nd	-
24	phenethyl ITC ^$^	1480	radish, gooseberry, sweet	horseradish-like, gooseberry-like	nd	nd	6	7	[48]
98	β-ionone	1495	soapy, fusty	floral, woody, fruity	2	nd	nd	6	[41]
113	benzyl tiglate ^$^	1500	musty	earthy, mushroom-like	nd	nd	4 *	nd	-

^a^ Linear retention index on a HP-5MS column; for identification please check Table 2. ^b^ Odour description of compound present in the Good Scents online database: http://www.thegoodscentscompany.com/ (accessed on 1 April 2021) and literature sources; † tentative new odour description. ^c^ Average of intensities recorded by three assessors evaluating each sample in duplicate (scoring scale: weak = 3, medium = 5, strong = 7); nd, not detected; * newly reported for species; ^$^ compound tentatively identified.

#### 3.1.1. ‘Salad’ Rocket

A total of 57 volatile compounds were identified or tentatively identified in the headspace of *E. sativa* leaf samples. Nineteen compounds are newly reported for this species, some of which make up relatively large portions of the total volatile compounds’ bouquet (Table 2). Compounds with the greatest relative abundances were (*Z*)-3-hexen-1-ol (**31**, 40.9%), (*E*)-2-hexenal (**44**, 14.3%), hexanal (**43**, 8.6%), erucin (**23**, 7.2%), and 1-isothiocyanato-4-methylpentane (**16**, 5%; Figure 2). These observations are broadly in agreement with previous studies of ‘salad’ rocket [3,12].

Despite its high relative abundance, (*Z*)-3-hexen-1-ol produced only a weak, green, radishy aroma (Table 3) in rocket leaves. (*E*)-2-Hexenal by comparison was 2.9-fold less abundant in relative terms but produced a slightly stronger aroma, described by assessors as green, and apple-like. Hexanal (**43**) produced a pungent, green, grassy aroma of relatively high intensity, which has not been previously described in rocket to our knowledge.

2-Isopropyl-3-methoxypyrazine (**102**) by comparison was of low relative abundance in the rocket headspace (0.3%; Table 2) but was found to have one of the strongest aromas in rocket (rotten, potato-like, vegetative; Table 3). 1-Isothiocyanato-4-methylpentane had a weak aroma and was given a tentative new description of ‘musty’, as no previous studies have reported an odour for this compound.

The ITC erucin (**23**) is known for its anticarcinogenic properties, but its aroma was only recently described [12]. In agreement with a previous report, this compound produced a radishy aroma of weak intensity. Interestingly, the compound previously associated with characteristic “rocket-like” aroma (sativin, **20**) [12] was relatively weak-smelling. In this study, the compound was found to have a burnt, rubbery, and soil-like aroma. This suggests that sativin is not the main driver of pungency or aroma in ‘salad’ rocket. Other compounds such as ethyl vinyl ketone (**65**; [41]), hexanal, 3-butenyl ITC (**11**) [48,54], and several unknown compounds (see final paragraph in this section) all had descriptions of pungency at higher intensities than sativin (Table 3). It may also be likely that no single compound is responsible for this attribute of rocket aroma but rather several.

Pentyl ITC (**15**, 0.6%) not previously identified in ‘salad’ rocket produced a strong odour, which was characterised as cabbage-like, green, and rotten (Table 3, Figure 2). As will be discussed in Section 3.2, we have tentatively identified pentyl GSL (Table 1) as a significant and previously unreported component of the GSL profile of ‘salad’ rocket, which gives rise to this ITC compound.

Other compounds not previously identified in ‘salad’ rocket included 2-phenylethanol, 4-heptenal and 2-*sec*-butyl-3-methoxypyrazine. 2-Phenylethanol (**37**, 0.2%) was noted to impart a floral aroma at a medium-weak intensity (Table 3). This compound is derived from phenylalanine and has been found to contribute to aroma and flavour in many foods, such as tomatoes [66]. 4-Heptenal (**45**) occurred in rocket leaves with a relative abundance of 0.1% (Table 2). Despite this low amount in terms of the overall volatile profile, the compound was perceived at a medium intensity by the assessors (Table 3) and described as grassy and green. This compound has been variously described as mushroom-like [50], fatty and fishy [67], and potatoey [68]. The variation in these descriptions may be associated with the isomerisation of the compound, which could not be resolved in this study. 2-*sec*-Butyl-3-methoxypyrazine (**106**, 0.2%) has been reported in several Brassicales species, such as white mustard, rapeseed [60], and horseradish [25], but not in *E. sativa* (Figure 2). It has been variously described as having a pea-like, musty, green, and bell pepper-like aroma. Assessors described the compound as earthy, similar to rotten potatoes, and vegetable-like. Despite its relatively low abundance, it was perceived as a medium-weak smelling compound in the sample headspace.

A total of 13 unidentified odorants were also detected in the headspace of rocket by GC-O (Table 3). These varied in intensity but all were distinguished and reported by assessors. Odour descriptions for these compounds were buttery (**116**), sulphury (**117**, **122**, **124**, **141**, **145**), horseradish-like, rancid (**117**), cabbage-like (**122**), rotten onion (**124**), mustard, pungent (**128**), oniony (**128**, **138**), green, sour apples (**131**), soily, earthy (**139**), gas (**141**, **145**), burnt (**141**), roasted (**141**, **142**), smoky (**142**), spicy, chemical (**144**), fresh cucumber, rotten, and vegetable-like (**150**).

#### 3.1.2. Wasabi

A total of 43 compounds were identified or tentatively identified in the headspace of wasabi roots (Table 2) with 30 compounds newly described for the species, making this a significant step forward in the understanding of aroma composition in wasabi roots. Two peaks of near-identical spectra were observed and identified as allyl ITC (**5/6**, 8.6%/52.1%) and were the most abundant compounds, which agrees with previous observations [7]. It is unknown why two distinct peaks were formed in this manner, and further investigation may be required to determine the isomeric differences responsible for the separation. 4-Pentenyl ITC (**14**, 12.4%) was also found to be high in terms of overall relative abundance.

Despite having near identical spectra, compounds **5** and **6** presented distinct differences in aroma and intensity. Peak 6 was characterised as being very pungent (Table 3) and having garlic, mustard, and horseradish-like qualities. Allyl ITC is one of the most well characterised ITCs and is well known for these properties [1,48,63] (Figure 2). Peak **5** by contrast had no discernible aroma in wasabi but was apparent in horseradish (see Section 3.1.3). 4-Pentenyl ITC likewise exhibited a pungent aroma and strong odour intensity but also had peppery, sulphurous, and musty notes. This compound is commonly reported in Brassicaceae crops [69]. 3-Butenyl ITC (**11**, 4.2%) was scored as a high odour intensity compound, despite its much lower relative abundance and was described as having a pungent, green, and aromatic odour.

Several other GHP odorants are newly reported in wasabi including cyclopropane ITC, isoamyl ITC, pentyl ITC, 1-isothiocyanato-4-methylpentane, and benzyl ITC. Cyclopropane ITC (**7**, 0.1%) is likely to be a cyclic reaction product of allyl ITC and has been previously reported in brown mustard [49] (Figure 2). To our knowledge, no odour description of this compound has been previously made, but assessors described it as sulphurous, horseradish, garlic, and onion-like (Table 3). The high odour intensity score indicates that it is a significant component of wasabi aroma. Isoamyl ITC (**13**, 0.4%) was described as having a pungent grassy aroma and being of high odour intensity. This compound is not commonly reported in Brassicales species, but it is used as a food additive [70]. Most ITC compounds are noted for their sulphurous and mustard-like potency in Brassicales; however, the contribution of grassy aroma ITCs to volatile compound bouquets has not been previously appreciated or fully understood. Pentyl ITC (**15**, 0.1%) and 1-isothiocyanato-4-methylpentane (**16**, <0.1%) were observed and shared the same odour characteristics as in ‘salad’ rocket (see Section 3.1.1.). Benzyl ITC (**22**, <0.1%) was reported to have a rotten grass and cooked aroma of medium-weak intensity. Similar to isoamyl ITC, this compound is not regularly reported as a constituent of Brassicales headspace, but these data indicate that even in very low relative abundance, it is odour active.

Another interesting compound was also found in wasabi headspace: cyclohexyl isocyanate (**101**, <0.1%; Figure 2). This has been previously reported in black mustard [53], though it is unclear if it is related to or derived from GHPs. Assessors perceived this odour having a medium-strong intensity and described it as peppery, cooked, and potato-like (Table 3). This is a tentative new odour description for this compound, and our data suggest it to be an important constituent of wasabi aroma.

Two additional compounds not previously identified in wasabi were 1-octen-3-ol and eucalyptol. 1-Octen-3-ol (**34**, <0.1%), despite its very low relative intensity in root tissue headspace (Table 2, Figure 2), exhibited a high odour intensity imparting a mushroom-like odour in agreement with previous descriptions [50]. Eucalyptol (**97**, <0.1%), previously observed in Brassicales crops [18] but not in wasabi (Figure 2), was found to have a medium-strong, characteristic eucalyptus, and mint aroma, and it is likely an important component of the overall volatile bouquet.

Similar to rocket, 17 unidentified odorants were detected in wasabi root samples (Table 3). Reported aromas were sulphury (**114**, **117**, **123**, **146**), buttery (**116**), horseradish-like (**117**, **119**), rancid (**117**), mustard (**119**, **128**), onion (**121**, **128**), garlic (**123**, **146**), pungent (**128**), cooked, roasted chicken, chicken soup (**129**), potato (**133**), spicy, cinnamon-like, nutty (**149**), fresh cucumber, rotten, vegetable-like (**150**), radish (**79**), green (**79**, **153**), sweet (**152**), floral (**152**, **158**), violets, perfume (**152**), peppery, earthy (**153**), liquorice, medicinal (**154**), soapy, and grassy (**158**), confirming our statement that wasabi’s volatile profile is far from complete.

#### 3.1.3. Horseradish

A total of 75 compounds were identified or tentatively identified in the headspace of horseradish roots, 38 of which are newly reported (Table 2). As with wasabi, the peaks with the highest relative abundances were dominated by GHPs. Compounds with the highest relative abundances were allyl ITC (**5/6**, 7.4%/39.3%), phenethyl ITC (**24**, 32.5%), thiiraneacetonitrile (**77**, 3.6%), 4-pentenyl ITC (**14**, 2.3%), (*Z*)-3-hexen-1-ol (**31**, 1.9%), and *sec*-butyl ITC (**9**, 1.8%; Figure 2).

As stated in Section 3.1.2, allyl ITC was identified as two distinct peaks (**5** and **6**). As in wasabi, **6** was of the greatest abundance and odour intensity, producing a very strong, pungent, garlic, mustard, and horseradish-like aroma (Table 3), whereas **5** produced a medium intensity, pungent horseradish smell. Thus far, the presence of two peaks has not been addressed or explained satisfactorily within the literature, with only one previous paper reporting the same phenomenon of separate and distinct allyl ITC peaks [71]. *Sec*-butyl ITC (**9**) produced a medium-weak intensity aroma that was vegetative and radish-like (Table 3). The compound was also present in wasabi at a medium intensity. The compound has been previously reported in horseradish as having green, chemical, and mustard like aromas [21], and it is known to activate the human Transient Receptor Potential Ankyrin 1 (TRPA1). This receptor is known to act in response to environmental irritants, and several ITCs identified in this study are known to activate it to varying degrees (isopropyl ITC, **3**; isobutyl ITC, **10**; allyl ITC, **5/6**; 3-butenyl ITC, **11**; 4-pentenyl ITC, **14**; benzyl ITC, **22**; phenylethyl ITC, **24**; [72]). Phenylethyl ITC (**24**) is known to be a key constituent of horseradish aroma, and our data are in agreement with previous reports [73]. Assessors described the compound as radish and gooseberry-like, with a sweet note. It had a high odour intensity and contributed significantly to the odour profile of roots. Likewise, 4-pentenyl ITC (**14**) was observed to have the same odour attributes as previous reports [1] and those found for wasabi in this study, but at a lower intensity. By contrast, pentyl ITC (**15**) was present at much lower relative intensities to other GHPs (0.2%, Table 2) but produced a strong, green, rotten, and cabbage-like aroma.

Thiiraneacetonitrile (**77**) has been previously reported in horseradish [22] and is an epithionitrile hydrolysis product of sinigrin. To our knowledge, no previous studies have described the odour of this compound. We found it to have a sweaty, gas-like aroma of medium intensity (Table 3).

2-Isopropyl-3-methoxypyrazine (**102**, 0.1%) and 2-*sec*-butyl-3-methoxypyrazine (**106**, 0.4%) have been previously described and characterised in horseradish roots [25] as having green and pepper-like aromas. Our data agree with previous reports but found the compounds to be of very high aroma intensity, despite relatively low abundances within the headspace (Table 3). Assessors described the compounds as rotten, earthy, potato-like, and vegetative.

We report several compounds previously unidentified in horseradish including GHPs, isocyanates, alcohols, aldehydes, and a ketone and ester. As in wasabi root, cyclopropane ITC (**7**, 0.1%) produced an intense aroma containing horseradish, garlic, onion, and sulphur notes (Table 3). Therefore, it is likely to be a significant contributor to root odour and the volatile profile, despite its very low abundance, which may be a reason why it has not been previously detected and/or reported.

As discussed in Section 3.1.2, it is unknown if the presence of isocyanates is linked with GSLs and their hydrolysis products. Allyl isocyanate (**99**, <0.1%) and phenethyl isocyanate (**107**, 0.4%) were both observed for the first time in horseradish roots. Given that high abundances of allyl ITCs (**5/6**) and phenethyl ITC (**24**) were observed, it seems likely that isocyanates may be derived from them and/or directly from parent GSLs. Isocyanates are not commonly reported in the literature, and their formation may be because of as-yet-unstudied enzymatic or post-hydrolysis modification processes. Allyl isocyanate was described as having a weak musty and burnt plastic aroma; and phenethyl isocyanate was described as being pungent, with ground pepper and horseradish-like quality at a medium intensity. We are not aware of any previous odour descriptions for these compounds, so these are tentative new characterisations.

1-Octen-3-ol (**34**, <0.1%) was identified, and as in wasabi root, it produced a mushroom-like aroma of medium-weak intensity (Table 3). Benzyl alcohol (**36**, 0.1%) has been previously reported in *Brassica oleracea* [74] and rapeseed [58], but not in horseradish. It was characterised as having a medium intensity aroma, described as fruity, medicinal, and wine-like. Its low abundance but relatively high odour intensity may make it a subtle but key constituent of the root aroma profile. Two aldehyde compounds were also found to contribute to odour within the headspace. 2-Pentenal (**41**, <0.1%) produced a green, apple-like aroma [43], and 2,4-hexadienal (**47**, <0.1%) produced a green, rotten smell, both of medium-weak intensity (Table 3; [51]). A ketone, 3,5-octadien-2-one (**71**, 0.1%) was also tentatively identified (Table 2) and described as having a pungent green aroma of medium intensity (Table 3).

In the esters group, methyl salicylate (**60**, <0.1%), a common compound throughout the plant kingdom, has previously been identified in Brassicales as part of systemic acquired resistance response to herbivory [75], and it was identified for the first time as a volatile constituent of horseradish headspace (Table 2). Its aroma is characteristic of, and present in, plants such as wintergreen. Assessors described its odour as medicinal and camphorous at a medium intensity (Table 3). Ethyl decanoate (**61**, <0.1%), known to be present in *B. oleracea* [59], was described by the assessors as green and waxy (Table 3; [65]). Benzyl tiglate (**113**, <0.1%) was reported at low relative abundance, but a perceptible musty aroma was apparent for this compound.

The presence of *D*-limonene (**96**, 0.1%) is reported for the first time in horseradish root. It is known to be a constituent of *B*. *oleracea* headspace [76], but its sensory contribution to Brassicales is not well defined. Assessors found this compound to have a medium-weak intensity aroma of lemon and being vegetable-like (Table 3 [57]). This agrees with previous descriptions of the odour properties of the compound.

Finally, 6-methylquinoline (**111**, <0.1%), an aromatic compound, produced an unpleasant hydrogen sulphide and egg-like aroma of medium-weak intensity, and it has not been previously reported.

Similar to the other species tested, 15 unidentified odorants were detected in horseradish root samples (Table 3) and were of weak to medium intensity. Reported aromas were sulphury (**114**, **117**, **123**, **141**, **145**, **146**, **155**), buttery (**116**), horseradish-like (**117**, **118**), rancid (**117**), rotten (**120**), cabbage-like (**120**, **155**), garlic, (**123**, **146**), mustard, pungent, oniony (**128**), cooked (**129**, **155**), roasted chicken, chicken soup (**129**), nutty, spicy (**130**), gas (**141**, **145**), burnt, roasted (**141**), sweet, floral, violets, perfume (**152**), minty, cooling, and fresh (**160**).

#### 3.1.4. Watercress

A total of 42 compounds were identified or tentatively identified in the headspace of watercress, 26 of which are newly reported (Table 2). The headspace profile was dominated by alcohol and ITC compounds: (*Z*)-3-hexen-1-ol (**31**, 36%), phenethyl ITC (**24**, 30.7%), 1-penten-3-ol (**25**, 8.1%), and (*Z*)-2-penten-1-ol (**28**, 4.8%; Figure 2).

1-Penten-3-ol is a compound present widely in Brassicales species [77]. It exhibited a high intensity in watercress leaves, producing a sulphurous and cabbage-like aroma. These attributes are often attributed to ITCs and other sulphur-containing compounds; however, our data suggest that some of these characteristics in watercress could be attributed to this alcohol. (*Z*)-3-Hexen-1-ol by comparison was much higher in relative abundance but produced a medium intensity aroma that was green and radishy (Table 3).

Phenethyl ITC (aroma attributes described in Section 3.1.2.) had one of the highest intensity aromas in watercress, along with phenylacetaldehyde (**52**, 0.2%). The latter, similar to phenethyl ITC, is derived from phenylalanine, but occurs in much lower abundance (Table 2, Figure 2). Its aroma was described as honey-sweet (Table 3) and is likely a significant contributor to watercress odour that has previously gone unrecognised.

Other compounds contributing high odour intensities despite low relative abundances were 3-pentanone (**66**, 1.9%) and β-ionone (**98**, 0.4%; Figure 2). 3-Pentanone is regularly reported in Brassicales [3] and was described as high intensity, green, grassy, and floral smelling (Table 3, Figure 2). β-Ionone is common to many plant species as a degradation product of carotenoids [78], and it was described as soapy and fusty by assessors, with a high intensity.

Several sulphur-containing compounds, aldehydes, alcohols, ketones, not previously identified in watercress were identified. Methyl thiocyanate (**2**, 0.5%) imparted a sulphury and oniony note, with a medium intensity. This compound is known to be a GSL hydrolysis product of GCP (methyl GSL), but as will be discussed in Section 3.2, this compound was not detected in the UPLC-MS/MS analysis. Therefore, we suggest that it is not directly derived from this GSL and may be a degradation product of other GSL hydrolysis products within the tissues and headspace of the tested Brassicaceae.

Cyclopentyl-1-thiaethane (**8**, 0.5%) was detected, and uniquely present in watercress compared with the other three species tested (Table 2). Little is known about this compound in a biological context. It produced a sweaty, sulphury, medium-weak intensity aroma that is tentatively described in this species for the first time (Table 3).

Five aldehydes, two alcohol and two ketones, are newly reported for watercress, which produced perceptible odours within the headspace bouquet: 2-pentenal (**41**, 0.5%), hexanal (**43**, 1.2%), 4-heptenal (**45**, 0.5%), heptenal (**46**, 0.1%), and nonanal (**54**, 0.3%). Both heptenal and nonanal exhibited fatty, green aromas of medium intensity (Table 3) and are common to other Brassicaceae species [77]. 1-Octen-3-ol (**34**, 0.1%) produced a medium strength mushroom-like aroma (as described in Section 3.1.3.), and 2-phenylethanol (**37**, 2.6%) produced a floral scent. 6-Methyl-5-hepten-2-one (**68**, 0.2%) has been previously observed in ‘wild’ rocket and described as having a citrus aroma [55,79]. In this study, it was also identified with this characteristic, but also as floral and perfume-like, in both watercress and ‘salad’ rocket. It produced a medium-strong aroma in watercress. 2,2,6-Trimethylcyclohexanone (**69**, 0.1%) has been variously described as thujonic, menthol-like, and camphorous [80]. Here, it was described by assessors as imparting floral and green odours, with citrus notes.

One ester, (*Z*)-pent-2-en-1-yl acetate (**57**, 0.1%), was only detected in watercress, and it produced a medium-weak aroma. It was described as sulphury and rotten, and we are not aware of any previous odour attributes associated with this compound. As such, this is a tentative first description.

Octyl ITC (**21**, 0.2%) has been previously identified in horseradish [64] but not watercress to our knowledge. Its exact derivation and parent GSL are unclear in the literature, though watercress has been reported to contain glucohirsutin (GHS, (*RS*)-8methylsulfinyl)octyl GSL; [38]). This will be discussed further in Section 3.2 and Section 3.3. Assessors found the compound to be of medium aroma strength having a green and vegetative character. Again, we are unaware of previous odour descriptions for this compound.

Finally, there were 29 unidentified odorants detected by GC-O, making this the highest number of the four species analysed (Table 3). Aromas described by assessors were sulphury (**114**, **124**, **125**, **126**, **141**), cooked onions (**115**, **125**), buttery (**116**), rotten, cabbage-like (**120**), rotten onion (**124**), pungent (**125**, **128**, **157**), oniony (**126**, **128**), green (**127**, **131**, **137**, **153**, **156**), parsley (**127**), mustard (**128**), cooked, roasted chicken, chicken soup (**129**), sour apples (**131**), apples (**132**), grass (**132**, **134**, **151**, **158**), potato (**133**, **156**), earthy (**135**, **153**), musty (**135**), petrol, aromatic (**135**, **147**), bread-like (**136**), medicinal (**140**, **159**), floral (**140**, **143**, **158**), gas, burnt, roasted (**141**, **142**), smoky (**142**), cucumber (**143**, **148**), flowers (**143**), fruity, chemical, dried fruit (**151**), peppery (**153**), radish (**156**), and soapy (**157**, **158**, **159**). This indicates that the volatile profile of watercress is far from complete, and further research is required to elucidate these compounds.

### 3.2. Non-Volatile Compounds (Glucosinolates)

GSL composition and concentrations for ‘salad’ rocket, wasabi, and watercress are presented in Table 4. Due to an unforeseen termination of supply, it was not possible to include horseradish roots in this analysis.

#### 3.2.1. ‘Salad’ Rocket

‘Salad’ rocket contained the highest dry weight concentrations of GSLs (111.1 ± 14.6 µmol g^−1^ dw). This was predominantly due to high amounts of DMB (78.9 ± 8.4 µmol g^−1^ dw). Other GSLs of note included GSV (6.6 ± 2.4 µmol g^−1^ dw), GRM (7.6 ± 1.2 µmol g^−1^ dw), and DGTB (4.6 ± 1.1 µmol g^−1^ dw), which are unique to the genera *Eruca* and *Diplotaxis*. Other routinely reported GSLs for this species were GRA (1.8 ± 0.5 µmol g^−1^ dw) and NGB (2.7 ± 0.4 µmol g^−1^ dw).

Interestingly, several other GSLs that have not been, or are rarely reported for the species, were also detected; some in relatively high concentrations: GIB (<0.1 ± <0.1 µmol g^−1^ dw), PEN (4.9 ± 1.6 µmol g^−1^ dw), GPJ (<0.1 ± <0.1 µmol g^−1^ dw), GBT (0.4 ± <0.1 µmol g^−1^ dw), GTP (<0.1 ± <0.1 µmol g^−1^ dw), 4MP (1.3 ± 0.8 µmol g^−1^ dw), HEX (0.2 ± <0.1 µmol g^−1^ dw), and BUT (0.3 ± <0.1 µmol g^−1^ dw).

Of note is the high abundance of PEN (*m/z* 388). It seems unlikely that a GSL of such relatively high concentration has gone undetected in previous analyses. Therefore, we postulate that previous studies may have attributed the negative ion mass incorrectly to that of PRO, which is also *m/z* 388 (Table 1). The authentic standard of PRO did not match the retention time or MS/MS spectra of PEN, and it was found in only very low concentrations by comparison. While PEN is only a tentative identification (due to the possibility of other isomeric GSLs such as glucojiaputin and 3-methylbutyl GSL), the presence of pentyl ITC (**17**) within the headspace of rocket makes this the most likely identification. See Section 3.3 for further discussion.

#### 3.2.2. Wasabi

Sixteen GSL compounds were identified in wasabi roots, totaling 27.5 ± 2.8 µmol g^−1^ dw (Table 4). The most abundant compound was SIN (11.1 ± 0.2 µmol g^−1^ dw), which agrees with previous studies [81]. Wasabi is known to have a diverse GSL profile, and we observed relatively high abundances for ISO (3.4 ± 0.8 µmol g^−1^ dw), GAL (2.1 ± 0.5 µmol g^−1^ dw), GPJ (2.3 ± 0.5 µmol g^−1^ dw), GCL (2.2 ± 0.2 µmol g^−1^ dw), 7MSH (4.6 ± 0.4 µmol g^−1^ dw), and NGB (1.5 ± 0.2 µmol g^−1^ dw). Other compounds occurring in low abundance that are not frequently reported were GIB and GRA.

#### 3.2.3. Watercress

Fourteen GSLs were found in watercress leaves, amounting to 31.6 ± 1.4 µmol g^−1^ dw (Table 4). In most previous studies of this species, GNT (1.5 ± <0.1 µmol g^−1^ dw) has been found to have the greatest abundance [4]; however, our analysis revealed that 7MSH had the highest total concentration (21.5 ± 1.2 µmol g^−1^ dw), dominating the overall profile in these samples. There were also relatively high concentrations of 7MTH (2.7 ± 0.1 µmol g^−1^ dw), and the indolic GSLs 4MGB (2 ± 0.1 µmol g^−1^ dw), and NGB (1.6 ± <0.1 µmol g^−1^ dw). Minor amounts of SIN, GRA, and GTP were also observed, and they are not frequently reported in this species.

### 3.3. Discrepancies between Identified Glucosinolate Hydrolysis Products and Glucosinolate Profile Precursors

There is often an ‘elephant in the room’ regarding volatile GSL hydrolysis products and reported GSL profiles in Brassicales crops: there are often GSLs found with no corresponding hydrolysis products, or more troublingly, hydrolysis products observed but no GSL precursor. Table 5 presents a list of the GSL-derived compounds identified within the headspace of ‘salad’ rocket, wasabi, and watercress, alongside their expected GSL precursors. It is apparent from our data that the present study is no exception when it comes to discrepancies of this nature, and there is a need to find a robust solution to prevent the inaccurate reporting of both GSLs and their volatile hydrolysis products.

As discussed in previous sections, compounds such as methyl thiocyanate (**2**) may be produced from degradation of other hydrolysis products. Others such as the presence of *sec*-butyl ITC (**9**) in rocket, butyl ITC (**12**) in wasabi, and octyl ITC (**21**) in watercress cannot be so easily explained. There are several explanations with varying levels of likelihood: firstly, the most likely is that the ITCs and other GSL hydrolysis products have been identified incorrectly, and that they belong to other parent GSLs present within the analysed tissues. Despite researchers’ best efforts to obtain authentic standards to test and match MS profiles, this is not always possible or affordable, and so there is a heavy reliance upon reference libraries. These are often incomplete and not always accurate. It is also possible that the high temperatures utilised in GC-MS cause thermolytic reactions to occur in GHPs, thus yielding compounds not ‘naturally’ produced by tissues. The next possibility is that the GSLs responsible for producing hydrolysis products are below the LOD of the MS/MS method. In this case, this is unlikely, as the LOD for each standard GSL was low, and it is likely much more sensitive and accurate than volatile compound measurements by GC-MS. Thirdly and perhaps least likely is that there is some as-yet-unknown mechanism(s) by which GSL hydrolysis products are modified post-hydrolysis. This is speculation, but there have been recent reports of previously unknown tautomeric rearrangements [82] and enzymatic actions [28] upon hydrolysis products that do not preclude this as an impossibility. Indeed, the dynamics of reactions occurring within the headspace of Brassicales is virtually unstudied, and so some may be produced through the degradation or rearrangement of structurally similar compounds. This is an area that requires much more detailed scrutiny.

## 4. Conclusions

This study has highlighted numerous newly identified and tentatively identified volatile compounds present in the headspace of ‘salad’ rocket, wasabi, horseradish, and watercress. Many of these appear to contribute strongly to the aroma profiles of each respective crop. We have also found 46 aroma traits present in the headspace of the samples that have no association with the identified compounds. This suggests that there are many as-yet undiscovered odour-active compounds present within Brassicales headspace.

Our data also highlight the need for more detailed studies on the volatilome of Brassicales species, and that the sole focus should not be upon GSL hydrolysis products. While accounting for a large proportion of the respective volatile profiles and odour active components of species, we have identified numerous instances where non-GSL derived compounds have odour intensities greater than those that are GSL-derived, such as 1-penten-3-ol (**25**), phenylacetaldehyde (**52**), 2-*sec*-butyl-3-methoxypyrazine (**106**), and β-ionone (**98**). Several non-GSL derived compounds also share similar pungent odour characteristics with GHPs, indicating that the latter may not be the only source of these sensations in Brassicales crops. There is also a clear need for the improvement of mass spectral libraries and the availability of GSL and GHP standards in order to overcome discrepancies between GSL profiles and the reported volatiles derived therefrom.

## Figures and Tables

**Table 4:** Glucosinolate concentrations of ‘salad’ rocket, wasabi, and watercress determined by UPLC-MS/MS.

Glucosinolate ^a^	Abbreviation	Concentration ^b^		
		Salad Rocket	Wasabi	Watercress
glucoiberin	GIB	0.007 ± 0.001	0.003 ± <0.001	nd
pentyl GSL ^$^	PEN	4.915 ± 1.633	nd	nd
progoitrin	PRO	0.074 ± 0.067	0.001 ± <0.001	nd
sinigrin	SIN	0.002 ± 0.001	11.121 ± 0.247	0.001 ± <0.001
isobutyl GSL ^$^	ISO	nd	3.382 ± 0.762	nd
glucoraphanin	GRA	1.761 ± 0.508	0.001 ± <0.001	0.006 ± <0.001
glucorucolamine	GRM	7.571 ± 1.208	nd	nd
glucoalyssin	GAL	0.215 ± 0.044	2.123 ± 0.477	0.470 ± 0.026
glucoputranjivin ^$^	GPJ	0.058 ± 0.024	2.289 ± 0.515	nd
gluconapin	GNP	0.001 ± 0.001	0.010 ± 0.001	nd
diglucothiobeinin	DGTB	4.622 ± 1.144	nd	nd
glucoberteroin	GBT	0.412 ± 0.086	nd	nd
4-hydroxyglucobrassicin	4HGB	0.012 ± 0.002	0.062 ± 0.002	0.006 ± <0.001
glucocochlearin	GCL	nd	2.184 ± 0.166	nd
glucosativin	GSV	6.639 ± 2.402	nd	nd
7-(methylsulfinyl)heptyl GSL	7MSH	nd	4.55 ± 0.393	21.472 ± 1.219
glucobrassicanapin	GBN	nd	0.146 ± 0.005	nd
dimeric 4-mercaptobutyl GSL	DMB	78.861 ± 8.384	nd	nd
glucobarbarin	GBB	nd	nd	0.730 ± 0.037
glucotropaeolin	GTP	0.027 ± 0.002	0.001 ± <0.001	0.001 ± <0.001
glucoerucin	GER	0.733 ± 0.088	nd	nd
glucobrassicin	GBC	0.027 ± 0.008	0.001 ± <0.001	0.118 ± 0.006
4-methoxyglucobrassicin	4MGB	0.366 ± 0.113	nd	2.001 ± 0.104
gluconasturtiin	GNT	0.001 ± <0.001	nd	1.514 ± 0.040
neoglucobrassicin	NGB	2.682 ± 0.433	1.525 ± 0.229	1.596 ± 0.037
4-methylpentyl GSL	4MP	1.293 ± 0.836	nd	nd
hexy GSL	HEX	0.225 ± 0.071	nd	0.434 ± 0.031
7-(methylthio)heptyl GSL	7MTH	nd	0.132 ± 0.035	2.663 ± 0.104
butyl GSL ^$^	BUT	0.301 ± 0.081	nd	0.606 ± 0.359
**Total**		111.098 ± 14.633	27.532 ± 2.831	31.645 ± 1.353

^a^ GSL: glucosinolate; ^$^ = tentative identification. ^b^ Concentration in μmol g^−1^ dry weight; means are from six replicates for salad rocket, five replicates for wasabi and eight replicates for watercress; nd, not detected.

**Table 5:** Identified volatile glucosinolate hydrolysis products in the headspace of ‘salad’ rocket, wasabi, and watercress, and the presence/absence of their respective glucosinolate precursor.

Precursor Glucosinolate	Glucosinolate Hydrolysis Product (Compound No.)	Glucosinolate Observed?			Hydrolysis Product Observed?		
		Salad Rocket	Wasabi	Watercress	Salad Rocket	Wasabi	Watercress
sinigrin	3-butenenitrile (**74**) ^$^	√	√	√	x	√	x
	allyl thiocyanate (**4**) ^$^	√	√	√	x	√	x
	allyl ITC (**5/6**) ^$^	√	√	√	x	√	x
	cyclopropane ITC (**7**) ^$^	√	√	√	x	√	x
	thiiraneacetonitrile (**77**) ^$^	√	√	√	x	x	x
glucocapparin	methyl thiocyanate (**2**) ^$^	x	x	x	√ *	x	√ *
glucoputranjivin ^$^	isopropyl ITC (**3**) ^$^	√	√	x	√	x	x
glucocochlearin ^$^	*sec*-butyl ITC (**9**) ^$^	x	√	x	√ *	√	x
4-methylpentyl GSL ^$^	5-methylhexanenitrile (**75**) ^$^	√	x	x	√	x	x
	1-isothiocyanato-4-methylpentane (**16**) ^$^	√	x	x	√	√ *	√ *
isobutyl GSL	isobutyl ITC (**10**) ^$^	x	√	x	x	√	x
<unknown>	6-heptenenitrile (**76**) ^$^	-	-	-	x	√ *	x
gluconapin	3-butenyl ITC (**11**) ^$^	√	√	x	√	√	x
butyl GSL	butyl ITC (**12**) ^$^	√	x	√	√	√ *	x
3-methylbutyl GSL	isoamyl ITC (**13**) ^$^	x	x	x	√ *	√ *	√ *
glucobrassicanapin	4-pentenyl ITC (**14**) ^$^	x	√	x	x	√	x
pentyl GSL	pentyl ITC (**15**) ^$^	√	x	x	√	√ *	x
glucotropaeolin	phenylacetonitrile (**78**) ^$^	√	√	√	x	x	x
	benzyl ITC (**22**) ^$^	√	√	√	x	√	x
hexyl GSL	cyclohexyl ITC (**17**) ^$^	√	x	√	x	√ *	x
glucoberteroin	5-(methylsulfanyl)pentanenitrile (**79**) ^$^	√	x	x	√	x	x
glucoerucin	4-(methylthio)butanenitrile (**80**) ^$^	√	x	x	x	x	x
	erucin (**23**) ^$^	√	x	x	√	√ *	x
gluconasturtiin	benzenepropanenitrile (**81**) ^$^	√	x	√	x	x	√
glucoiberverin	iberverin (**19**) ^$^	x	x	x	√ *	√ *	x
glucosativin	sativin (**20**) ^$^	√	x	x	√	x	x
<unknown>	octyl ITC (**21**) ^$^	x	x	x	x	x	√ *
gluconasturtiin	phenylethyl ITC (**24**) ^$^	√	x	√	x	√ *	√

* Hydrolysis product observed but not glucosinolate precursor; ^$^ tentatively identified.

## Data Availability

The data presented in this study is available on request from the corresponding author.

## Supplementary Materials

The following are available online at https://www.mdpi.com/article/10.3390/foods10051055/s1, Supplementary Data S1. Compound fragmentation spectra with corresponding library matches (where available) and GC-MS chromatograms.

## References

[1] Bell L., Oloyede O.O., Lignou S., Wagstaff C., Methven L. (2018). Taste and flavor perceptions of glucosinolates, isothiocyanates, and related compounds. Mol. Nutr. Food Res..

[2] Ravindran P.N., Pillai G.S., Divakaran M., Peter K.V. (2012). Other herbs and spices: Mango ginger to wasabi. Handbook of Herbs and Spices.

[3] Bell L., Spadafora N.D., Müller C.T., Wagstaff C., Rogers H.J. (2016). Use of TD-GC–TOF-MS to assess volatile composition during post-harvest storage in seven accessions of rocket salad (*Eruca sativa*). Food Chem..

[4] Giallourou N., Oruna-Concha M.J., Harbourne N. (2016). Effects of domestic processing methods on the phytochemical content of watercress (*Nasturtium officinale*). Food Chem..

[5] Sultana T., Savage G.P., Mcneil D.L., Porter G.P., Clark B. (2003). Comparison of flavour compounds in wasabi and horseradish. J. Food Agric. Environ..

[6] Kroener E.-M., Buettner A. (2017). Unravelling important odorants in horseradish (*Armoracia rusticana*). Food Chem..

[7] Depree J.A., Howard T.M., Savage G.P. (1999). Flavour and pharmaceutical properties of the volatile sulphur compounds of Wasabi (*Wasabia japonica*). Food Res. Int..

[8] Bell L., Wagstaff C. (2014). Glucosinolates, myrosinase hydrolysis products, and flavonols found in rocket (*Eruca sativa* and *Diplotaxis tenuifolia*). J. Agric. Food Chem..

[9] Palaniswamy U.R. (2001). Watercress: A salad crop with chemopreventive potential. Horttechnology.

[10] Siegmund B., Parker J.K., Elmore J.S., Methven L. (2015). Biogenesis of aroma compounds: Flavour formation in fruits and vegetables. Flavour Development, Analysis and Perception in Food and Beverages.

[11] Kim J.I., Dolan W.L., Anderson N.A., Chapple C. (2015). Indole glucosinolate biosynthesis limits phenylpropanoid accumulation in *Arabidopsis thaliana*. Plant Cell.

[12] Raffo A., Masci M., Moneta E., Nicoli S., Sánchez del Pulgar J., Paoletti F., Pulgar D. (2018). Characterization of volatiles and identification of odor-active compounds of rocket leaves. Food Chem..

[13] Blazevic I., Mastelic J. (2008). Free and bound volatiles of rocket (*Eruca sativa* Mill.). Flavour Fragrance J..

[14] Jirovetz L., Smith D., Buchbauer G. (2002). Aroma compound analysis of *Eruca sativa* (Brassicaceae) SPME headspace leaf samples using GC, GC−MS, and olfactometry. J. Agric. Food Chem..

[15] Miyazawa M., Maehara T., Kurose K. (2002). Composition of the essential oil from the leaves of Eruca sativa. Flavour Fragrance J..

[16] Nakanishi A., Miyazawa N., Haraguchi K., Watanabe H., Kurobayashi Y., Nammoku T. (2014). Determination of the absolute configuration of a novel odour-active lactone, cis-3-methyl-4-decanolide, in wasabi (*Wasabia japonica* Matsum.). Flavour Fragrance J..

[17] Kameoka H., Hashimoto S. (1982). Volatile flavor components from wild *Wasabia japonica* Matsum. (Wasabi) and *Nasturtium officinale* R. Br. (Orandagarashi). J. Agric. Chem. Soc. Japan.

[18] Liu Y., Zhang H., Umashankar S., Liang X., Lee H., Swarup S., Ong C. (2018). Characterization of plant volatiles reveals distinct metabolic profiles and pathways among 12 Brassicaceae vegetables. Metabolites.

[19] Silva M.F., Campos V.P., Barros A.F., Terra W.C., Pedroso M.P., Gomes V.A., Ribeiro C.R., Silva F.J. (2020). Volatile emissions of watercress (*Nasturtium officinale*) leaves and passion fruit (*Passiflora edulis*) seeds against Meloidogyne incognita. Pest Manag. Sci..

[20] Spence R.-M.M., Tucknott O.G. (1983). An apparatus for the comparative collection of headspace volatiles of watercress. J. Sci. Food Agric..

[21] Agneta R., Möllers C., Rivelli A.R. (2013). Horseradish (*Armoracia rusticana*), a neglected medical and condiment species with a relevant glucosinolate profile: A review. Genet. Resour. Crop Evol..

[22] Dekić M.S., Radulović N.S., Stojanović N.M., Randjelović P.J., Stojanović-Radić Z.Z., Najman S., Stojanović S. (2017). Spasmolytic, antimicrobial and cytotoxic activities of 5-phenylpentyl isothiocyanate, a new glucosinolate autolysis product from horseradish (*Armoracia rusticana* P. Gaertn., B. Mey. & Scherb., Brassicaceae). Food Chem..

[23] Gilbert J., Nursten H.E. (1972). Volatile constituents of horseradish roots. J. Sci. Food Agric..

[24] Pilipczuk T., Kusznierewicz B., Chmiel T., Przychodzeń W., Bartoszek A. (2017). Simultaneous determination of individual isothiocyanates in plant samples by HPLC-DAD-MS following SPE and derivatization with N -acetyl- l -cysteine. Food Chem..

[25] Kroener E.-M., Buettner A. (2018). Sensory-analytical comparison of the aroma of different horseradish varieties (*Armoracia rusticana*). Front. Chem..

[26] Pasini F., Verardo V., Caboni M.F., D’Antuono L.F. (2012). Determination of glucosinolates and phenolic compounds in rocket salad by HPLC-DAD–MS: Evaluation of *Eruca sativa* Mill. and *Diplotaxis tenuifolia* L. genetic resources. Food Chem..

[27] Bell L., Wagstaff C. (2019). Rocket science: A review of phytochemical & health-related research in Eruca & Diplotaxis species. Food Chem. X.

[28] Agerbirk N., Matthes A., Erthmann P.Ø., Ugolini L., Cinti S., Lazaridi E., Nuzillard J.-M., Müller C., Bak S., Rollin P. (2018). Glucosinolate turnover in Brassicales species to an oxazolidin-2-one, formed via the 2-thione and without formation of thioamide. Phytochemistry.

[29] Bell L., Oruna-Concha M.J., Wagstaff C. (2015). Identification and quantification of glucosinolate and flavonol compounds in rocket salad (*Eruca sativa*, *Eruca vesicaria* and *Diplotaxis tenuifolia*) by LC–MS: Highlighting the potential for improving nutritional value of rocket crops. Food Chem..

[30] Rochfort S.J., Trenerry V.C., Imsic M., Panozzo J., Jones R. (2008). Class targeted metabolomics: ESI ion trap screening methods for glucosinolates based on MSn fragmentation. Phytochemistry.

[31] Andini S., Dekker P., Gruppen H., Araya-Cloutier C., Vincken J.-P. (2019). Modulation of glucosinolate composition in Brassicaceae seeds by germination and fungal elicitation. J. Agric. Food Chem..

[32] Cataldi T.R.I., Rubino A., Lelario F., Bufo S.A. (2007). Naturally occurring glucosinolates in plant extracts of rocket salad (*Eruca sativa* L.) identified by liquid chromatography coupled with negative ion electrospray ionization and quadrupole ion-trap mass spectrometry. Rapid Commun. Mass Spectrom..

[33] Agneta R., Rivelli A.R., Ventrella E., Lelario F., Sarli G., Bufo S.A. (2012). Investigation of glucosinolate profile and qualitative aspects in sprouts and roots of horseradish (*Armoracia rusticana*) using LC-ESI–hybrid linear ion trap with fourier transform ion cyclotron resonance mass spectrometry and infrared multiphoton dissociation. J. Agric. Food Chem..

[34] Maldini M., Foddai M., Natella F., Petretto G.L., Rourke J.P., Chessa M., Pintore G. (2017). Identification and quantification of glucosinolates in different tissues of *Raphanus raphanistrum* by liquid chromatography tandem-mass spectrometry. J. Food Compos. Anal..

[35] Shi H., Zhao Y., Sun J., Yu L., Chen P. (2017). Chemical profiling of glucosinolates in cruciferous vegetables-based dietary supplements using ultra-high performance liquid chromatography coupled to tandem high resolution mass spectrometry. J. Food Compos. Anal..

[36] Mellon F.A., Bennett R.N., Holst B., Williamson G. (2002). Intact glucosinolate analysis in plant extracts by programmed cone voltage electrospray LC/MS: Performance and comparison with LC/MS/MS methods. Anal. Biochem..

[37] Bianco G., Agerbirk N., Losito I., Cataldi T.R.I. (2014). Acylated glucosinolates with diverse acyl groups investigated by high resolution mass spectrometry and infrared multiphoton dissociation. Phytochemistry.

[38] Blažević I., Montaut S., Burčul F., Olsen C., Burow M., Rollin P., Agerbirk N. (2019). Glucosinolate structural diversity, identification, chemical synthesis and metabolism in plants. Phytochemistry.

[39] Al-Gendy A.A., Lockwood G.B. (2003). GC-MS analysis of volatile hydrolysis products from glucosinolates in Farsetia aegyptia var. ovalis. Flavour Fragrance J..

[40] Wang C., Xing J., Chin C.-K., Ho C.-T., Martin C.E. (2001). Modification of fatty acids changes the flavor volatiles in tomato leaves. Phytochemistry.

[41] Buttery R.G., Teranishi R., Ling L.C., Turnbaugh J.G. (1990). Quantitative and sensory studies on tomato paste volatiles. J. Agric. Food Chem..

[42] Deasy W., Shepherd T., Alexander C.J., Birch A.N.E., Evans K.A. (2016). Development and validation of a SPME-GC-MS method for in situ passive sampling of root volatiles from glasshouse-grown broccoli plants undergoing blow-ground herbivory by larvae of cabbage root fly, *Delia radicum* L.. Phytochem. Anal..

[43] Moshonas M.G., Shaw P.E. (1973). Some newly found orange essence components including trans-2-pentenal. J. Food Sci..

[44] Berger R.G., Drawert F., Kollmannsberger H. (1989). The flavour of cape gooseberry (*Physalis peruviana* L.). Z. Für Lebensm. -Unters. und Forsch..

[45] Hatanaka A., Harada T. (1973). Formation of cis-3-hexenal, trans-2-hexenal and cis-3-hexenol in macerated *Thea sinensis* leaves. Phytochemistry.

[46] D’Auria J.C., Pichersky E., Schaub A., Hansel A., Gershenzon J. (2007). Characterization of a BAHD acyltransferase responsible for producing the green leaf volatile (Z)-3-hexen-1-yl acetate in Arabidopsis thaliana. Plant J..

[47] Angerosa F. (2000). Virgin olive oil odour notes: Their relationships with volatile compounds from the lipoxygenase pathway and secoiridoid compounds. Food Chem..

[48] Kato M., Imayoshi Y., Iwabuchi H., Shimomura K. (2011). Kinetic changes in glucosinolate-derived volatiles by heat-treatment and myrosinase activity in nakajimana (*Brassica rapa* L. cv. *nakajimana*). J. Agric. Food Chem..

[49] Sharma A., Rai P.K., Prasad S. (2018). GC–MS detection and determination of major volatile compounds in *Brassica juncea* L. leaves and seeds. Microchem. J..

[50] Song H., Liu J. (2018). GC-O-MS technique and its applications in food flavor analysis. Food Res. Int..

[51] Aparicio R., Morales M.T., Alonso V. (1997). Authentication of European virgin olive oils by their chemical compounds, sensory attributes, and consumers’ attitudes. J. Agric. Food Chem..

[52] Hossain S., Bergkvist G., Berglund K., Glinwood R., Kabouw P., Mårtensson A., Persson P. (2014). Concentration- and time-dependent effects of isothiocyanates produced from Brassicaceae shoot tissues on the pea root rot pathogen *Aphanomyces euteiches*. J. Agric. Food Chem..

[53] Kask K., Kännaste A., Talts E., Copolovici L., Niinemets Ü. (2016). How specialized volatiles respond to chronic and short-term physiological and shock heat stress in *Brassica nigra*. Plant Cell Environ..

[54] Bruce T.J.A. (2014). Glucosinolates in oilseed rape: Secondary metabolites that influence interactions with herbivores and their natural enemies. Ann. Appl. Biol..

[55] Hansen M., Laustsen A.M., Olsen C.E., Poll L., SØRensen H. (1997). Chemical and sensory quality of Broccoli (*Brassica oleracea* L. Var *italica*). J. Food Qual..

[56] Hanschen F.S., Kaufmann M., Kupke F., Hackl T., Kroh L.W., Rohn S., Schreiner M. (2018). Brassica vegetables as sources of epithionitriles: Novel secondary products formed during cooking. Food Chem..

[57] Fernandes F., Pereira D.M., Guedes de Pinho P., Valentão P., Pereira J.A., Bento A., Andrade P.B. (2010). Headspace solid-phase microextraction and gas chromatography/ion trap-mass spectrometry applied to a living system: Pieris brassicae fed with kale. Food Chem..

[58] Bartlet E., Blight M.M., Lane P., Williams I.H. (1997). The responses of the cabbage seed weevil *Ceutorhynchus assimilis* to volatile compounds from oilseed rape in a linear track olfactometer. Entomol. Exp. Appl..

[59] Fernandes F., Guedes de Pinho P., Valentão P., Pereira J.A., Andrade P.B. (2009). Volatile constituents throughout *Brassica oleracea* L. Var. *acephala* germination. J. Agric. Food Chem..

[60] Ortner E., Granvogl M., Schieberle P. (2016). Elucidation of thermally induced changes in key odorants of white mustard seeds (*Sinapis alba* L.) and rapeseeds (*Brassica napus* L.) using molecular sensory science. J. Agric. Food Chem..

[61] Smid H.M., Van Loon J.J.A., Posthumus M.A., Vet L.E.M. (2002). GC-EAG-analysis of volatiles from brussels sprouts plants damaged by two species of Pieris caterpillars: Olfactory receptive range of a specialist and a generalist parasitoid wasp species. Chemoecology.

[62] Klopsch R., Witzel K., Börner A., Schreiner M., Hanschen F.S. (2017). Metabolic profiling of glucosinolates and their hydrolysis products in a germplasm collection of *Brassica rapa* turnips. Food Res. Int..

[63] Angelino D., Jeffery E. (2014). Glucosinolate hydrolysis and bioavailability of resulting isothiocyanates: Focus on glucoraphanin. J. Funct. Foods.

[64] Petrović S., Drobac M., Ušjak L., Filipović V., Milenković M., Niketić M. (2017). Volatiles of roots of wild-growing and cultivated *Armoracia macrocarpa* and their antimicrobial activity, in comparison to horseradish, *A. rusticana*. Ind. Crops Prod..

[65] Tohar N., Mohd M.A., Jantan I., Awang K. (2006). A comparative study of the essential oils of the genus Plumeria Linn. from Malaysia. Flavour Fragr. J..

[66] Tieman D., Taylor M., Schauer N., Fernie A.R., Hanson A.D., Klee H.J. (2006). Tomato aromatic amino acid decarboxylases participate in synthesis of the flavor volatiles 2-phenylethanol and 2-phenylacetaldehyde. Proc. Natl. Acad. Sci. USA.

[67] Triqui R., Reineccius G. (1995). Changes in flavor profiles with ripening of anchovy (*Engraulis encrasicholus*). J. Agric. Food Chem..

[68] Parker J.K., Parker J.K., Elmore J.S., Methven L. (2015). Introduction to aroma compounds in foods. Flavour Development, Analysis and Perception in Food and Beverages.

[69] Schreiner M., Krumbein A., Ruppel S. (2009). Interaction between plants and bacteria: Glucosinolates and phyllospheric colonization of cruciferous vegetables by Enterobacter radicincitans DSM 16656. J. Mol. Microbiol. Biotechnol..

[70] Deng Q., Zinoviadou K.G., Galanakis C.M., Orlien V., Grimi N., Vorobiev E., Lebovka N., Barba F.J. (2015). The effects of conventional and non-conventional processing on glucosinolates and its derived forms, isothiocyanates: Extraction, degradation, and applications. Food Eng. Rev..

[71] Park H.-W., Choi K.-D., Shin I.-S. (2013). Antimicrobial activity of isothiocyanates (ITCs) extracted from horseradish (*Armoracia rusticana*) root against oral microorganisms. Biocontrol Sci..

[72] Terada Y., Masuda H., Watanabe T. (2015). Structure–activity relationship study on isothiocyanates: Comparison of TRPA1-activating ability between allyl isothiocyanate and specific flavor components of wasabi, horseradish, and white mustard. J. Nat. Prod..

[73] Masuda H., Harada Y., Tanaka K., Nakajima M., Tabeta H. (1996). Characteristic odorants of wasabi (*Wasabia japonica matum*), Japanese horseradish, in comparison with those of horseradish (*Armoracia rusticana*). Biotechnology for Improved Foods and Flavors.

[74] Lv J., Wu J., Zuo J., Fan L., Shi J., Gao L., Li M., Wang Q. (2017). Effect of Se treatment on the volatile compounds in broccoli. Food Chem..

[75] Blight M.M., Pickett J.A., Wadhams L.J., Woodcock C.M. (1995). Antennal perception of oilseed rape, *Brassica napus* (Brassicaceae), volatiles by the cabbage seed weevil *Ceutorhynchus assimilis* (Coleoptera, Curculionidae). J. Chem. Ecol..

[76] Chen H.-Z., Zhang M., Guo Z. (2019). Discrimination of fresh-cut broccoli freshness by volatiles using electronic nose and gas chromatography-mass spectrometry. Postharvest Biol. Technol..

[77] Müller-Maatsch J., Gurtner K., Carle R., Björn Steingass C. (2019). Investigation into the removal of glucosinolates and volatiles from anthocyanin-rich extracts of red cabbage. Food Chem..

[78] Davies K. (2004). Annual Plant Reviews, Plant Pigments and Their Manipulation.

[79] Mastrandrea L., Amodio M.L., Pati S., Colelli G. (2017). Effect of modified atmosphere packaging and temperature abuse on flavor related volatile compounds of rocket leaves (*Diplotaxis tenuifolia* L.). J. Food Sci. Technol..

[80] Silva Souza M.A., Peres L.E., Freschi J.R., Purgatto E., Lajolo F.M., Hassimotto N.M. (2020). Changes in flavonoid and carotenoid profiles alter volatile organic compounds in purple and orange cherry tomatoes obtained by allele introgression. J. Sci. Food Agric..

[81] Li L., Lee W., Lee W.J., Auh J.H., Kim S.S., Yoon J. (2010). Extraction of allyl isothiocyanate from wasabi (*Wasabia japonica Matsum*) using supercritical carbon dioxide. Food Sci. Biotechnol..

[82] Fechner J., Kaufmann M., Herz C., Eisenschmidt D., Lamy E., Kroh L.W., Hanschen F.S. (2018). The major glucosinolate hydrolysis product in rocket (*Eruca sativa* L.), sativin, is 1,3-thiazepane-2-thione: Elucidation of structure, bioactivity, and stability compared to other rocket isothiocyanates. Food Chem..

