# Supplementary material for: Important Odorants of Four Brassicaceae Species, and Discrepancies between Glucosinolate Profiles and Observed Hydrolysis Products
Source: Foods. 2021 May 11;10(5):1055. doi: 10.3390/foods10051055 (PMC8150828; doi:10.3390/foods10051055)
Supplement: Supplementary file 1 [file foods-10-01055-s001.zip › foods-1196604-supplementary.pdf]

Supplementary data S1. Compound fragmentation spectra with corresponding library matches (where available) and GC-MS chromatograms.

For number coding and references please see Table 2.

1. Carbon disulfide

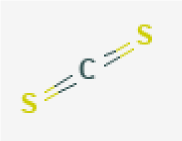

Library Searched : C:\Database\NIST11.L
Quality : 90
ID : Carbon disulfide

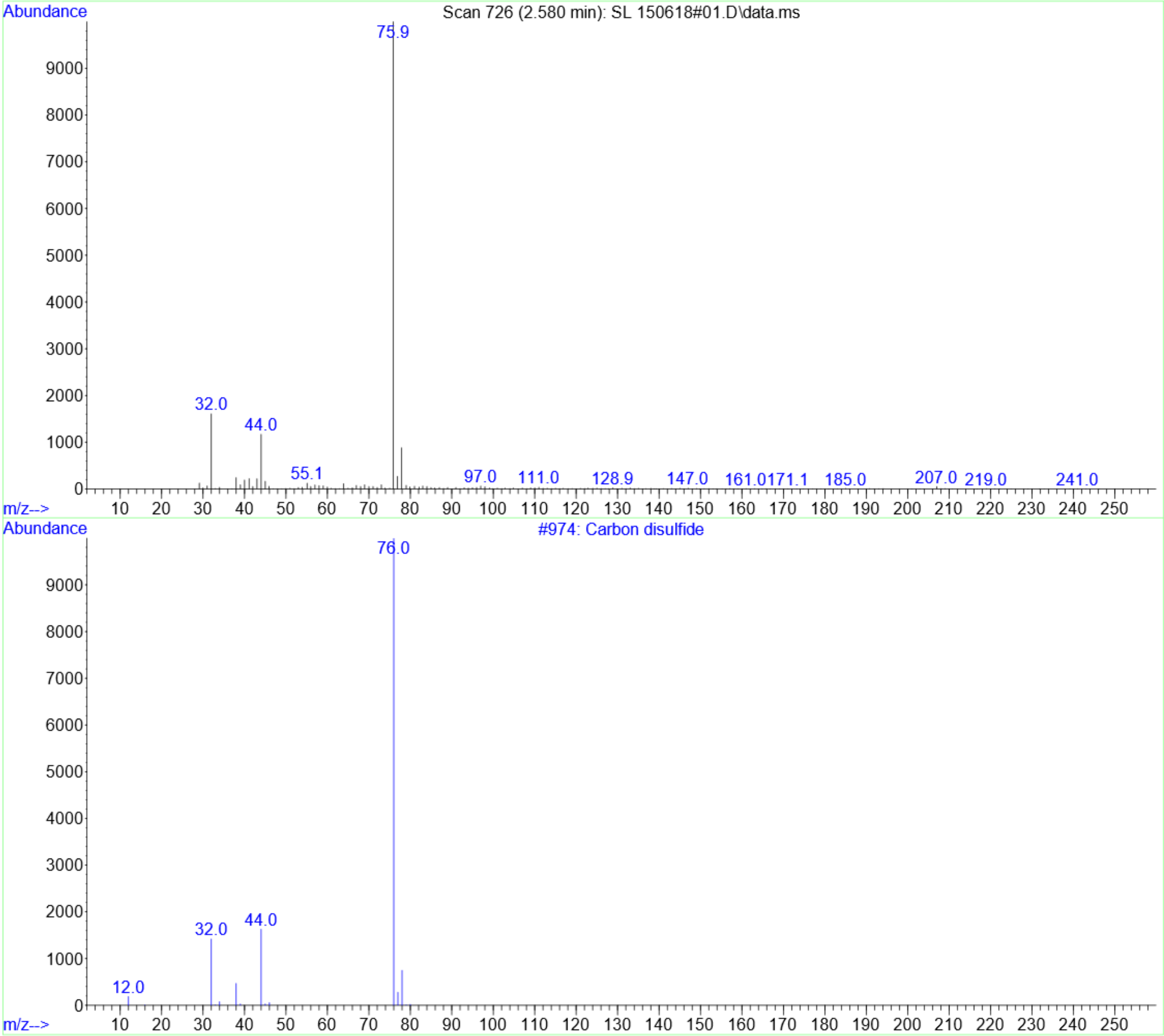

2. Methyl thiocyanate

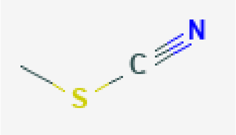

Library Searched : C:\Database\NIST11.L
Quality : 91
ID : Thiocyanic acid, methyl ester

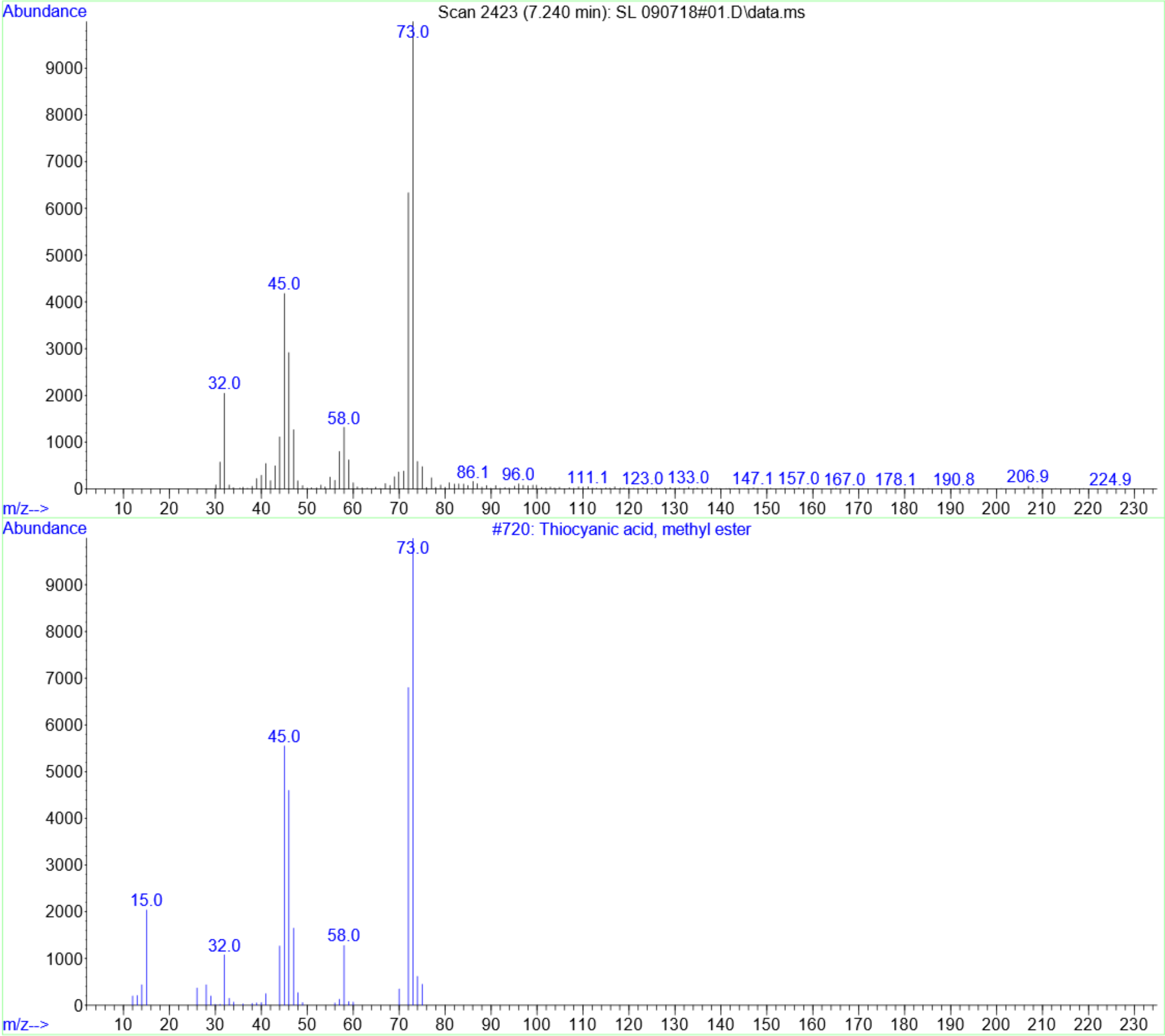

3. Isopropyl isothiocyanate

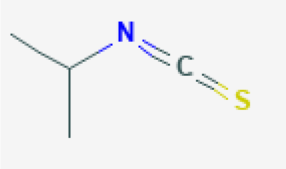

Library Searched : C:\Database\NIST11.L
Quality : 91
ID : Isopropyl isothiocyanate

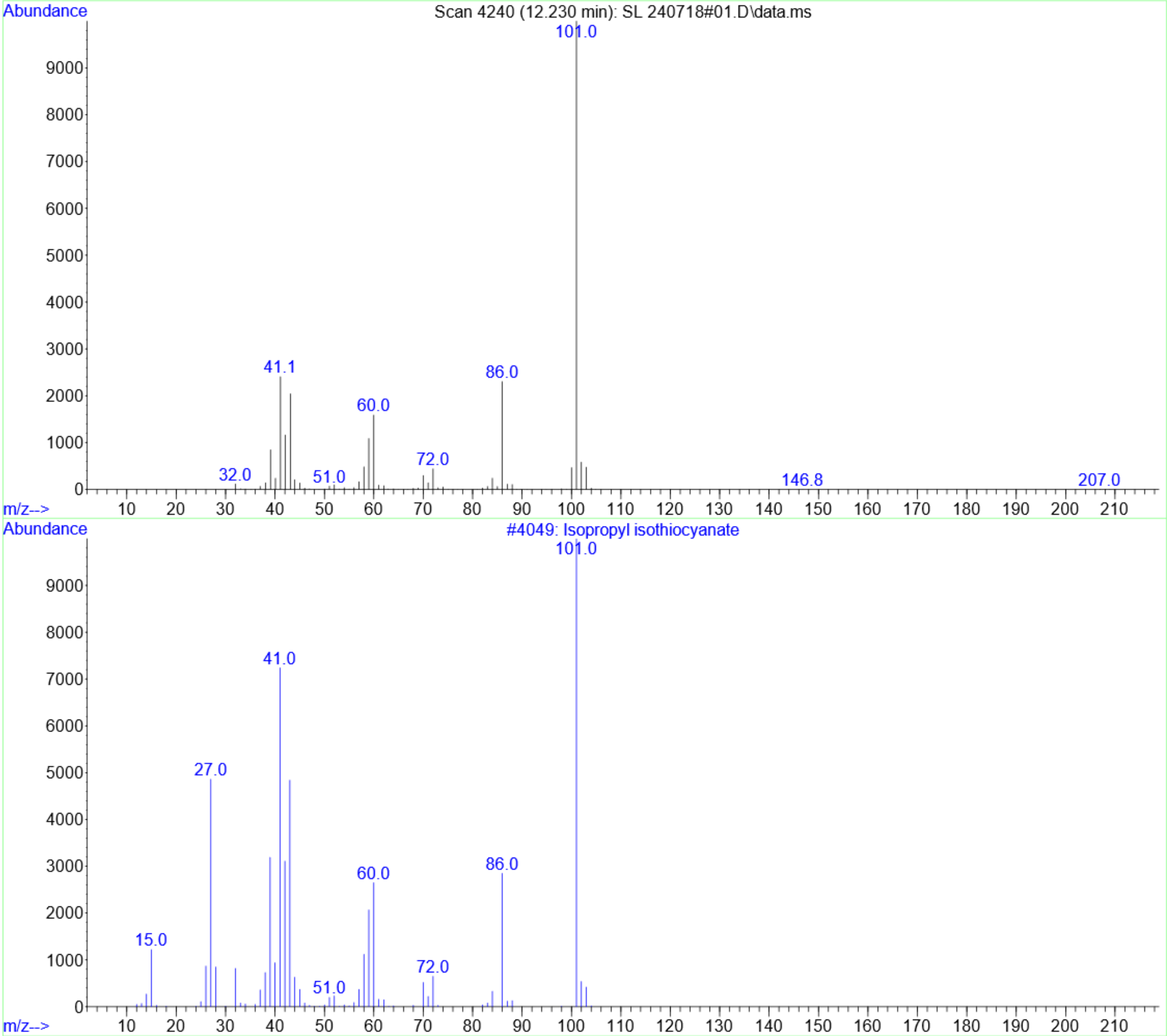

4. Allyl thiocyanate

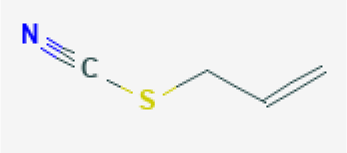

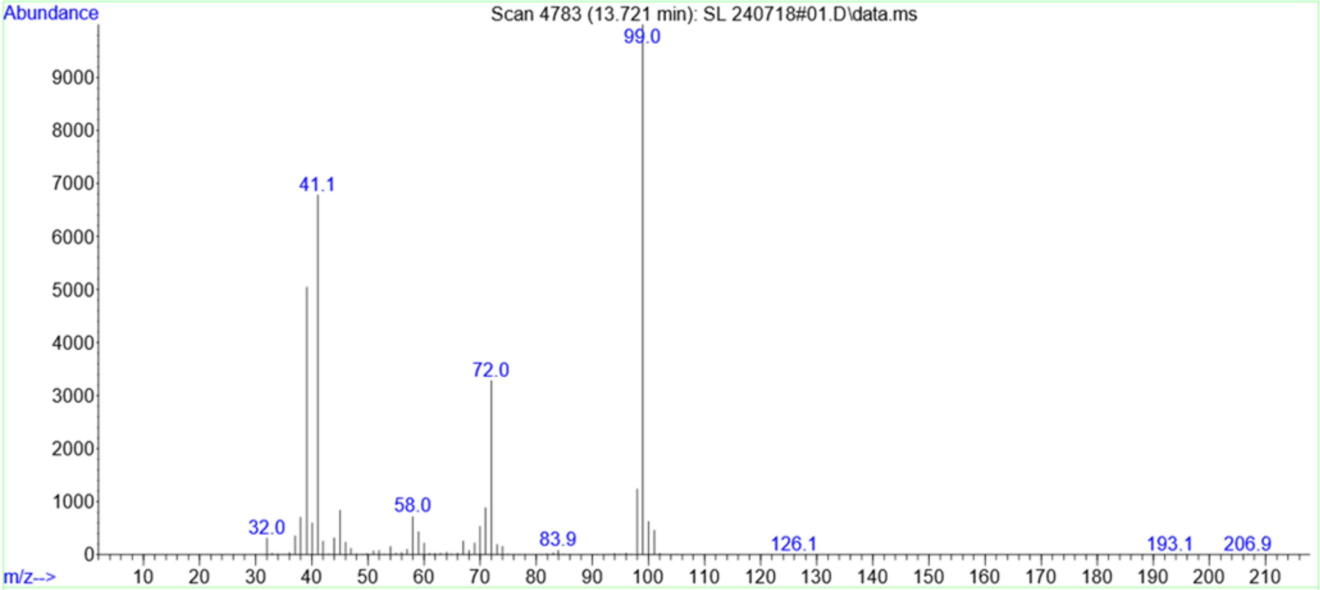

5. Allyl isothiocyanate (peak 1)

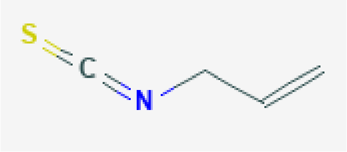

Library Searched : C:\Database\NIST11.L
Quality : 91
ID : Allyl Isothiocyanate

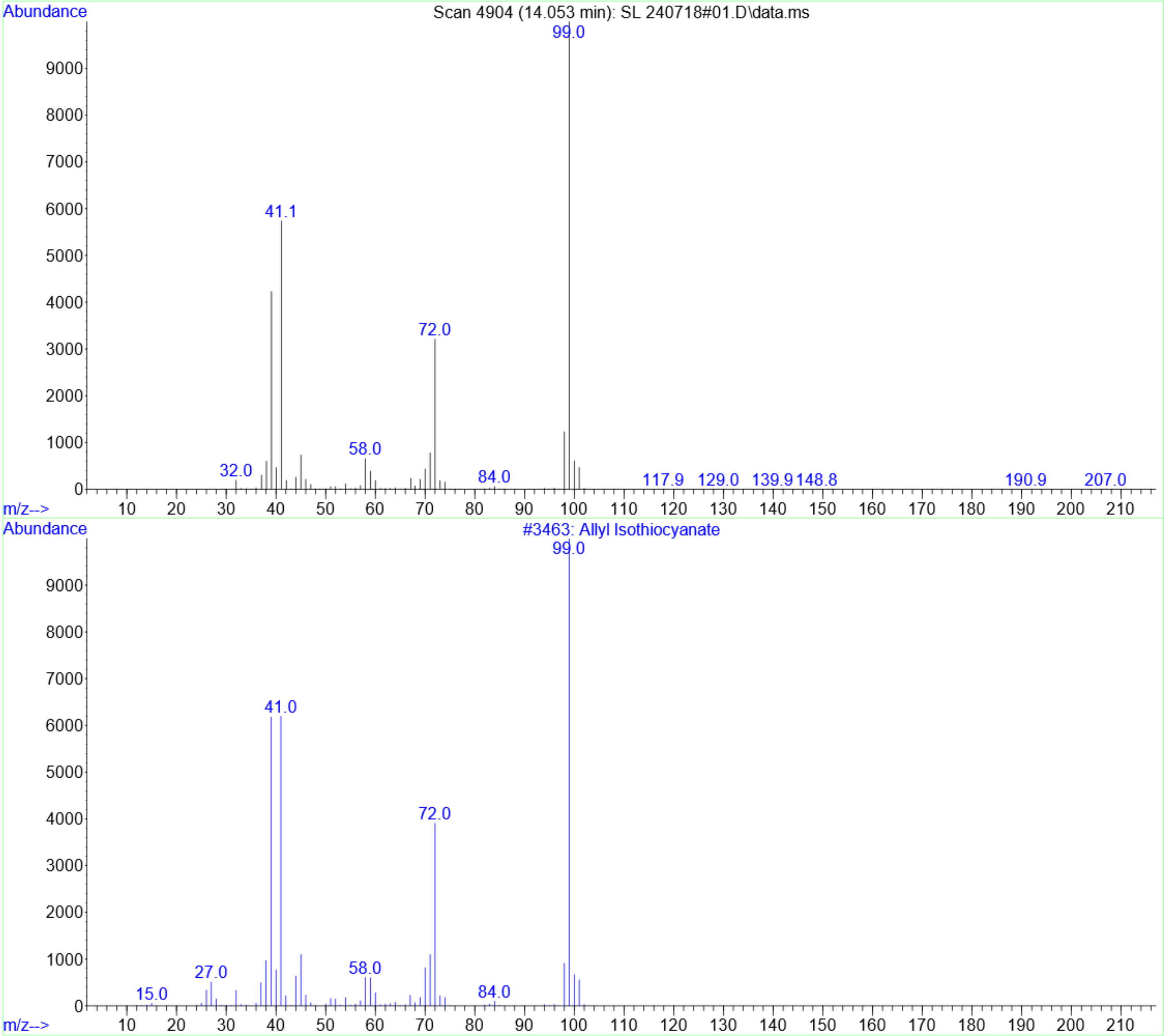

6. Allyl isothiocyanate (peak 2)

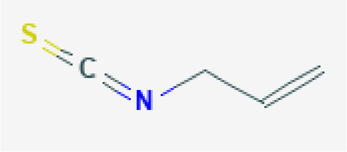

Library Searched : C:\Database\NIST11.L
Quality : 94
ID : Allyl Isothiocyanate

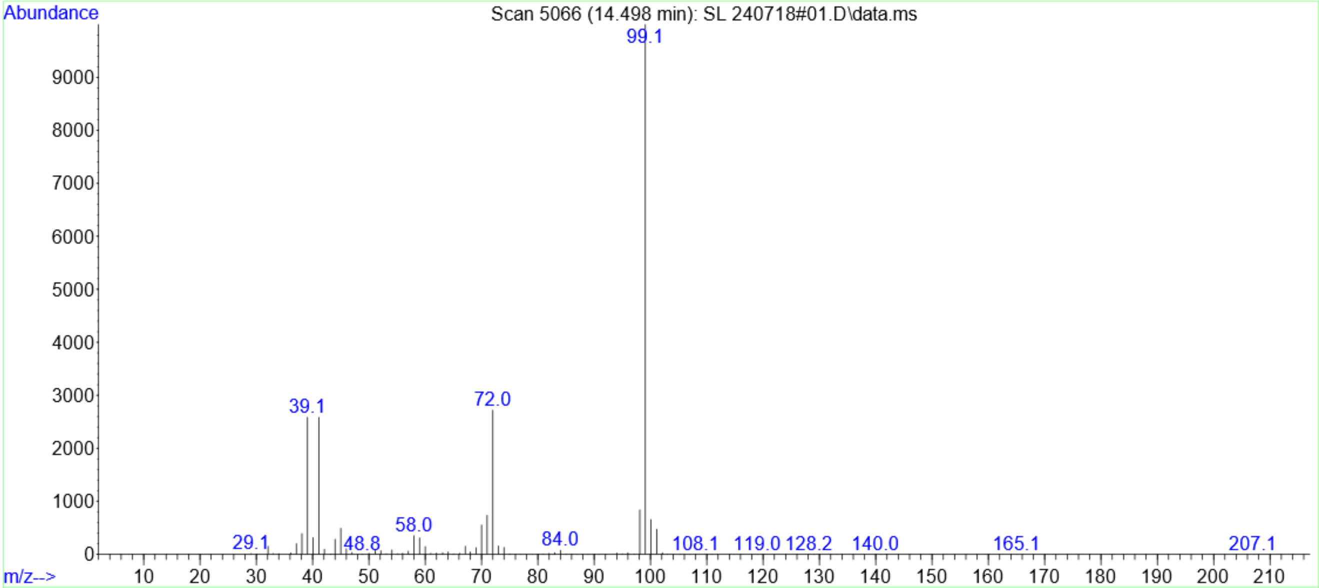

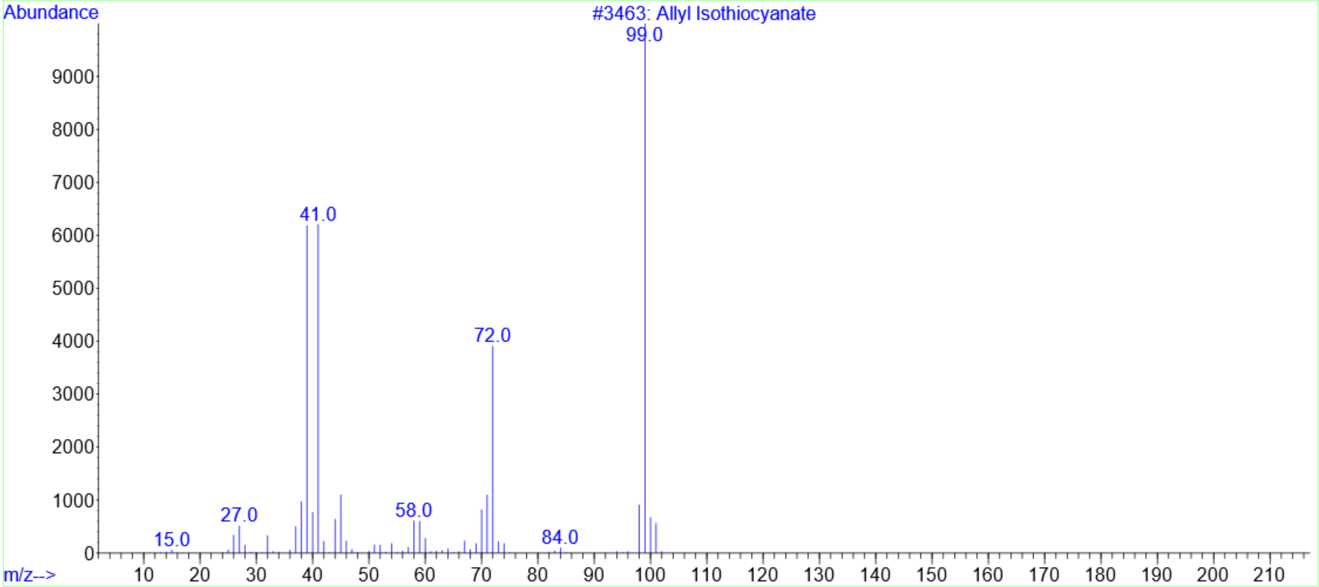

7. Cyclopropane isothiocyanate

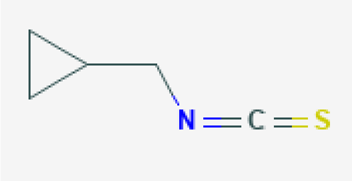

Library Searched : C:\Database\NIST11.L
Quality : 80
ID : Cyclopropane, isothiocyanato-

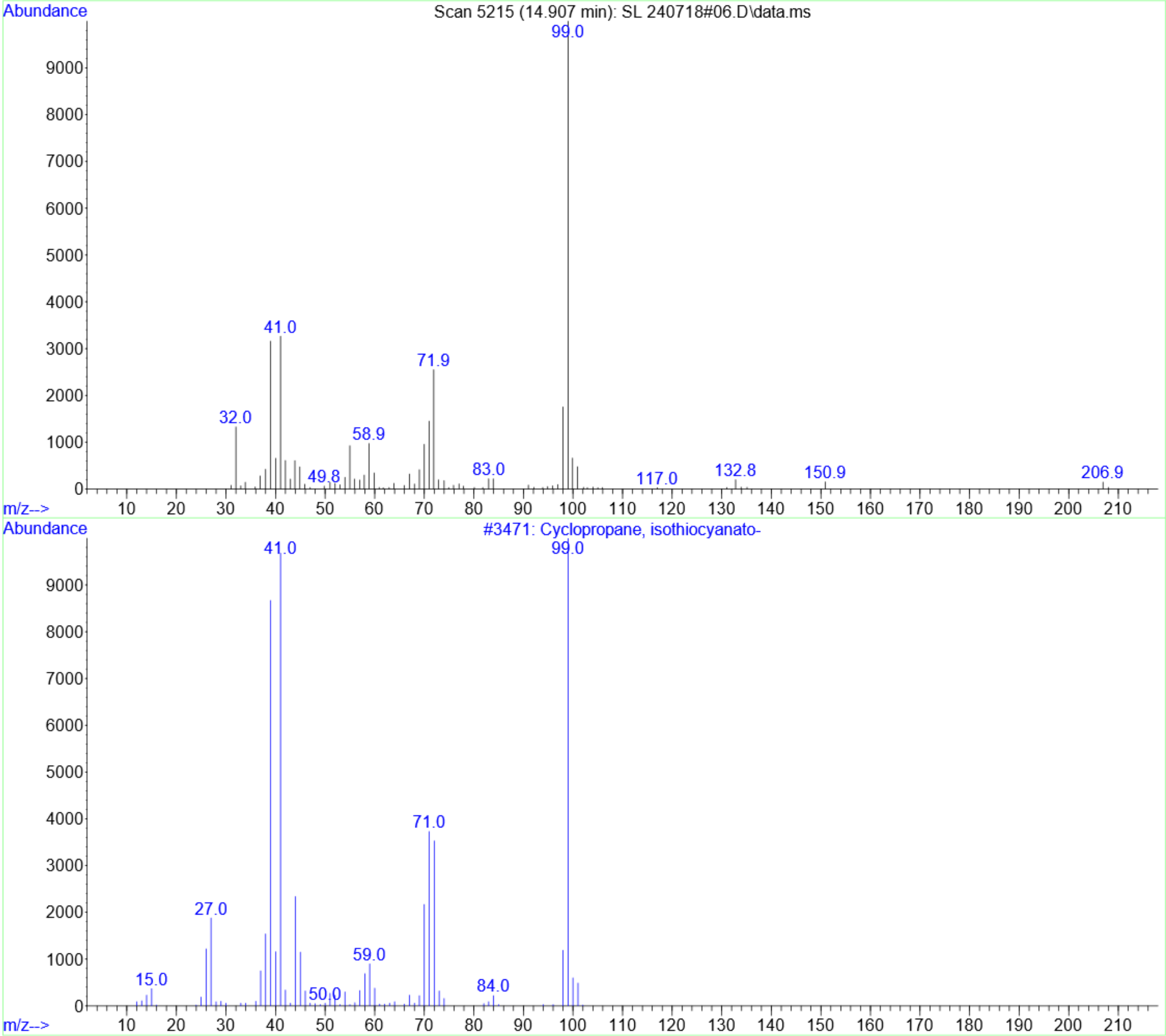

8. Cyclopentyl-1-thiaethane

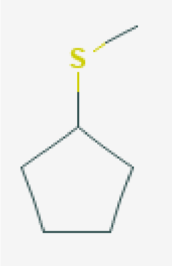

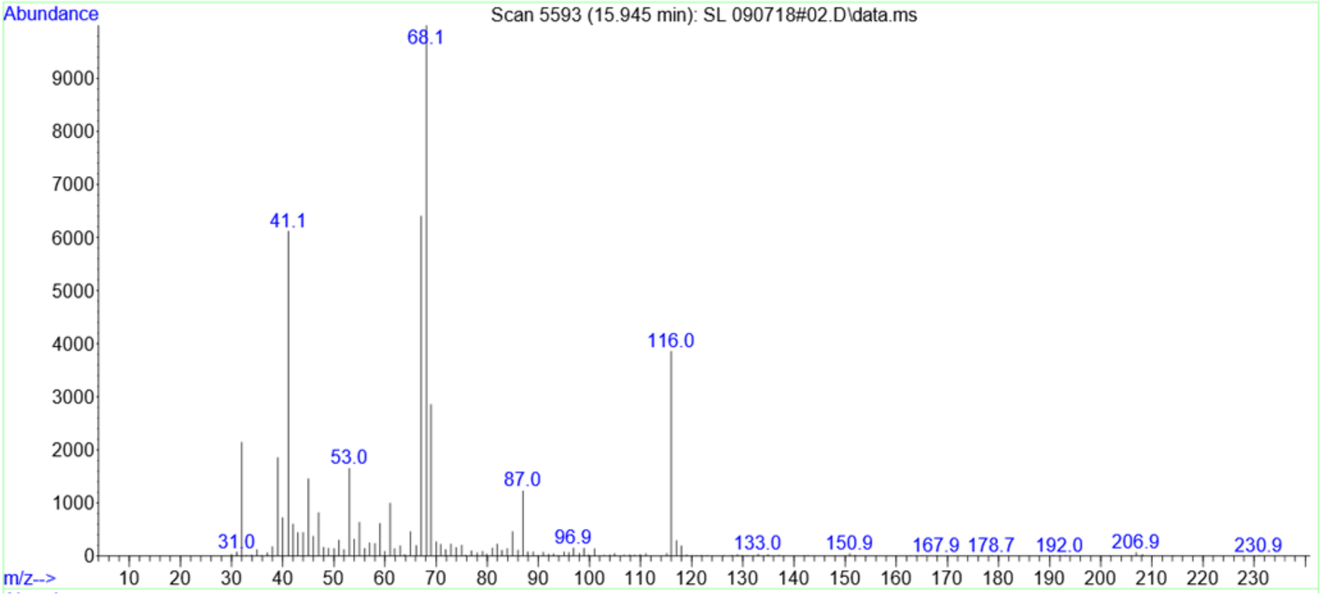

9. *Sec*-butyl isothiocyanate

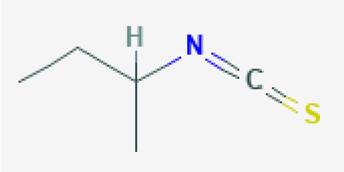

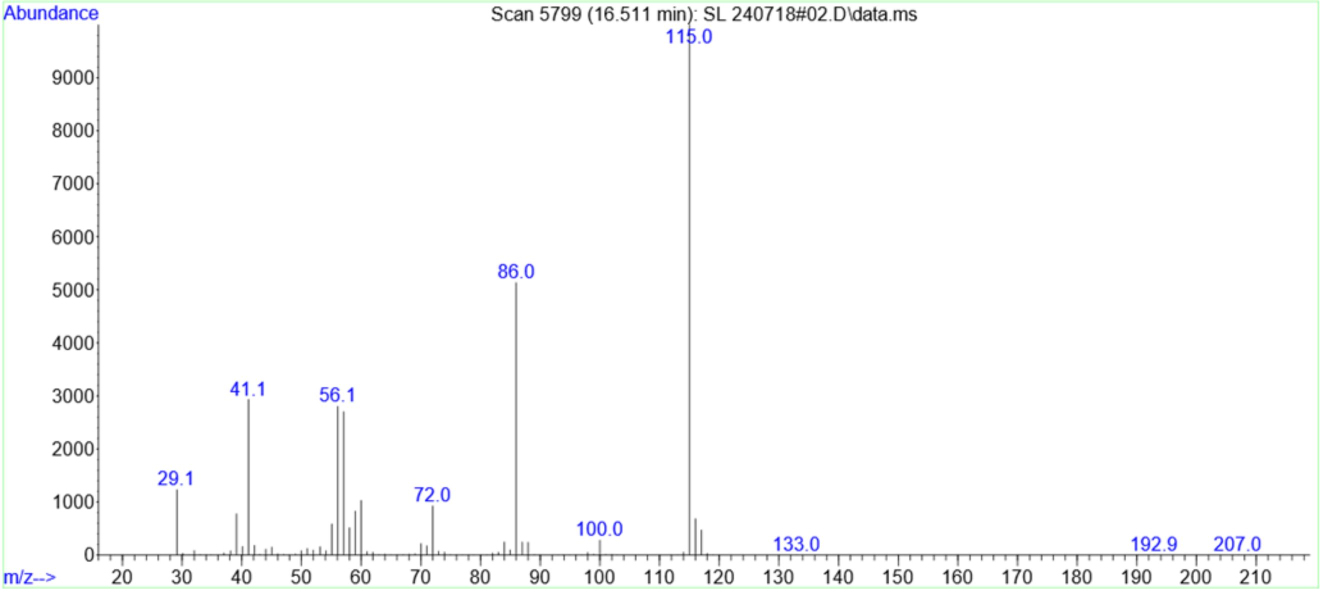

10. Isobutyl isothiocyanate

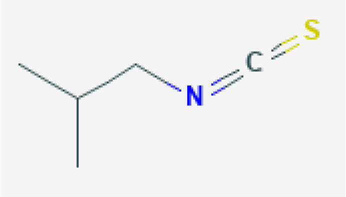

Library Searched : C:\Database\NIST11.L
Quality : 91
ID : Isobutyl isothiocyanate

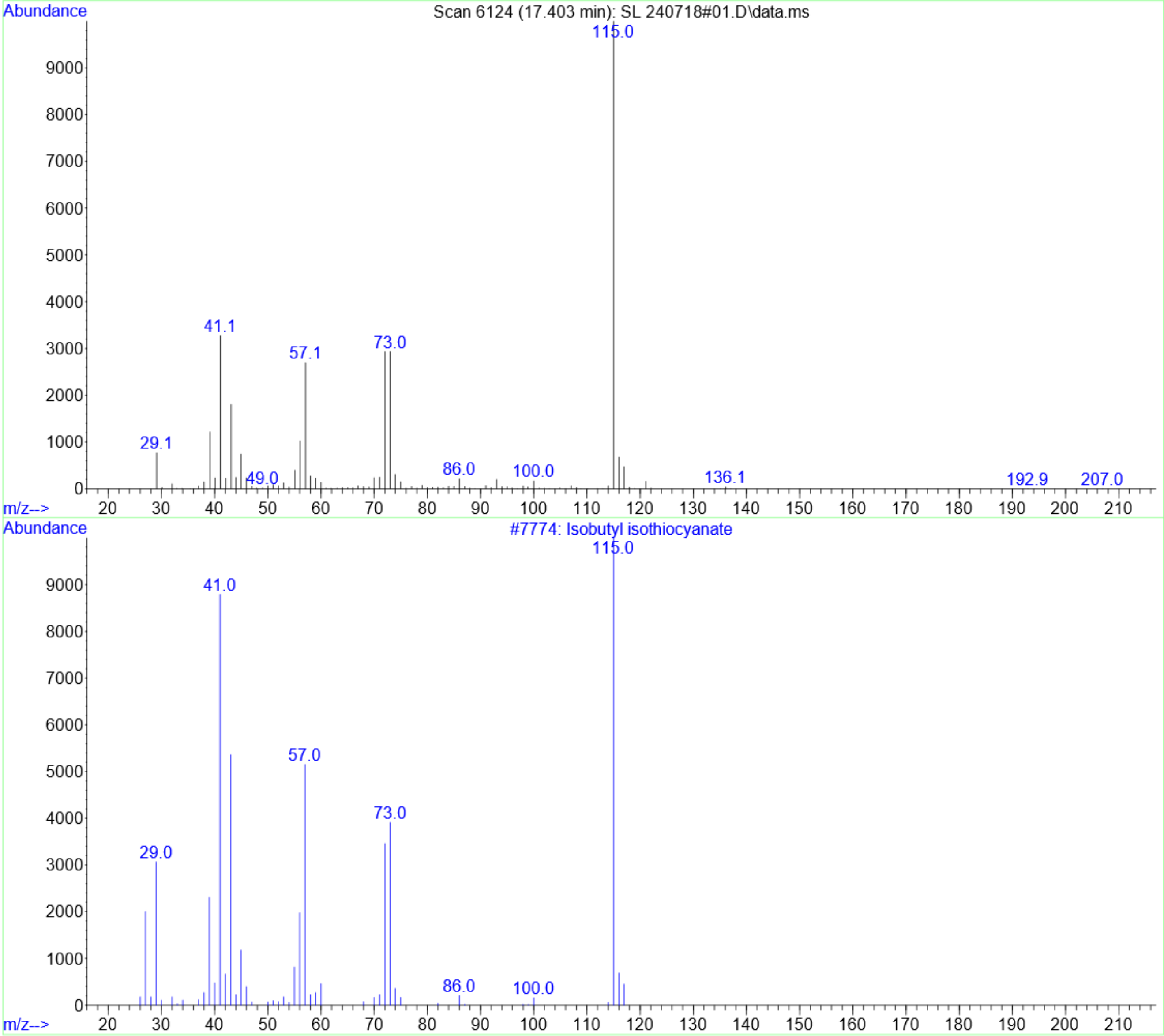

11. 3-butenyl isothiocyanate

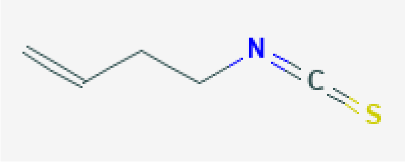

Library Searched : C:\Database\NIST11.L
Quality : 87
ID : 1-Butene, 4-isothiocyanato-

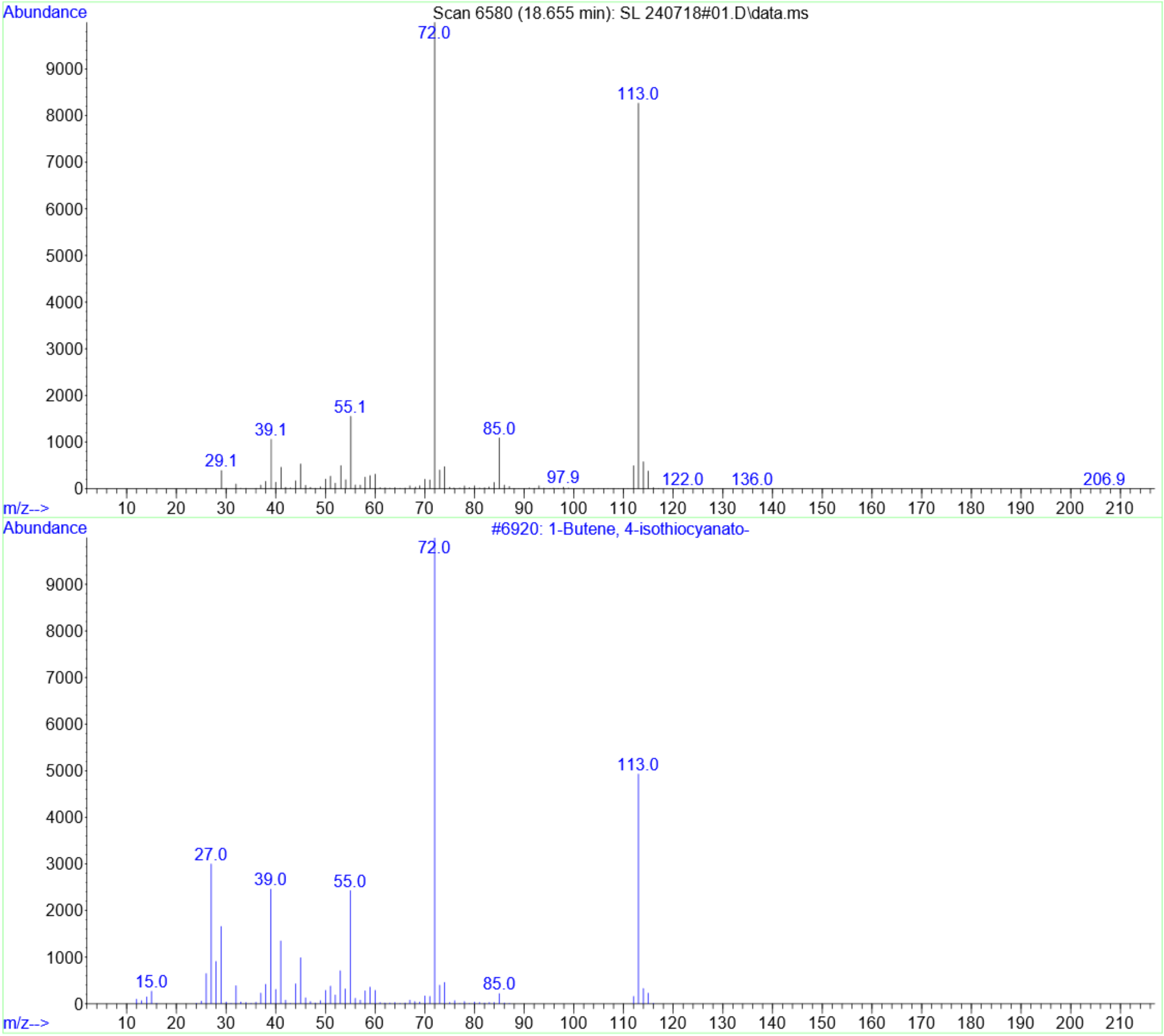

12. Butyl isothiocyanate

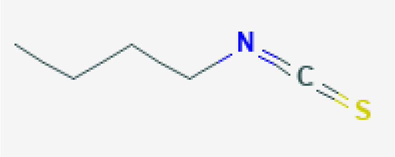

Library Searched : C:\Database\NIST11.L
Quality : 94
ID : Butane, 1-isothiocyanato-

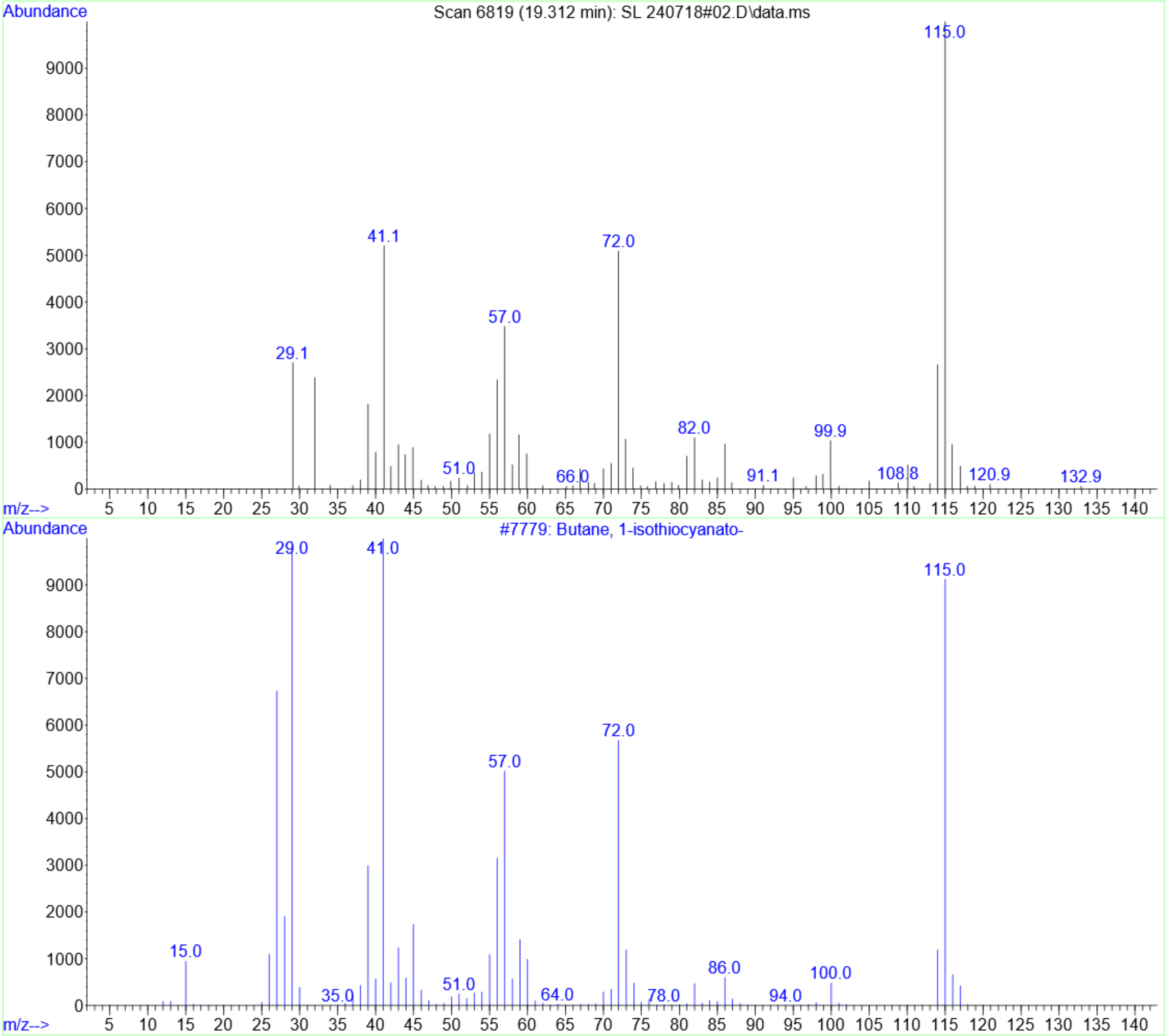

13. Isoamyl isothiocyanate

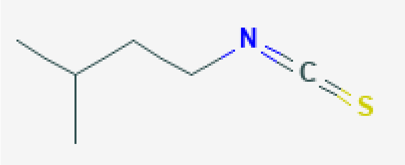

Library Searched : C:\Database\NIST11.L
Quality : 87
ID : Butane, 1-isothiocyanato-3-methyl-

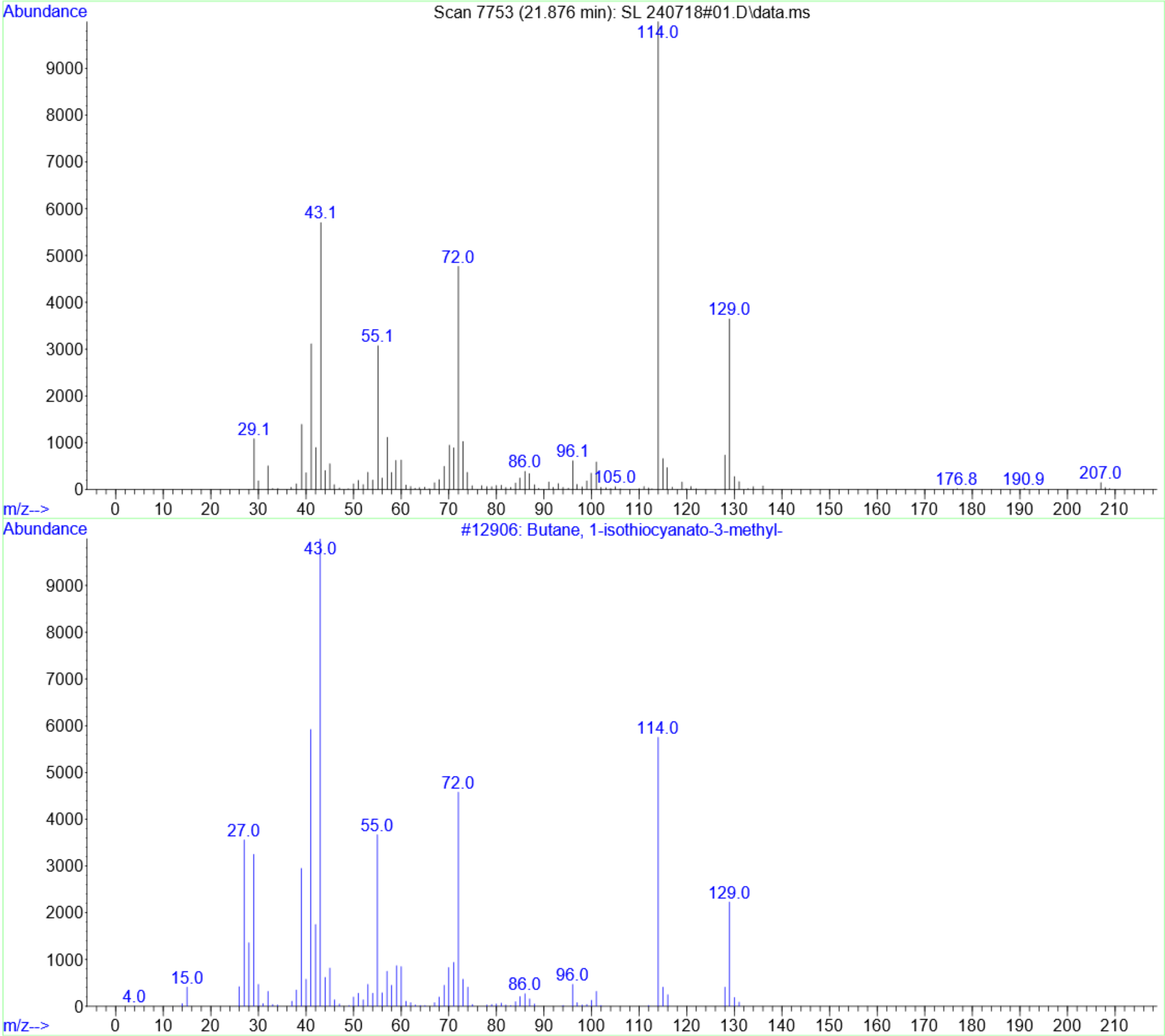

14. 4-pentenyl isothiocyanate

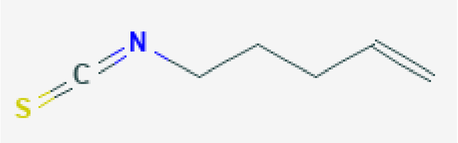

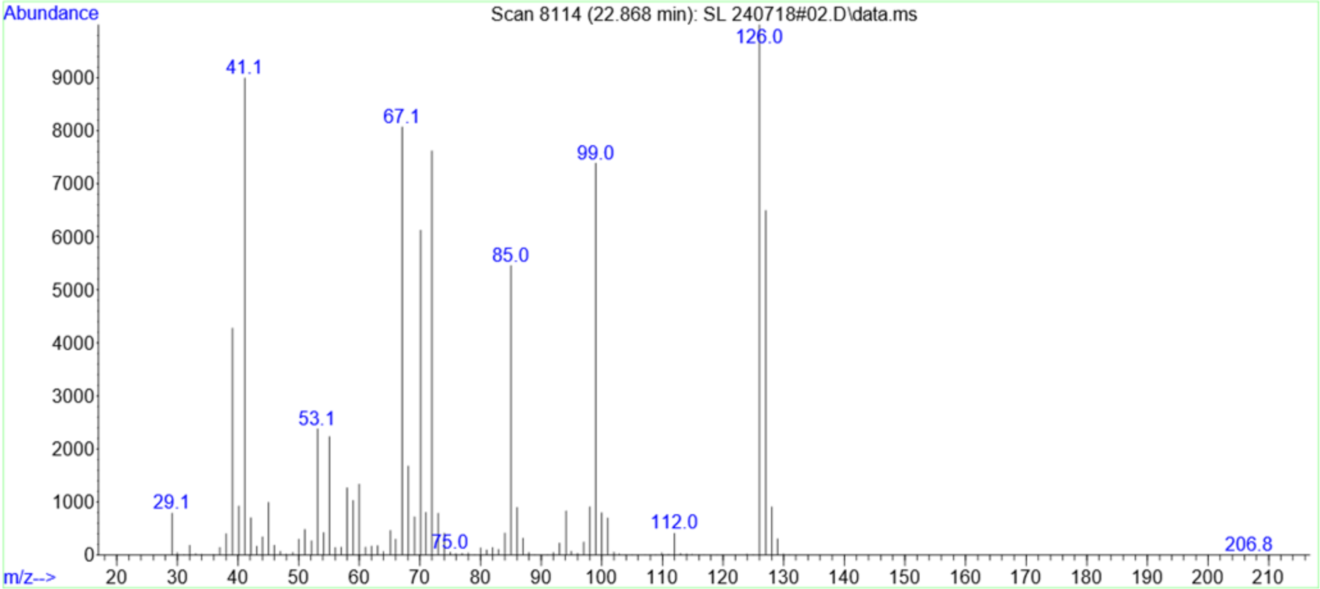

15. Pentyl isothiocyanate

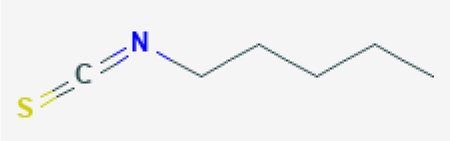

Library Searched : C:\Database\NIST11.L
Quality : 94
ID : n-Pentyl isothiocyanate

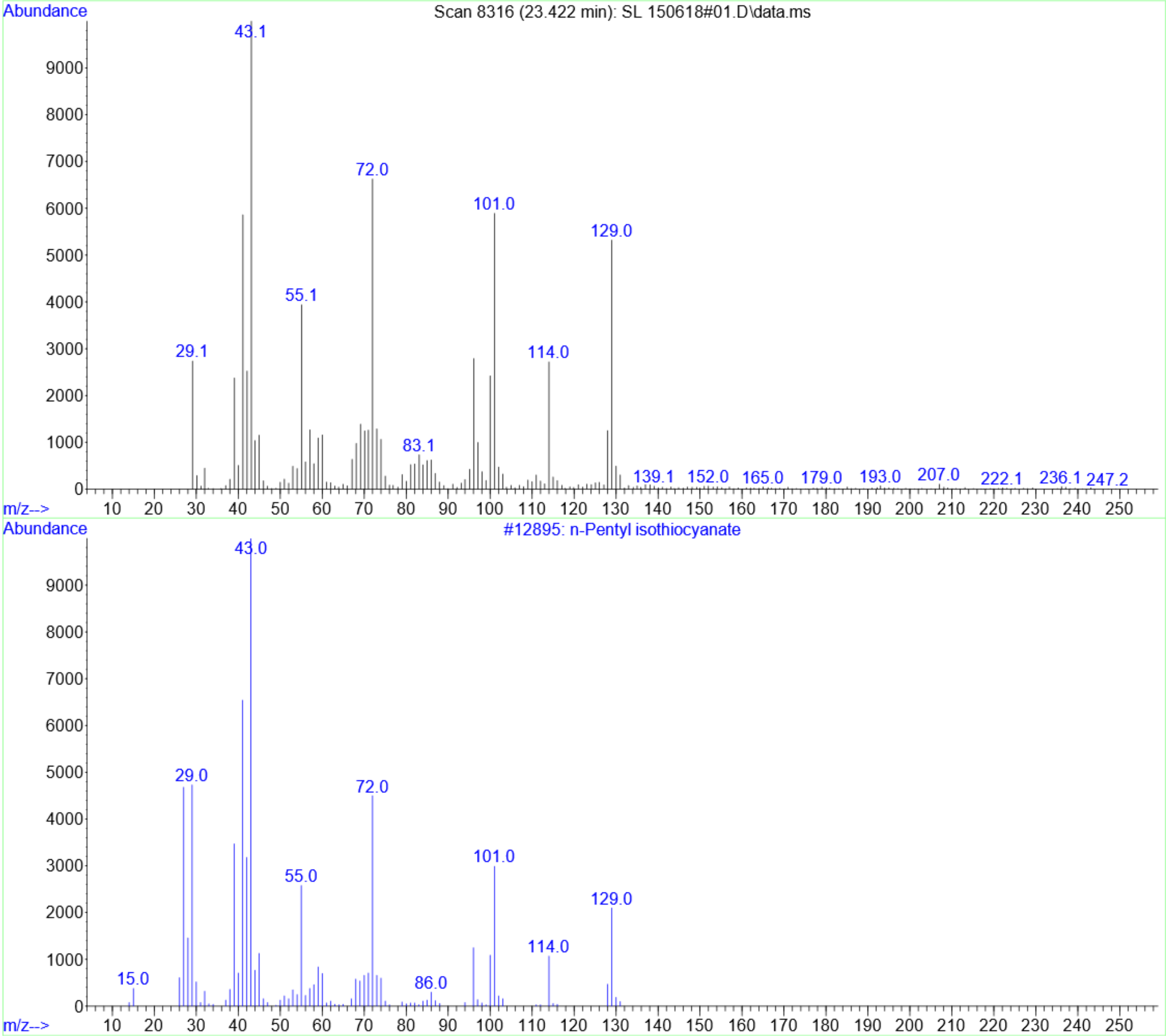

16. 1-isothiocyanato-4-methylpentane

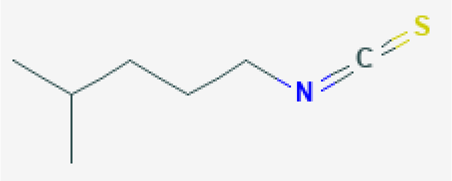

Library Searched : C:\Database\NIST11.L
Quality : 91
ID : 4-Methylpentyl isothiocyanate

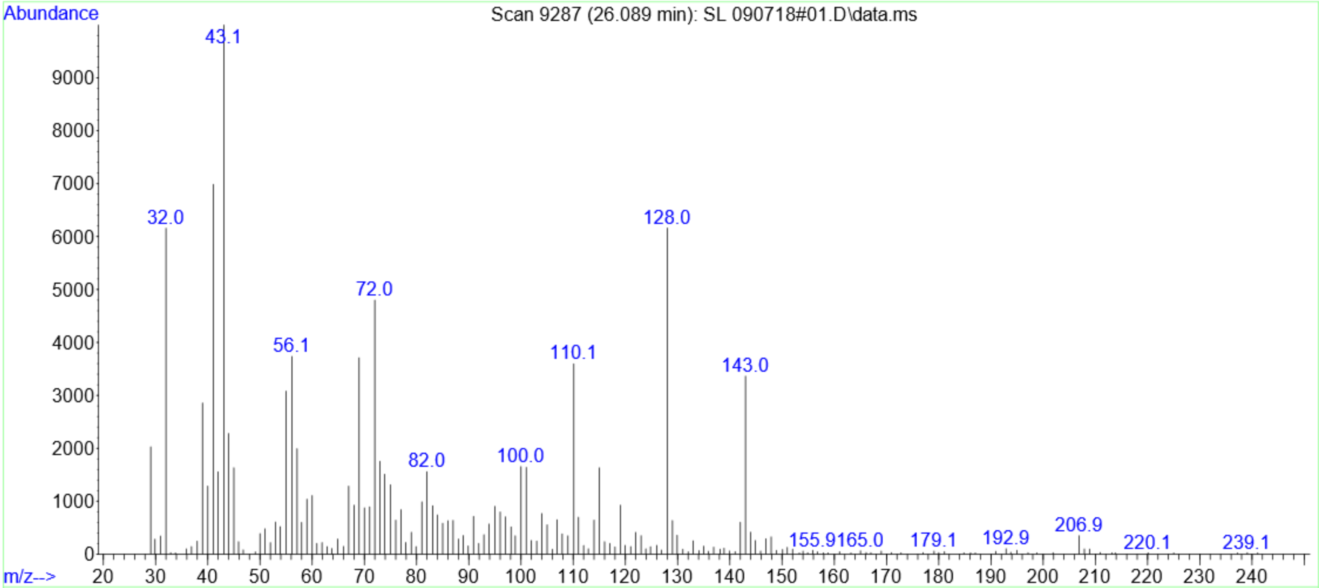

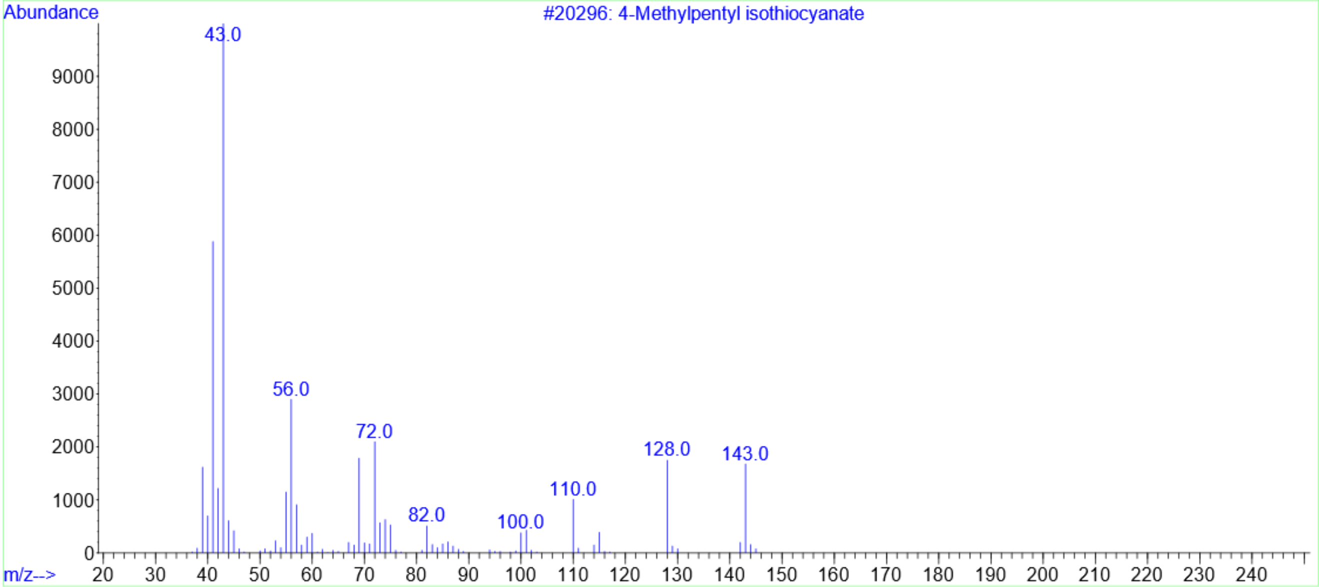

17. Cyclohexyl isothiocyanate

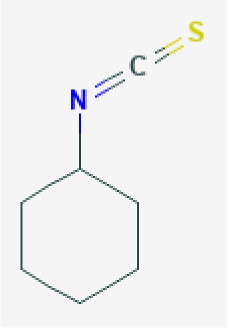

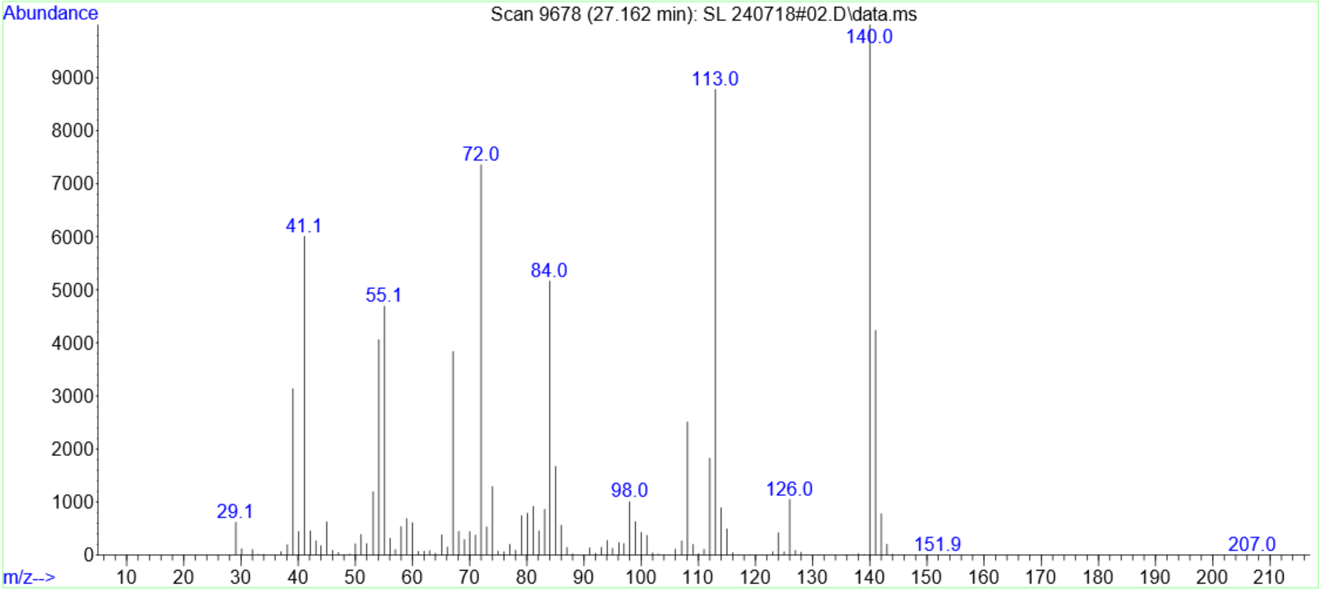

19. Iberverin

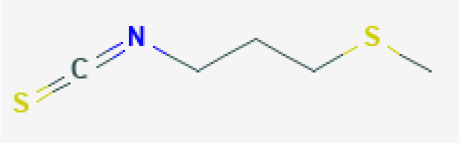

Library Searched : C:\Database\NIST11.L
Quality : 83
ID : Propane, 1-isothiocyanato-3-(methylthio)-

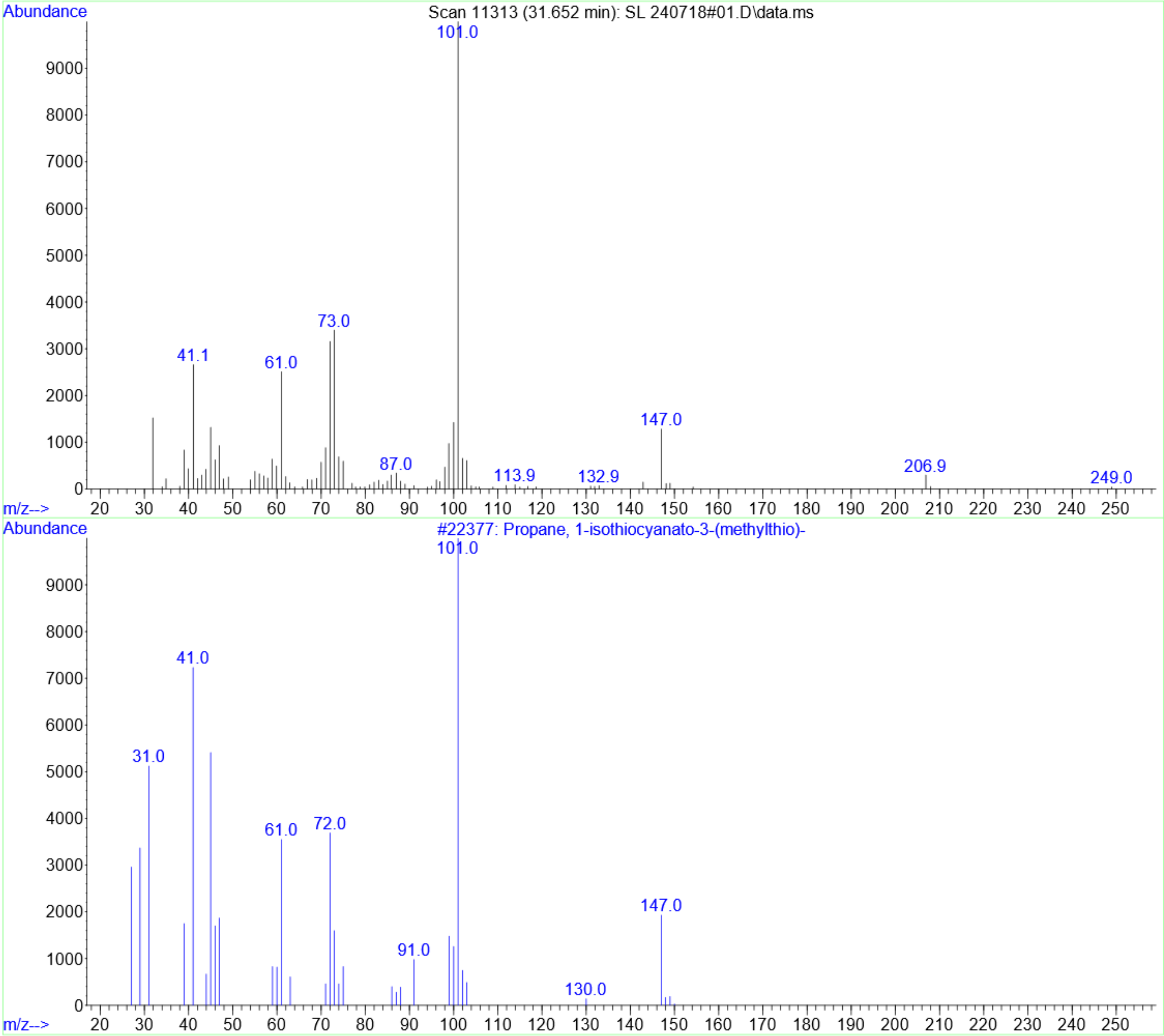

20. Sativin

N.B. It is unknown if this is the ITC or tautomer rearrangement of this compound.

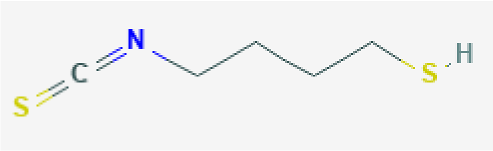

or

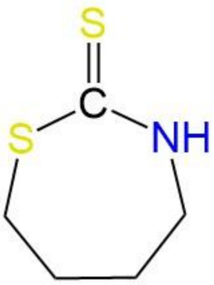

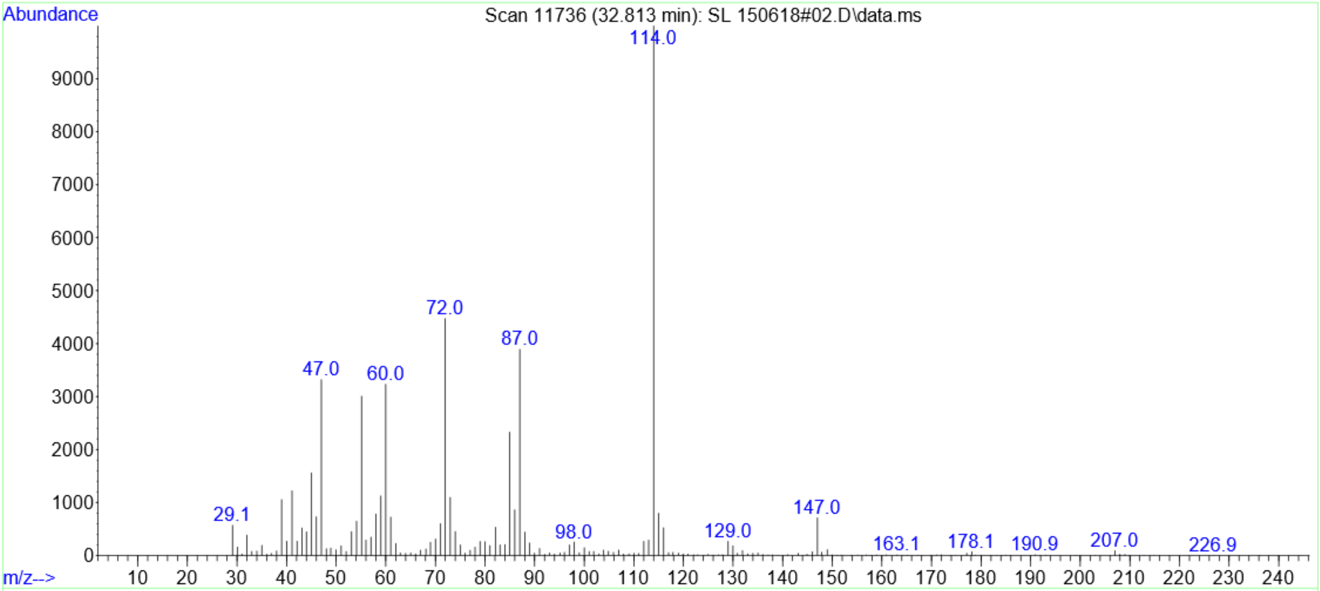

21. Octyl isothiocyanate

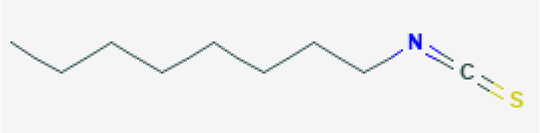

Library Searched : C:\Database\NIST11.L
Quality : 53
ID : Octane, 1-isothiocyanato-

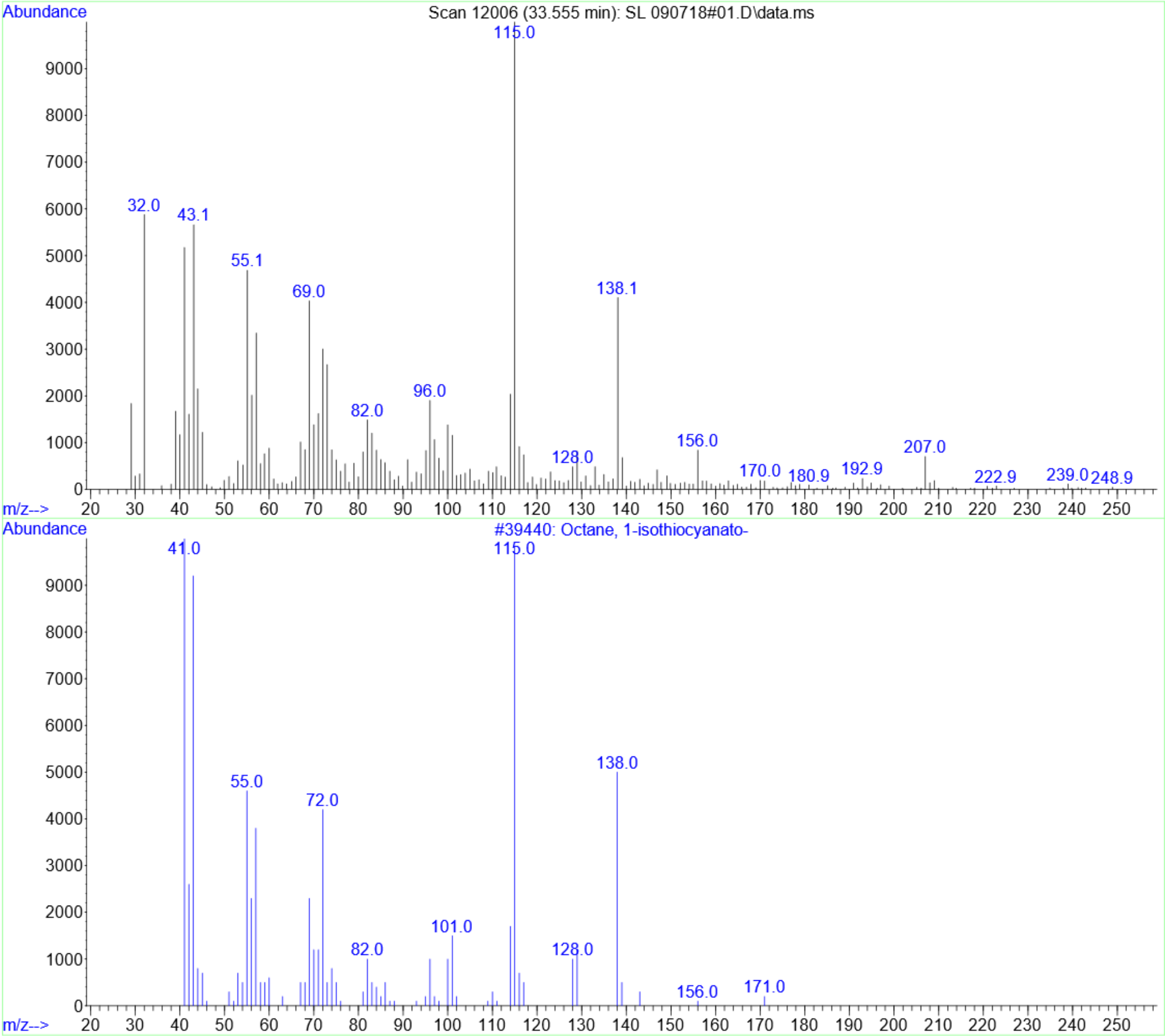

22. Benzyl isothiocyanate

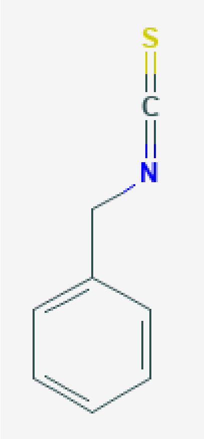

Library Searched : C:\Database\NIST11.L
Quality : 91
ID : Benzene, (isothiocyanatomethyl)-

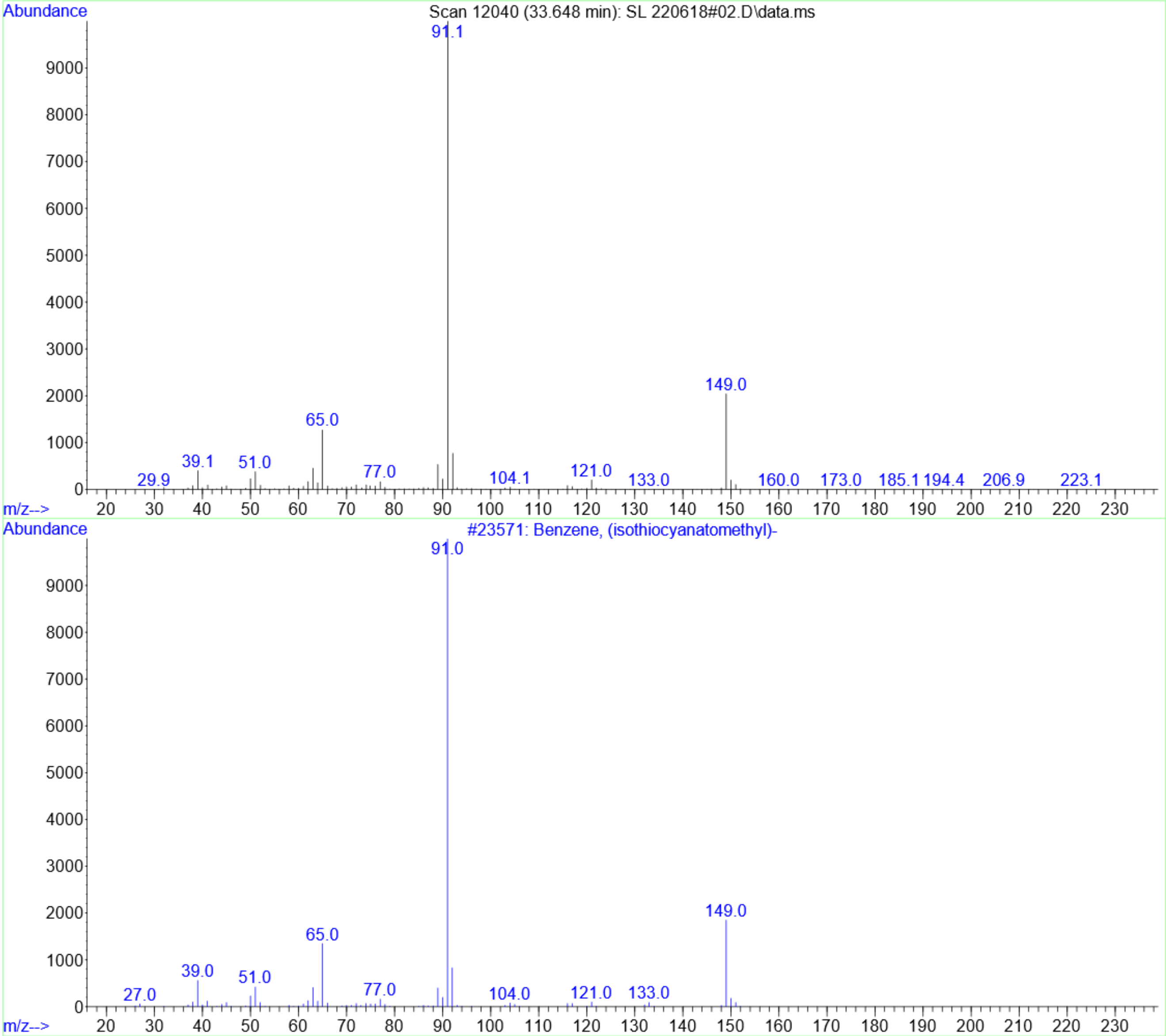

23. Erucin

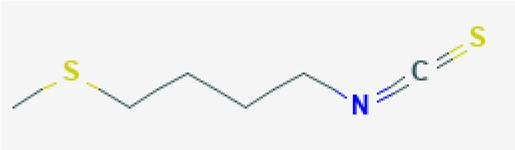
S
N
C
S

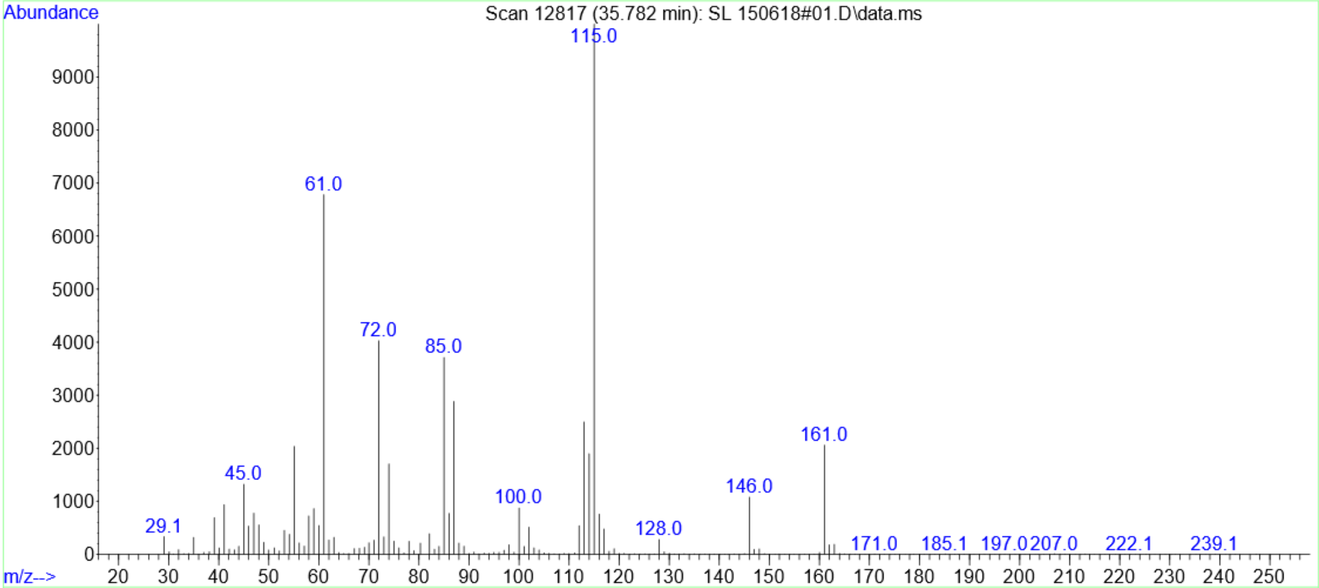
Abundance
Scan 12817 (35.782 min): SL 150618#01.D\data.ms
115.0
9000
8000
7000
6000
5000
4000
3000
2000
1000
0
61.0
72.0
85.0
161.0
45.0
146.0
100.0
29.1
128.0
171.0
185.1
197.0
207.0
222.1
239.1
m/z-->
20 30 40 50 60 70 80 90 100 110 120 130 140 150 160 170 180 190 200 210 220 230 240 250

24. Phenethyl isothiocyanate

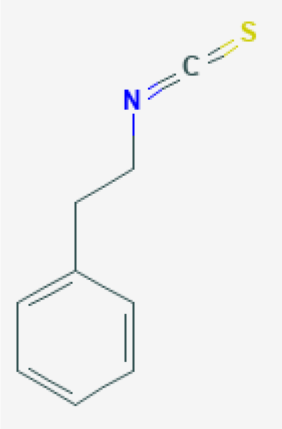

Library Searched : C:\Database\NIST11.L
Quality : 95
ID : Benzene, (2-isothiocyanatoethyl)-

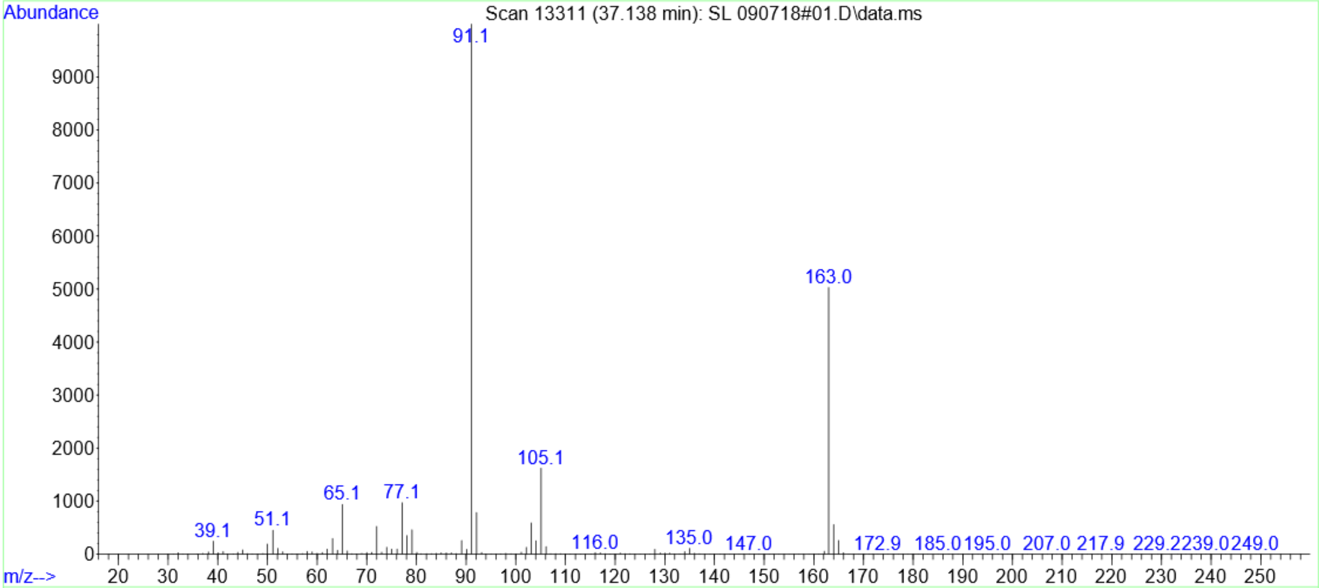

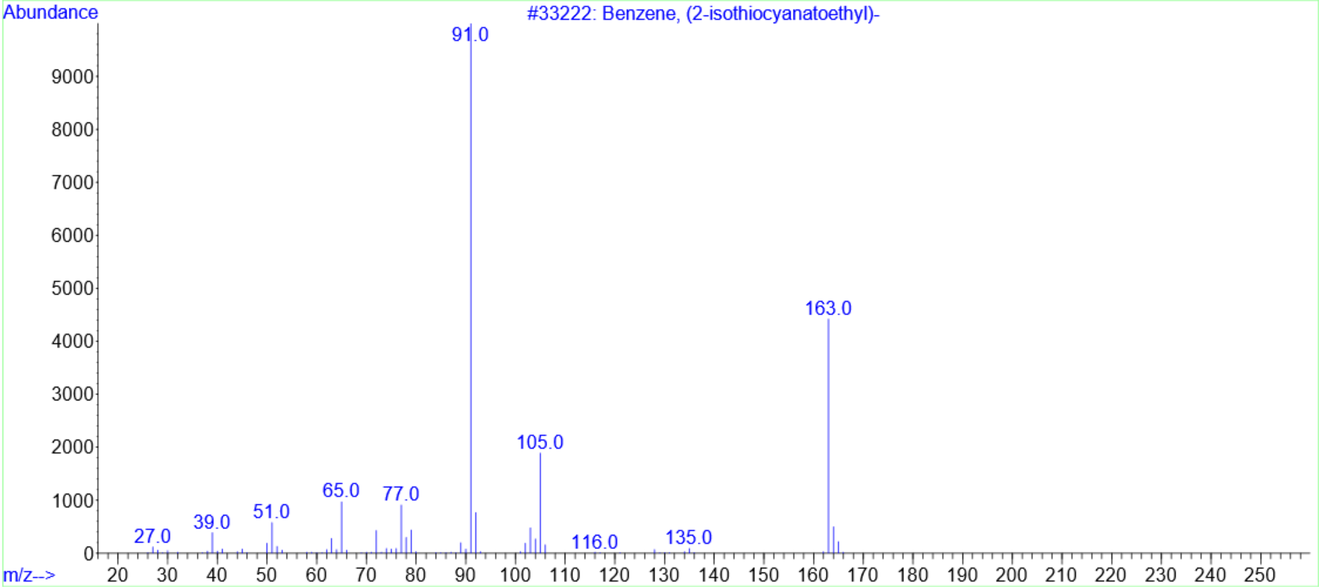

25. 1-penten-3-ol

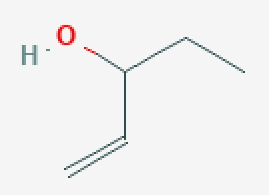

Library Searched : C:\Database\NIST11.L
Quality : 90
ID : 1-Penten-3-ol

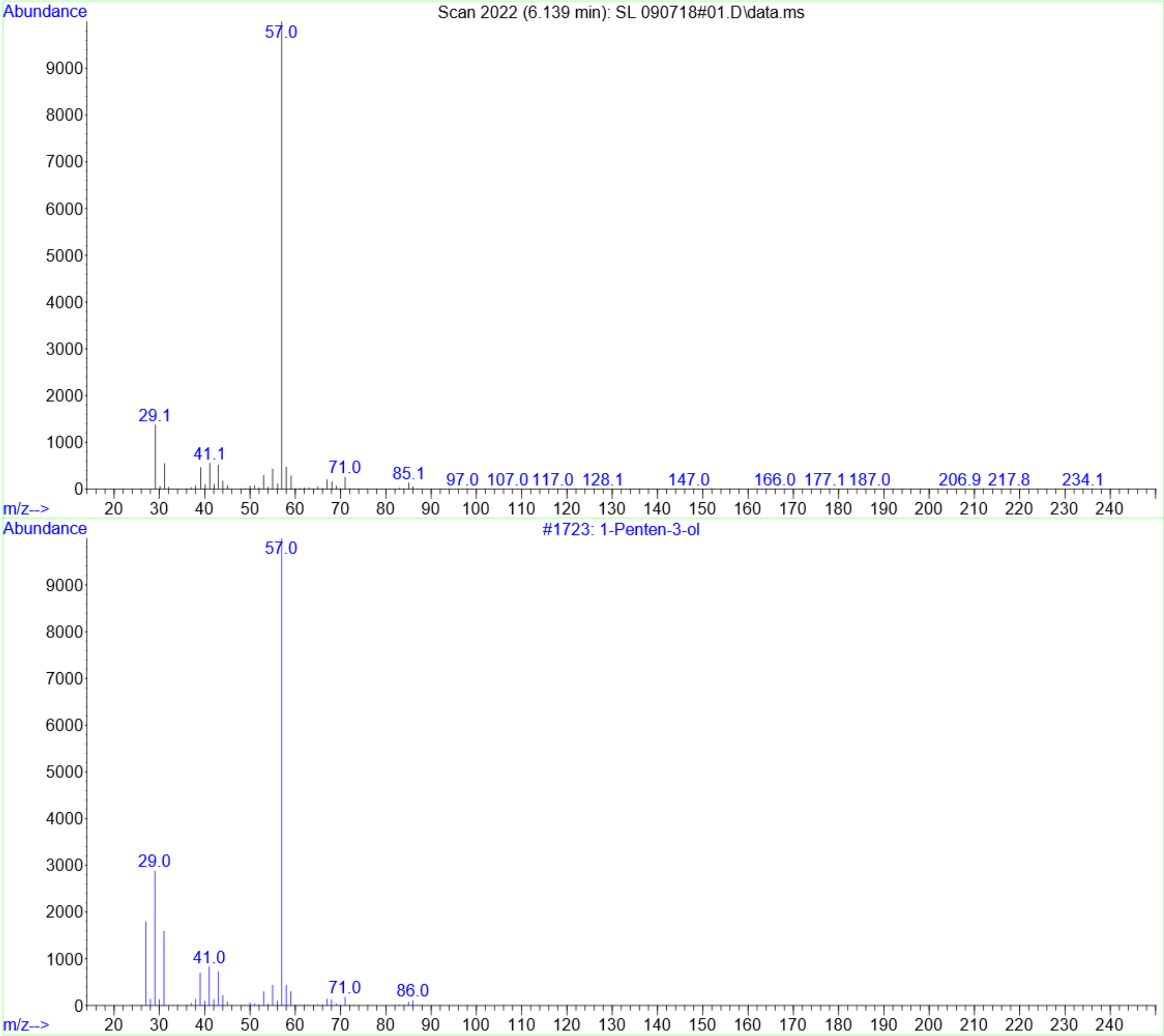

26. Pentan-1-ol

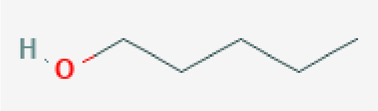

Library Searched : C:\Database\NIST11.L
Quality : 47
ID : 1-Pentanol

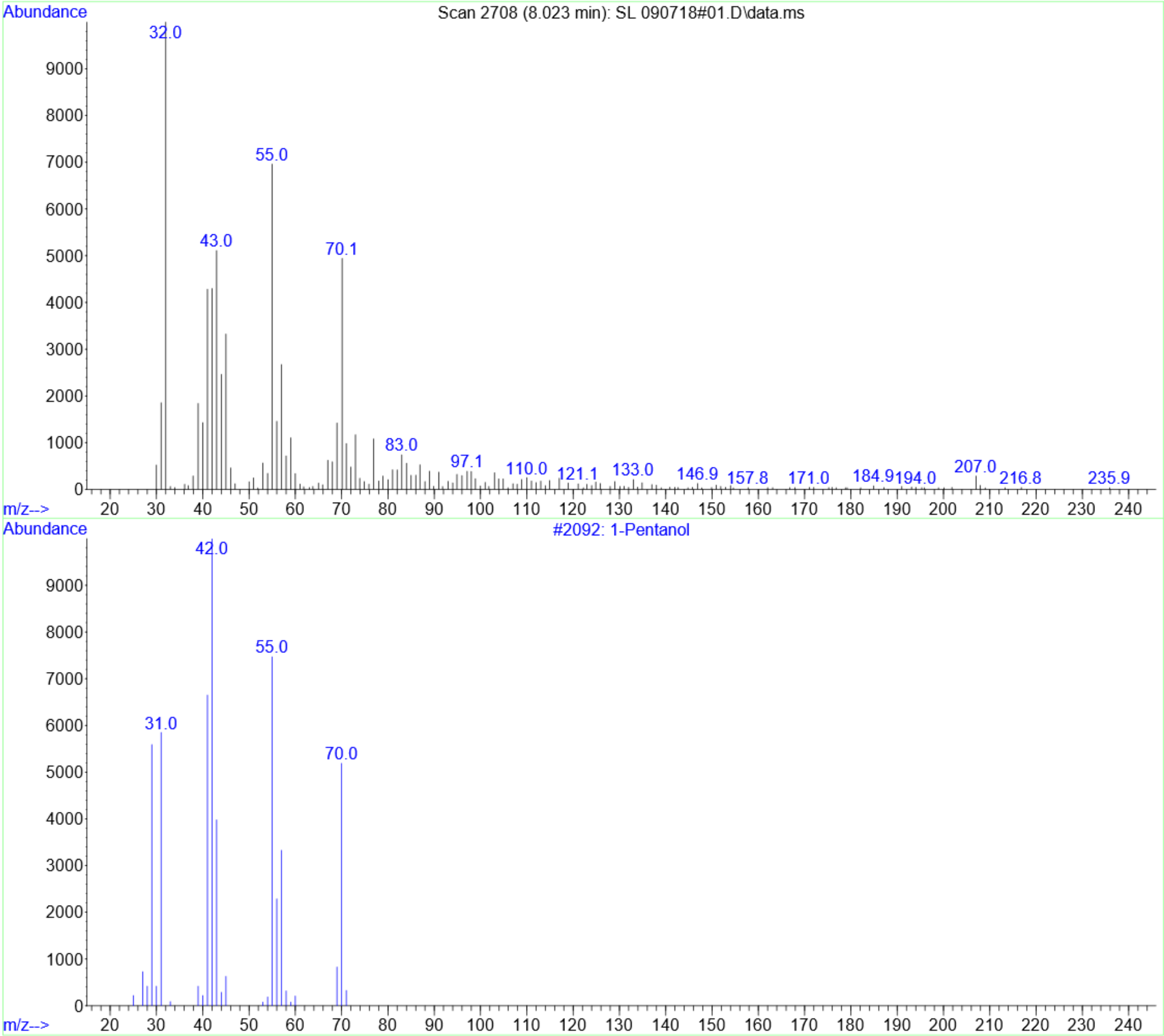

27. (*E*)-2-penten-1-ol

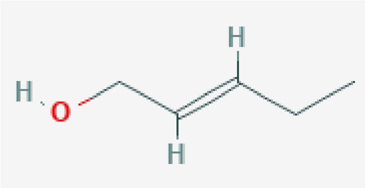

Library Searched : C:\Database\NIST11.L
Quality : 72
ID : 2-Penten-1-ol, (E)-

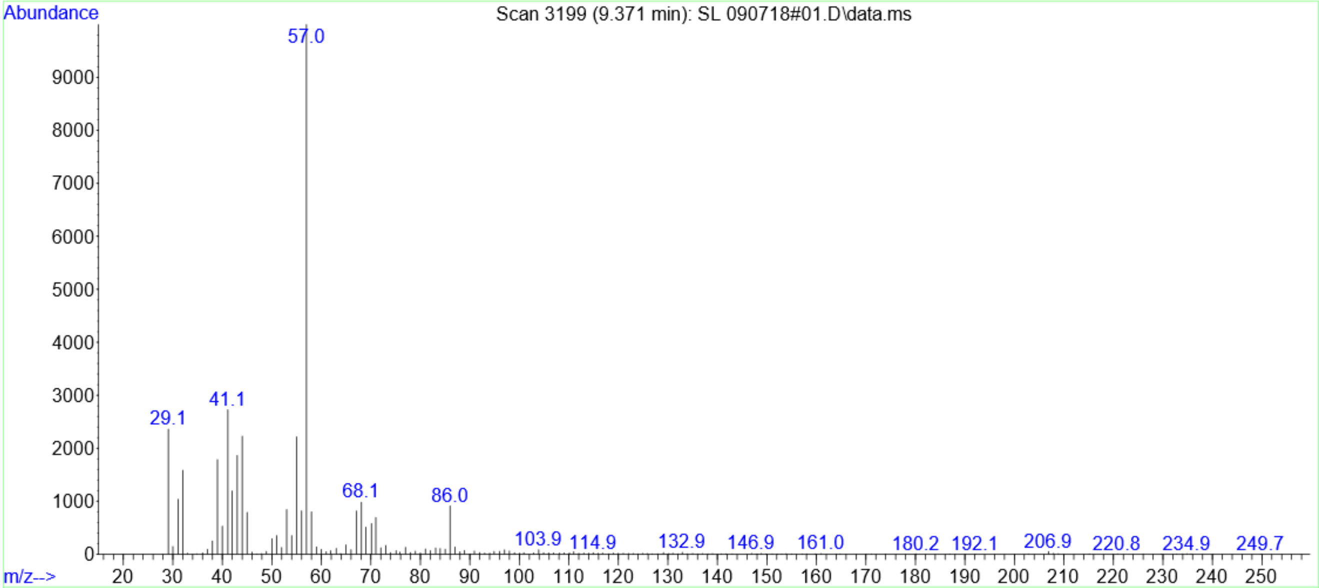

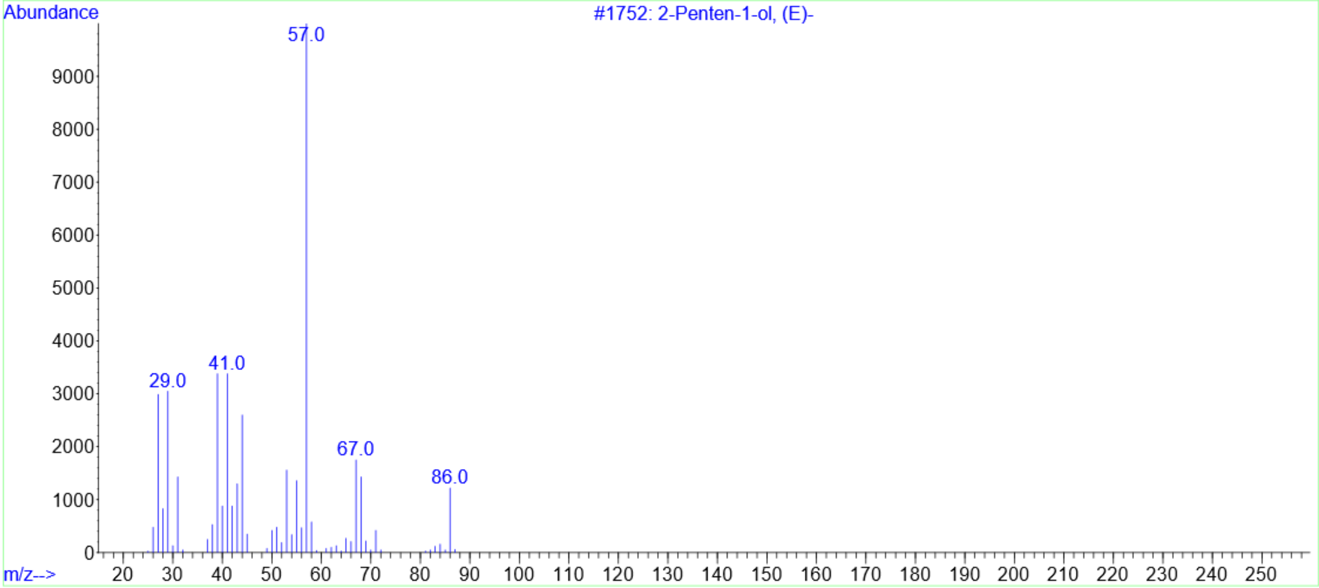

28. (*Z*)-2-penten-1-ol

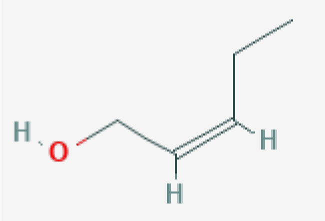

Library Searched : C:\Database\NIST11.L
Quality : 93
ID : 2-Penten-1-ol, (Z)-

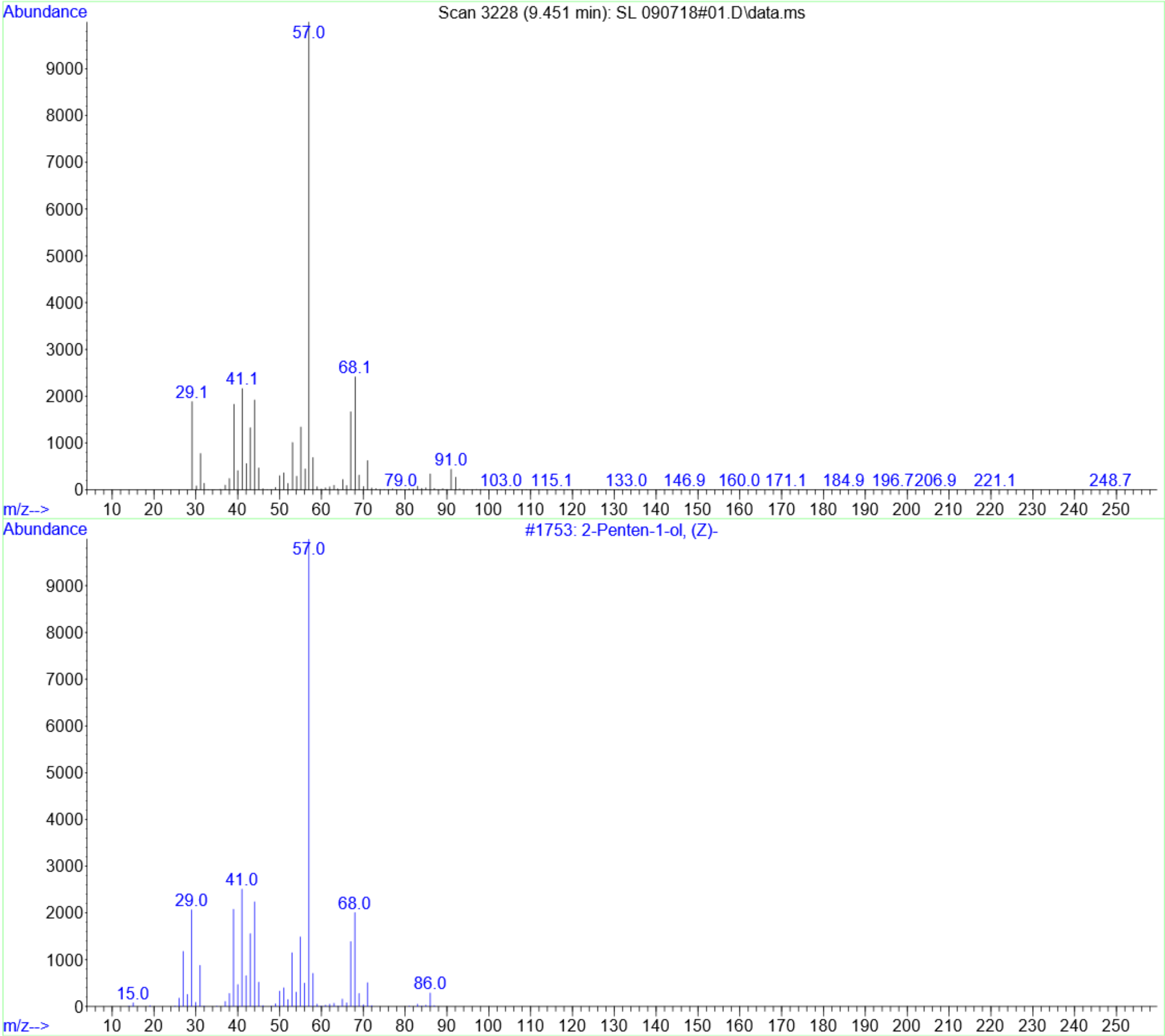

29. 1-propoxy-2-propanol

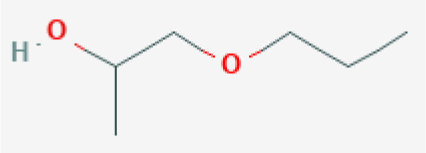

Library Searched : C:\Database\NIST11.L
Quality : 87
ID : 2-Propanol, 1-propoxy-

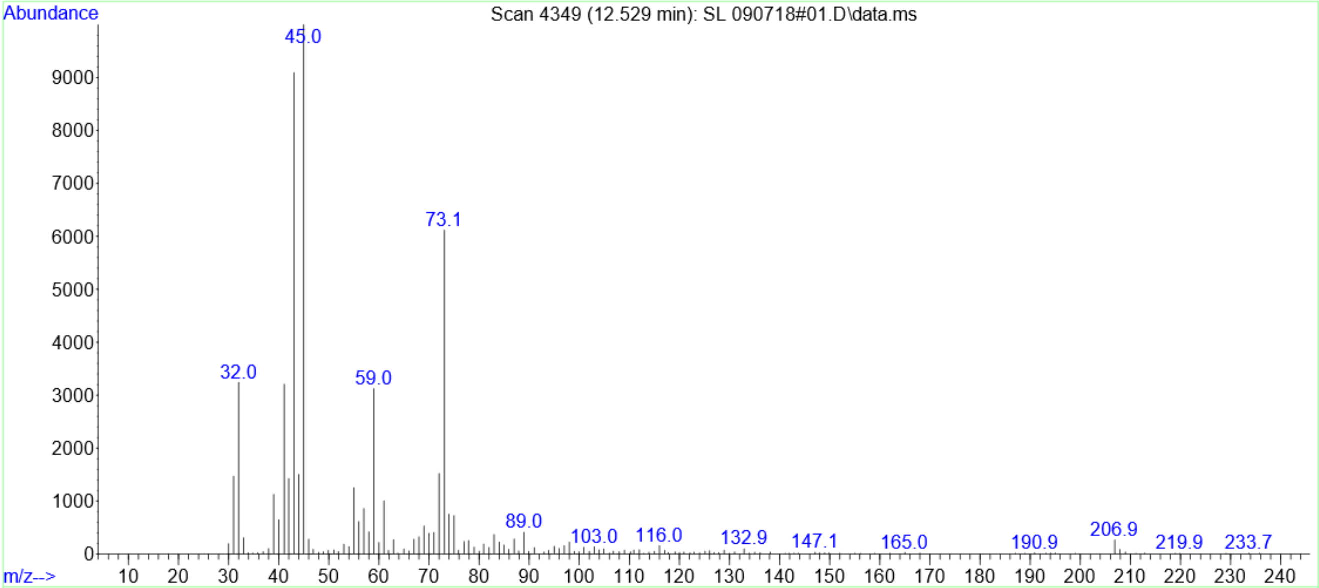

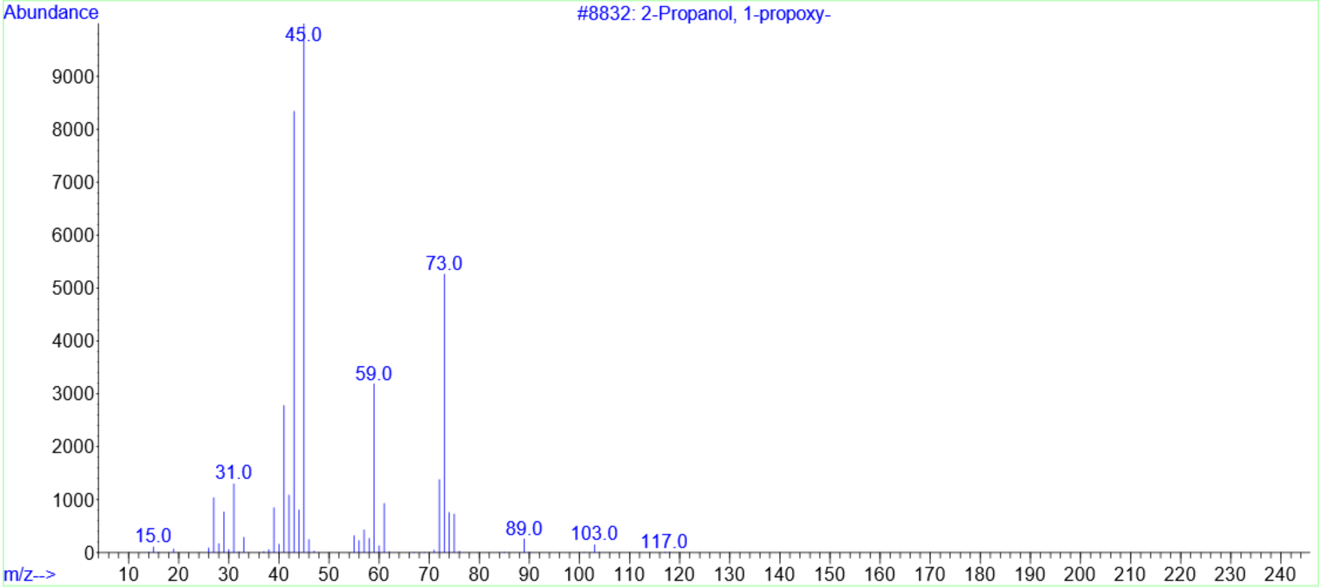

30. (*E*)-3-hexen-1-ol

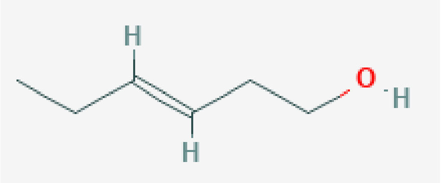

Library Searched : C:\Database\NIST11.L
Quality : 95
ID : 3-Hexen-1-ol, (E)-

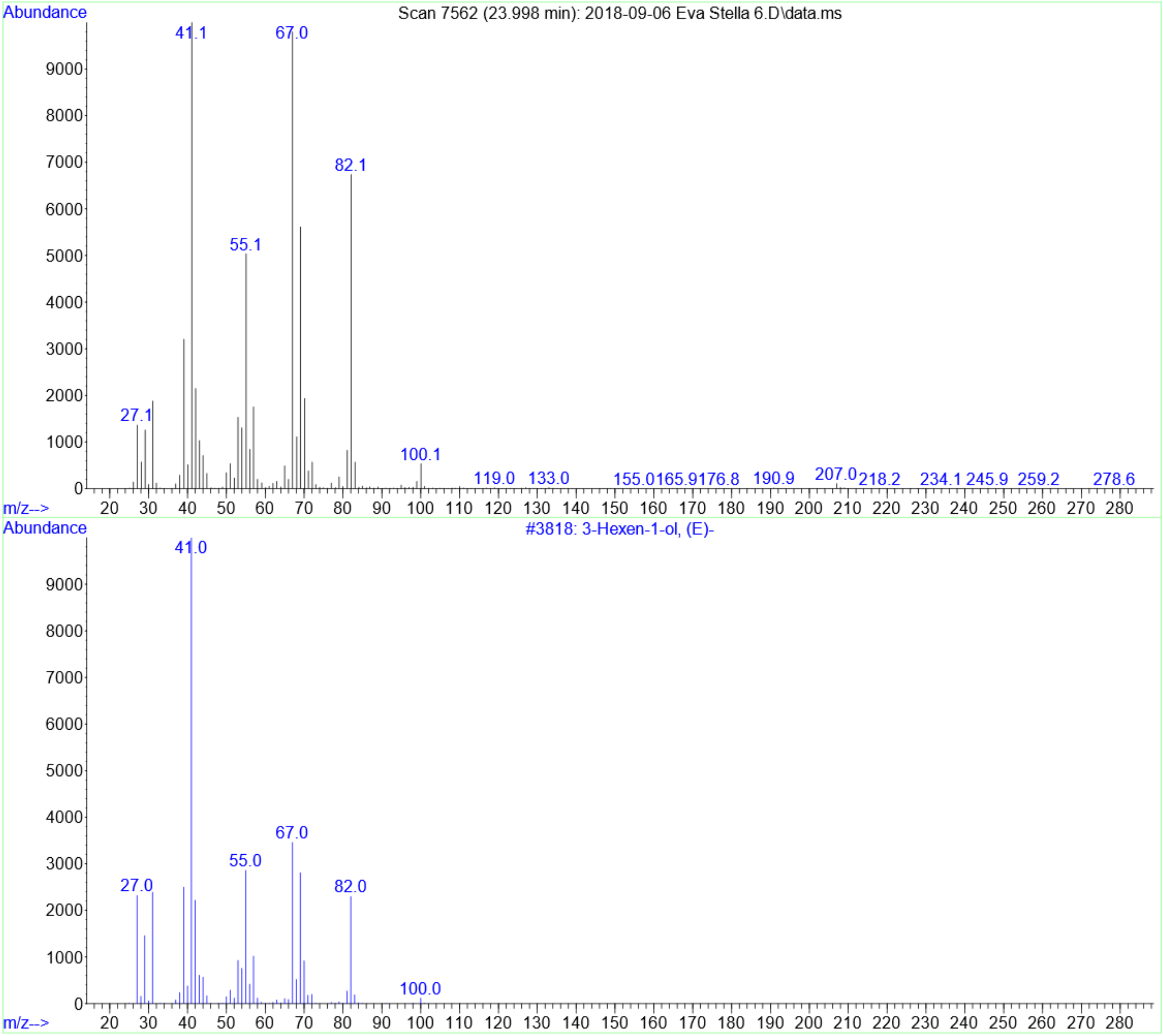

31. (*Z*)-3-hexen-1-ol

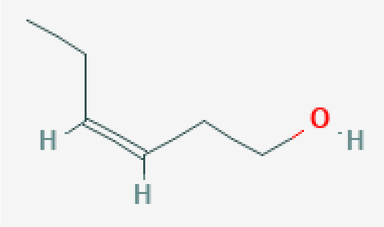

Library Searched : C:\Database\NIST11.L
Quality : 91
ID : 3-Hexen-1-ol, (Z)-

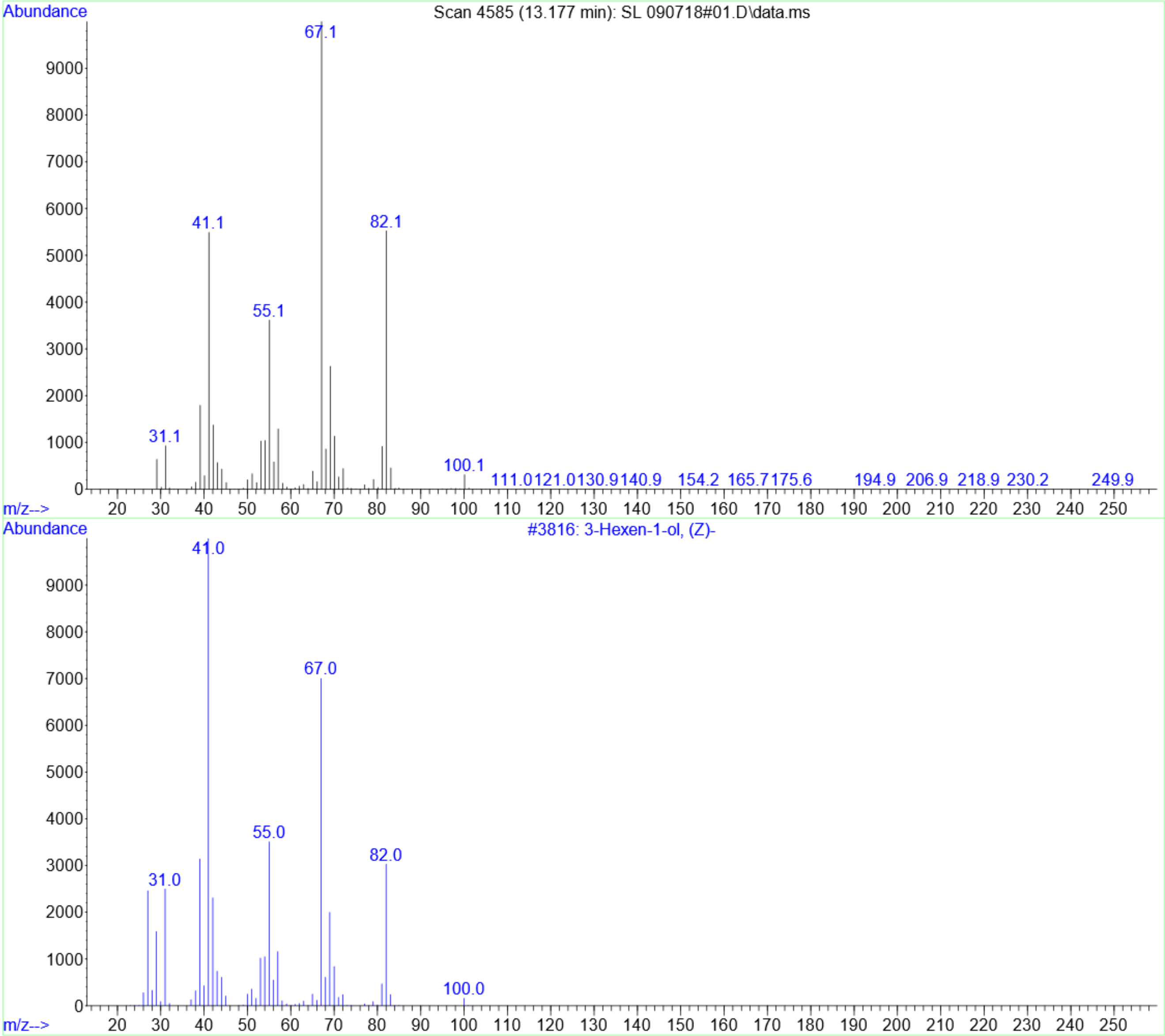

32. 2-hexen-1-ol

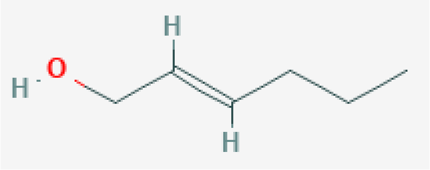

Library Searched : C:\Database\NIST11.L
Quality : 91
ID : 2-Hexen-1-ol, (E)-

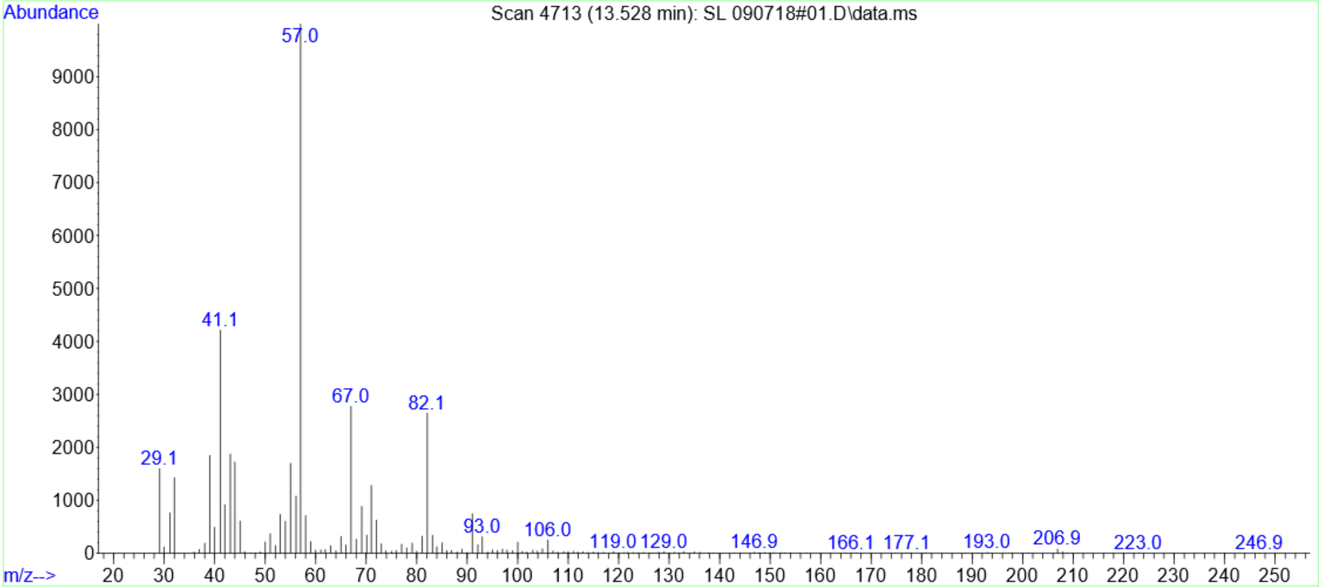

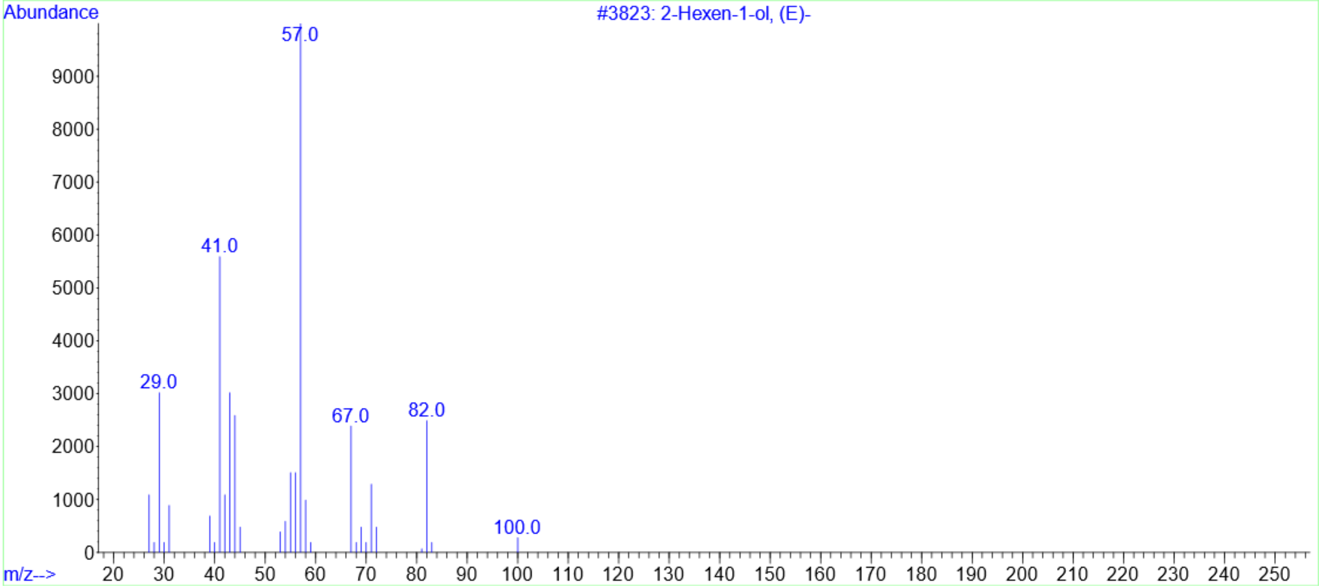

33. Hexan-1-ol

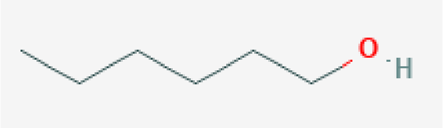

Library Searched : C:\Database\NIST11.L
Quality : 90
ID : 1-Hexanol

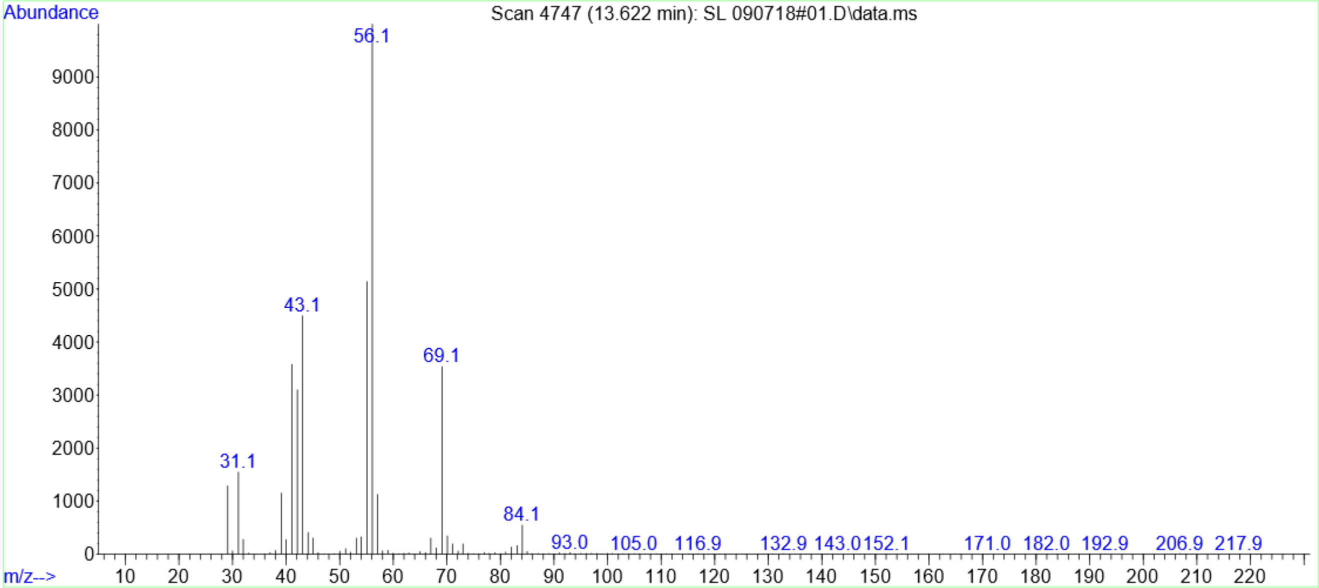

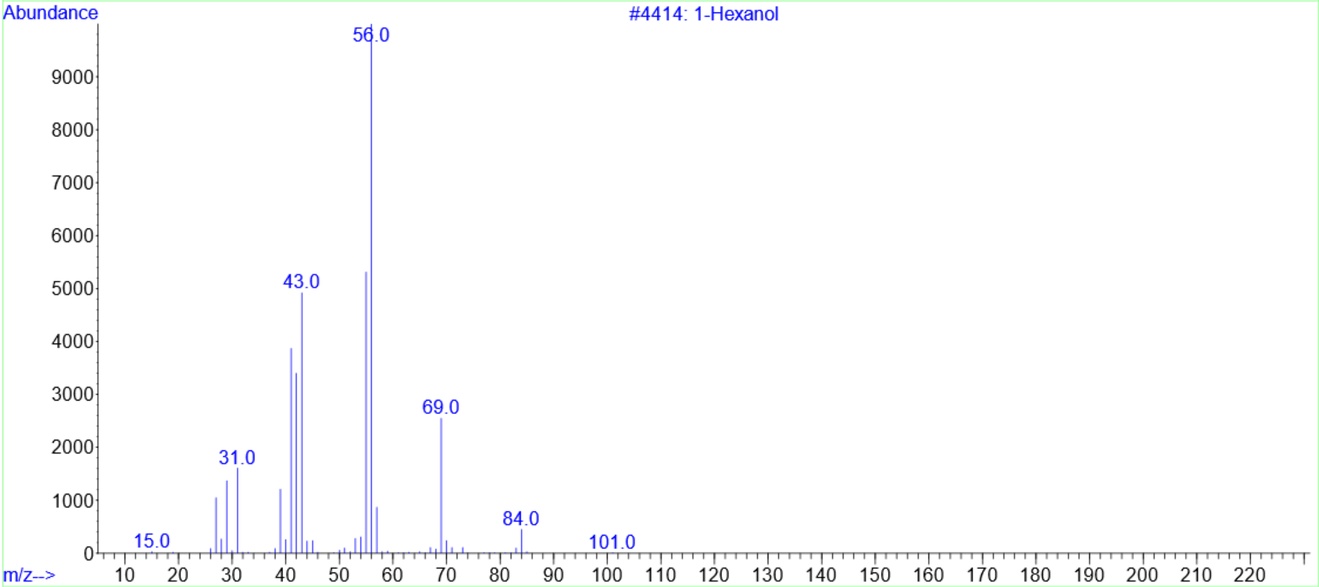

34. 1-octen-3-ol

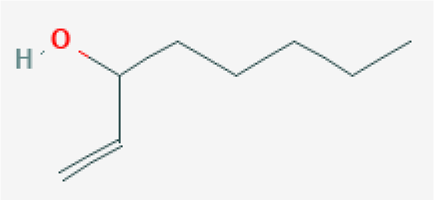

Library Searched : C:\Database\NIST11.L
Quality : 47
ID : 1-Octen-3-ol

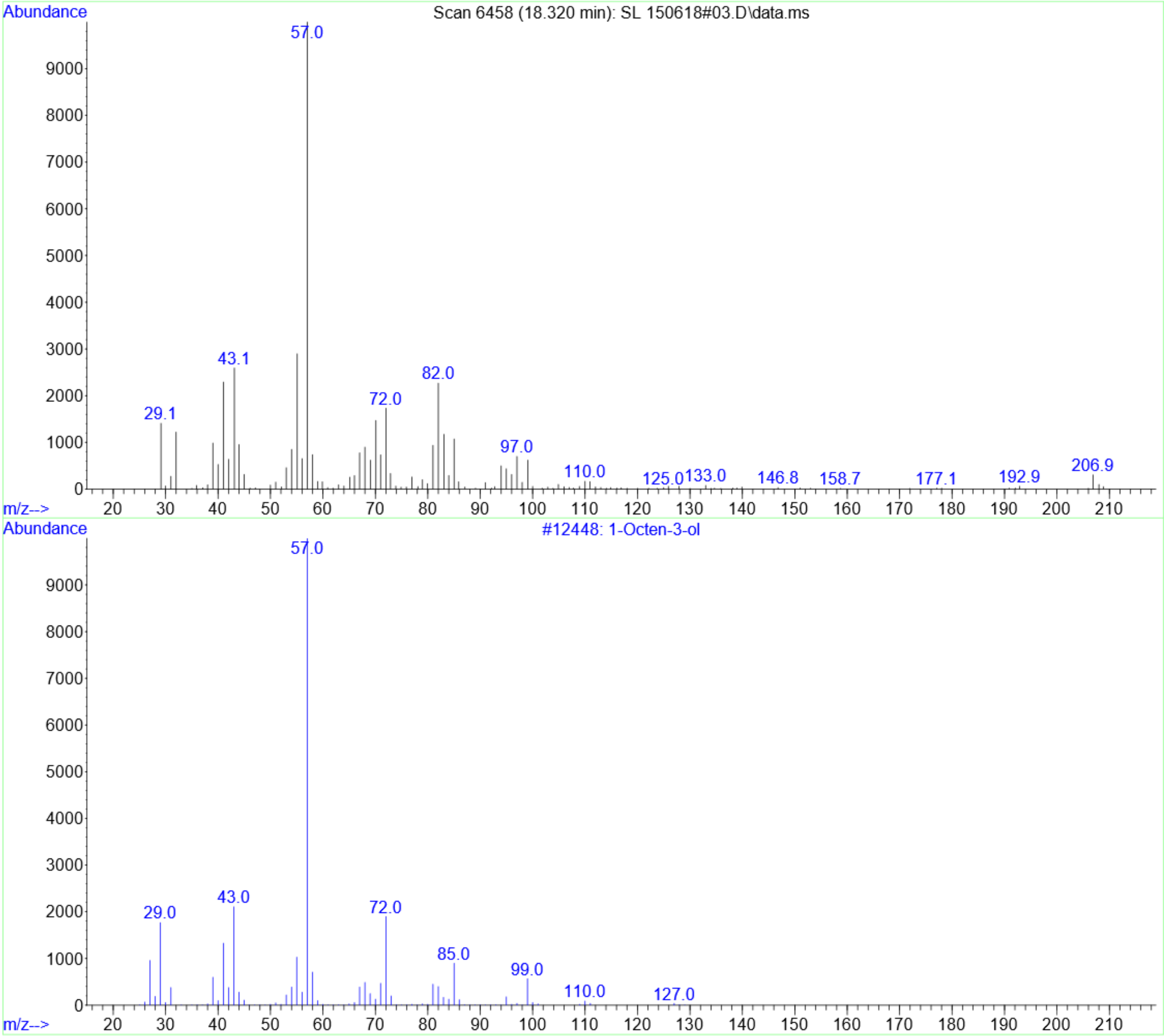

35. 2-ethylhexanol

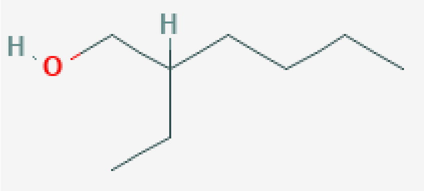

Library Searched : C:\Database\NIST11.L
Quality : 86
ID : 1-Hexanol, 2-ethyl-

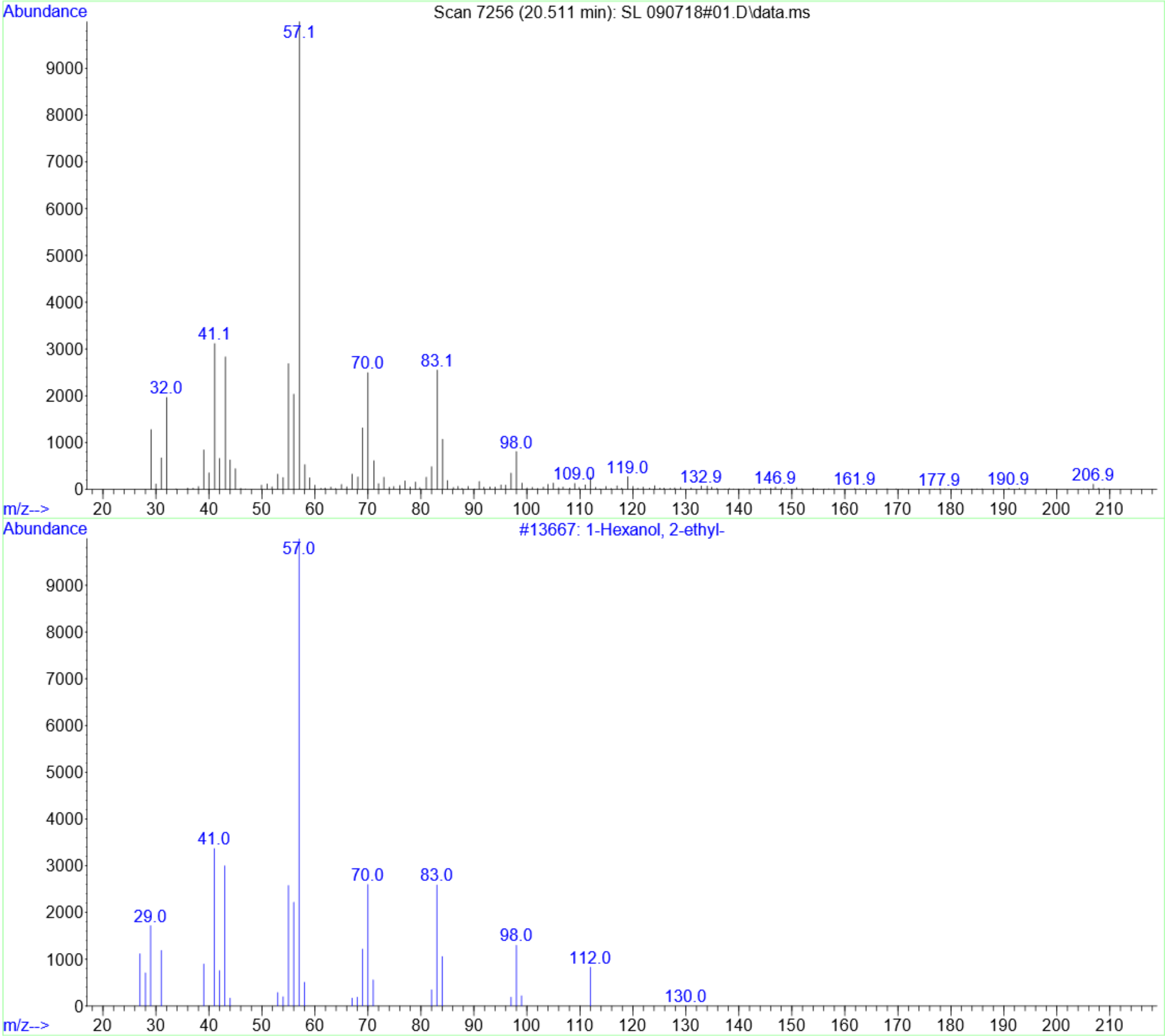

36. Benzyl alcohol

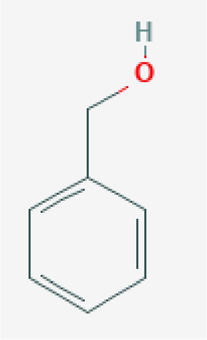

Library Searched : C:\Database\Adams.L
Quality : 97
ID : 8.77 Benzyl alcohol

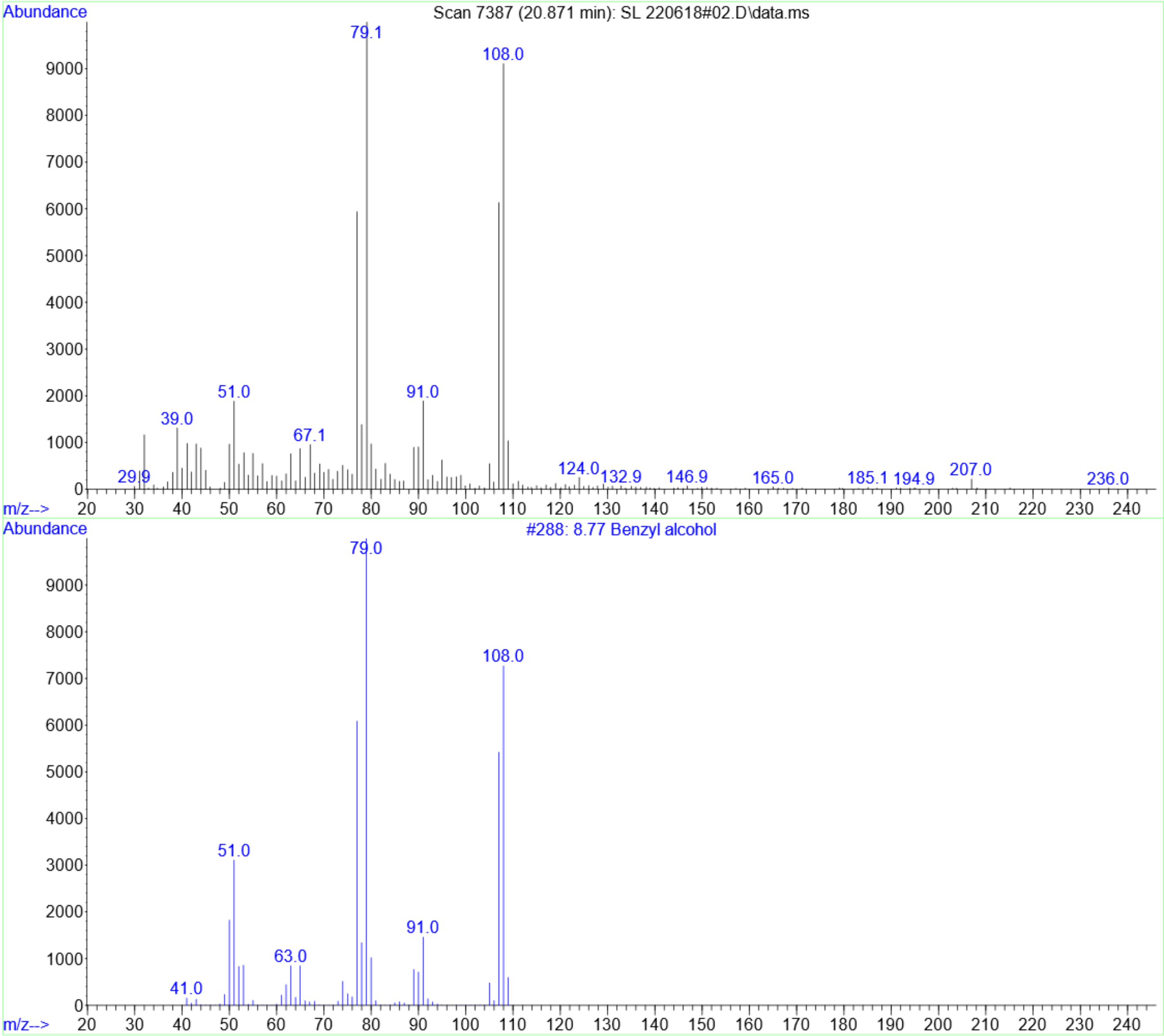

37. 2-phenylethanol

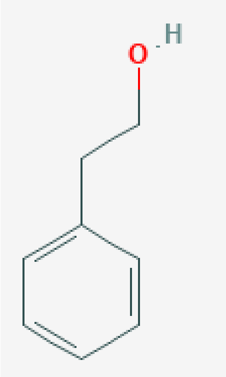

Library Searched : C:\Database\NIST11.L
Quality : 94
ID : Phenylethyl Alcohol

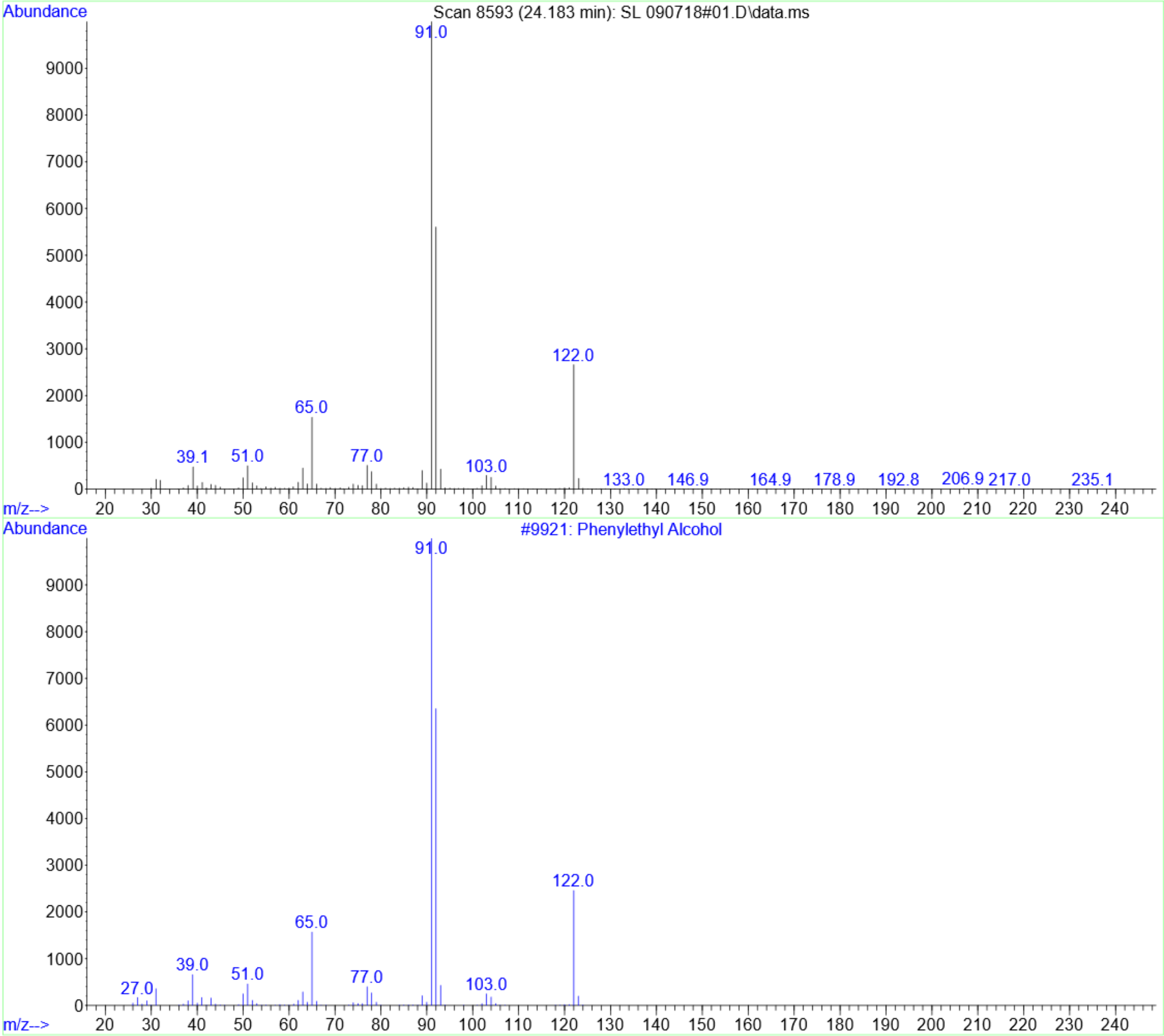

38. 1-nonanol

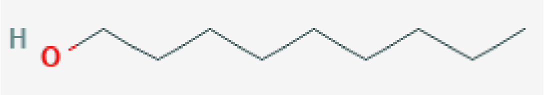

Library Searched : C:\Database\Adams.L
Quality : 78
ID : 14.29 Nonanol<n->

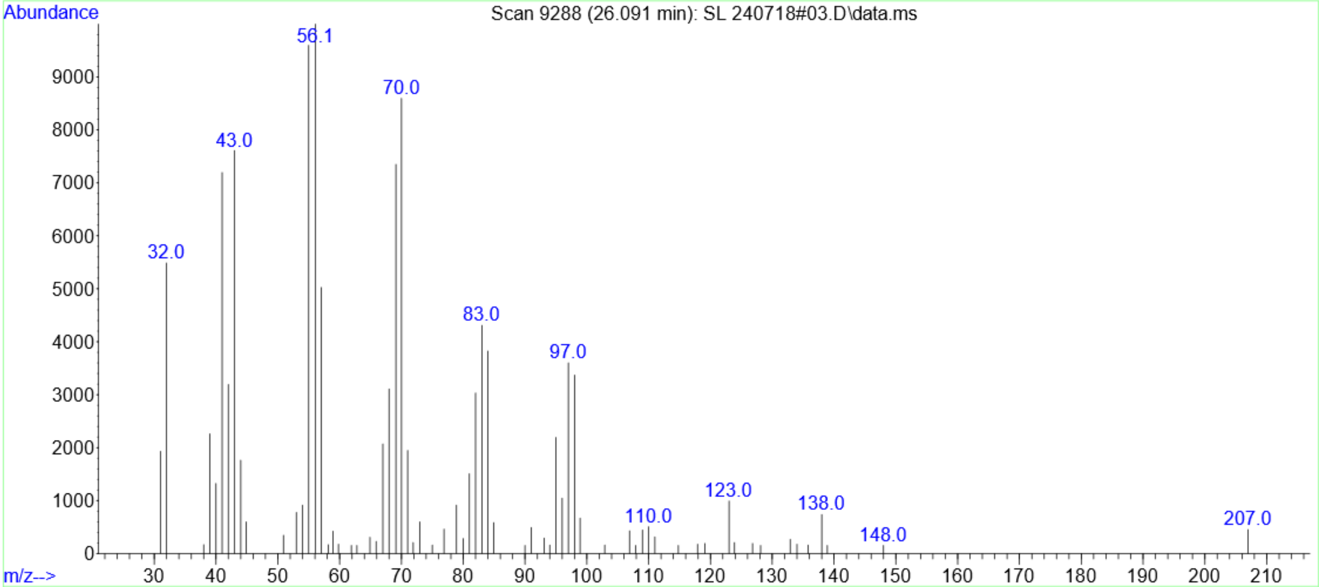

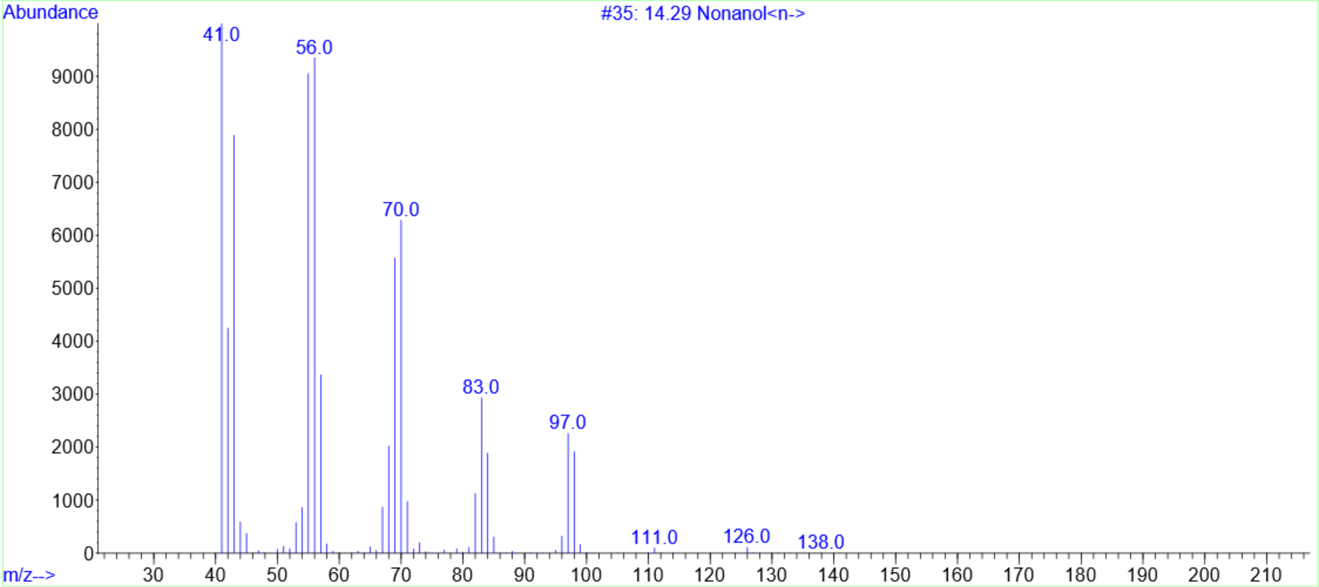

39. Terpinen-4-ol

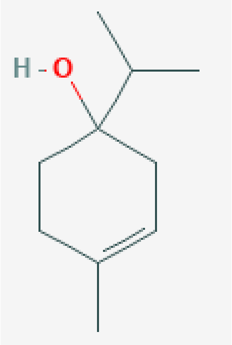

Library Searched : C:\Database\Adams.L
Quality : 93
ID : 14.66 Terpinen-4-ol

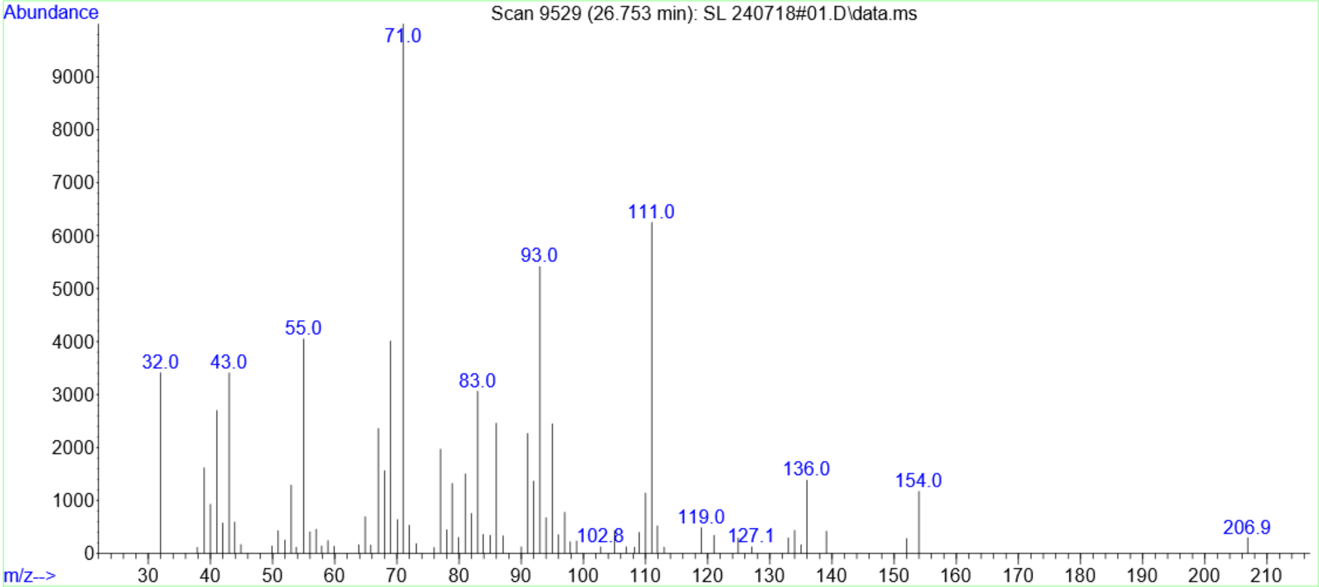

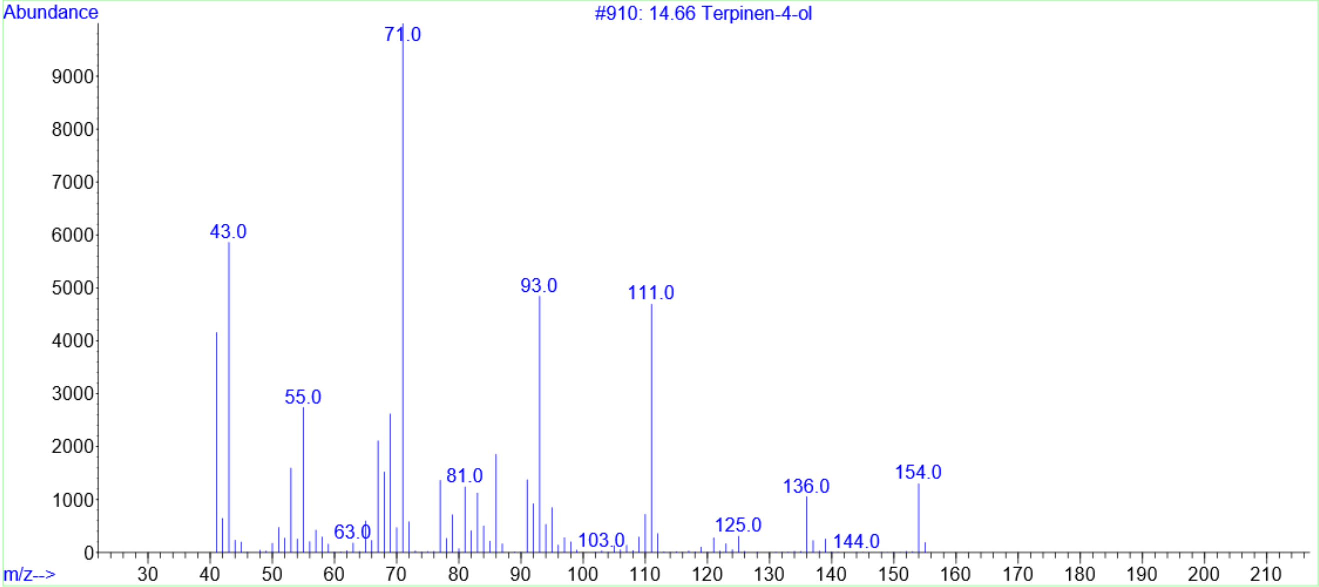

40. 2-pentanal

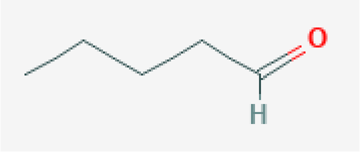

Library Searched : C:\Database\Adams.L
Quality : 87
ID : 2.27 Pentanal

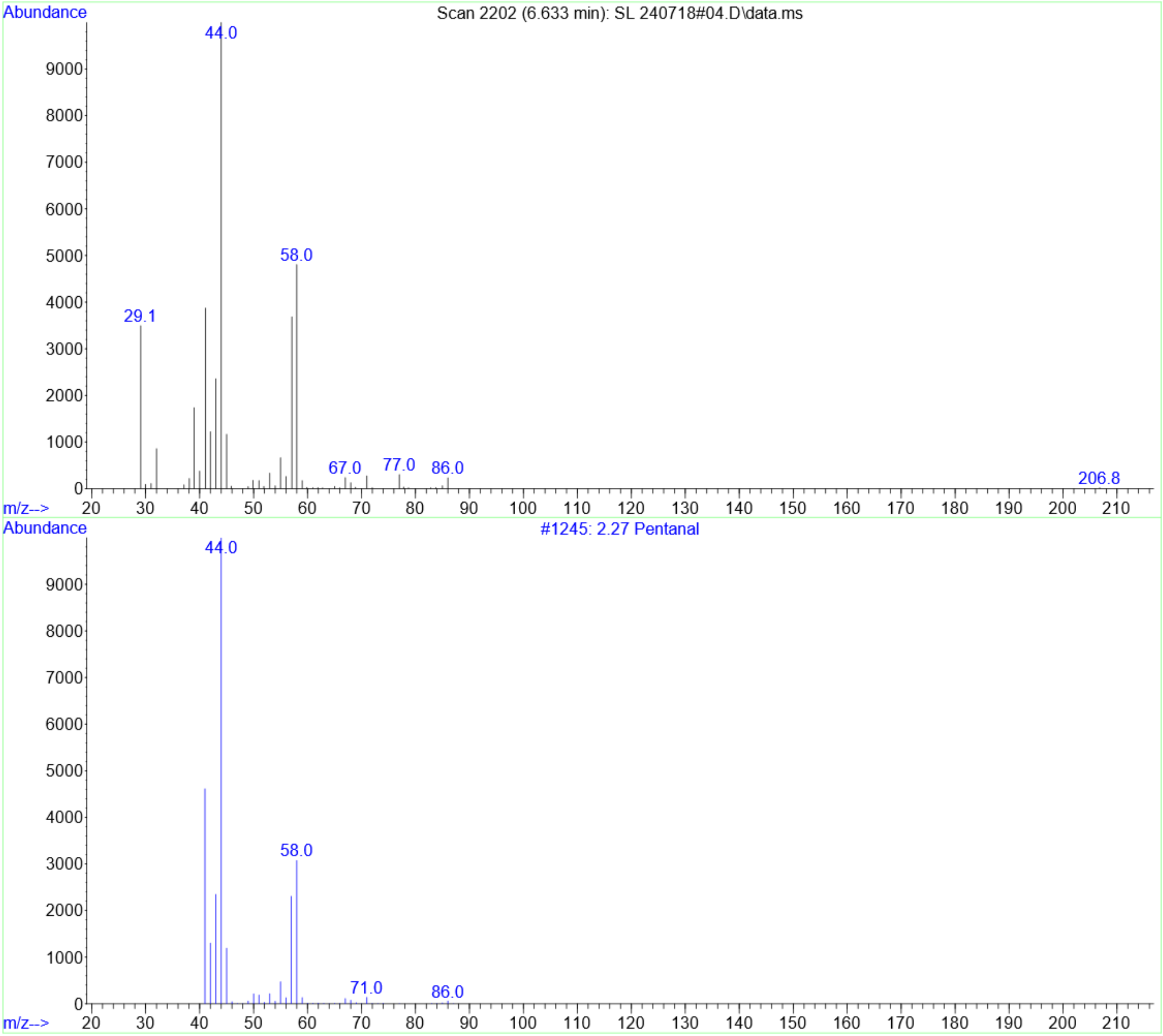

41. 2-pentenal

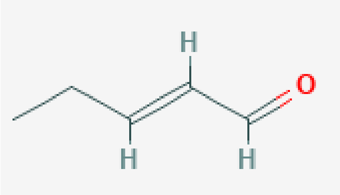

Library Searched : C:\Database\NIST11.L
Quality : 81
ID : 2-Pentenal, (E)-

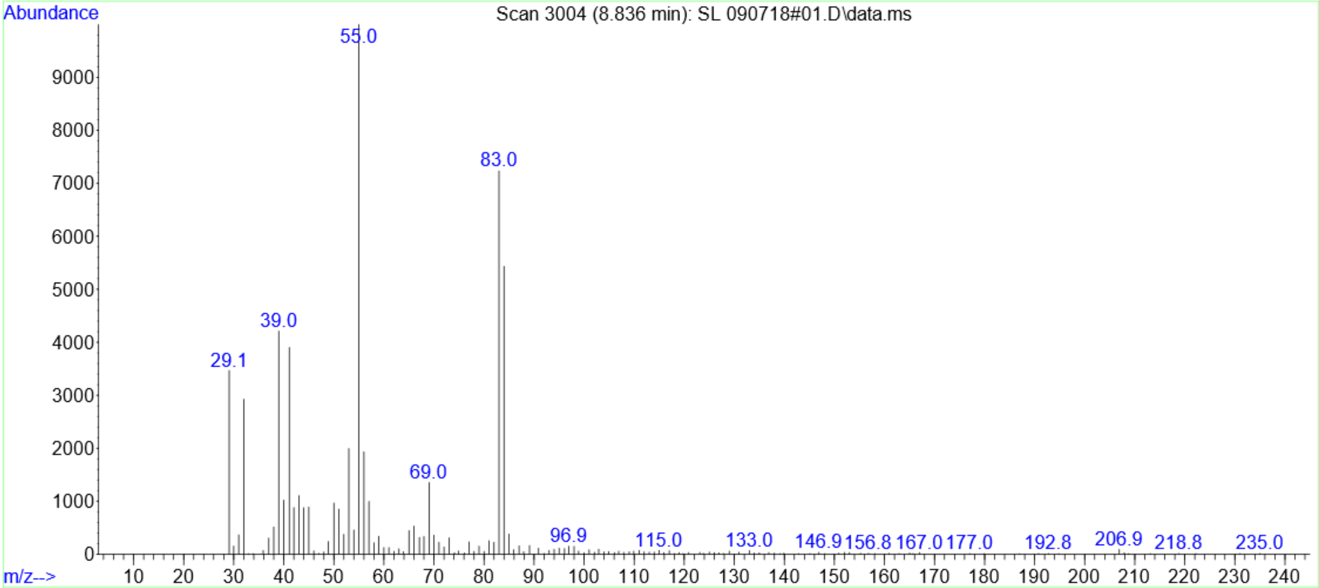

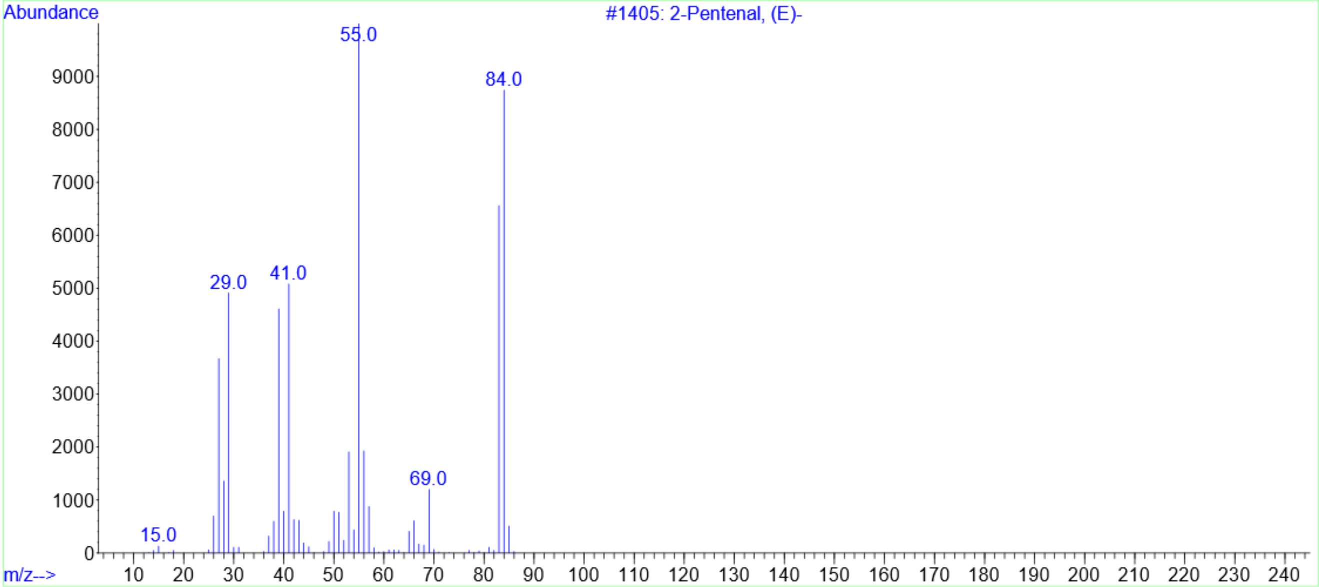

42. 3-hexenal

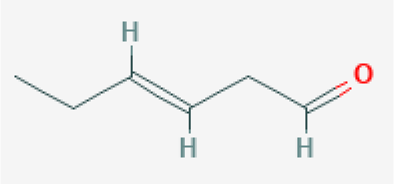

Library Searched : C:\Database\NIST11.L
Quality : 90
ID : 3-Hexenal

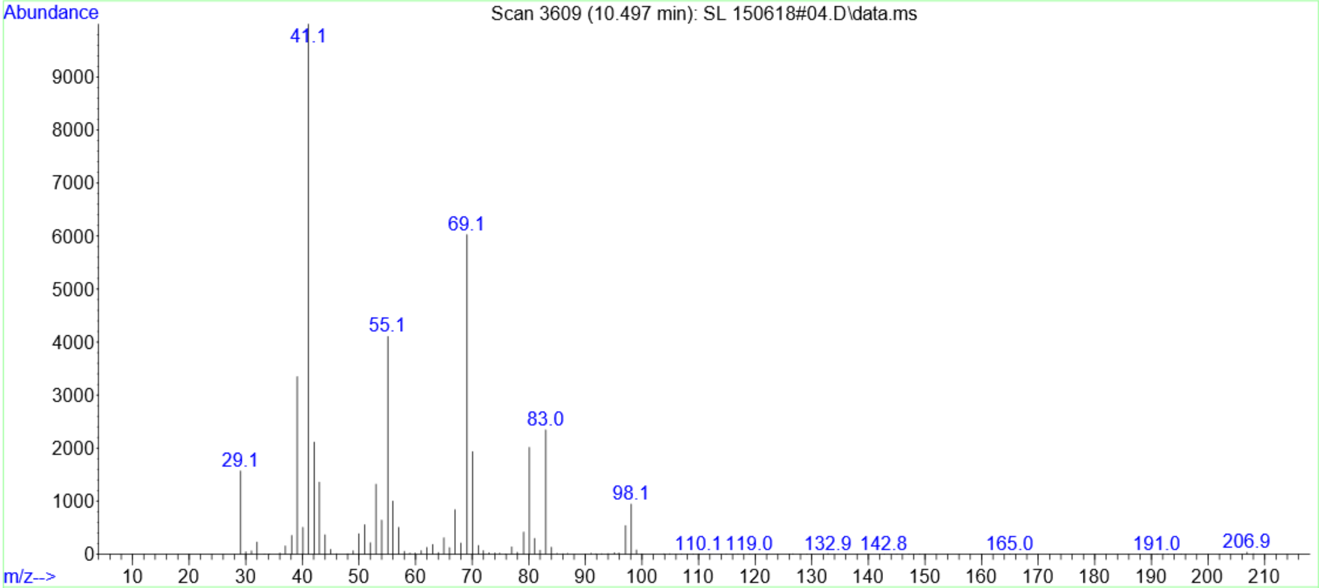

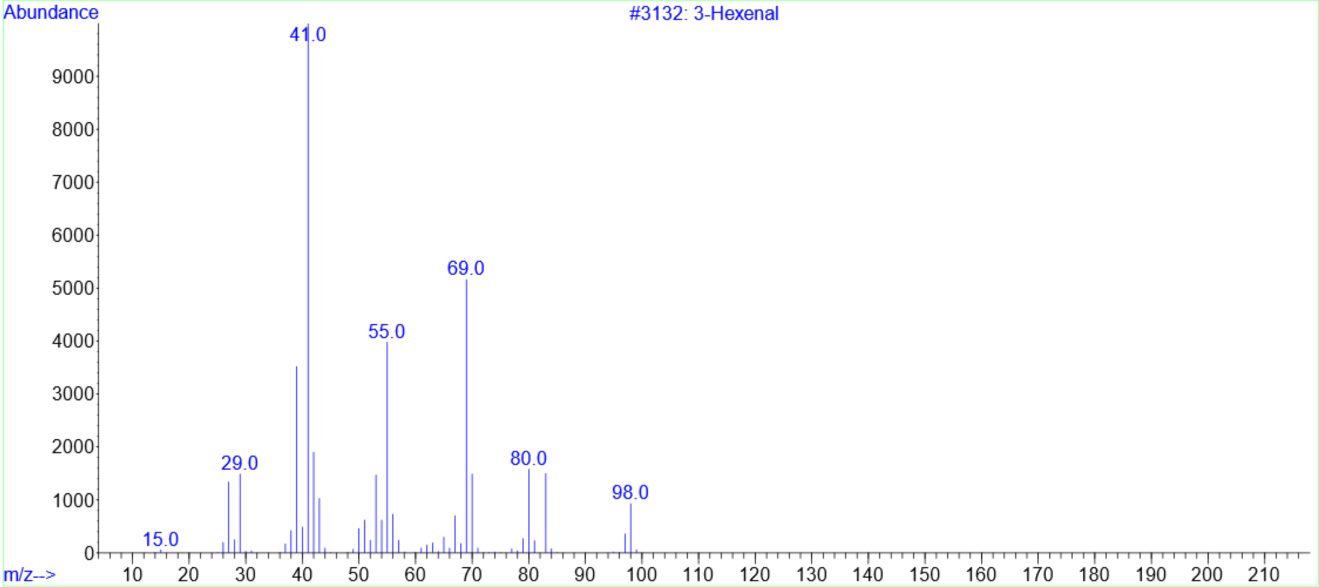

43. Hexanal

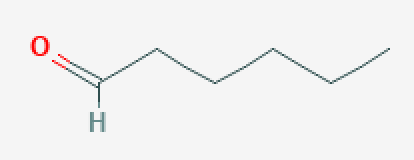

Library Searched : C:\Database\NIST11.L
Quality : 90
ID : Hexanal

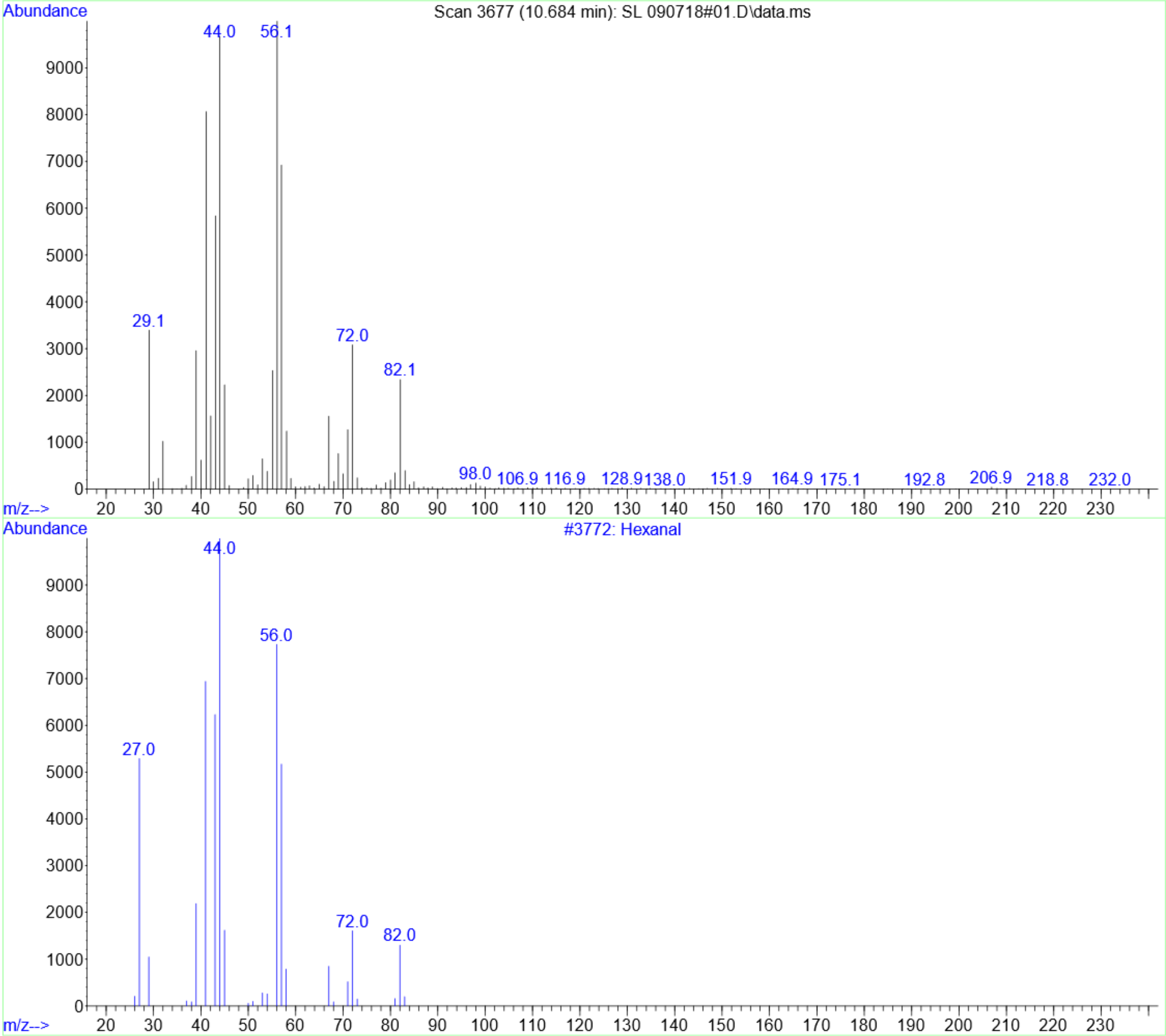

44. (*E*)-2-hexenal

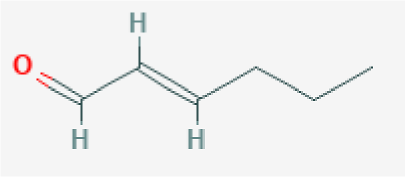

Library Searched : C:\Database\NIST11.L
Quality : 98
ID : 2-Hexenal, (E)-

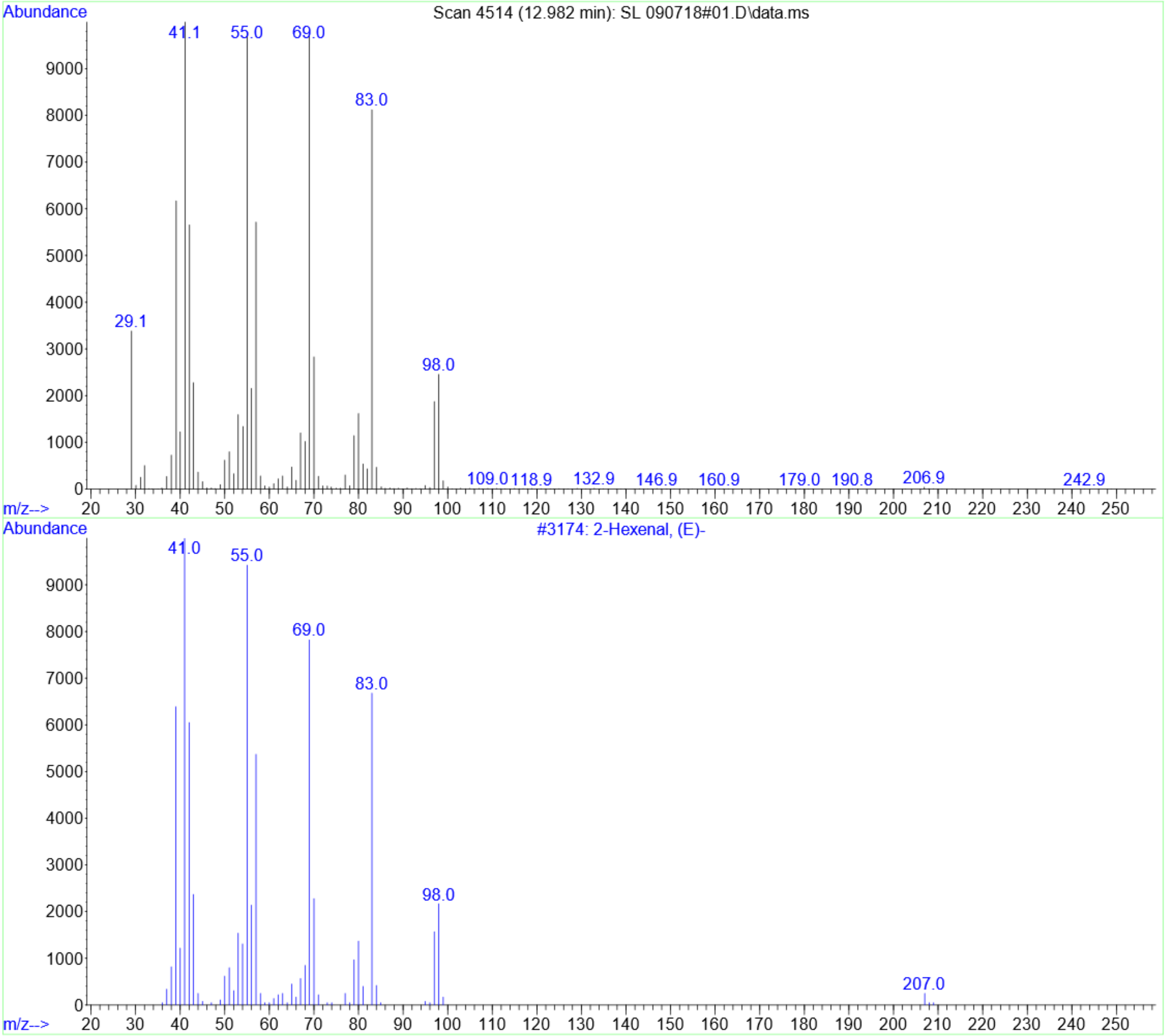

45. 4-heptenal

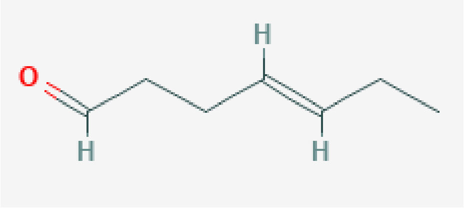

Library Searched : C:\Database\NIST11.L
Quality : 50
ID : 4-Heptenal, (Z)-

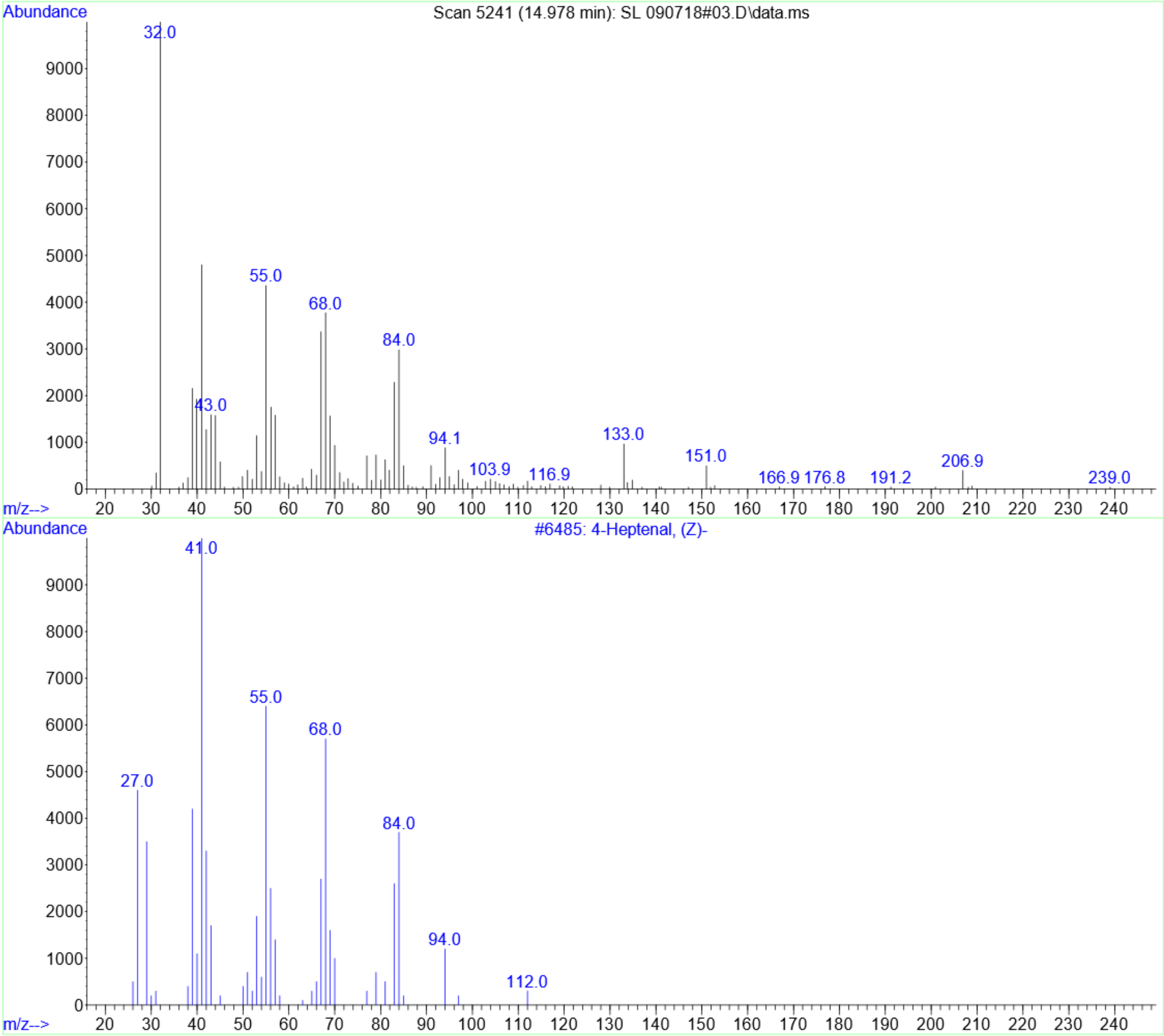

46. Heptanal

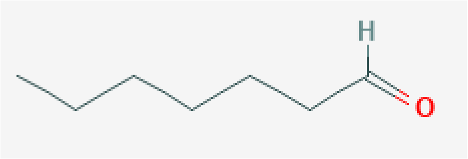

Library Searched : C:\Database\NIST11.L
Quality : 92
ID : Heptanal

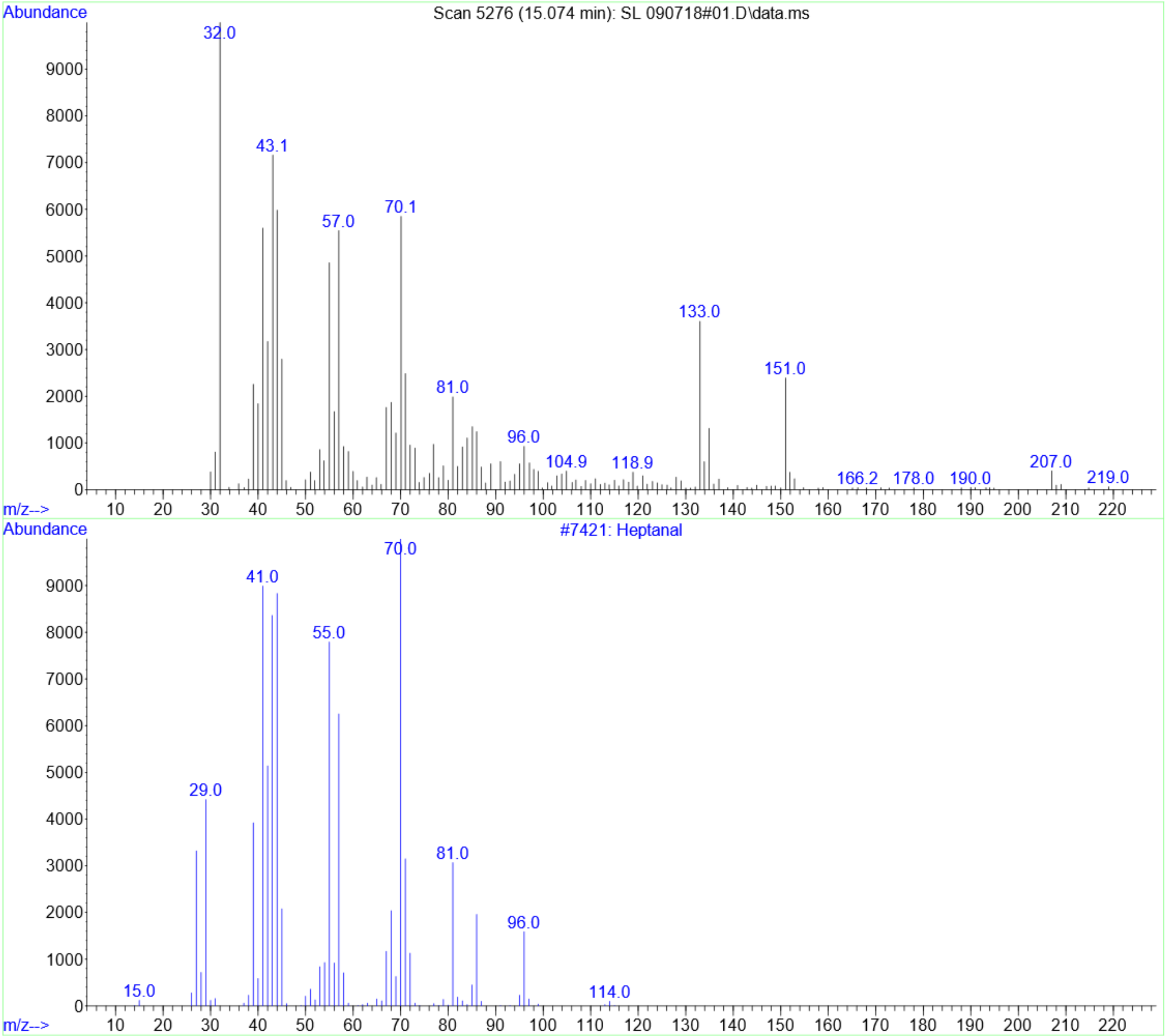

47. 2,4-hexadienal

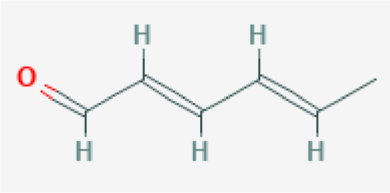

Library Searched : C:\Database\NIST11.L
Quality : 70
ID : 2,4-Hexadienal, (E,E)-

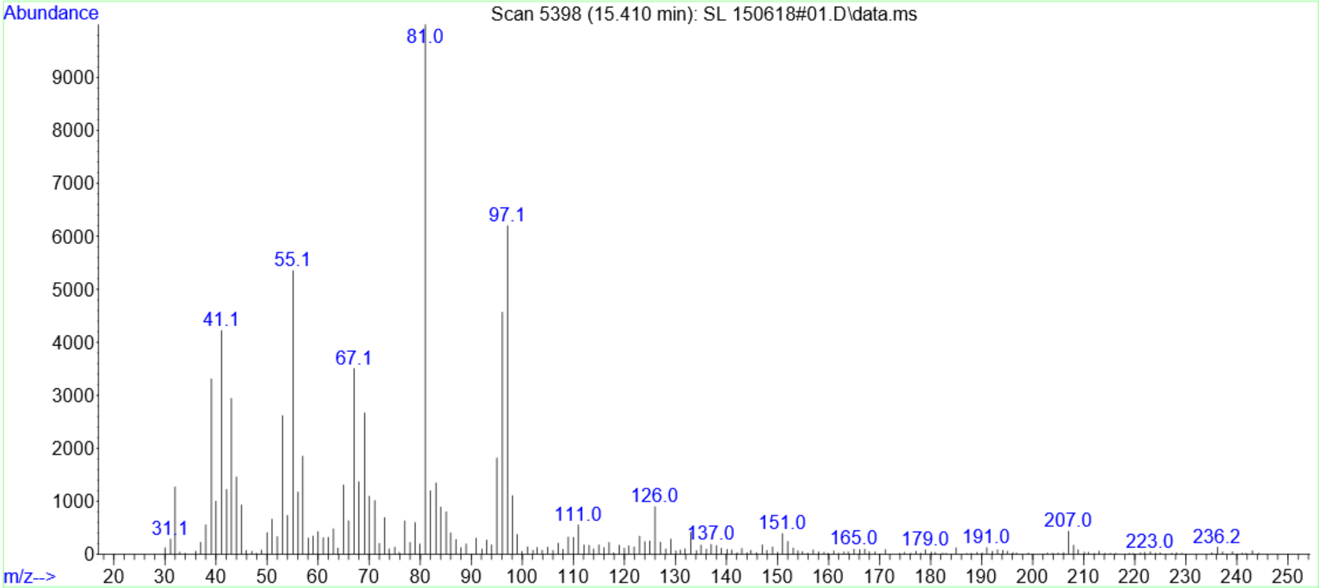

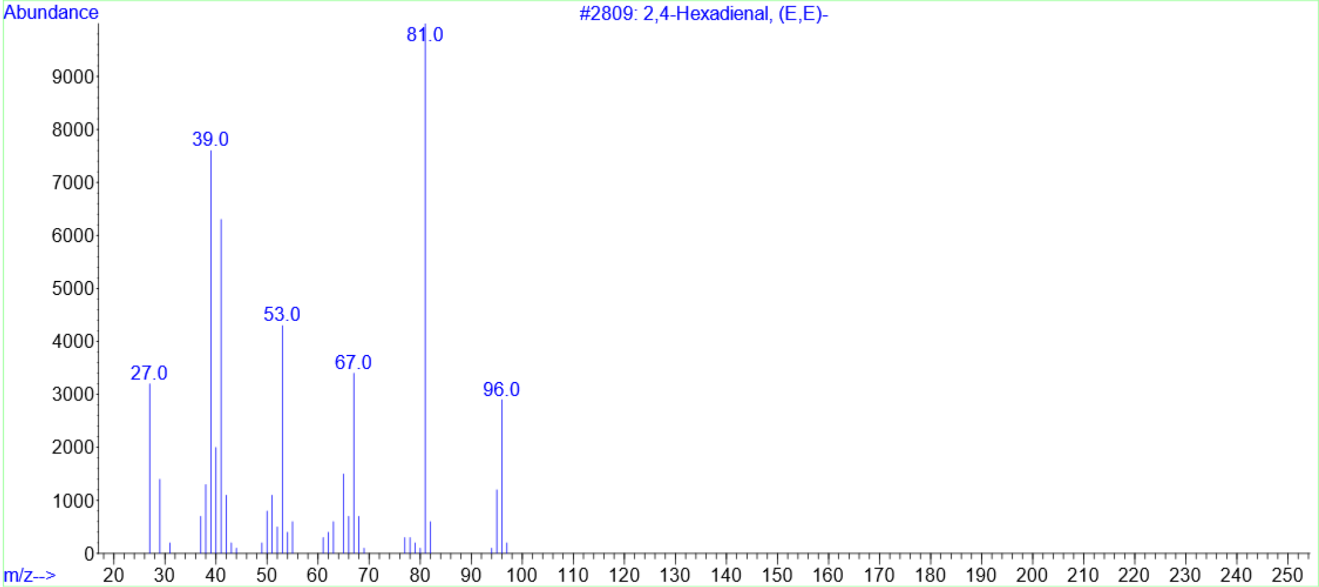

48. Benzaldehyde

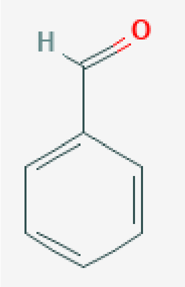

Library Searched : C:\Database\NIST11.L
Quality : 95
ID : Benzaldehyde

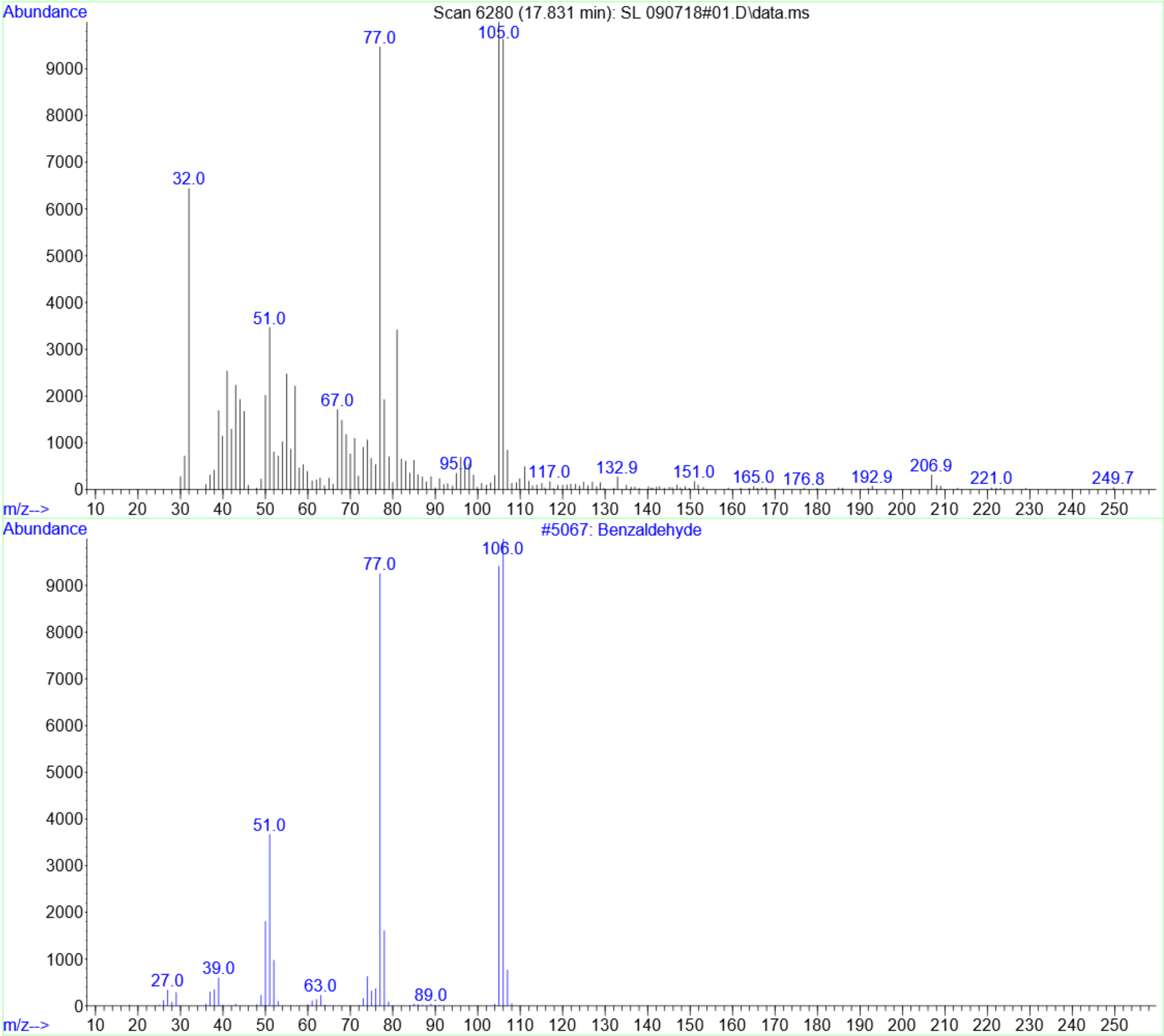

49. 2,4-heptadienal isomer 1

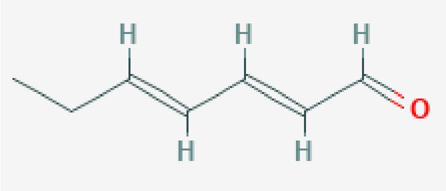

Library Searched : C:\Database\NIST11.L
Quality : 93
ID : 2,4-Heptadienal, (E,E)-

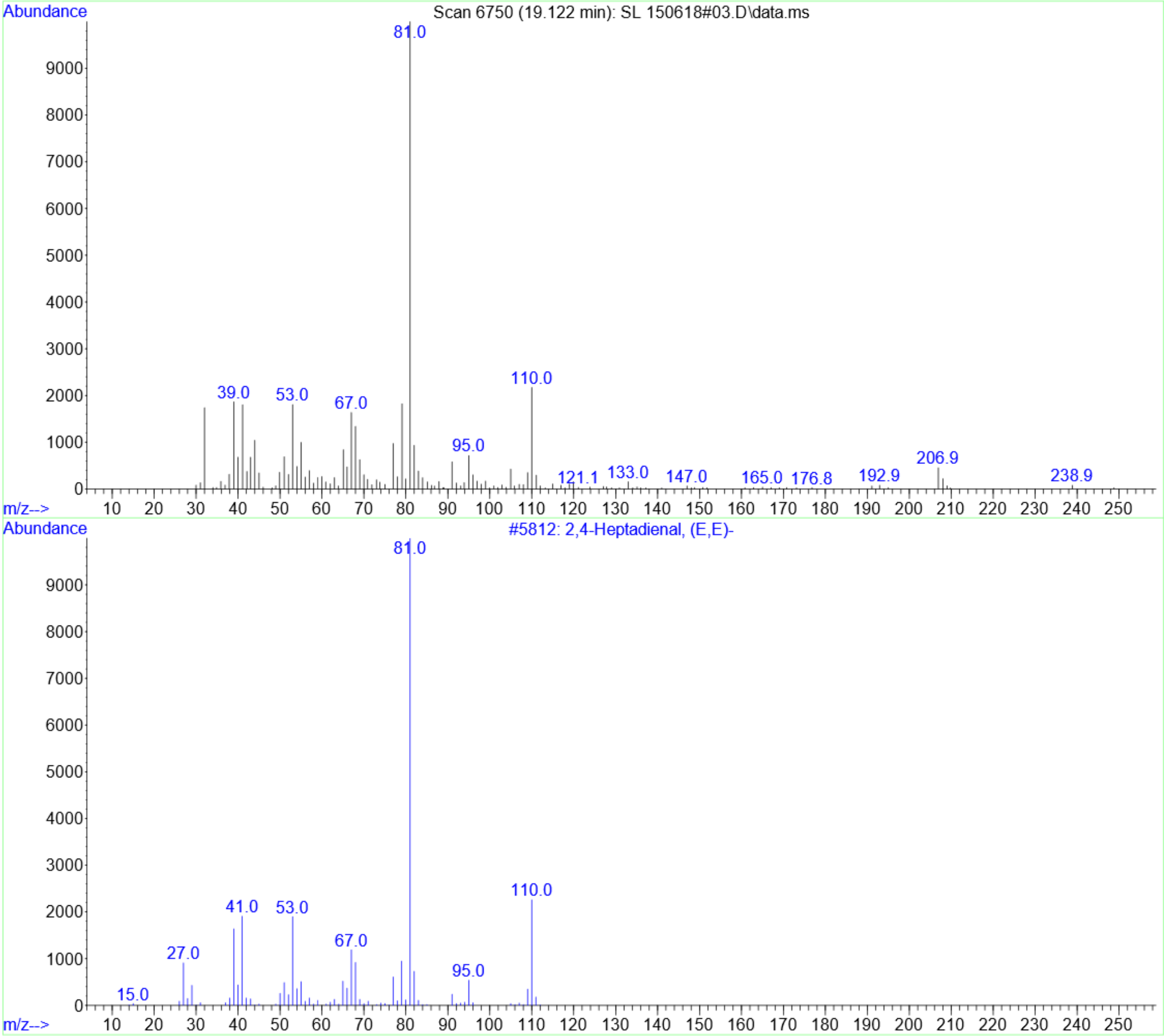

50. Octanal

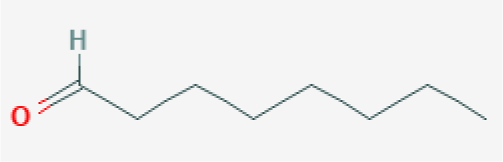

Library Searched : C:\Database\Adams.L
Quality : 90
ID : 7.72 Octanal<n->

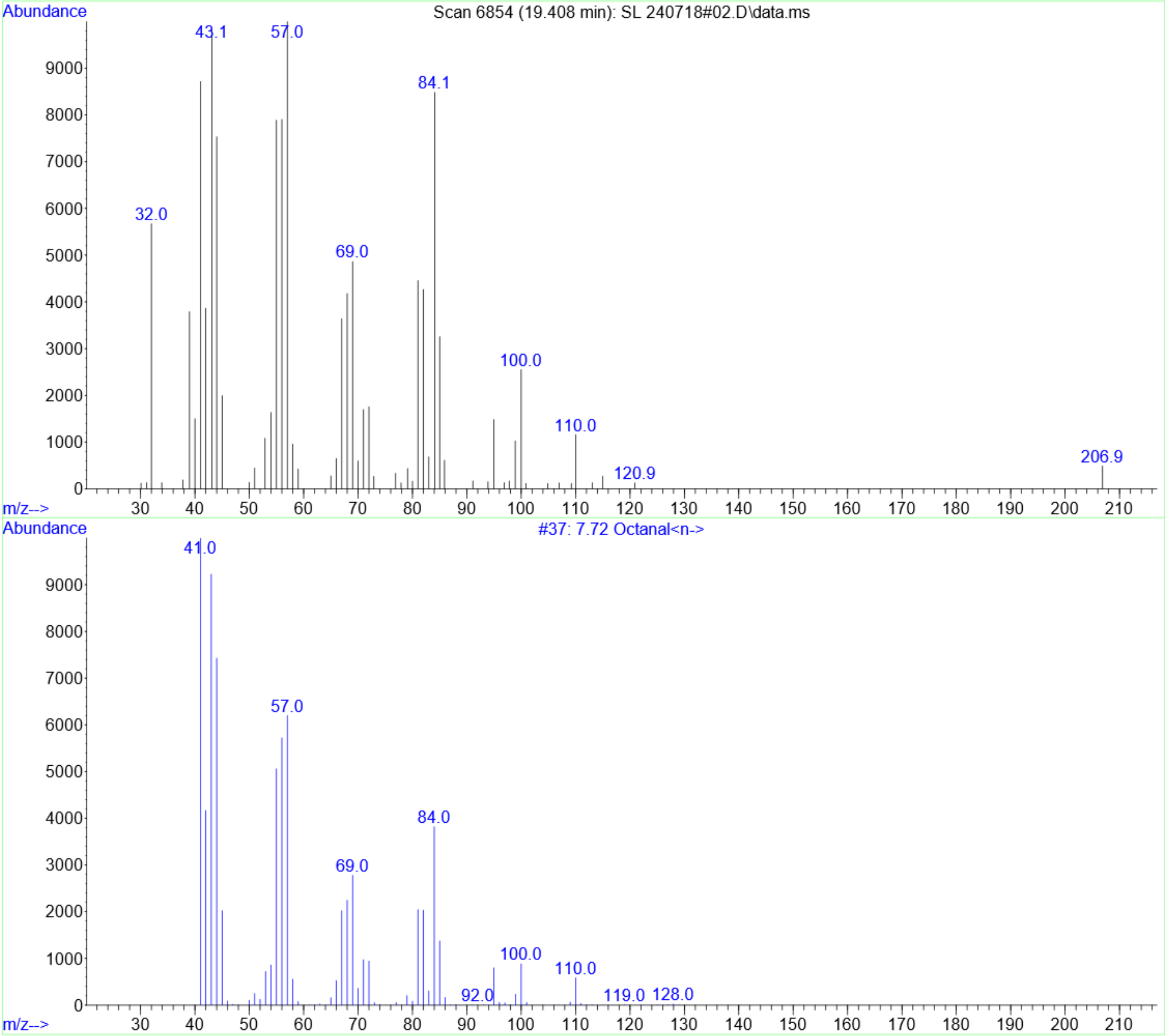

51. 2,4-heptadienal isomer 2

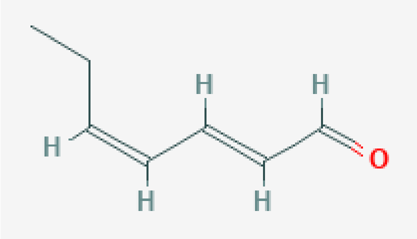

Library Searched : C:\Database\NIST11.L
Quality : 95
ID : 2,4-Heptadienal, (E,E)-

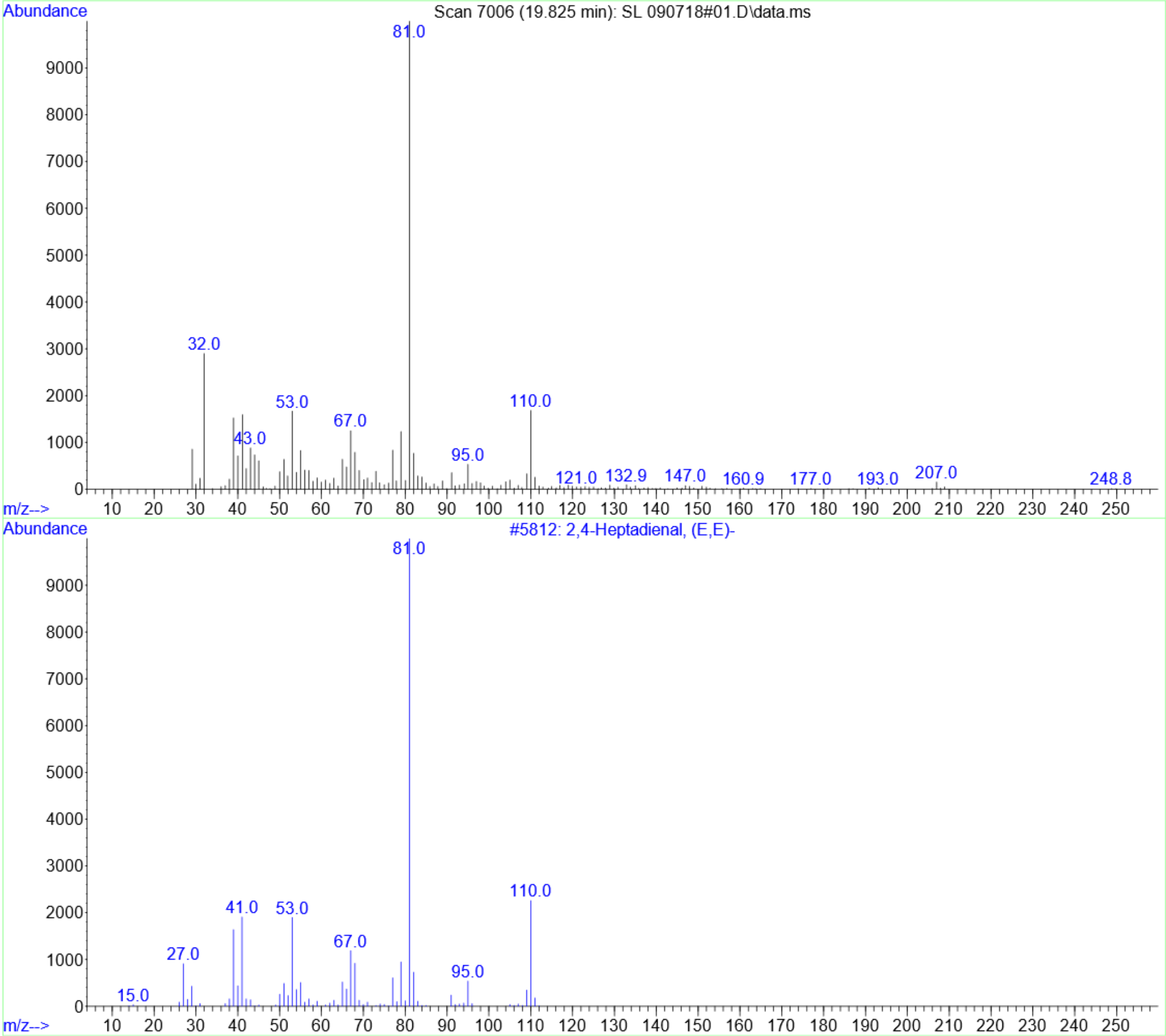

52. Phenylacetaldehyde

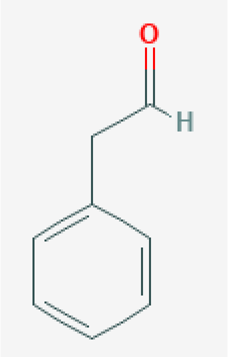

Library Searched : C:\Database\NIST11.L
Quality : 94
ID : Benzeneacetaldehyde

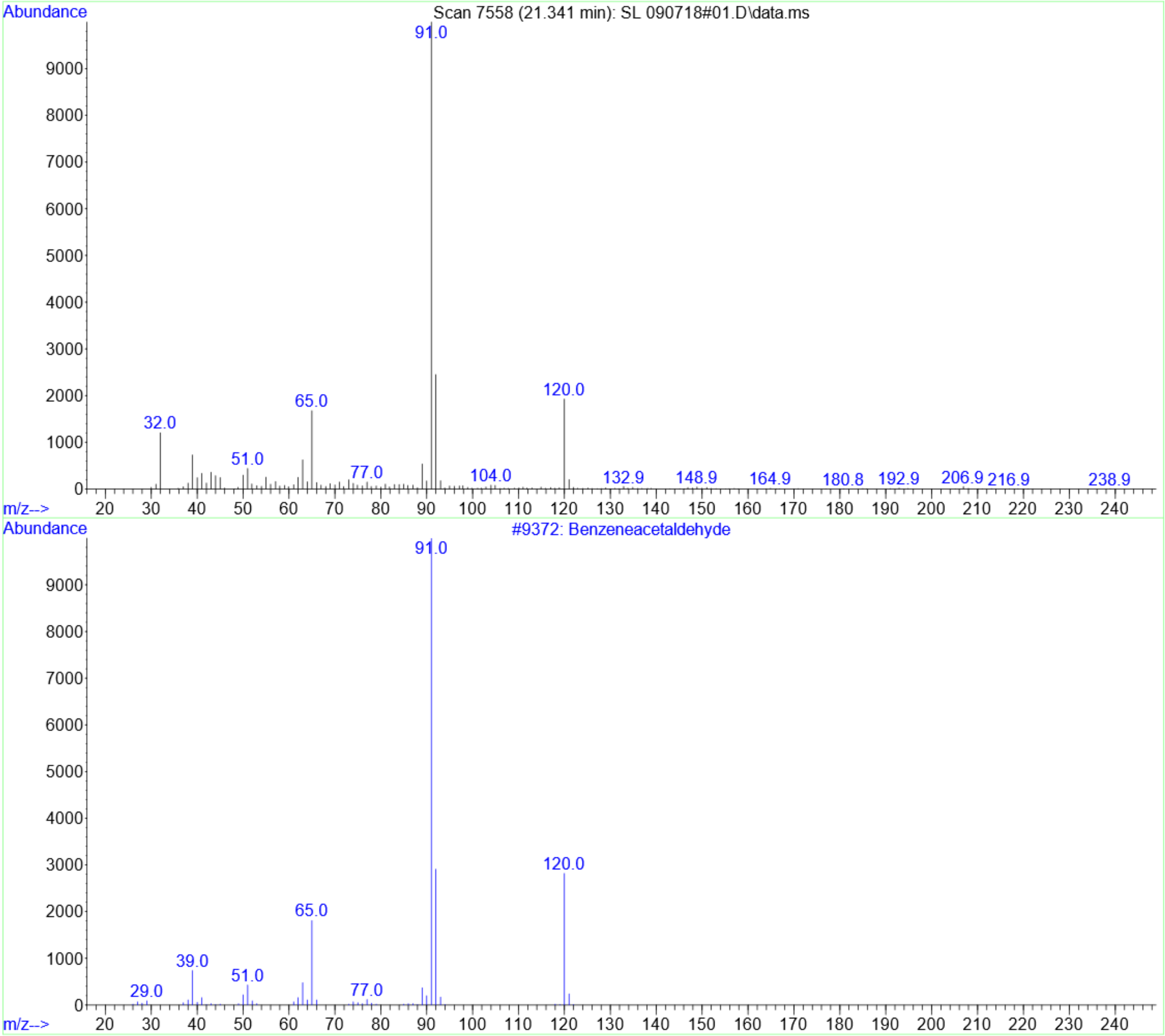

53. 2-octenal

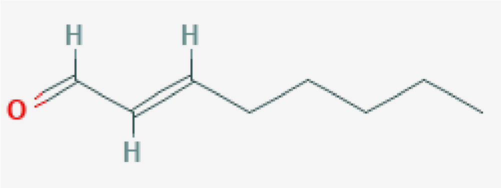

Library Searched : C:\Database\NIST11.L
Quality : 91
ID : 2-Octenal, (E)-

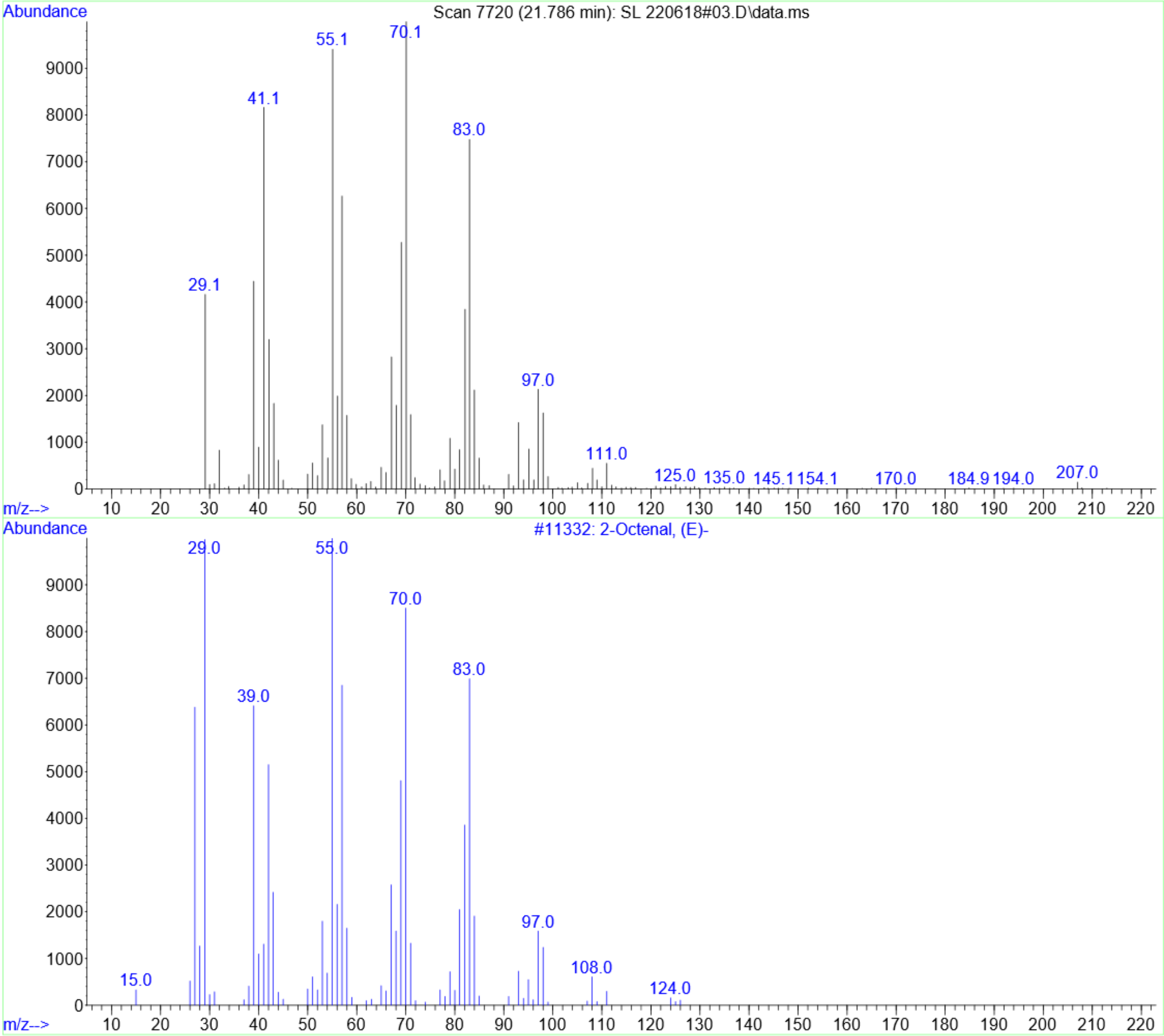

54. Nonanal

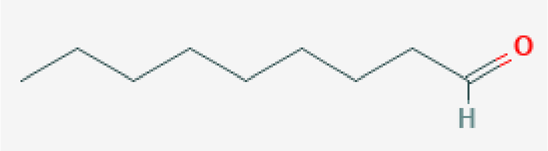

Library Searched : C:\Database\NIST11.L
Quality : 91
ID : Nonanal

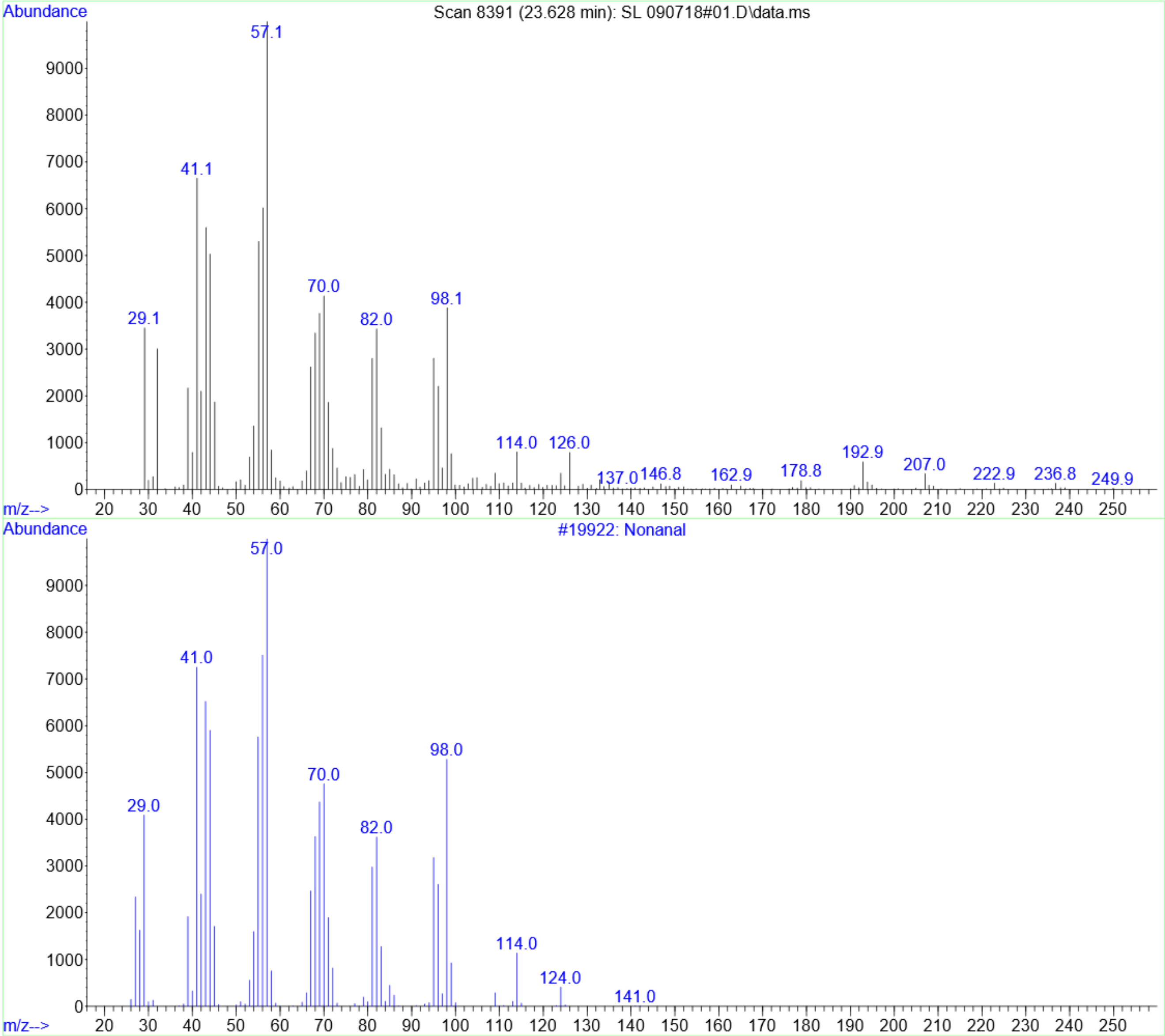

55. Decanal

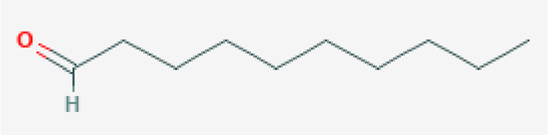

Library Searched : C:\Database\NIST11.L
Quality : 93
ID : Decanal

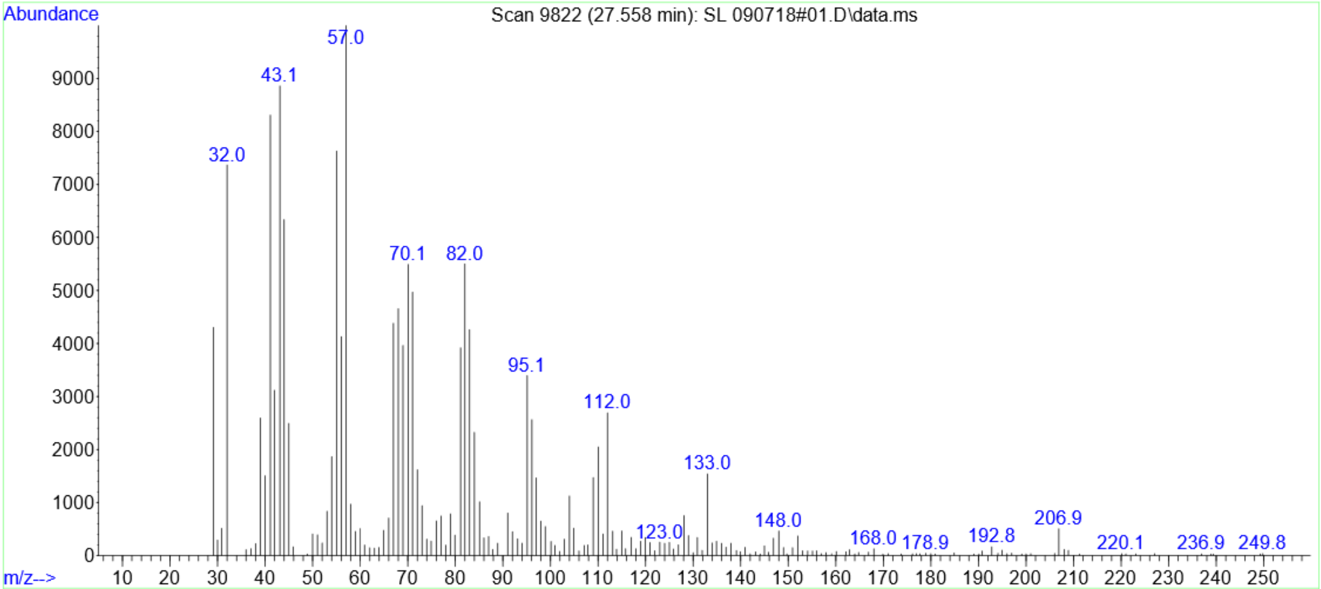

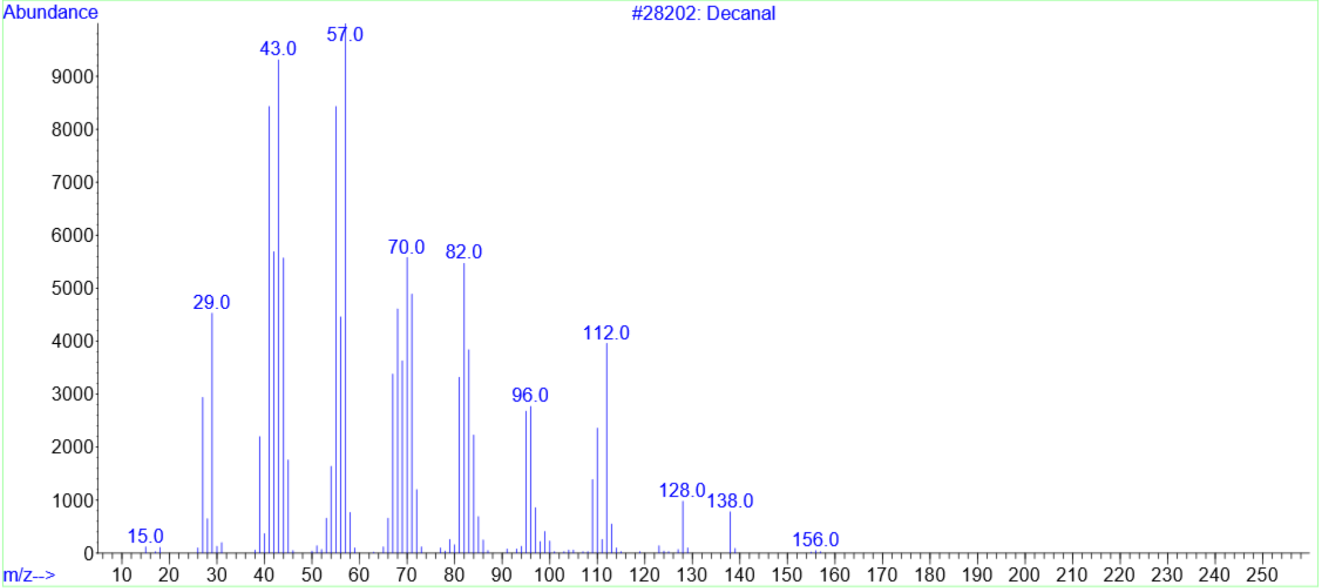

56. Vanillin

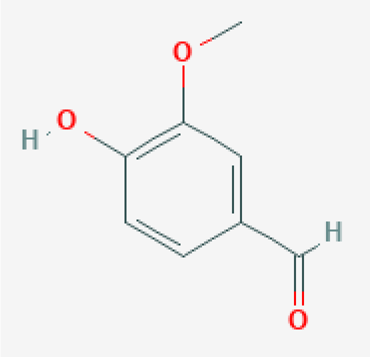

Library Searched : C:\Database\NIST11.L
Quality : 83
ID : Vanillin

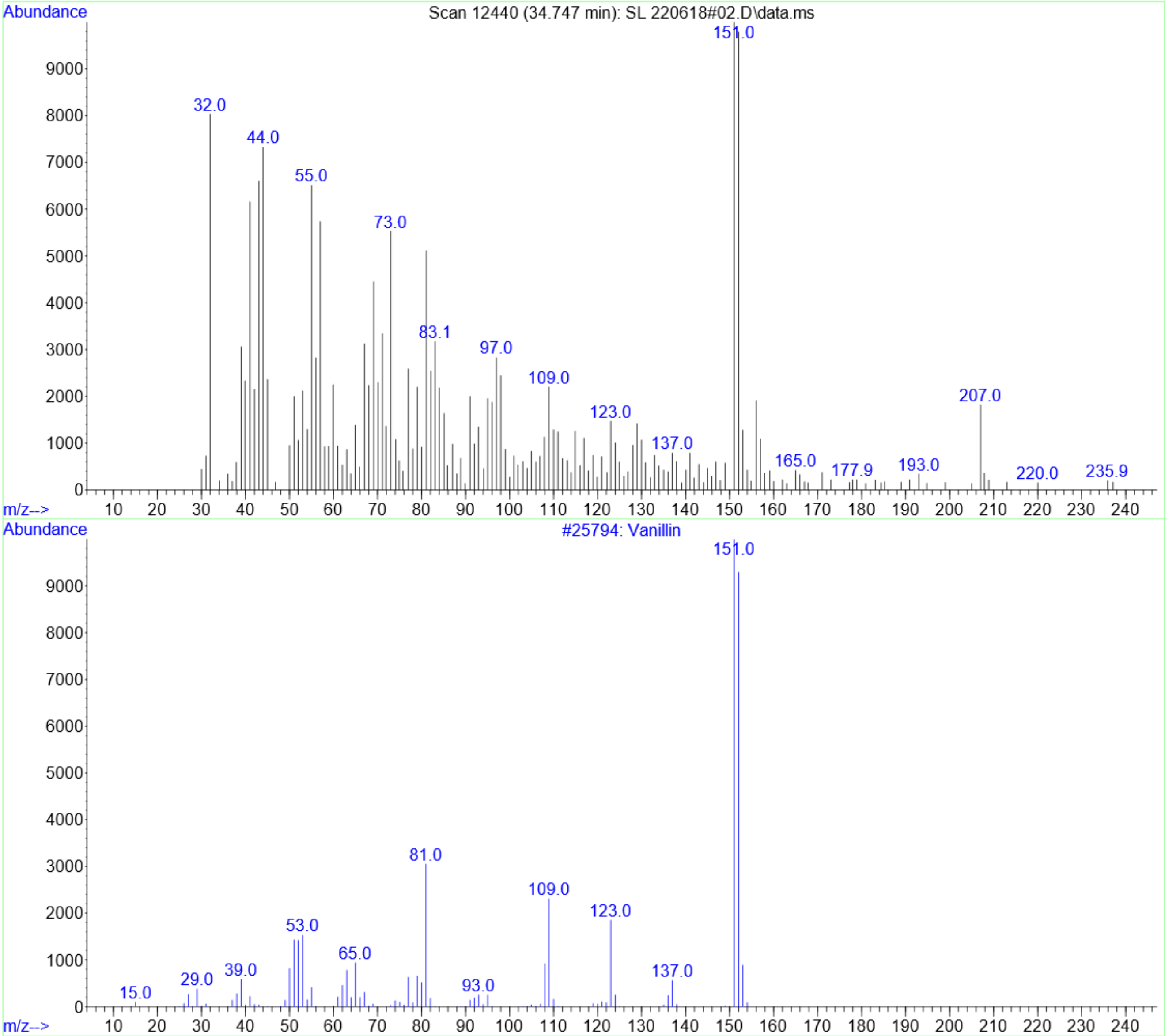

57. (*Z*)-pent-2-en-1-yl acetate

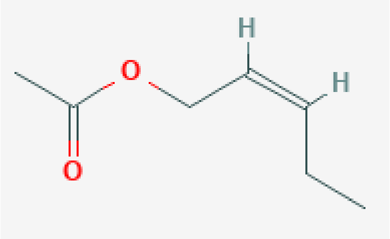

Library Searched : C:\Database\NIST11.L
Quality : 64
ID : 2-Penten-1-ol, acetate, (Z)-

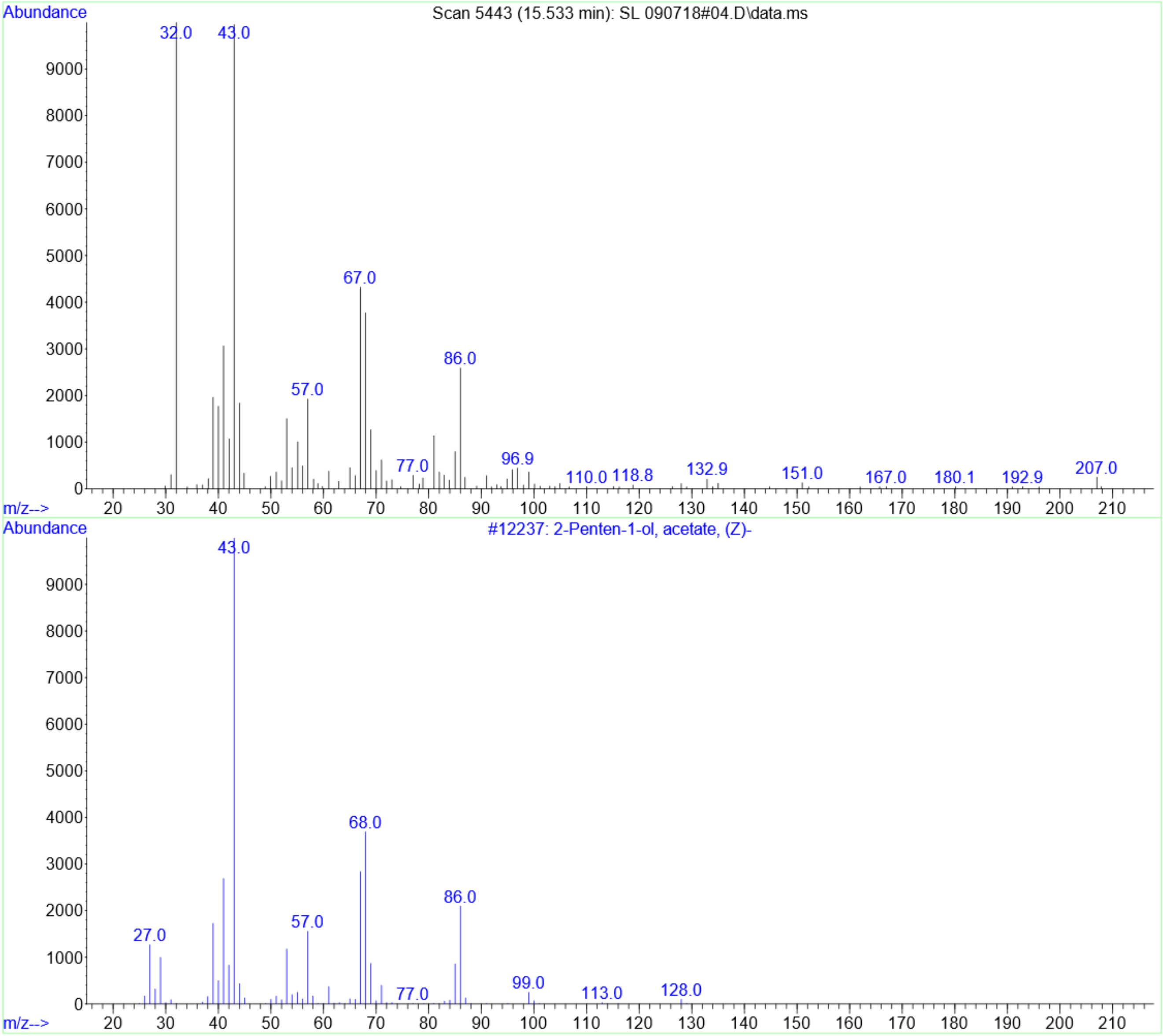

58. 3-hexenyl acetate

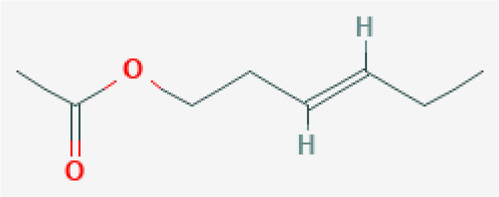

Library Searched : C:\Database\NIST11.L
Quality : 90
ID : 3-Hexen-1-ol, acetate, (Z)-

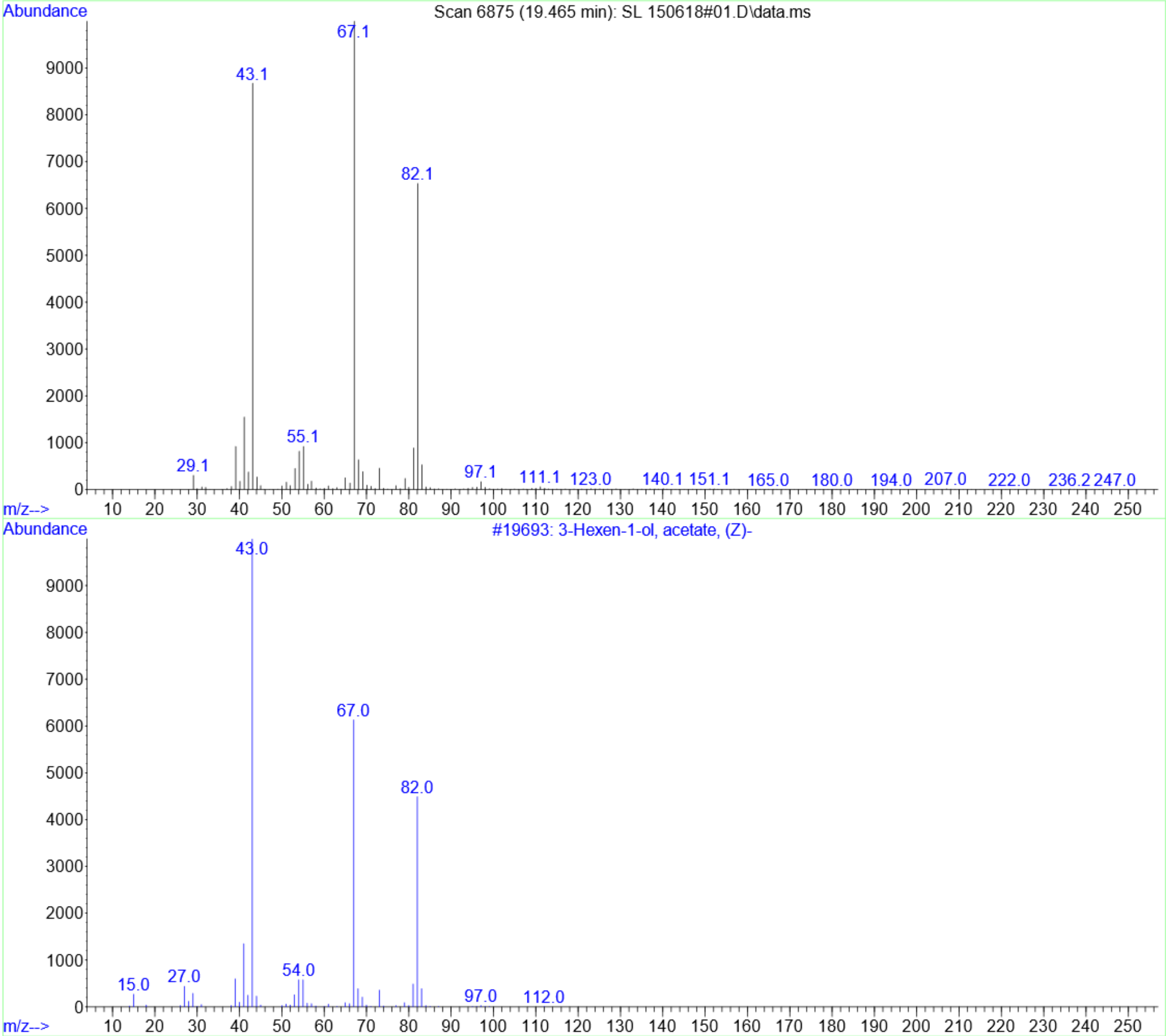

59. (*Z*)-3-hexenyl butanoate

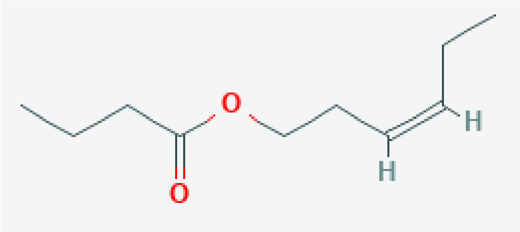

Library Searched : C:\Database\NIST11.L
Quality : 90
ID : Butanoic acid, 3-hexenyl ester, (Z)-

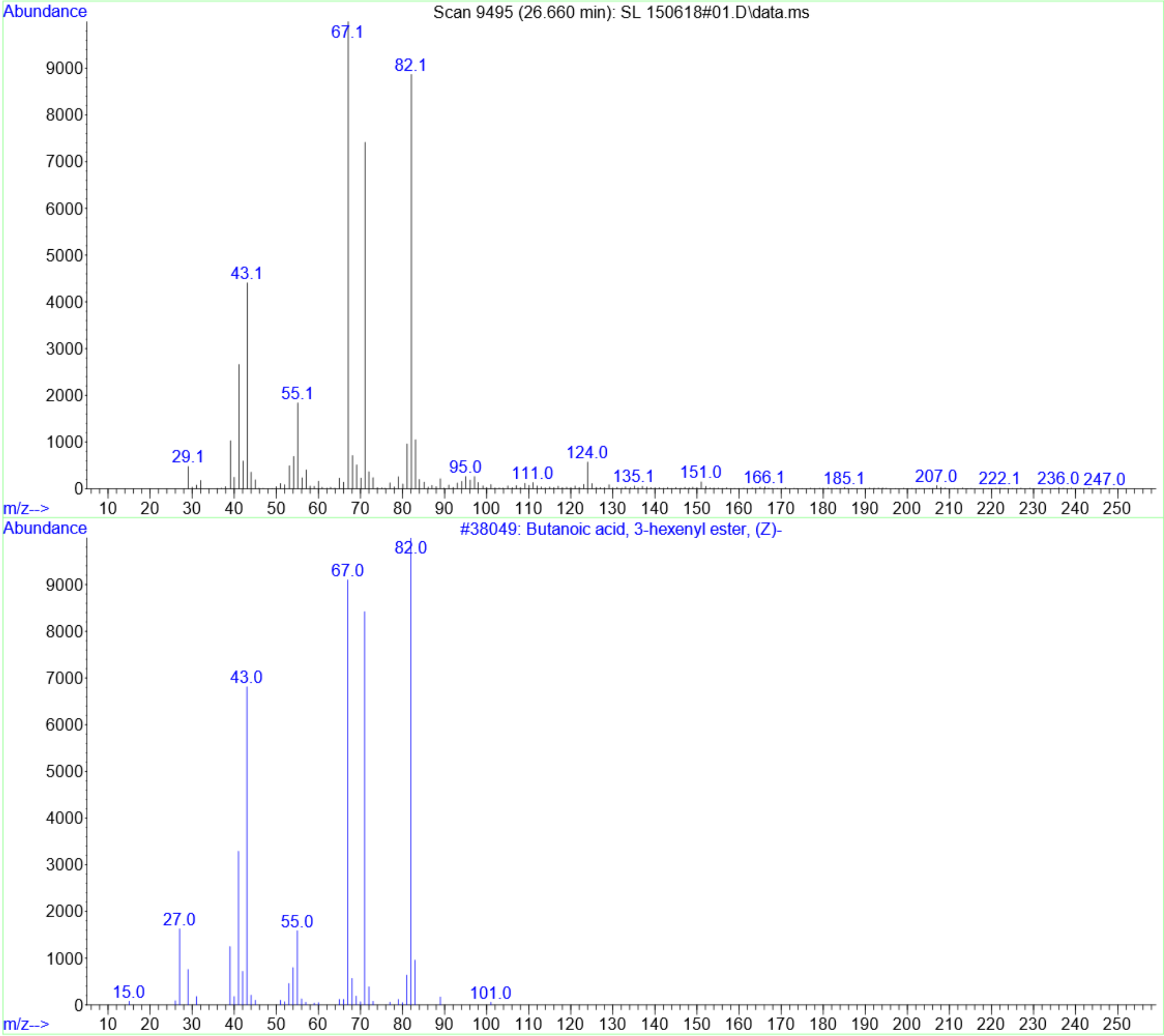

60. Methyl salicylate

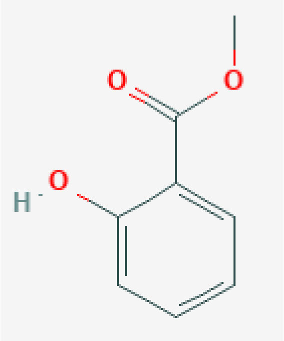

Library Searched : C:\Database\Adams.L
Quality : 89
ID : 15.35 Methyl salicylate

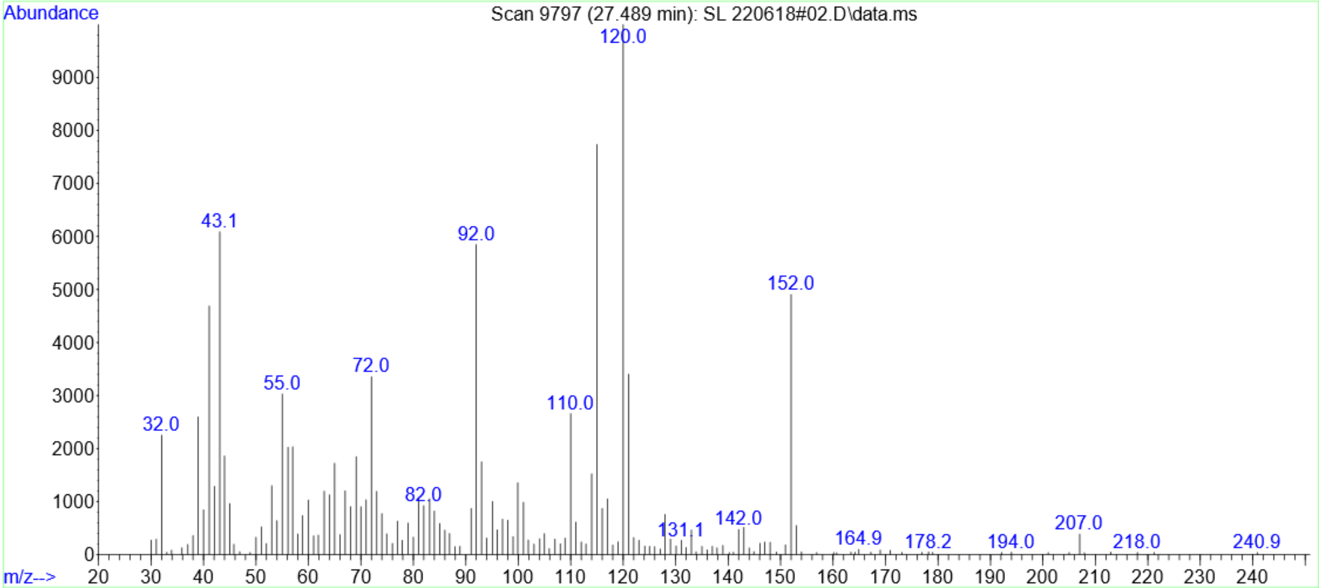

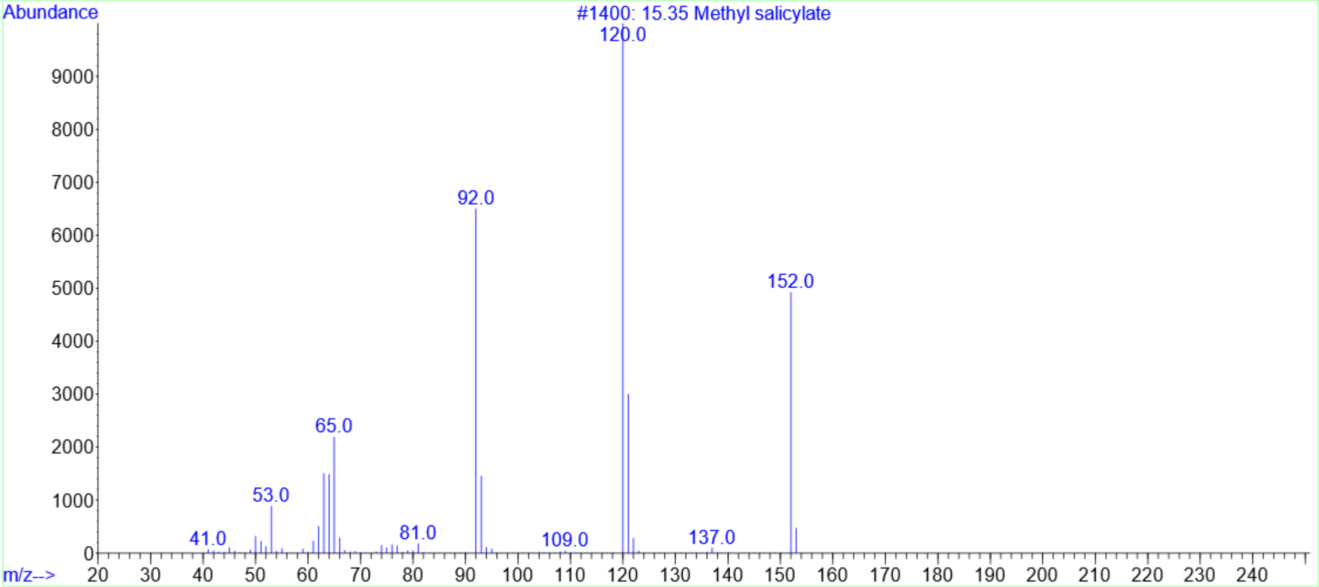

61. Ethyl decanoate

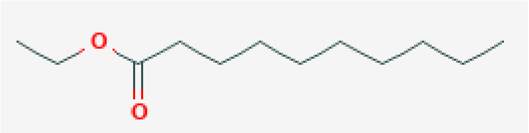

Library Searched : C:\Database\NIST11.L
Quality : 90
ID : Decanoic acid, ethyl ester

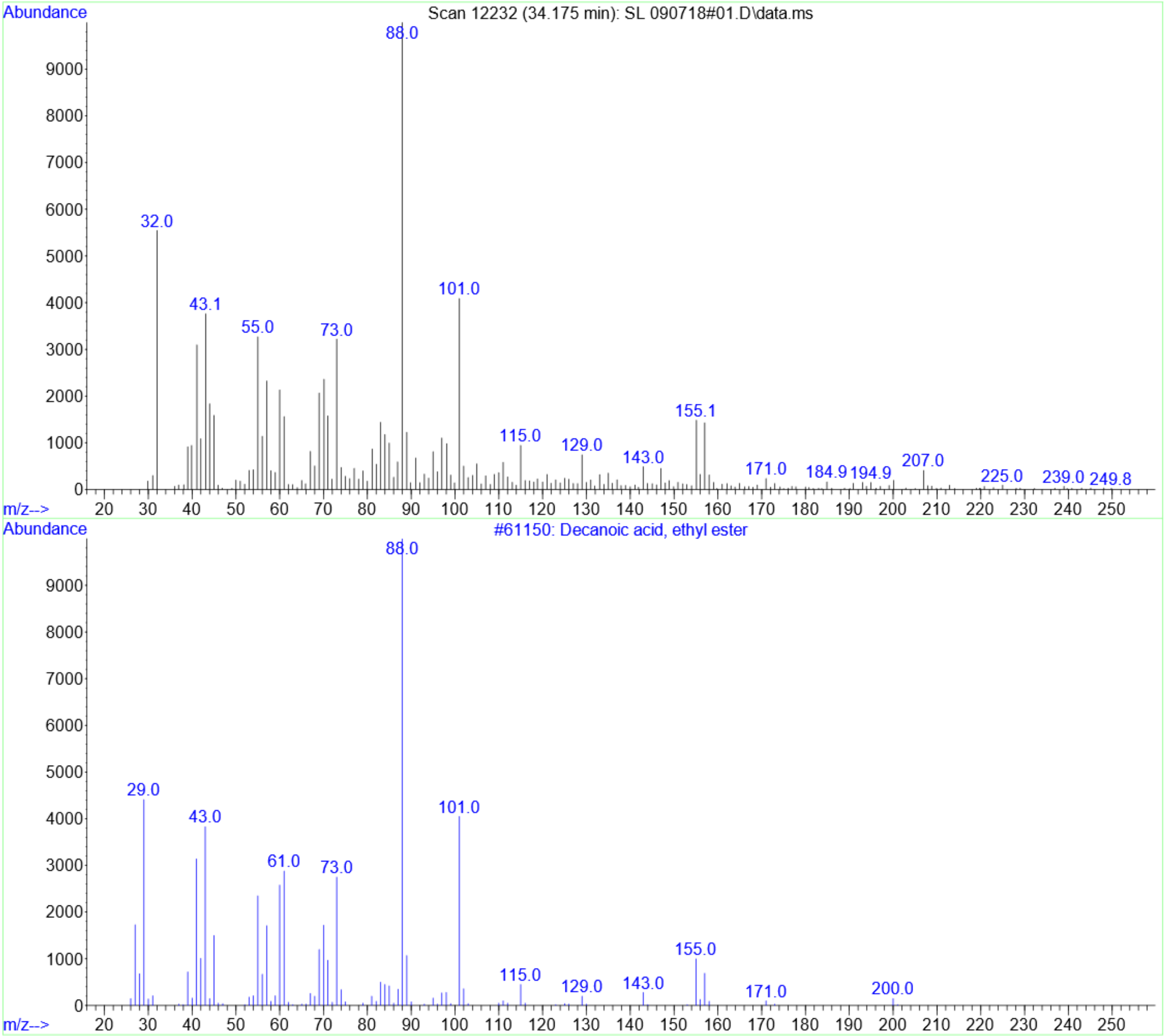

62. Methyl dodecanoate

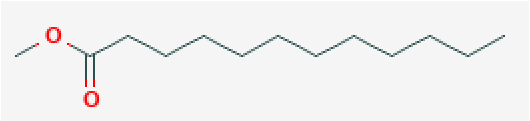

Library Searched : C:\Database\Adams.L
Quality : 93
ID : 29.82 Methyl dodecanoate

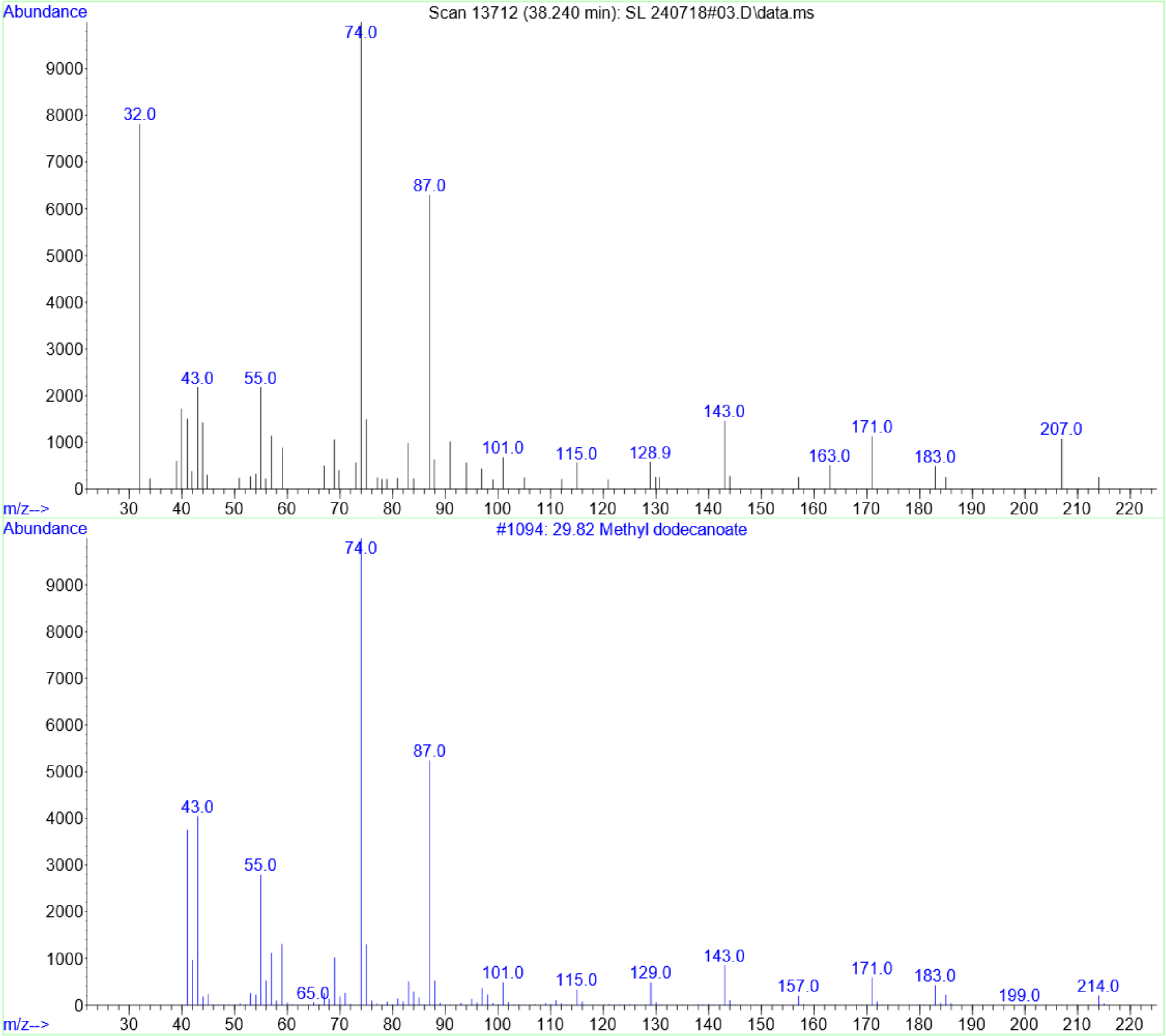

63. Diethyl phthalate

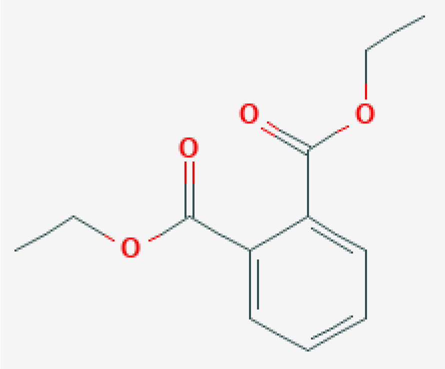

Library Searched : C:\Database\NIST11.L
Quality : 90
ID : Diethyl Phthalate

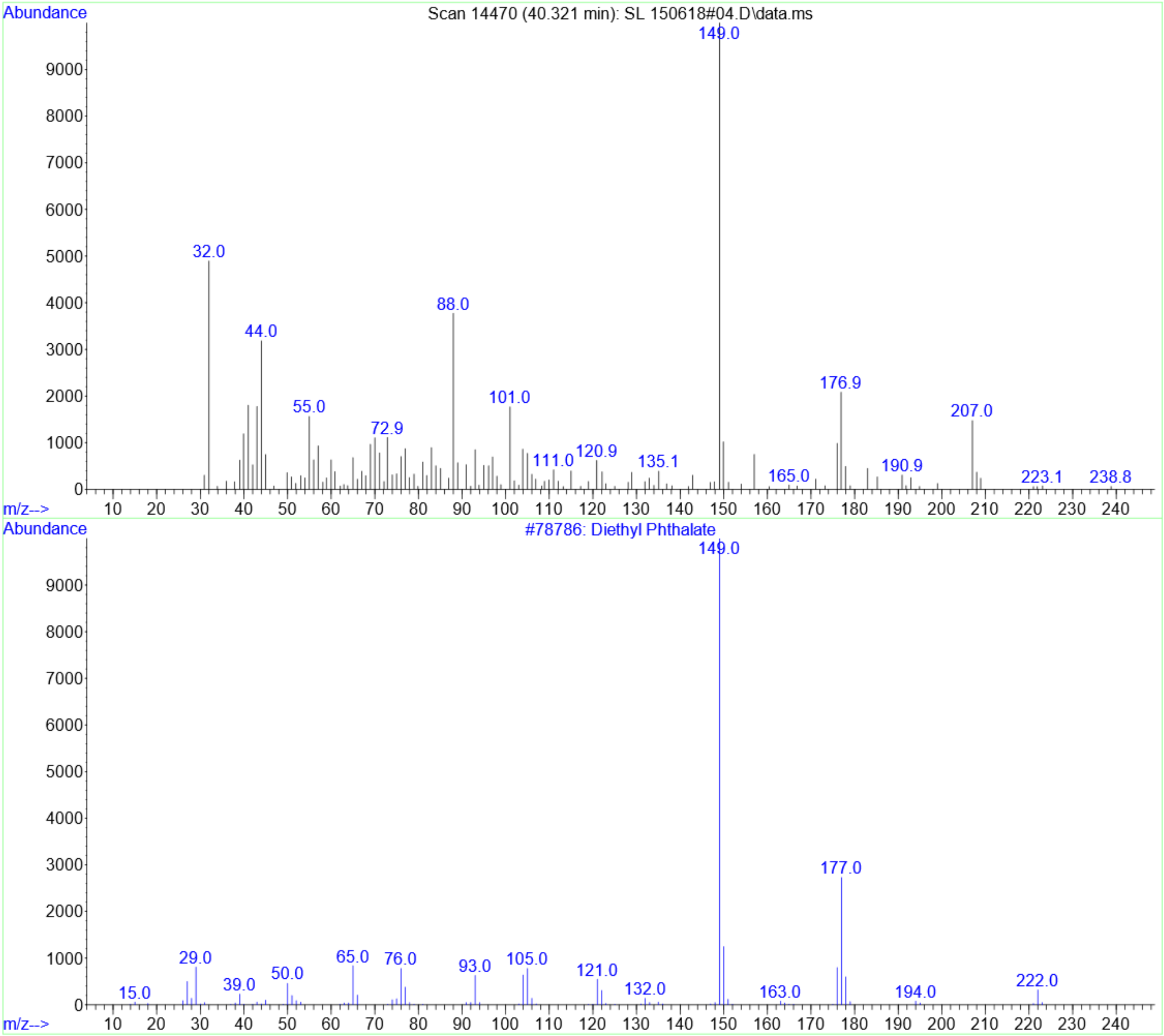

64. Ethyl laurate

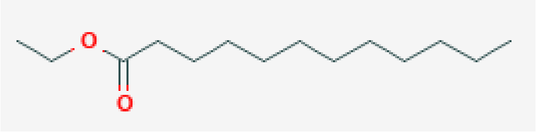

Library Searched : C:\Database\NIST11.L
Quality : 68
ID : Dodecanoic acid, ethyl ester

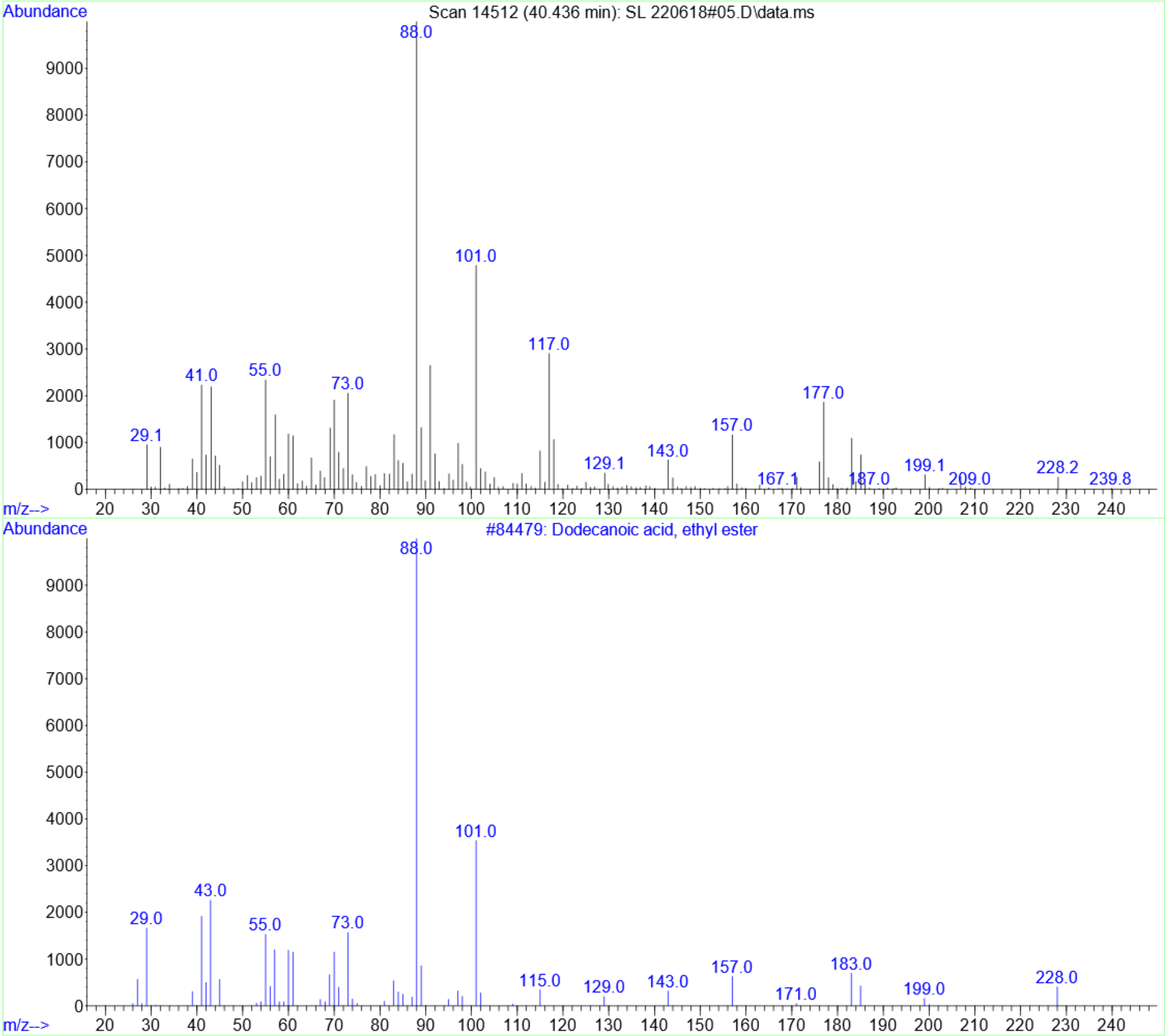

65. Ethyl vinyl ketone

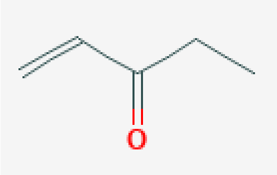

Library Searched : C:\Database\NIST11.L
Quality : 52
ID : 1-Penten-3-one

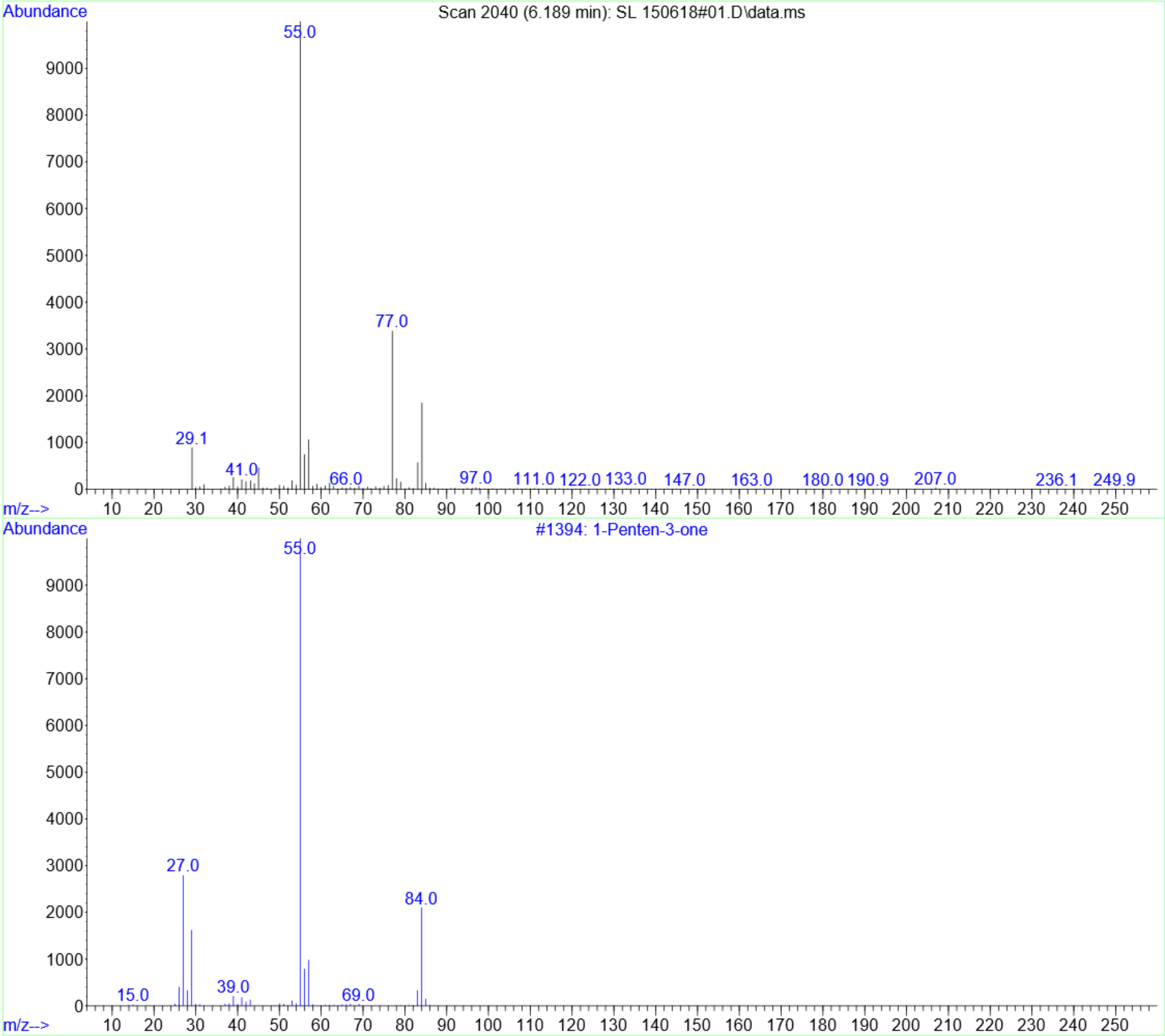

66. 3-pentanone

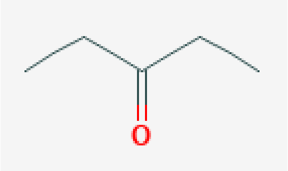

Library Searched : C:\Database\NIST11.L
Quality : 78
ID : 3-Pentanone

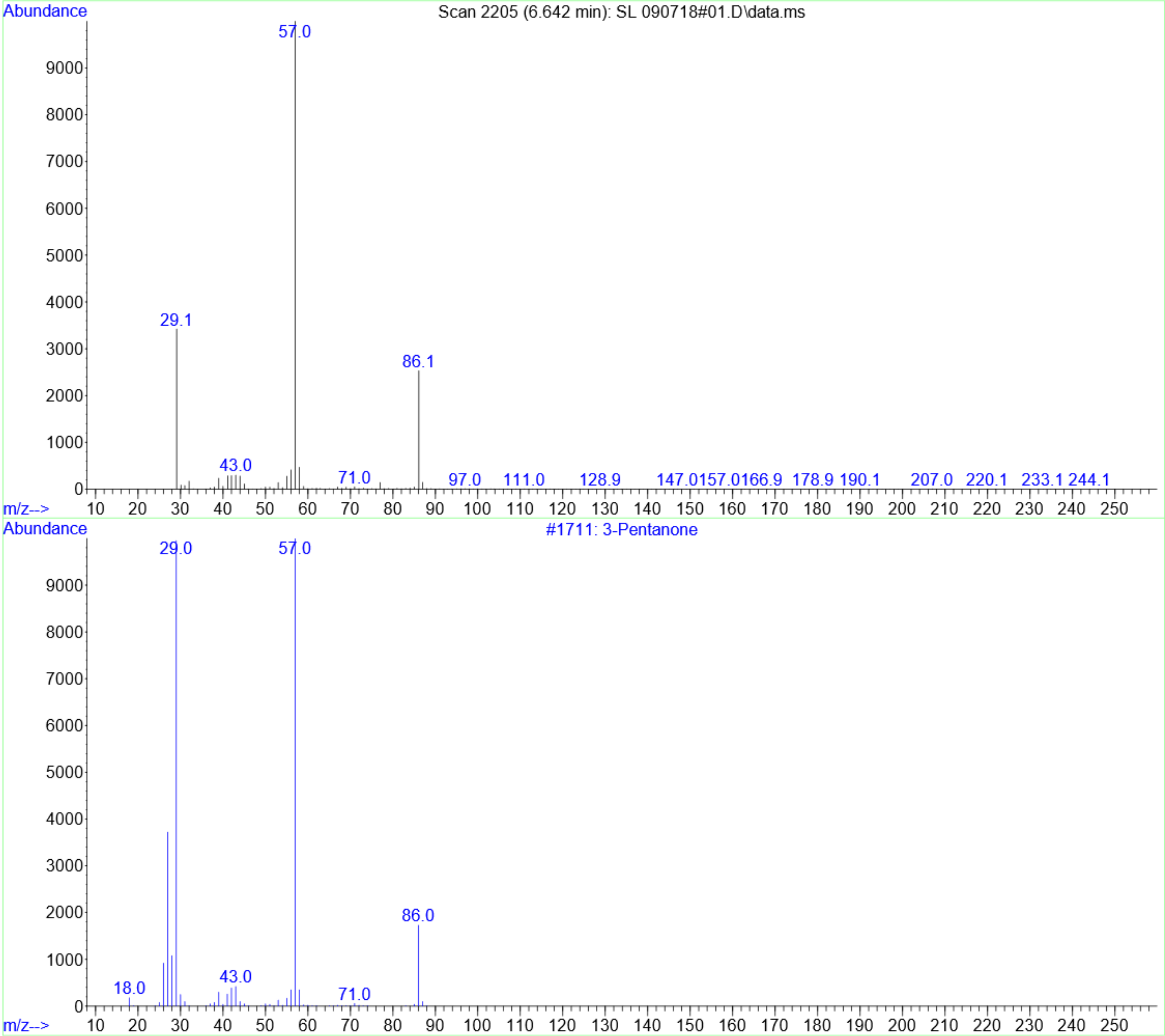

67. 2,3-octanedione

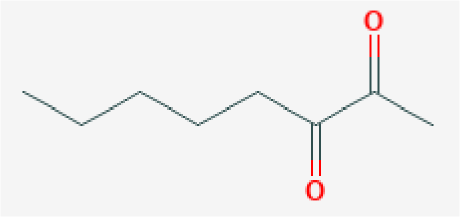

Library Searched : C:\Database\NIST11.L
Quality : 64
ID : 3-Octanone, 2-methyl-

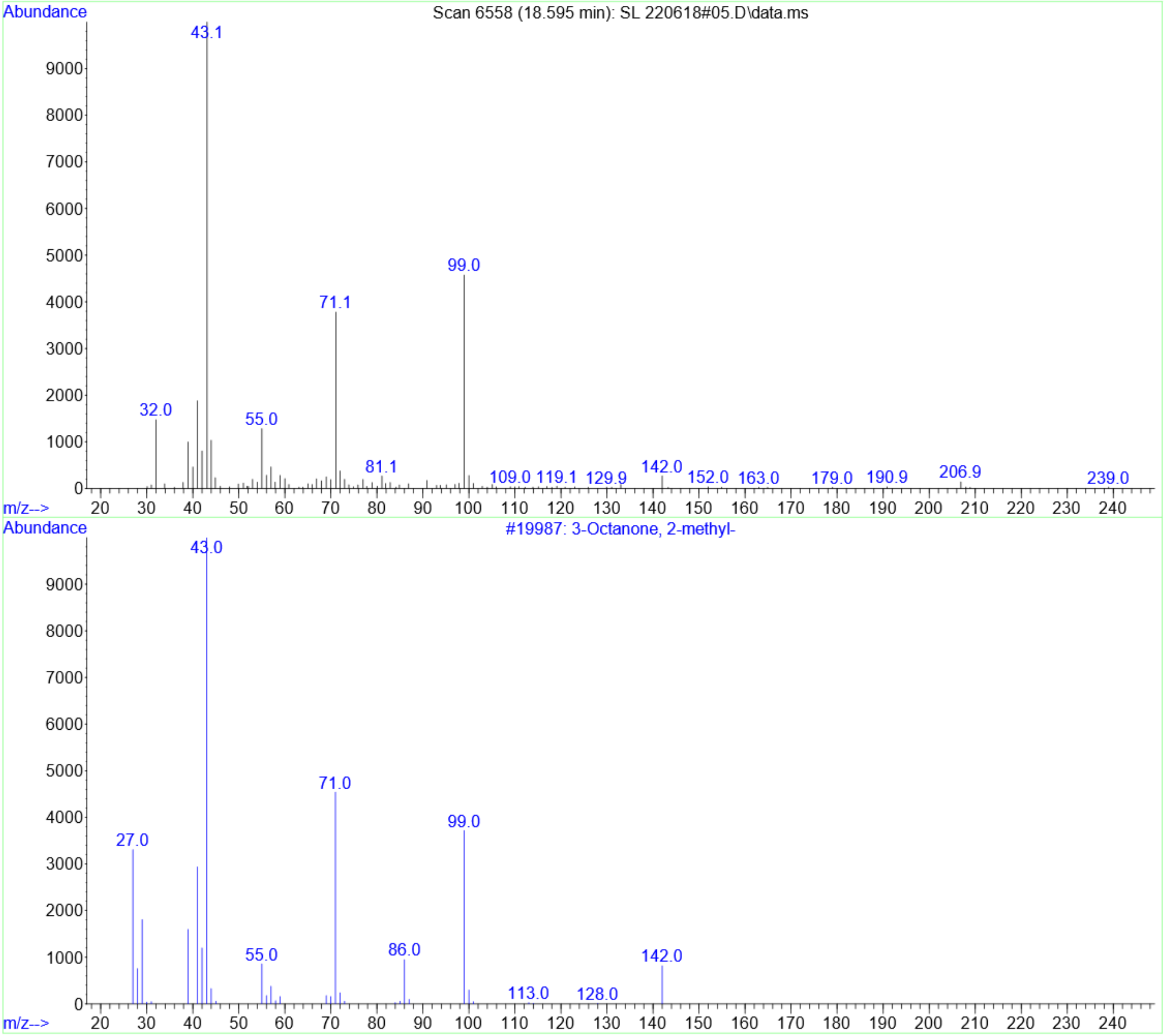

68. 6-methyl-5-hepten-2-one

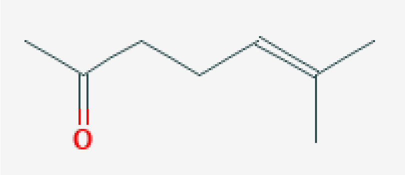

Library Searched : C:\Database\NIST11.L
Quality : 93
ID : 5-Hepten-2-one, 6-methyl-

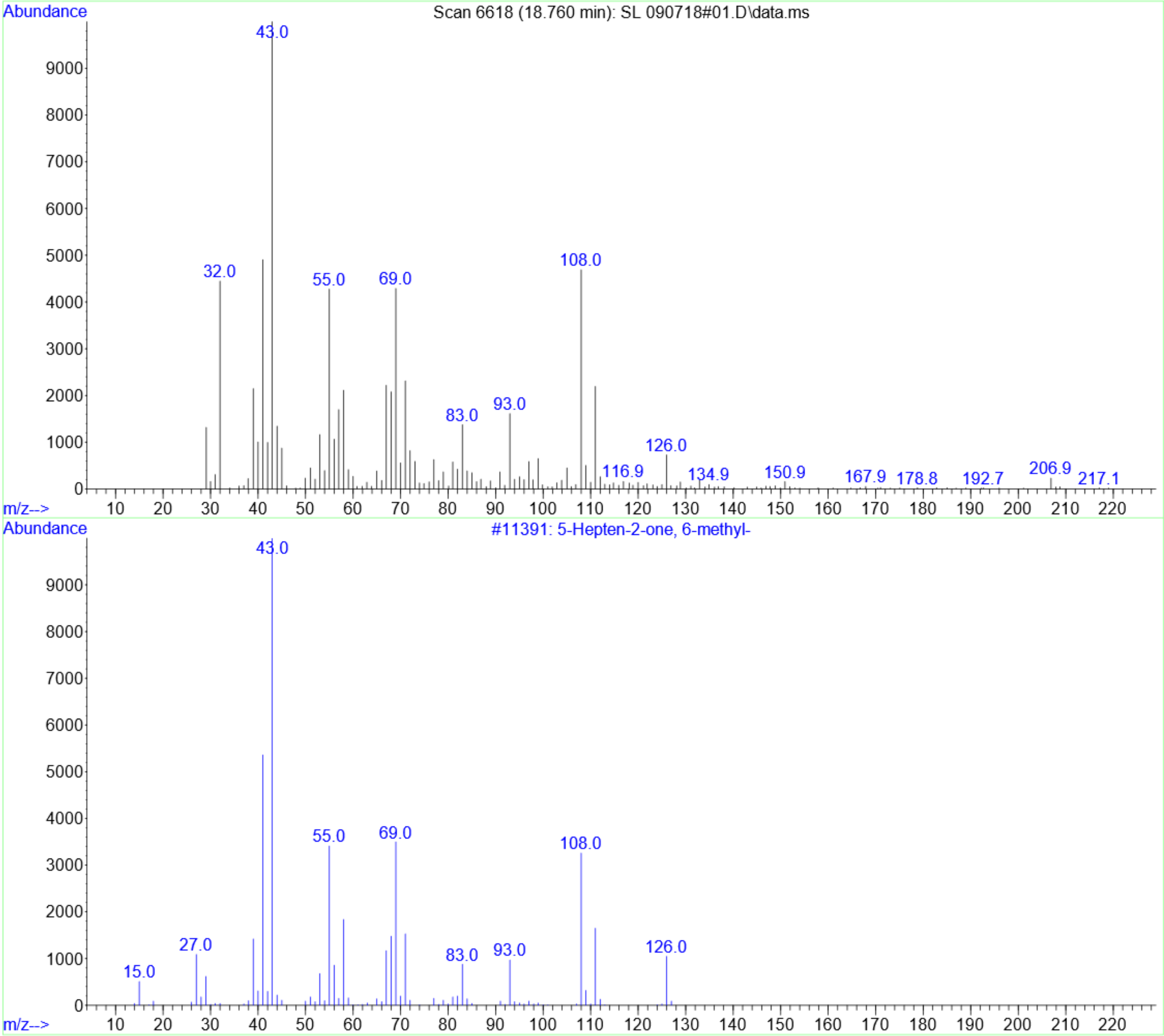

69. 2,2,6-trimethylcyclohexanone

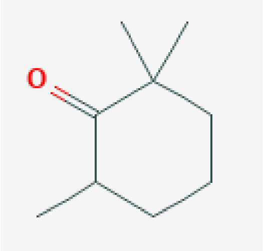

Library Searched : C:\Database\NIST11.L
Quality : 58
ID : Cyclohexanone, 2,2,6-trimethyl-

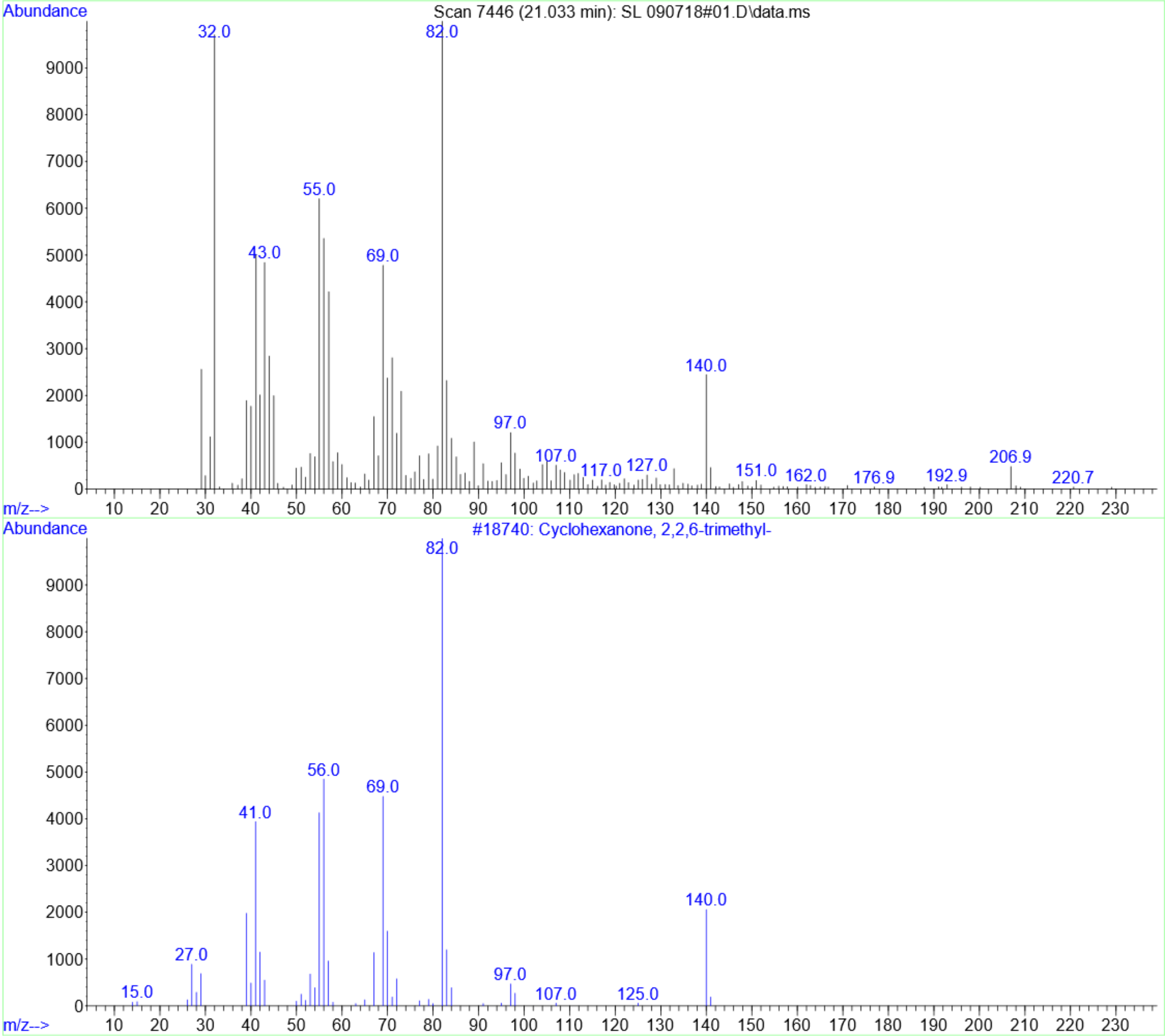

70. (*E*,*E*)-3,5-octadien-2-one

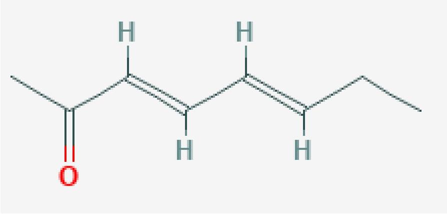

Library Searched : C:\Database\NIST11.L
Quality : 87
ID : 3,5-Octadien-2-one

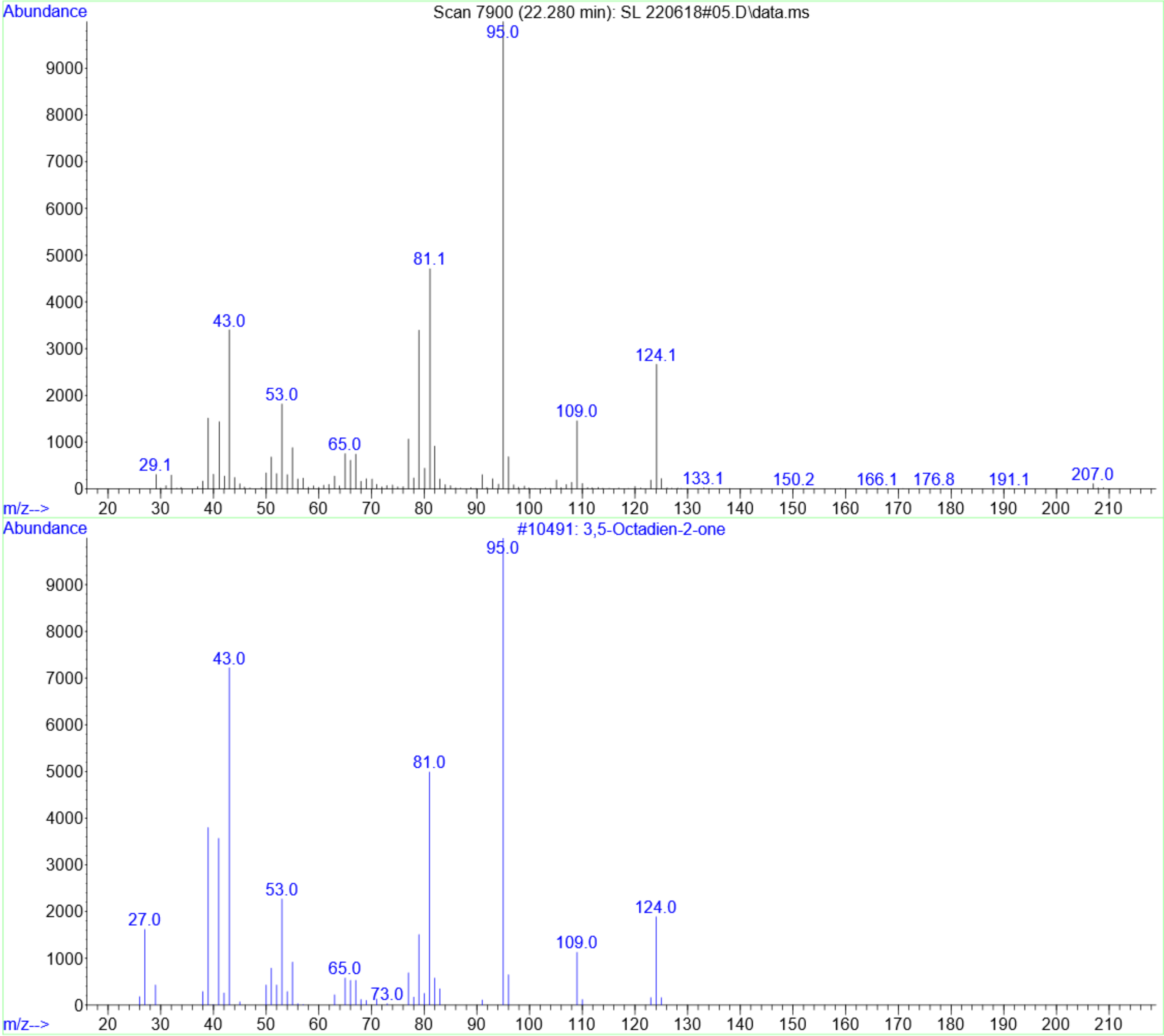

71. 3,5-octadien-2-one

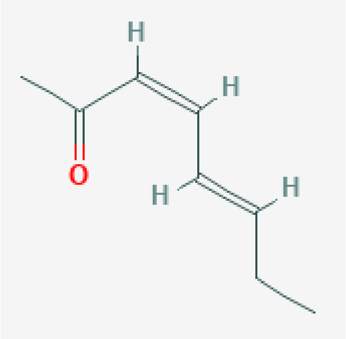

Library Searched : C:\Database\NIST11.L
Quality : 90
ID : 3,5-Octadien-2-one

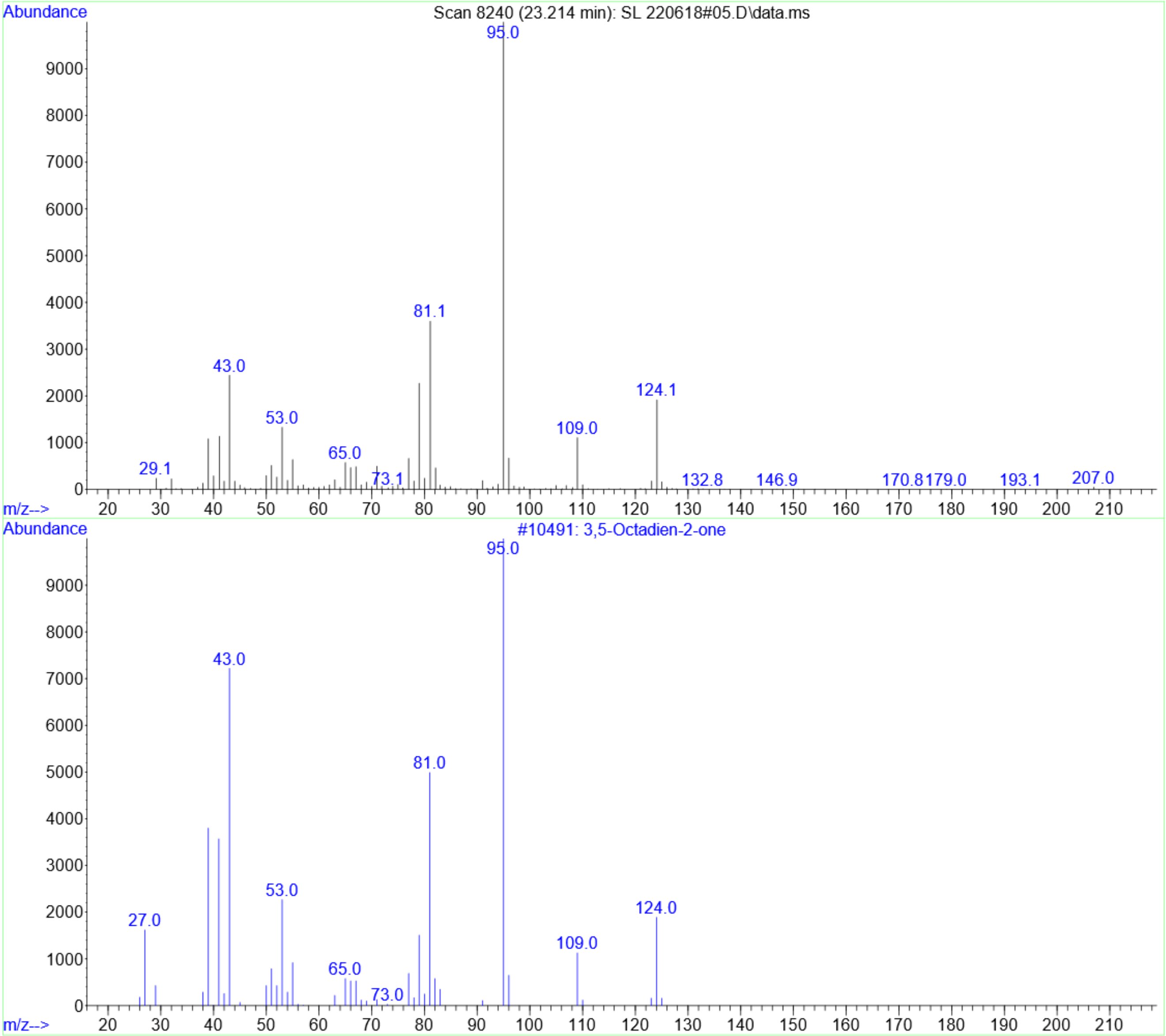

72. Dihydro-2H-thiopyran-3(4H)-one

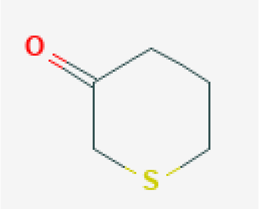

Library Searched : C:\Database\NIST11.L
Quality : 53
ID : 2H-Thiopyran-3(4H)-one, dihydro-

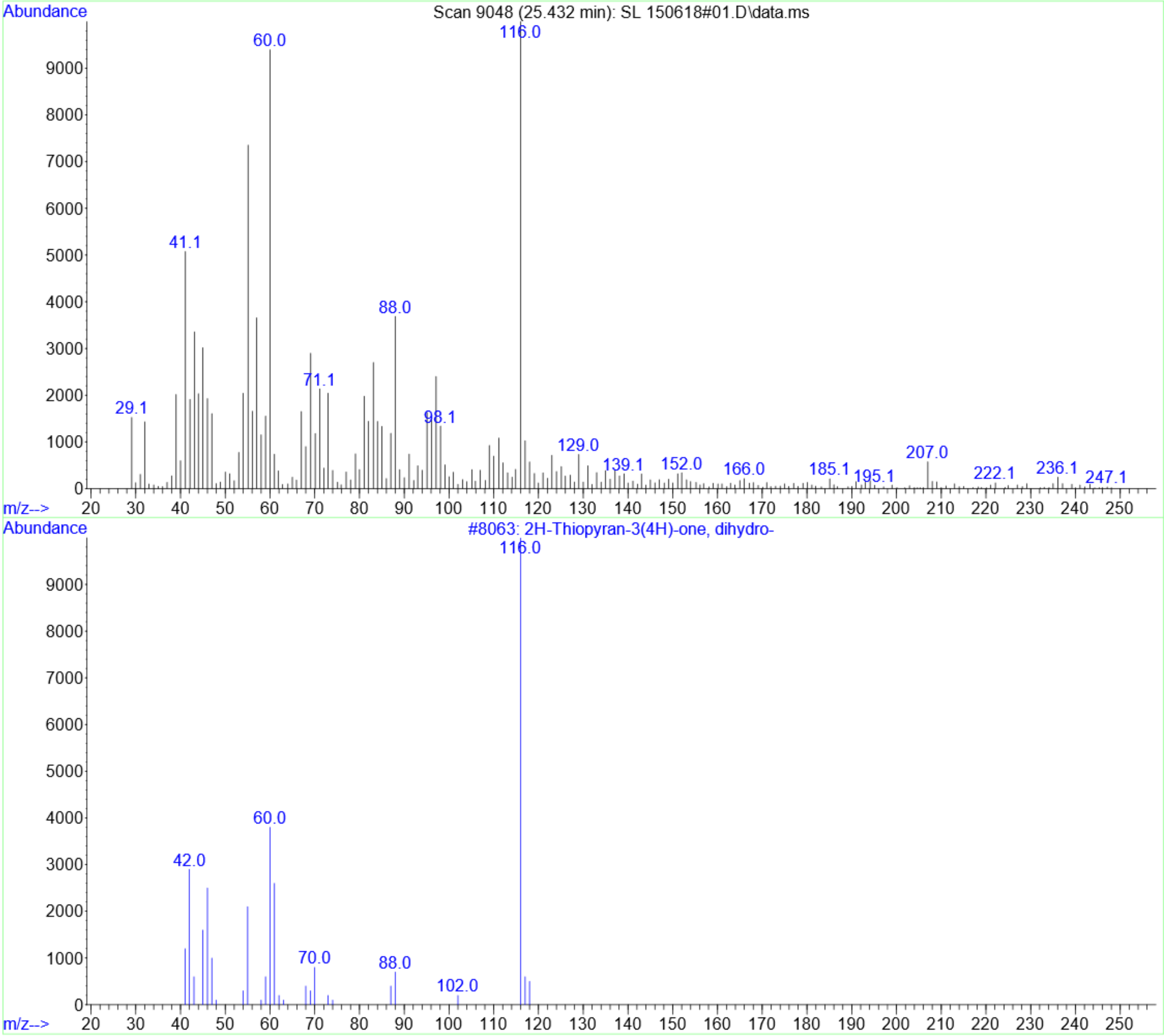

73. Geranylacetone

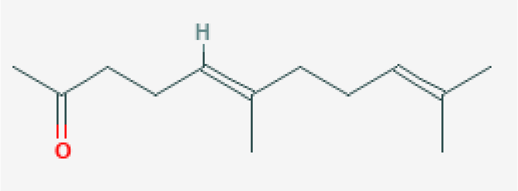

Library Searched : C:\Database\Adams.L
Quality : 78
ID : 26.85 Geranyl acetone

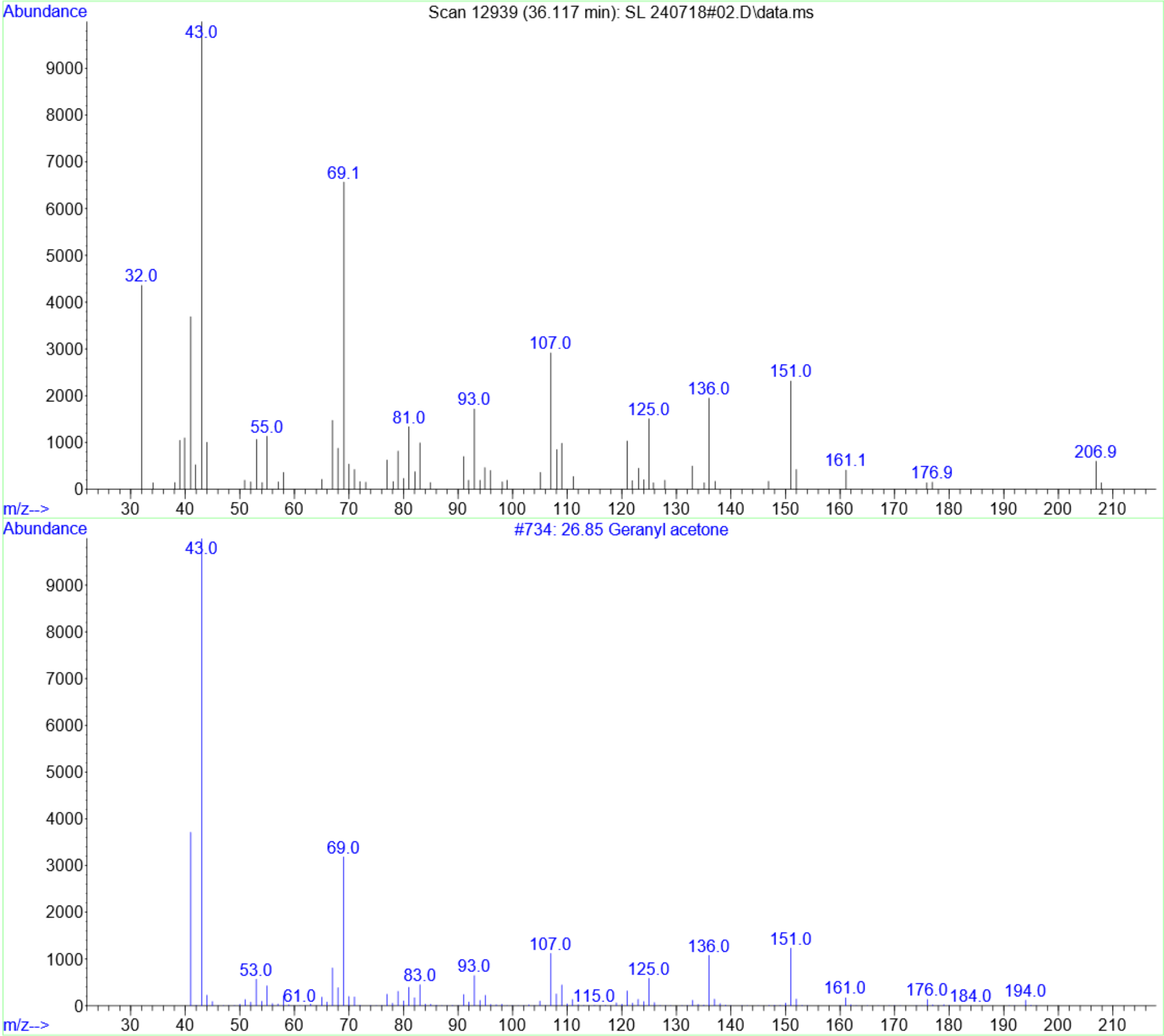

74. 3-butenenitrile

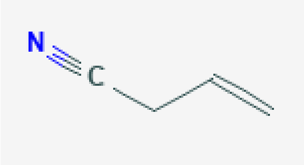

Library Searched : C:\Database\NIST11.L
Quality : 94
ID : 3-Butenenitrile

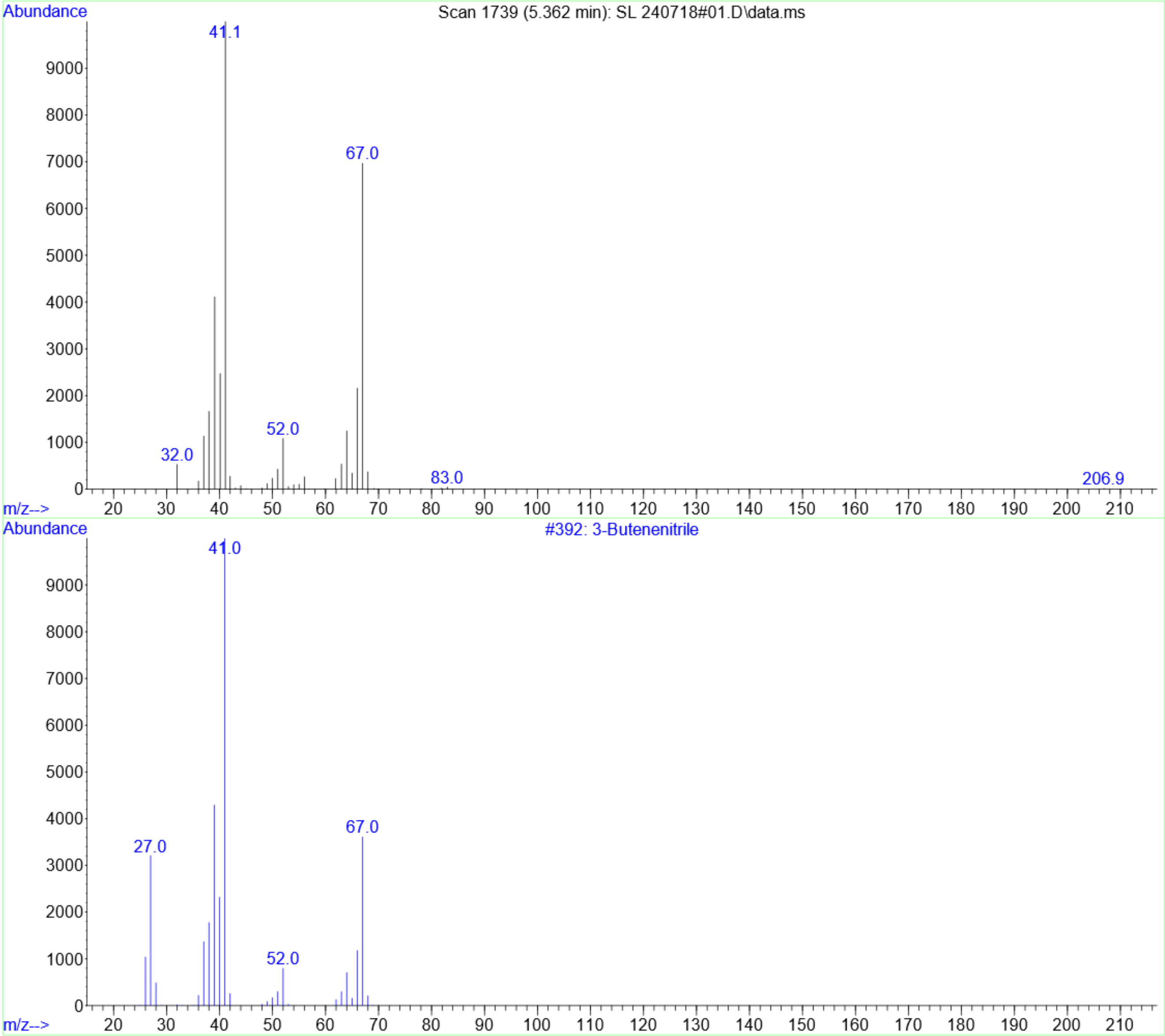

75. 5-methylhexanenitrile

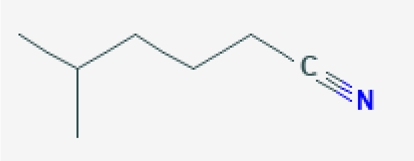

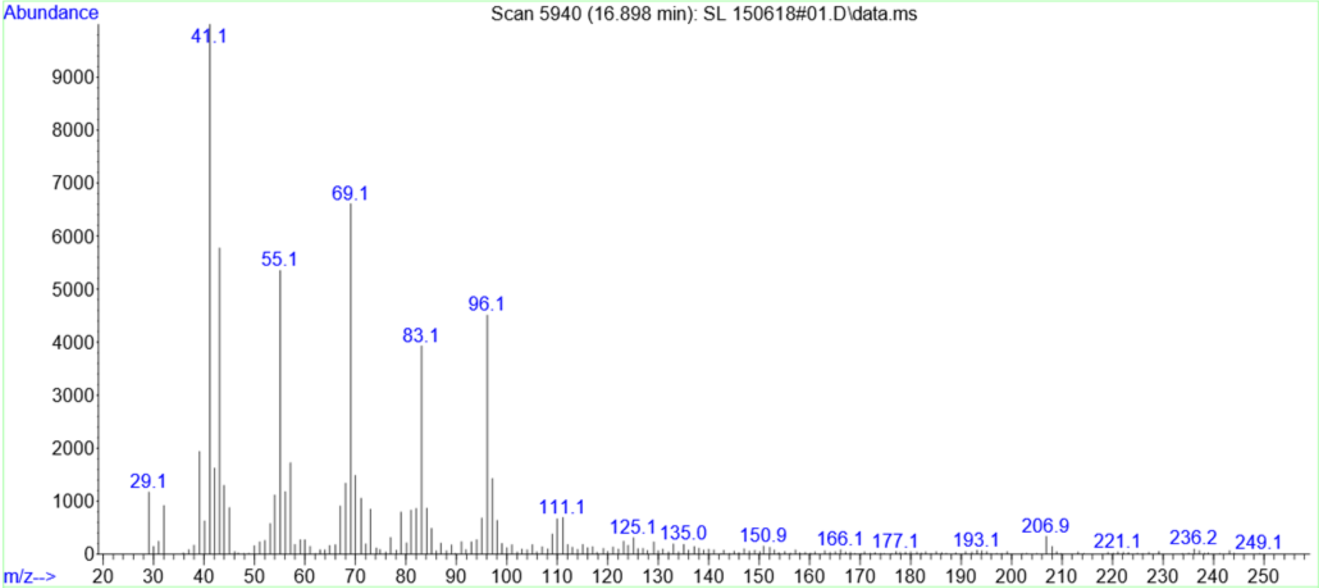

76. 6-heptenenitrile

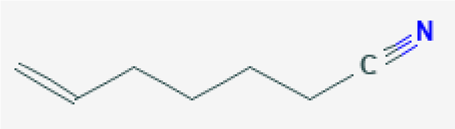

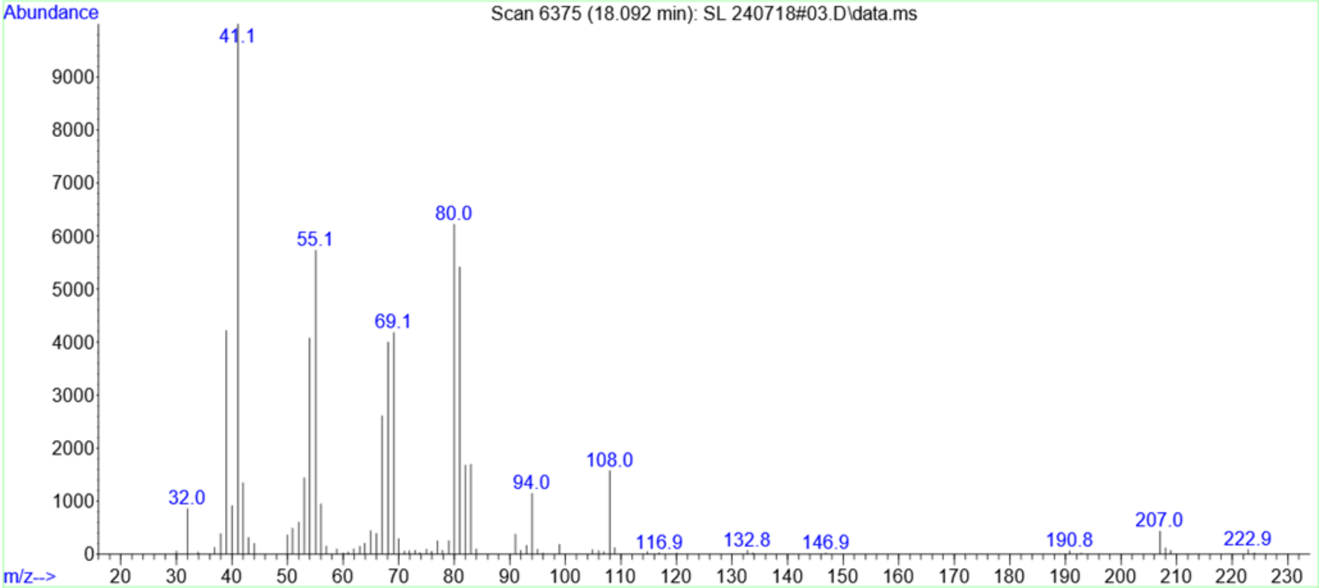

77. Thiiraneacetonitrile

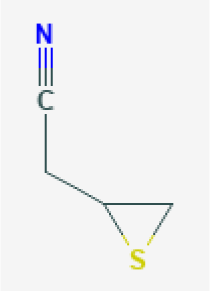

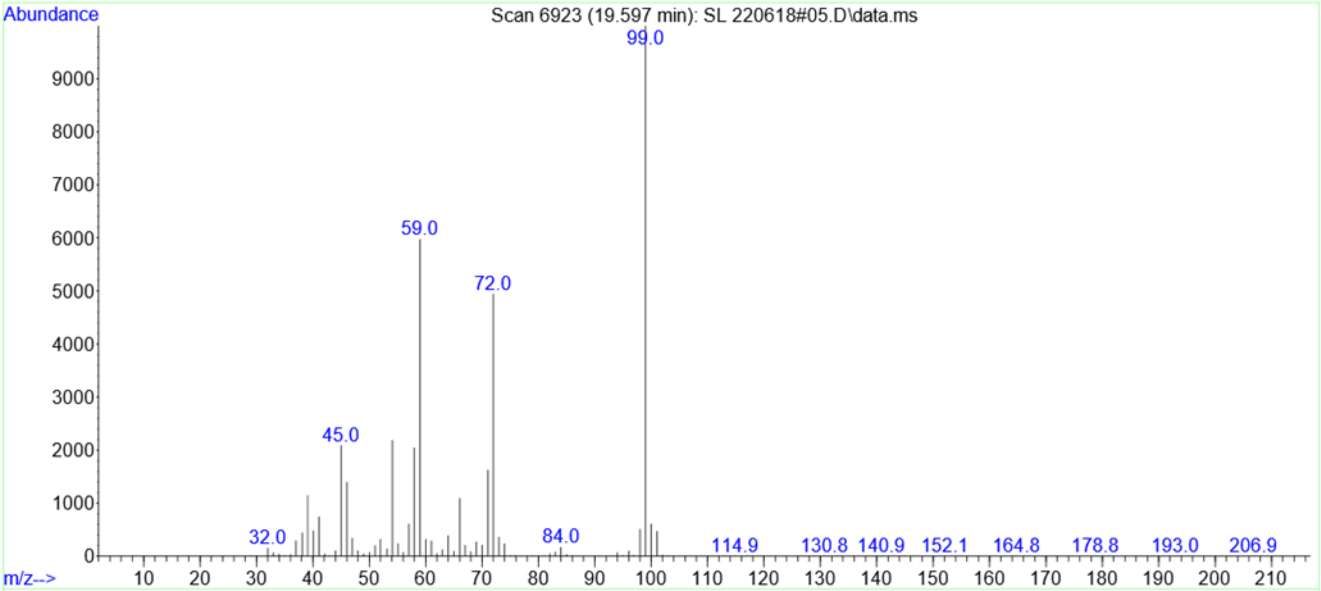

78. Phenylacetonitrile

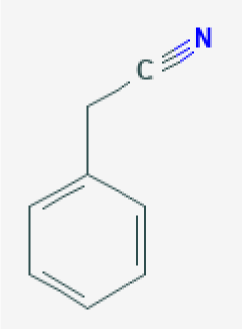

Library Searched : C:\Database\NIST11.L
Quality : 95
ID : Benzyl nitrile

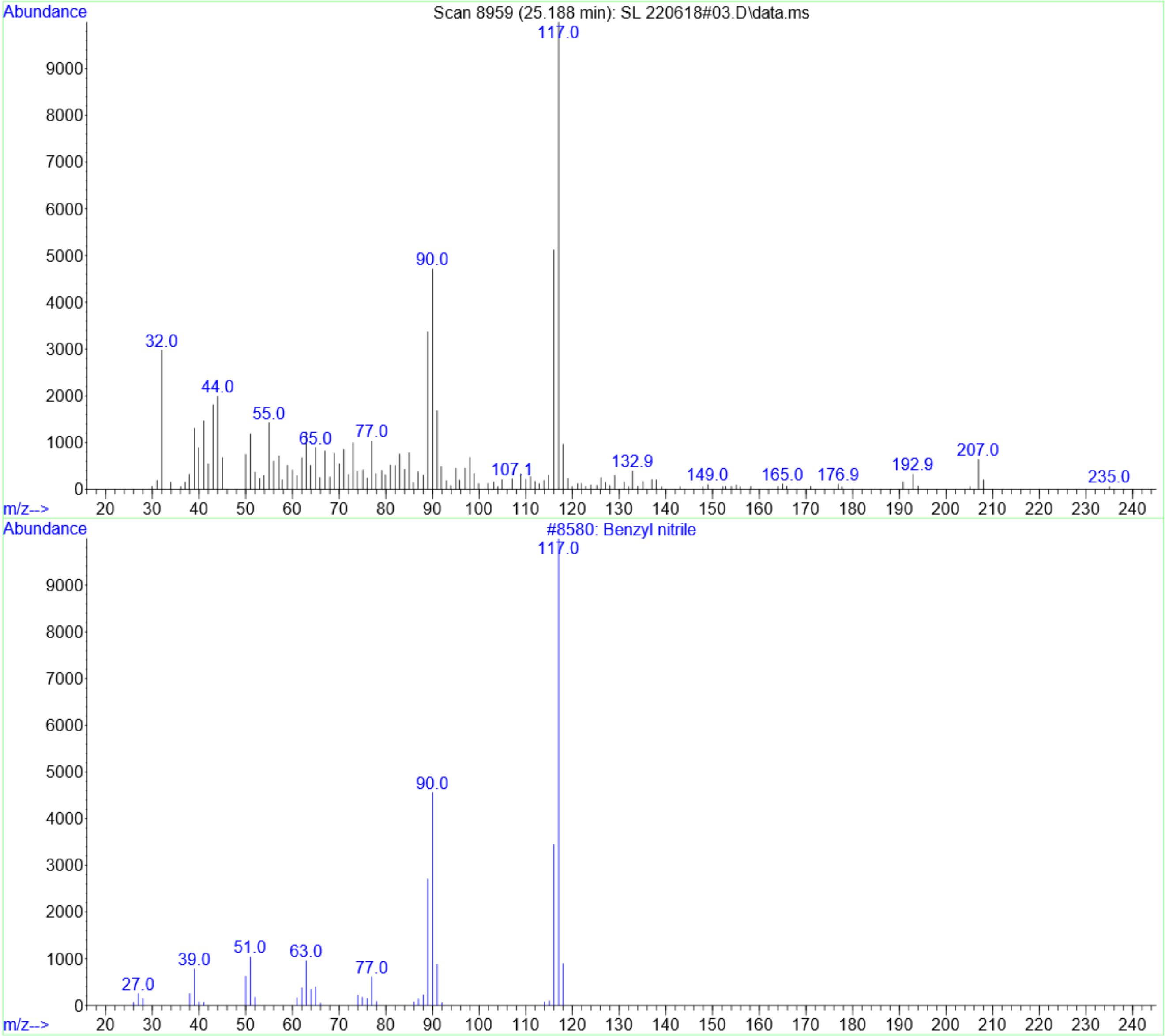

79. 5-(methylsulfanyl)pentanenitrile

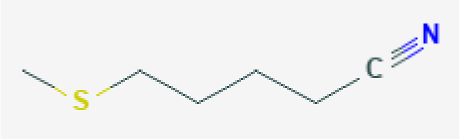

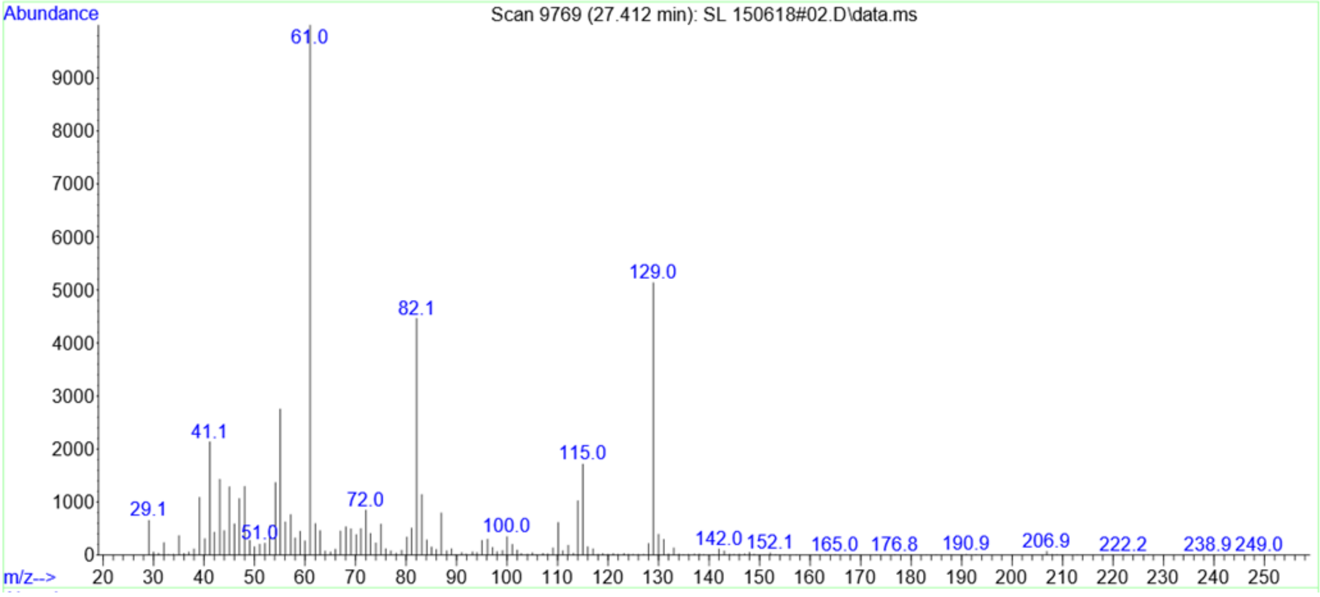

80. 4-(methylthio)butanenitrile

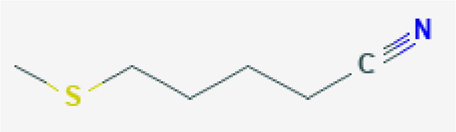

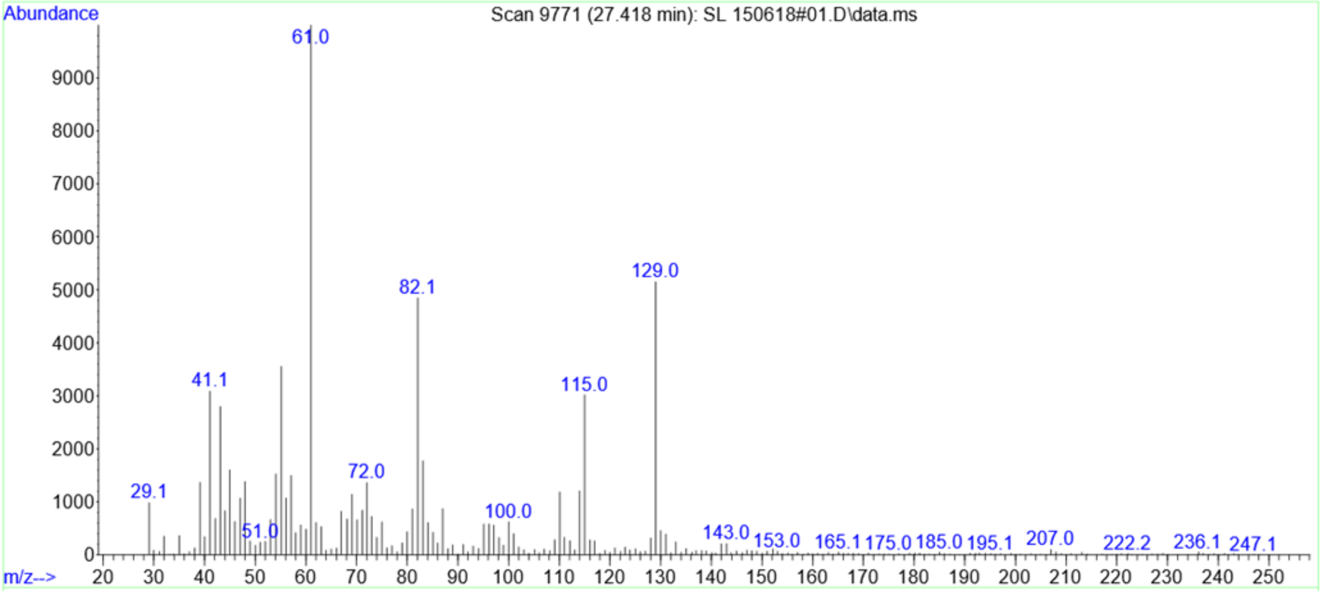

81. Benzenepropanenitrile

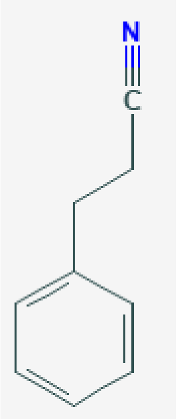

Library Searched : C:\Database\NIST11.L
Quality : 91
ID : Benzenepropanenitrile

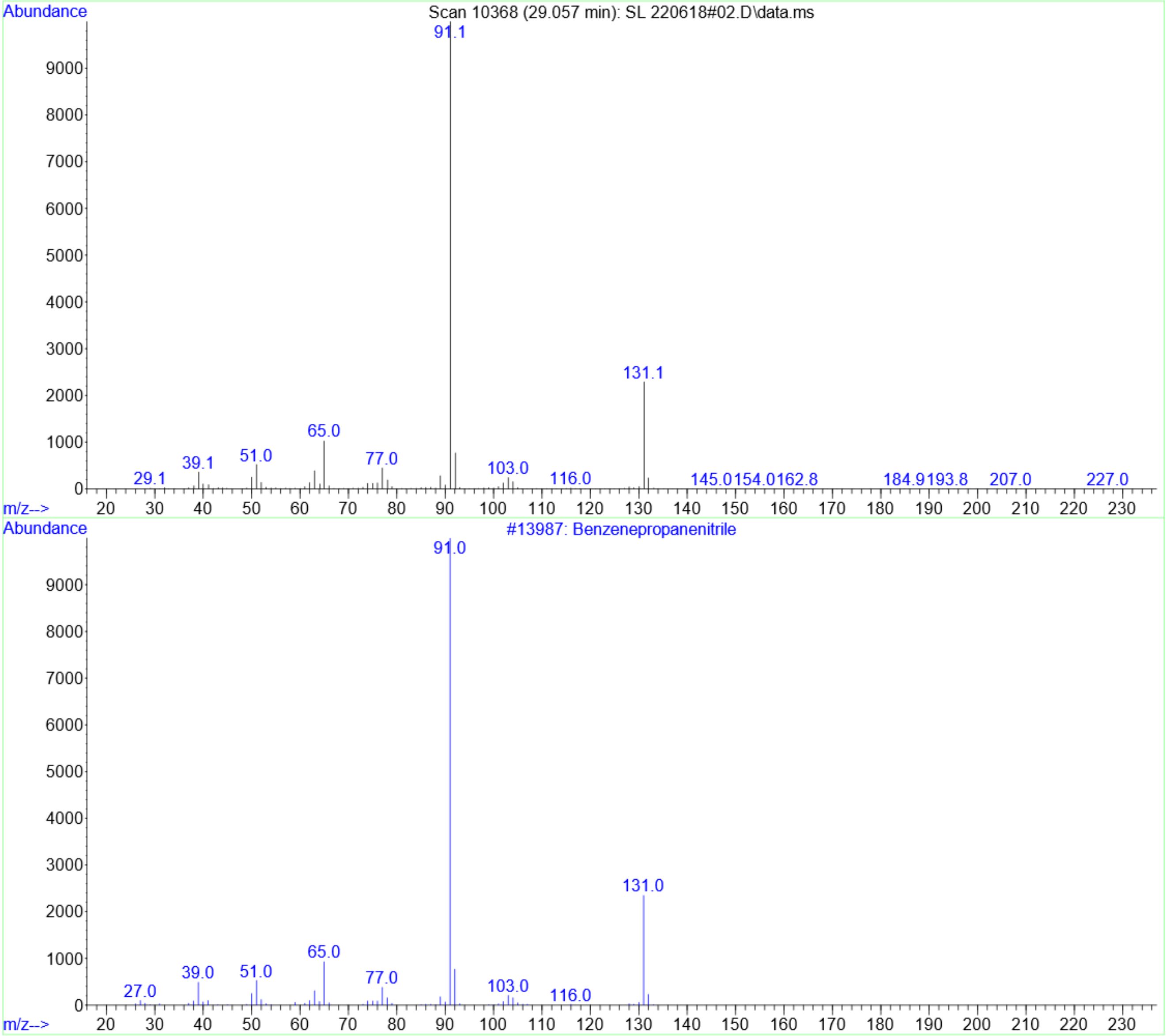

82. unidentified nitrile

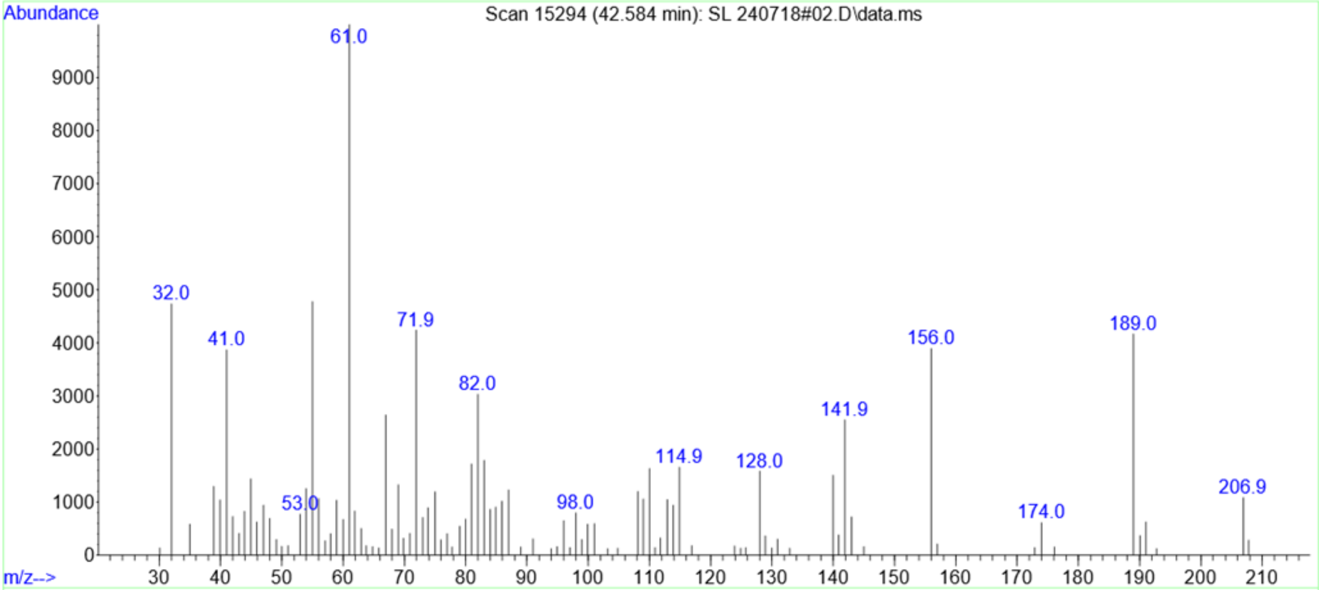

83. 2,2,4,6,6-pentamethylheptane

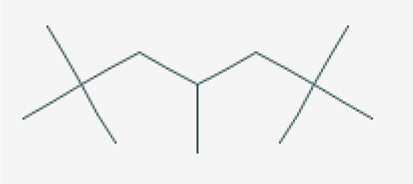

Library Searched : C:\Database\NIST11.L
Quality : 72
ID : Heptane, 2,2,4,6,6-pentamethyl-

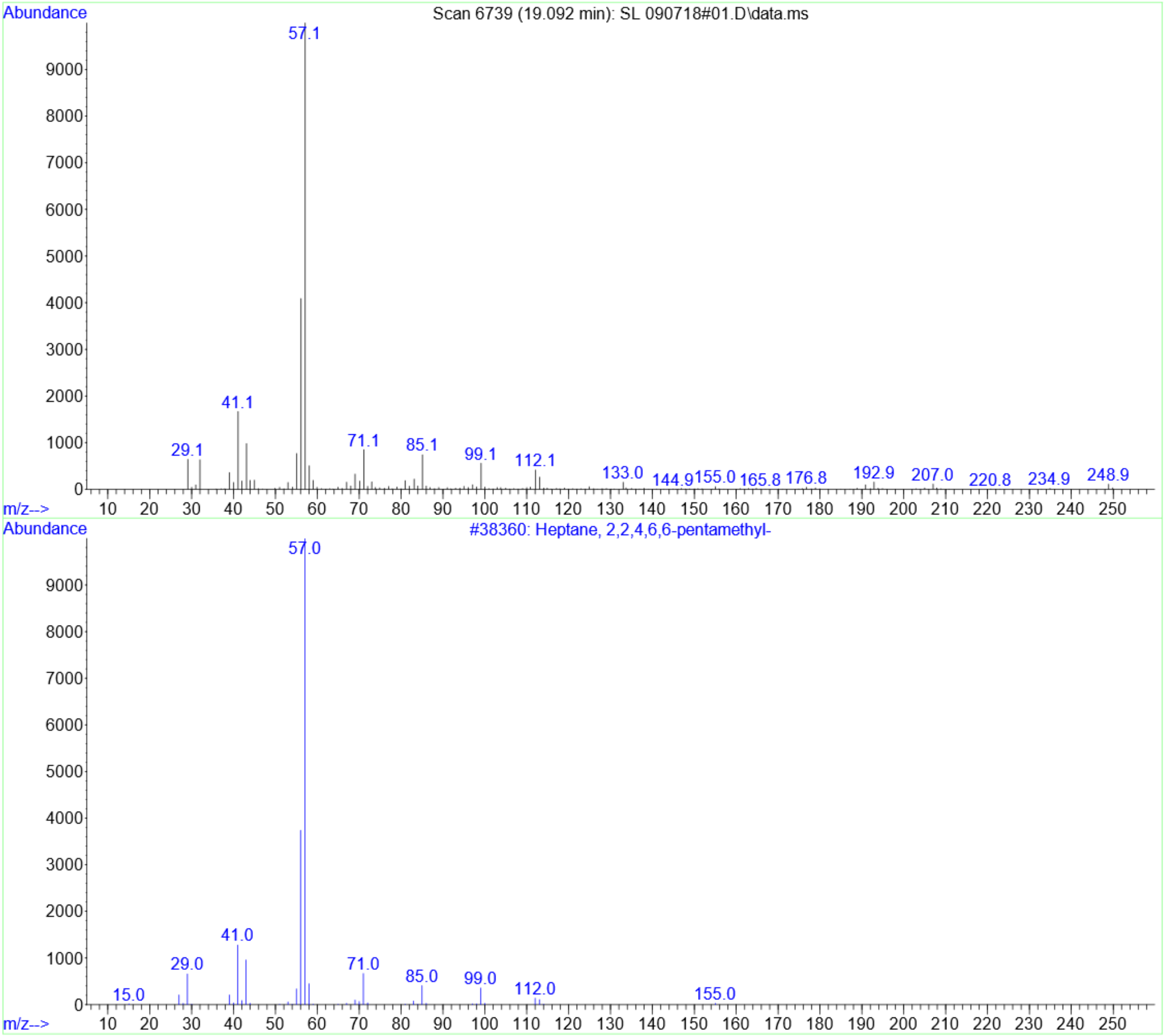

84. Undecane

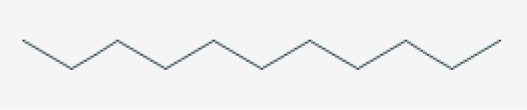

Library Searched : C:\Database\NIST11.L
Quality : 94
ID : Undecane

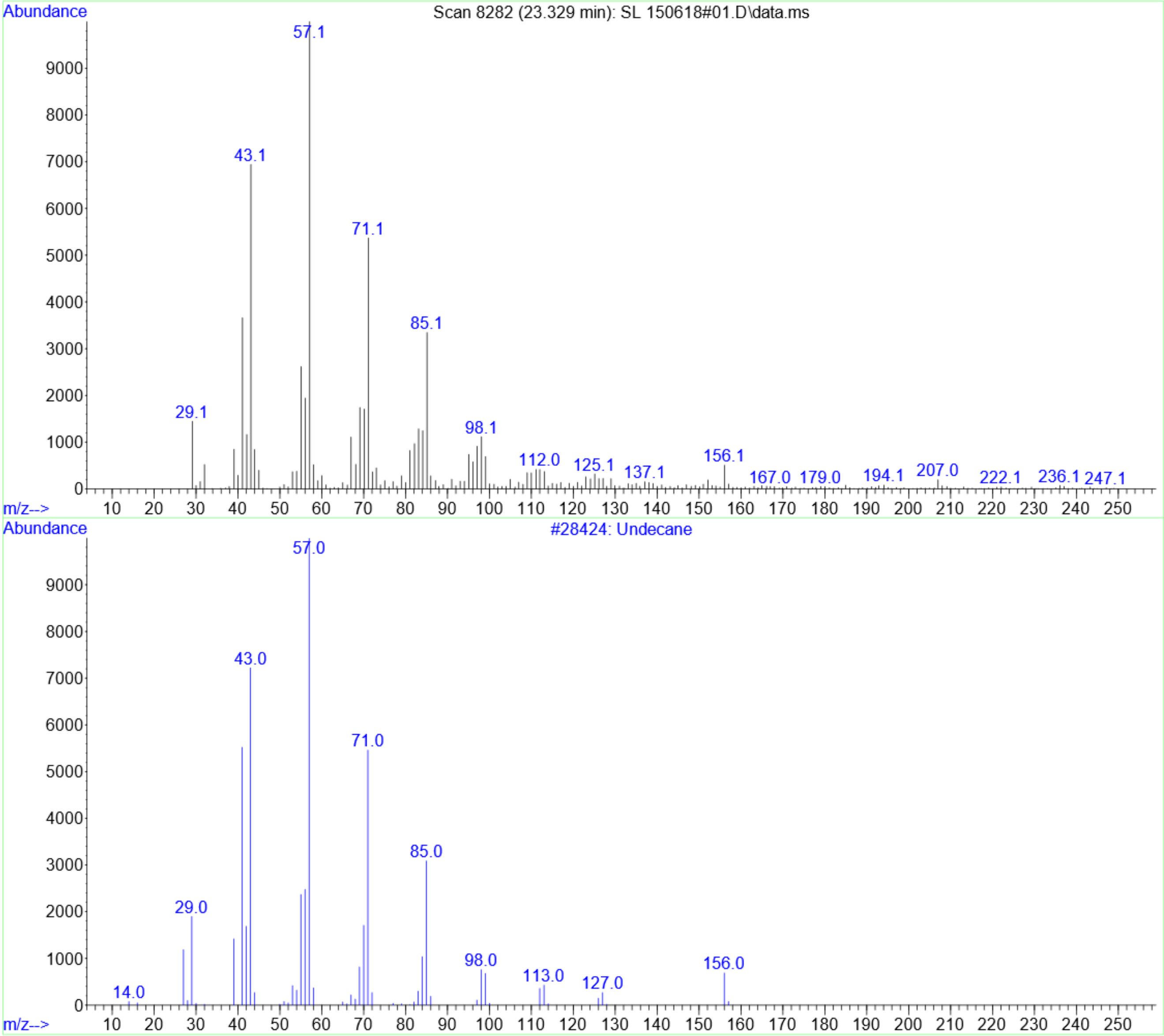

85. 1-dodecene

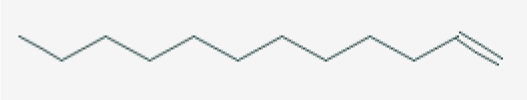

Library Searched : C:\Database\NIST11.L
Quality : 95
ID : 1-Dodecene

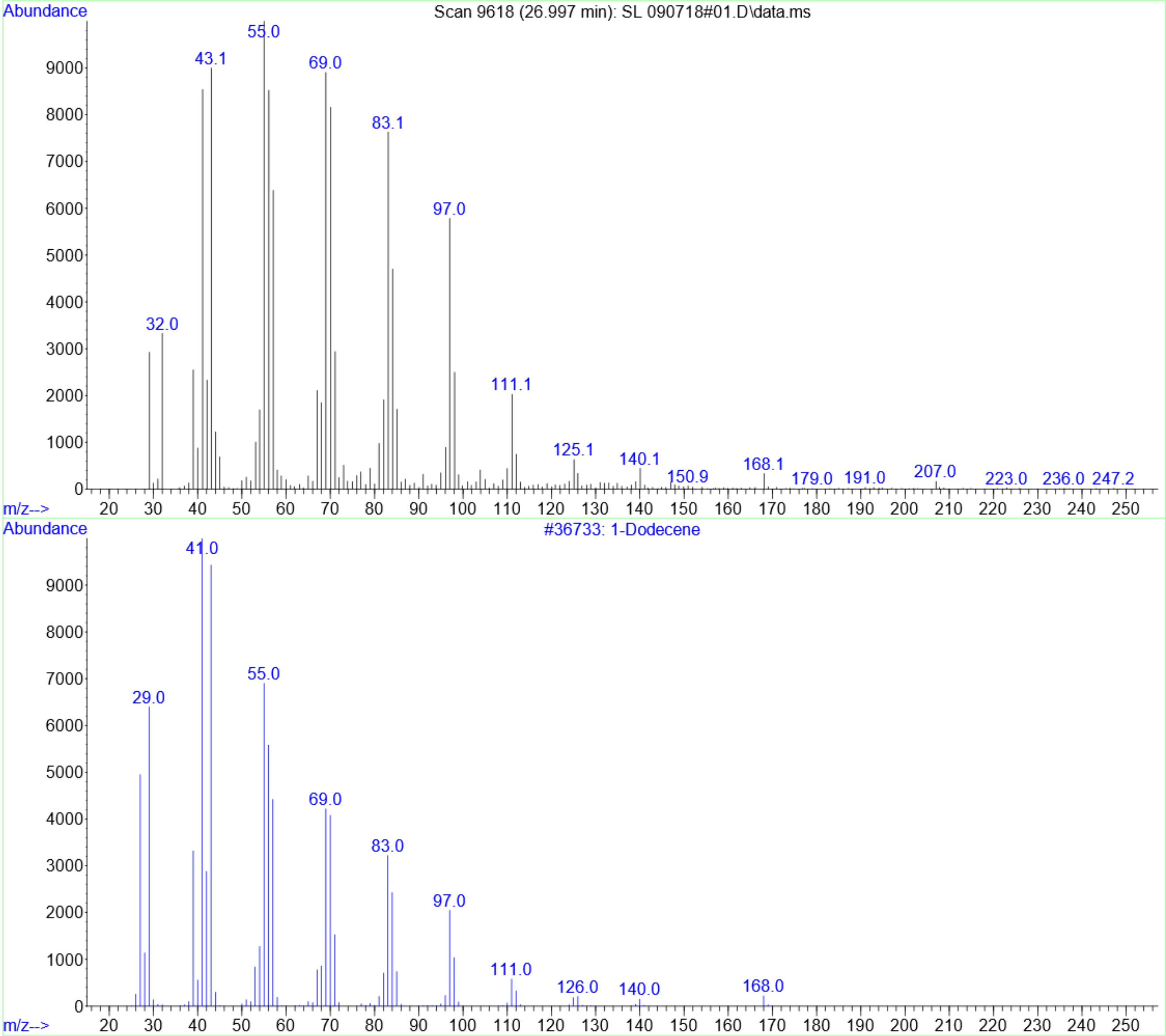

86. Dodecane

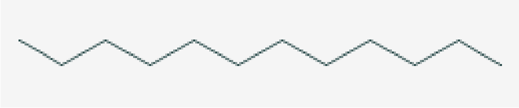

Library Searched : C:\Database\Adams.L
Quality : 95
ID : 15.77 Dodecane

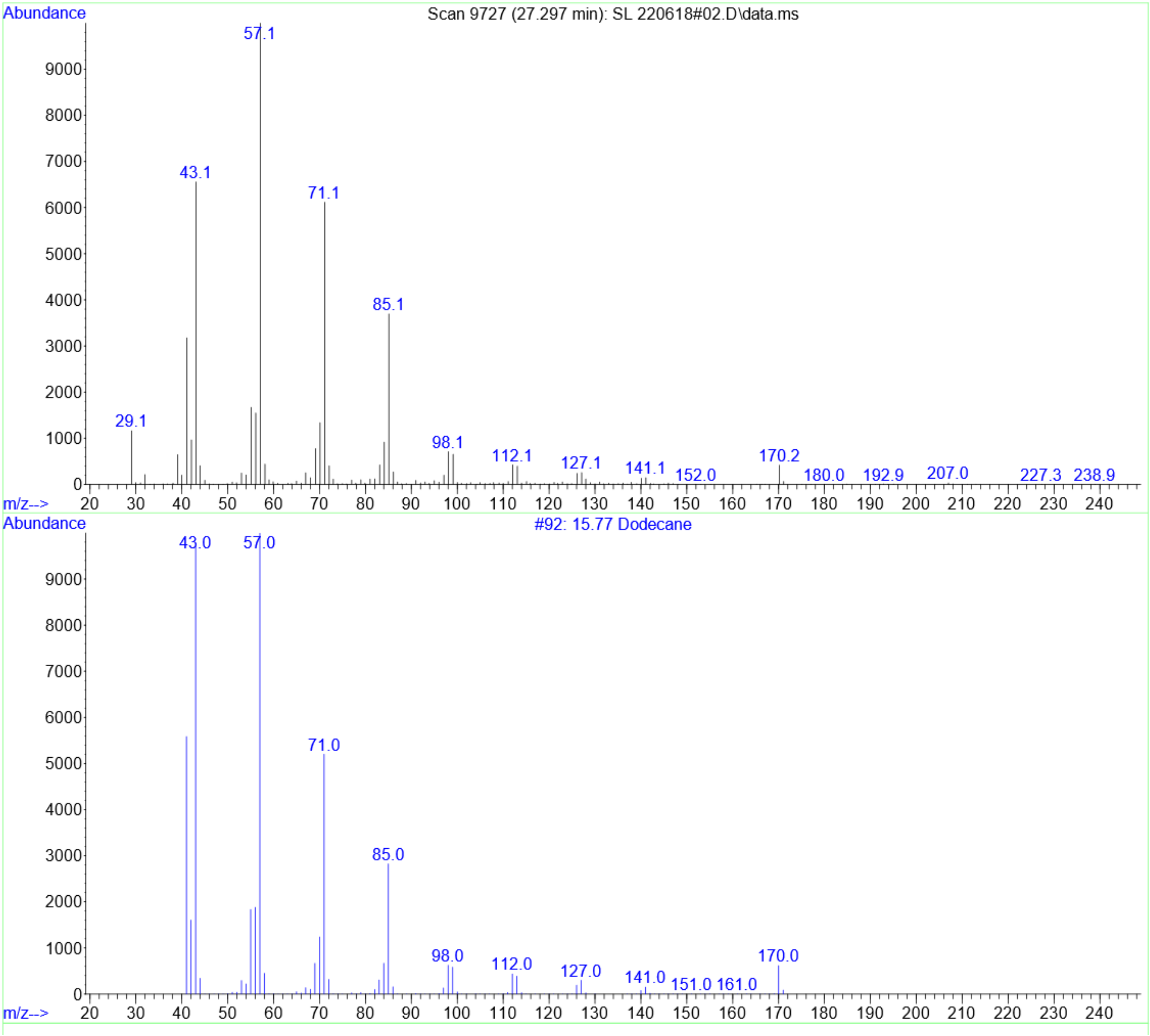

87. Tridecane

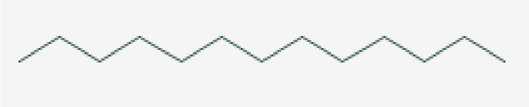

Library Searched : C:\Database\NIST11.L
Quality : 96
ID : Tridecane

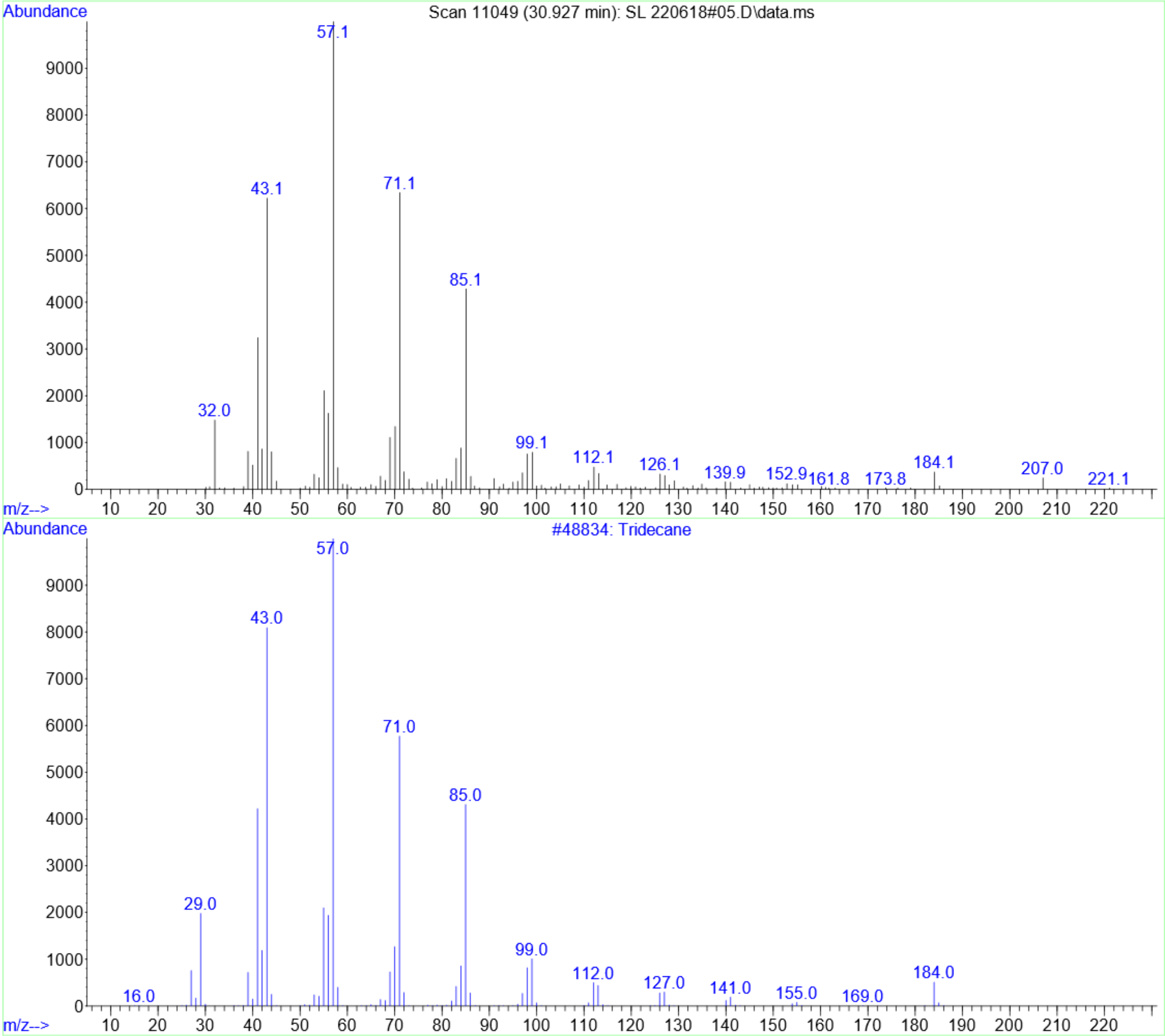

88. Tetradecane

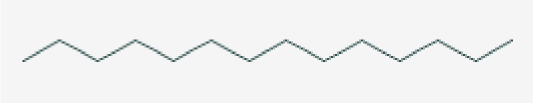

Library Searched : C:\Database\NIST11.L
Quality : 98
ID : Tetradecane

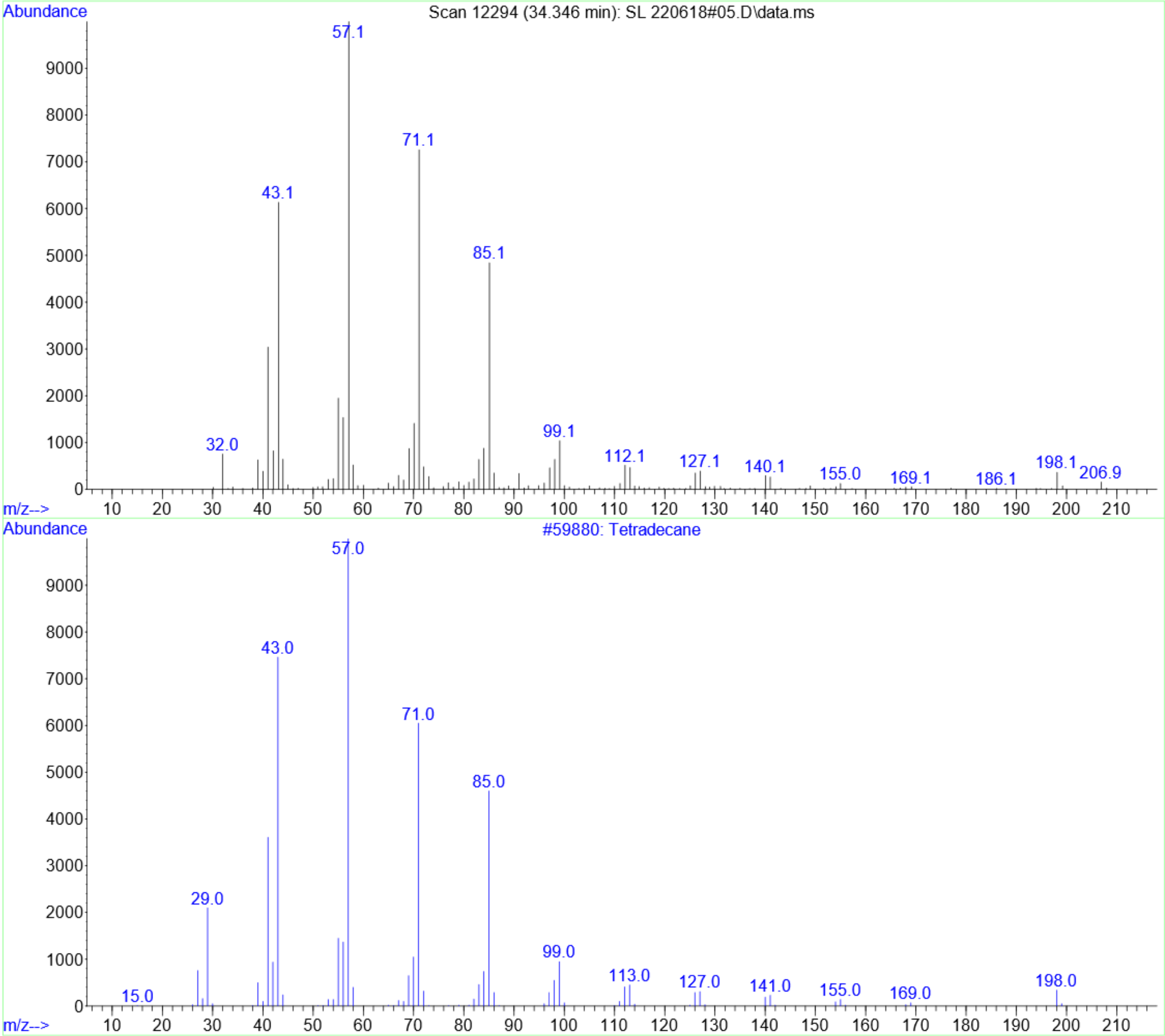

89. Pentadecane

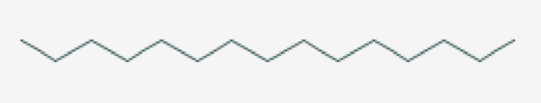

Library Searched : C:\Database\Adams.L
Quality : 90
ID : 28.82 Pentadecane

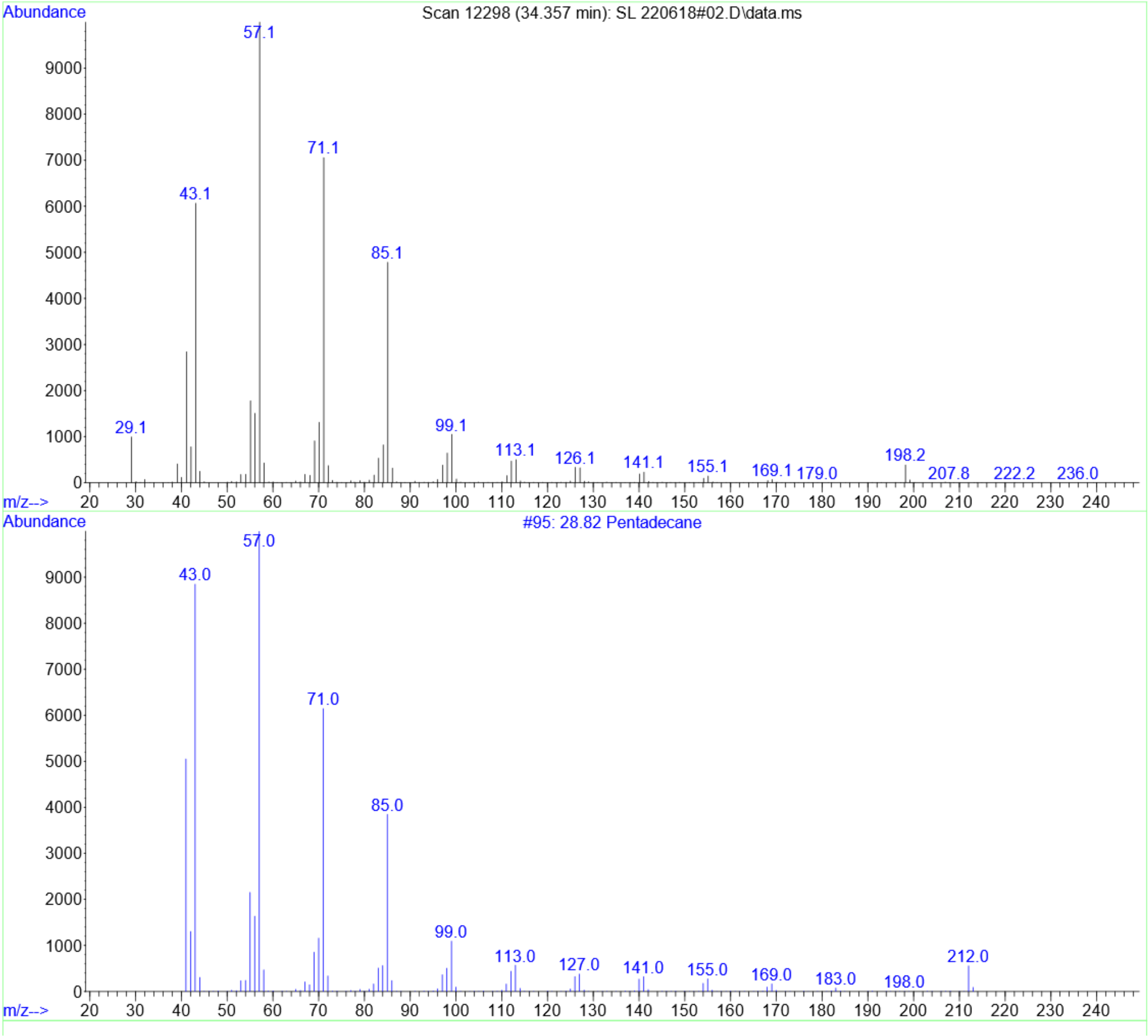

90. Hexadecane

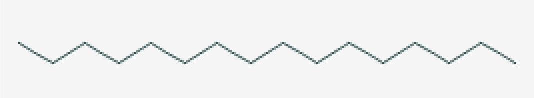

Library Searched : C:\Database\NIST11.L
Quality : 96
ID : Hexadecane

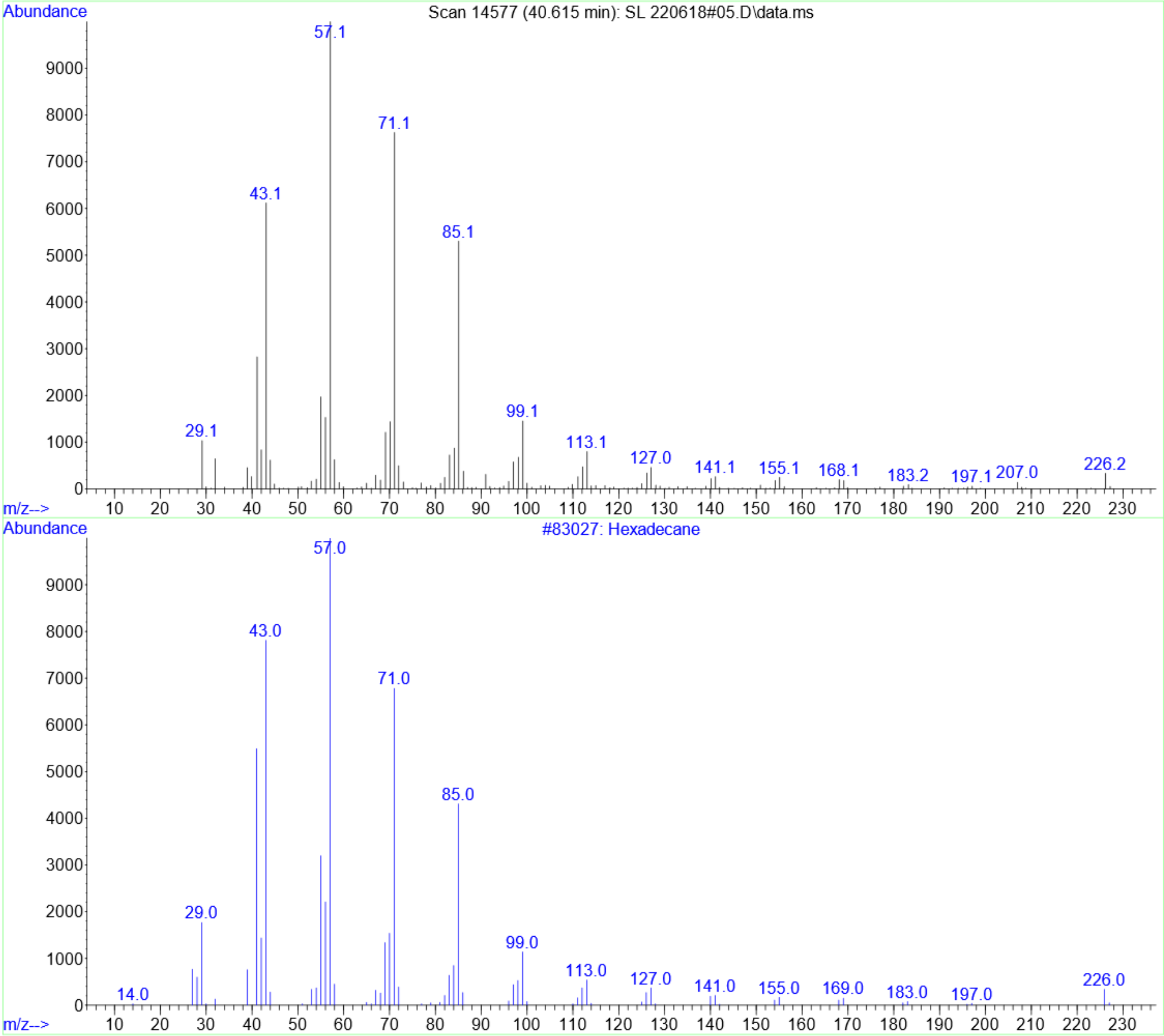

91. Heptadecane

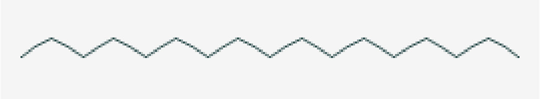

Library Searched : C:\Database\NIST11.L
Quality : 98
ID : Heptadecane

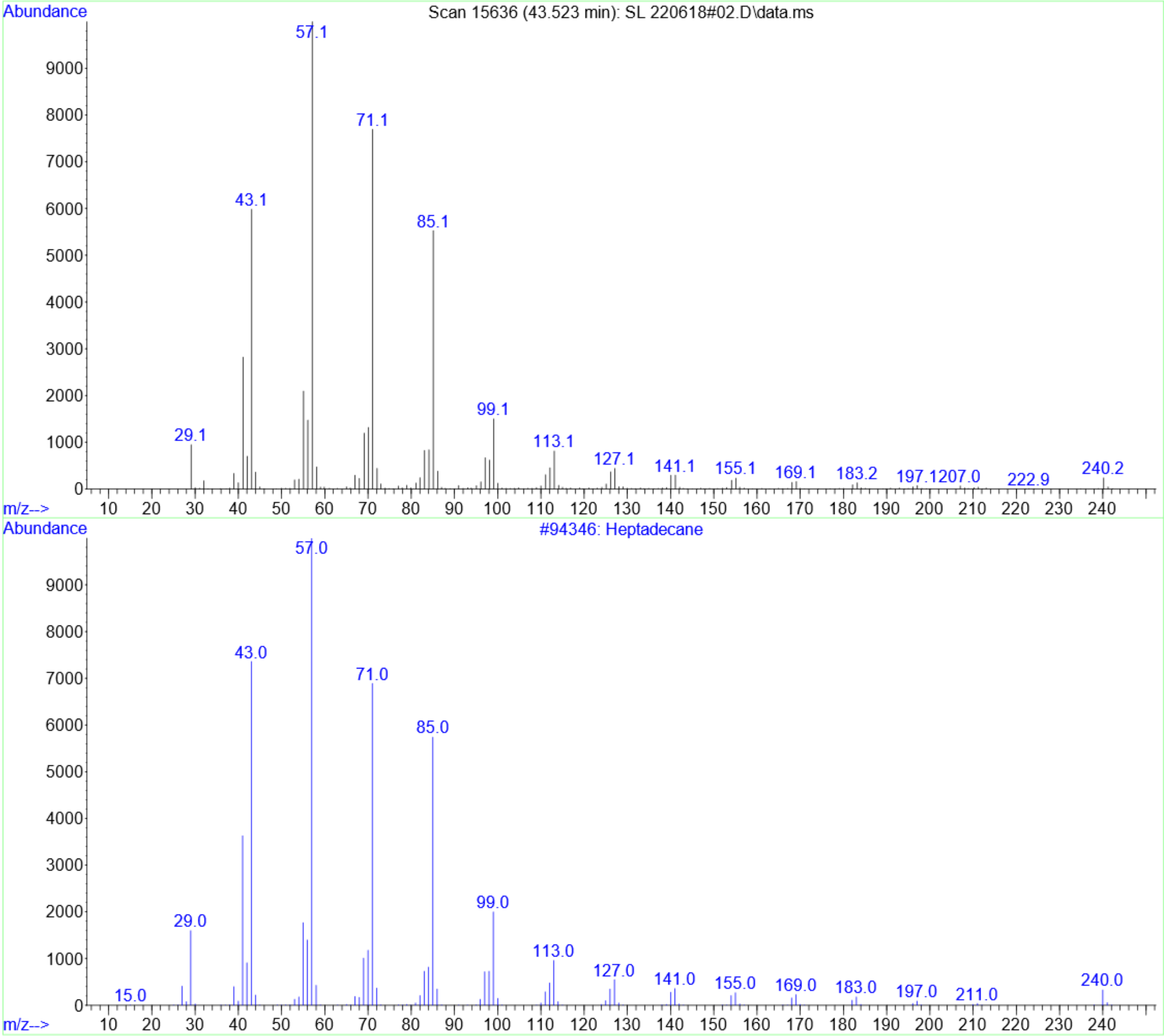

92. Octadecane

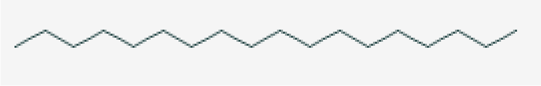

Library Searched : C:\Database\NIST11.L
Quality : 91
ID : Octadecane

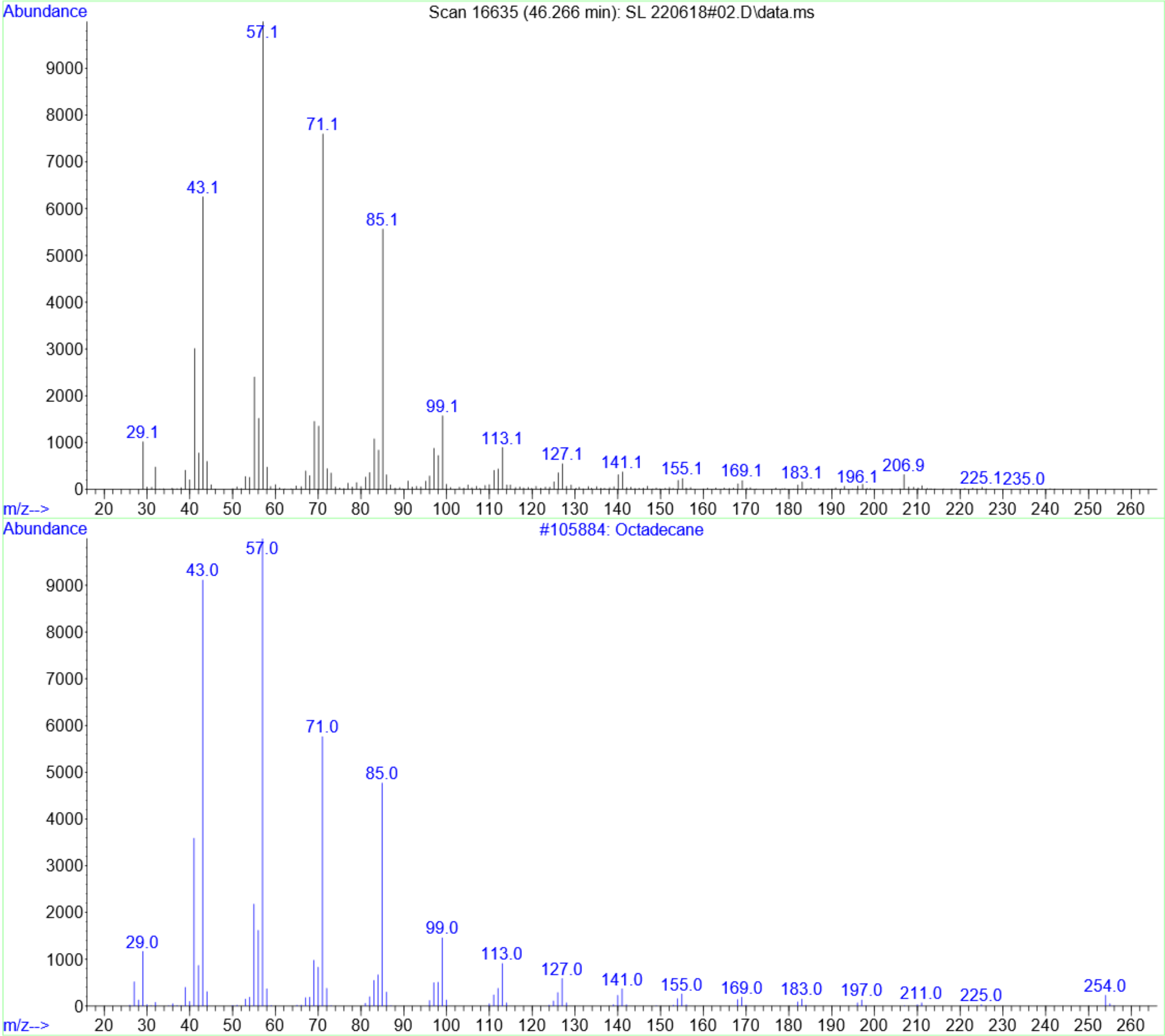

94. *P*-cymene

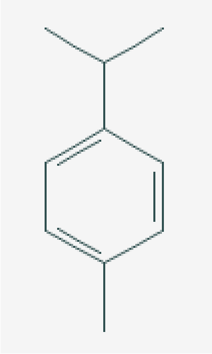

Library Searched : C:\Database\NIST11.L
Quality : 92
ID : p-Cymene

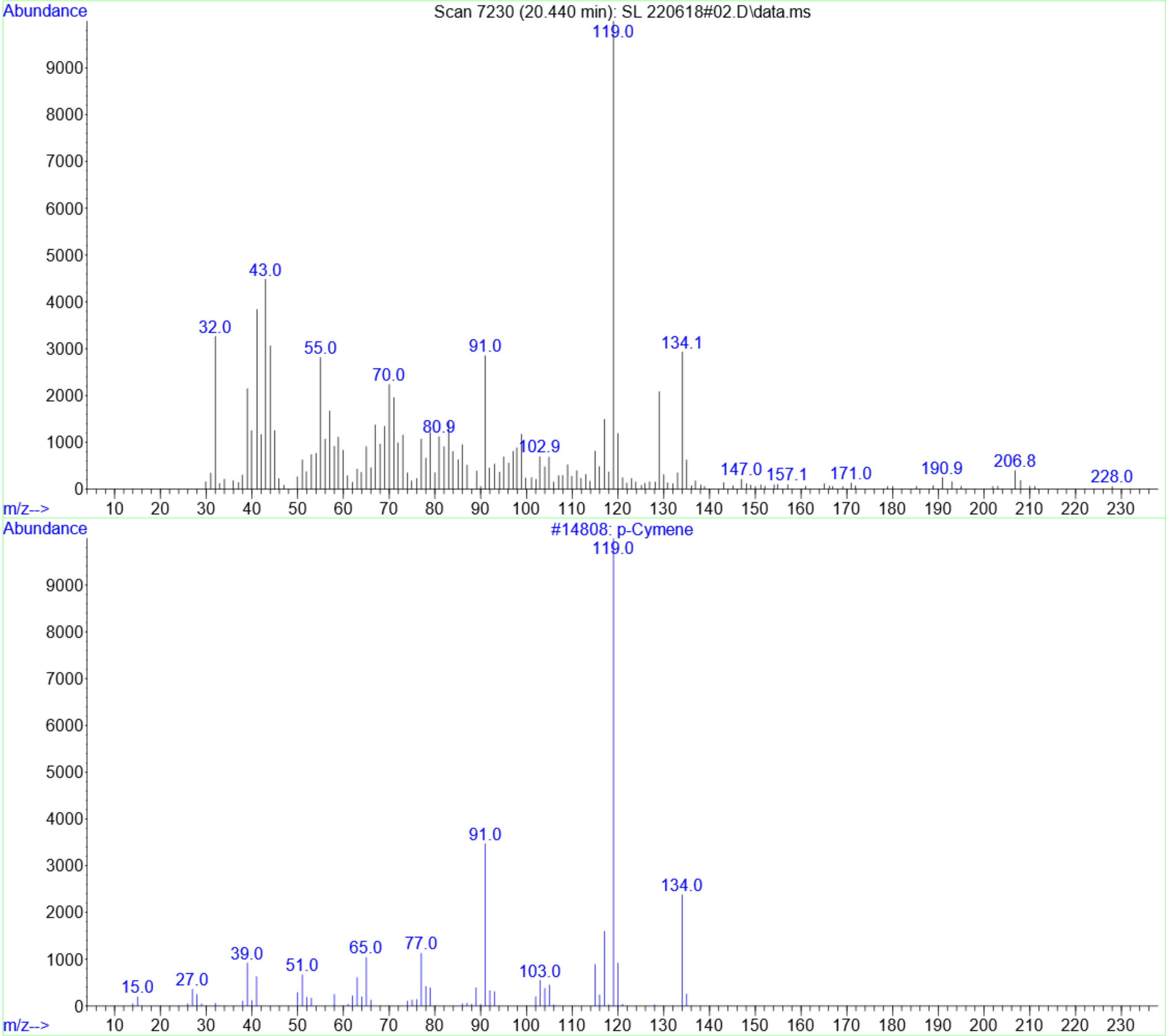

95. *O*-cymene

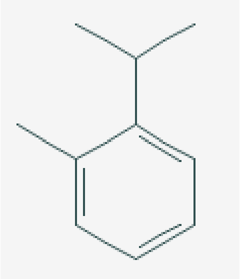

Library Searched : C:\Database\Adams.L
Quality : 95
ID : 8.59 Cymene<ortho->

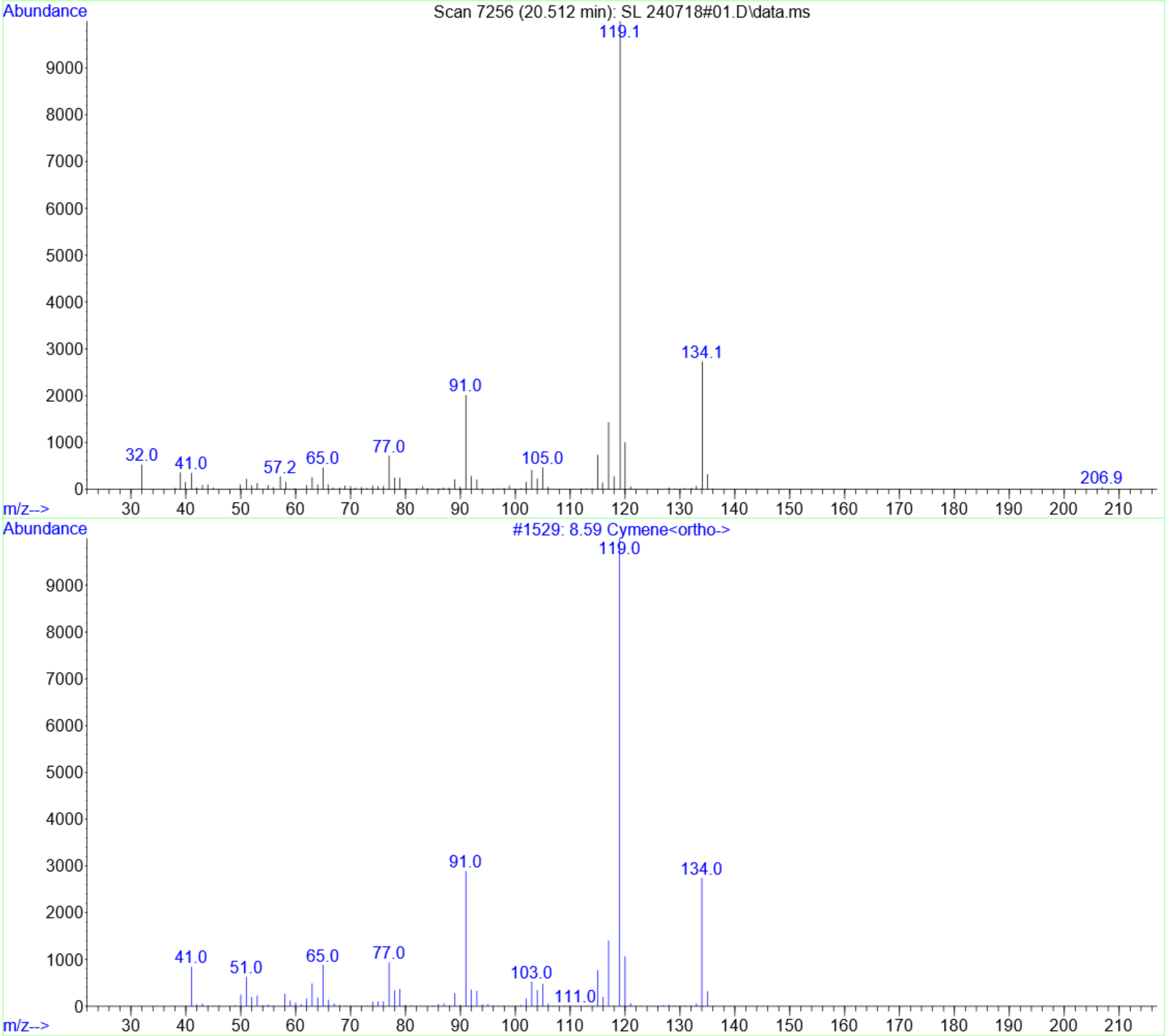

96. *D*-limonene

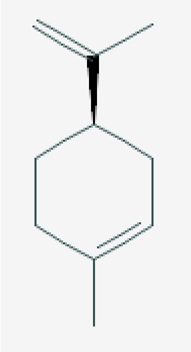

Library Searched : C:\Database\NIST11.L
Quality : 99
ID : D-Limonene

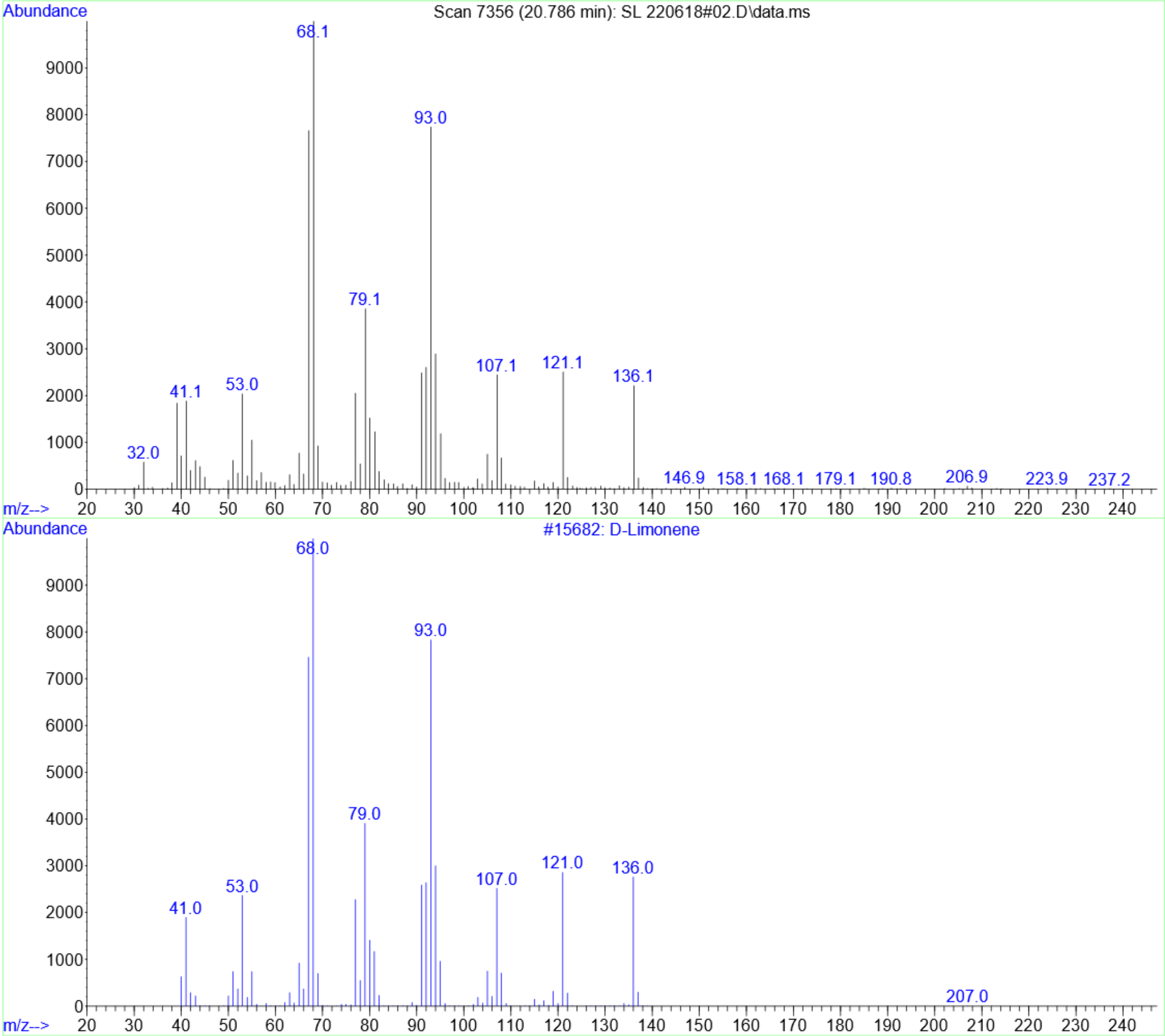

97. Eucalyptol

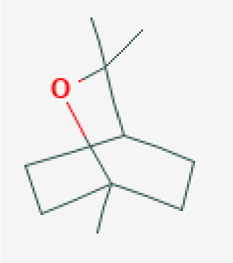

Library Searched : C:\Database\NIST11.L
Quality : 98
ID : Eucalyptol

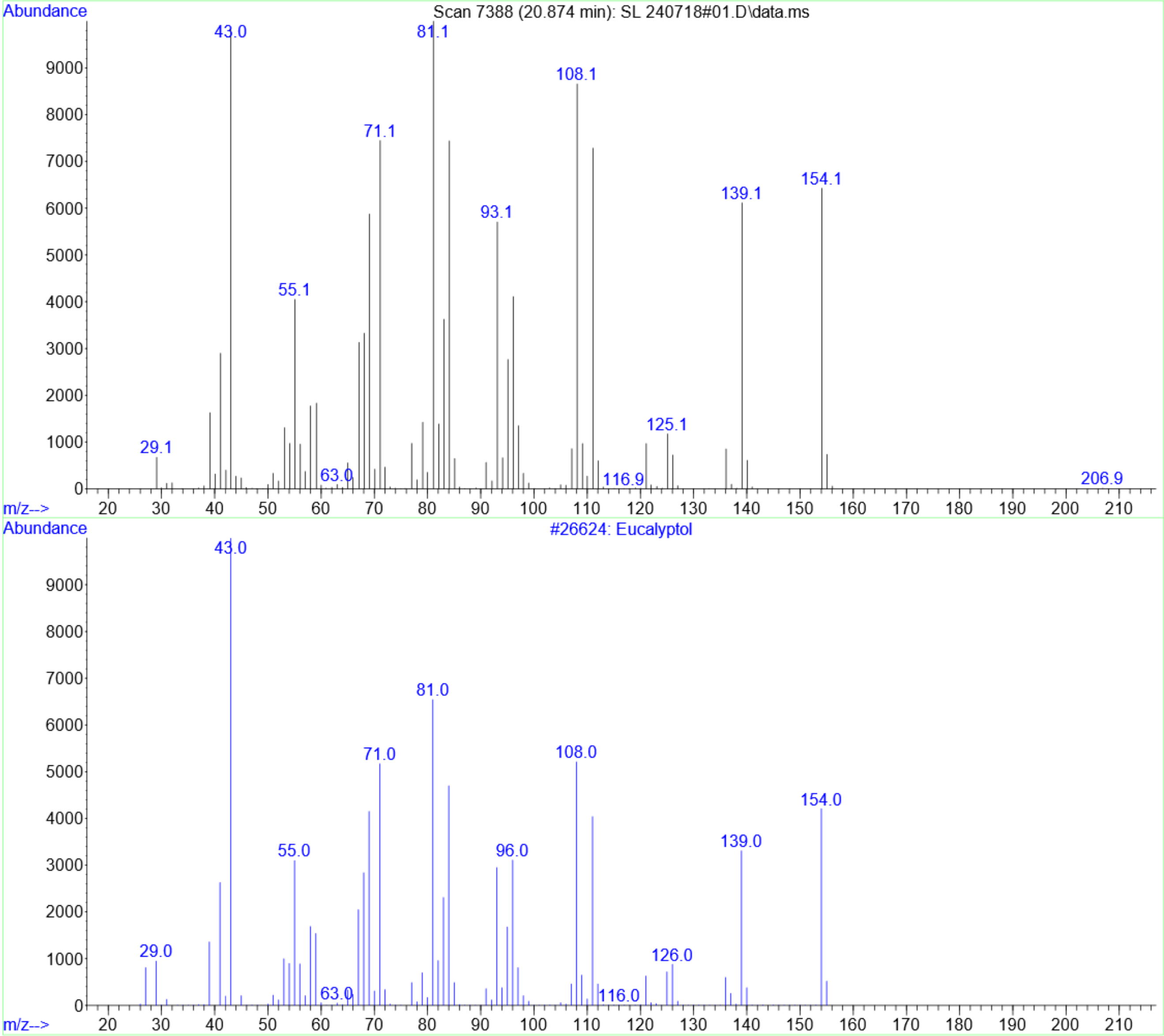

98. β-ionone

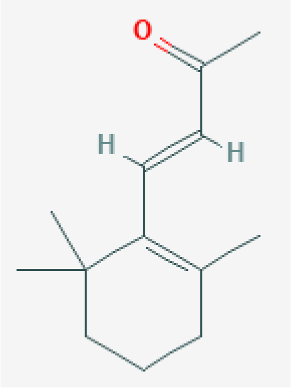

Library Searched : C:\Database\NIST11.L
Quality : 97
ID : 3-Buten-2-one, 4-(2,6,6-trimethyl-1-cyclohexen-1-yl)-

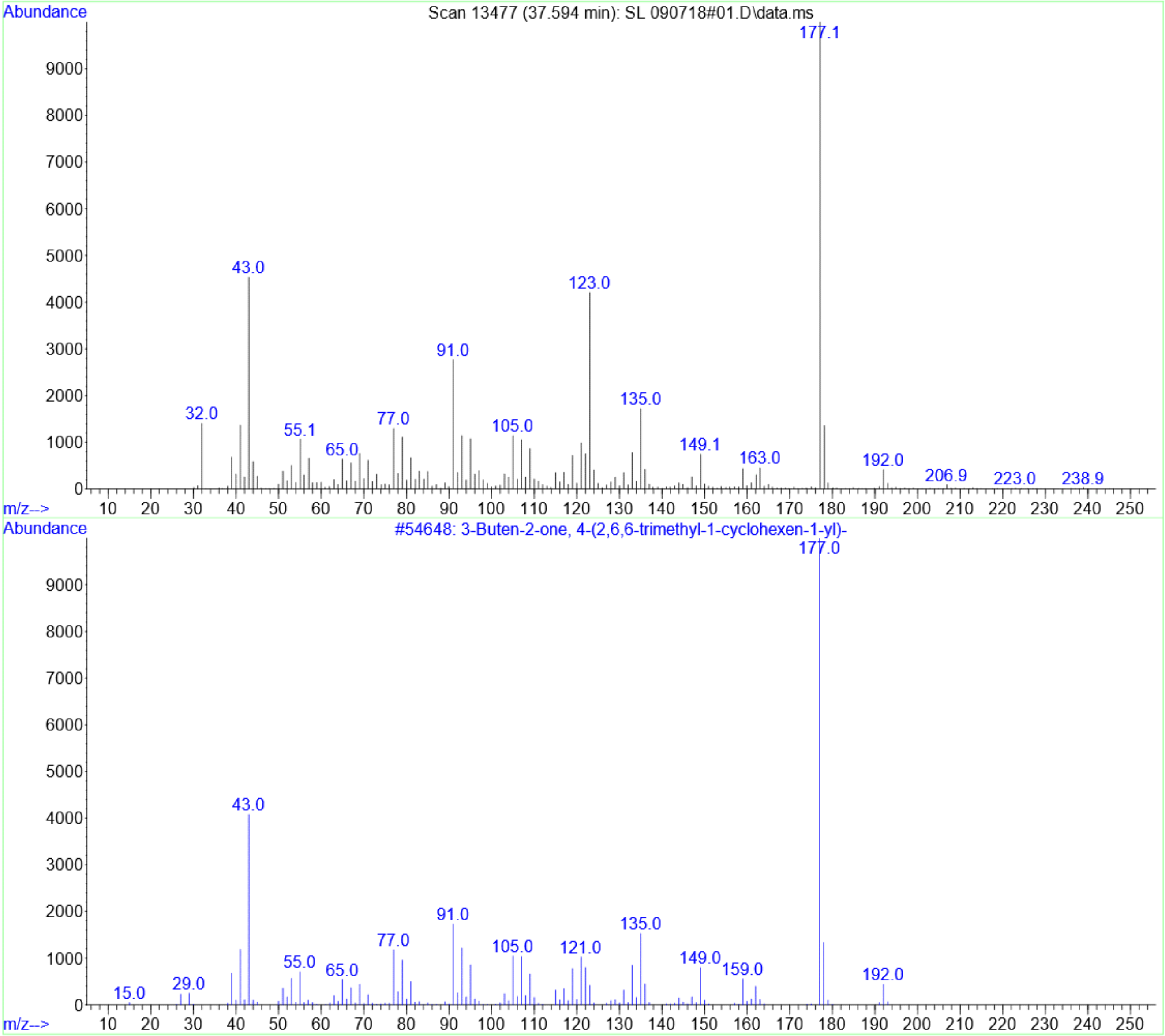

99. Allyl isocyanate

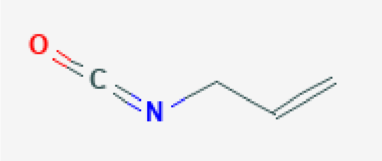

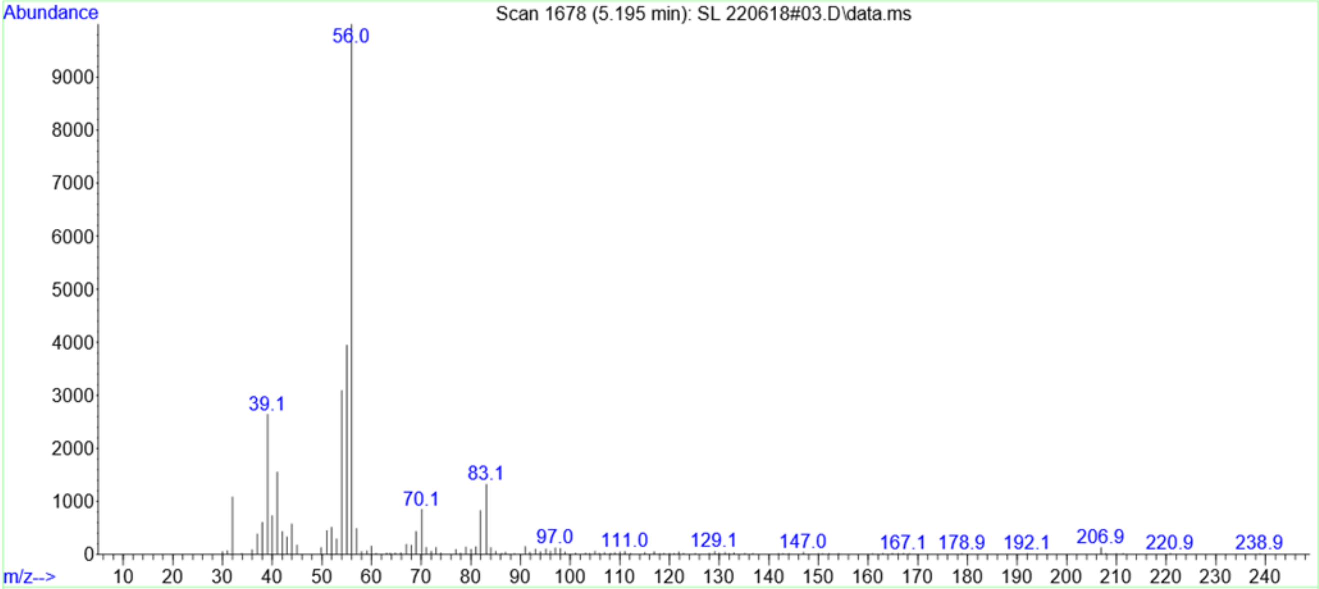

100. 2-ethylfuran

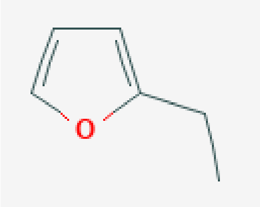

Library Searched : C:\Database\NIST11.L
Quality : 76
ID : Furan, 2-ethyl-

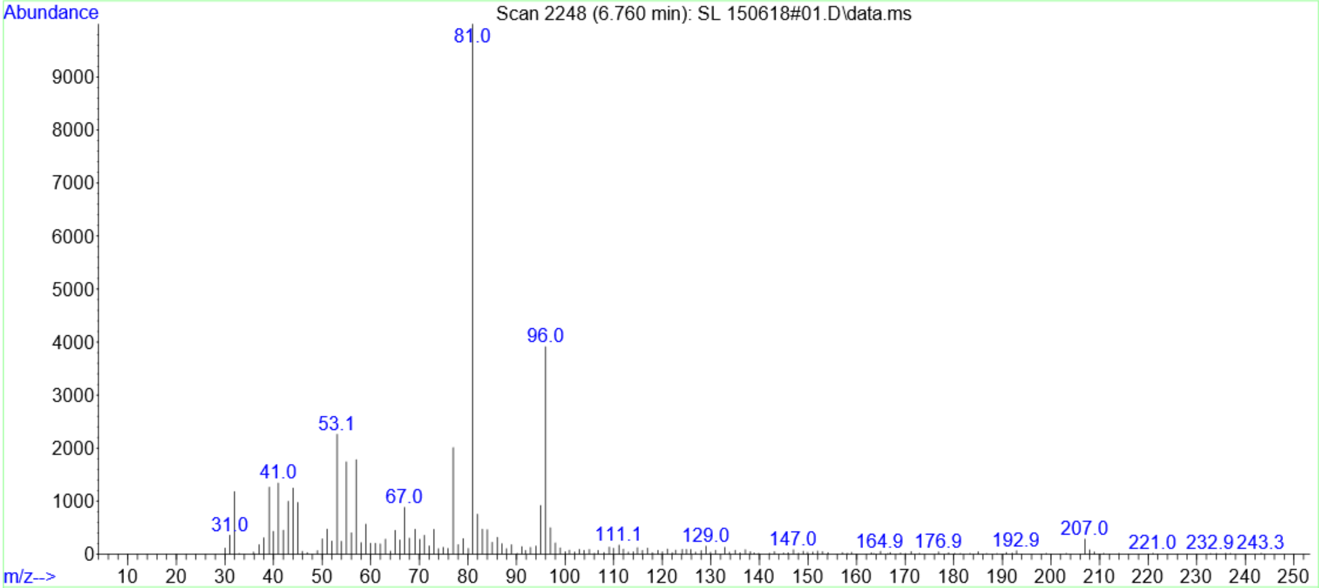

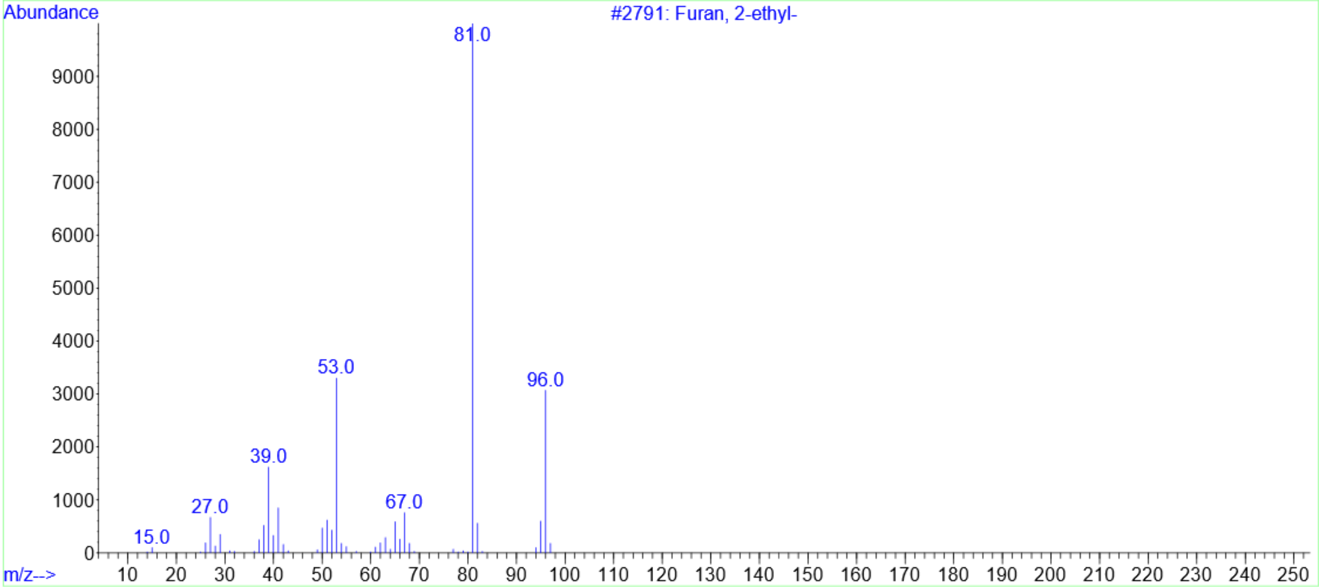

102. 2-isopropyl-3-methoxypyrazine

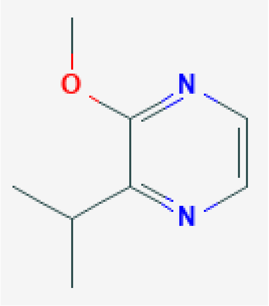

Library Searched : C:\Database\NIST11.L
Quality : 96
ID : Pyrazine, 2-methoxy-3-(1-methylpropyl)-

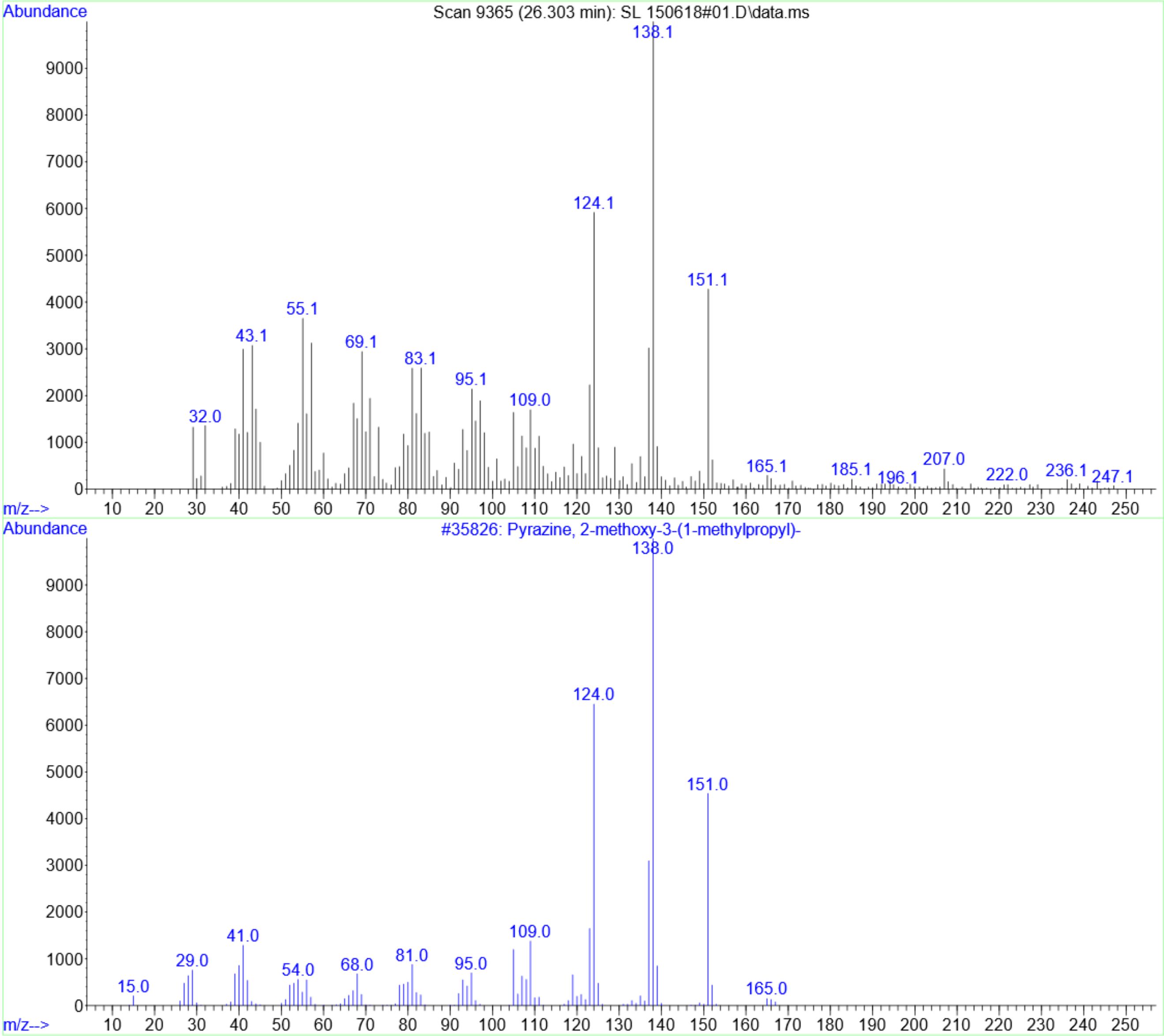

103. 1,2,3,5-tetramethylbenzene

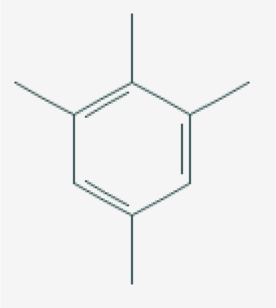

Library Searched : C:\Database\LAB429.L
Quality : 38
ID : 1,2,3,5-Tetramethylbenzene

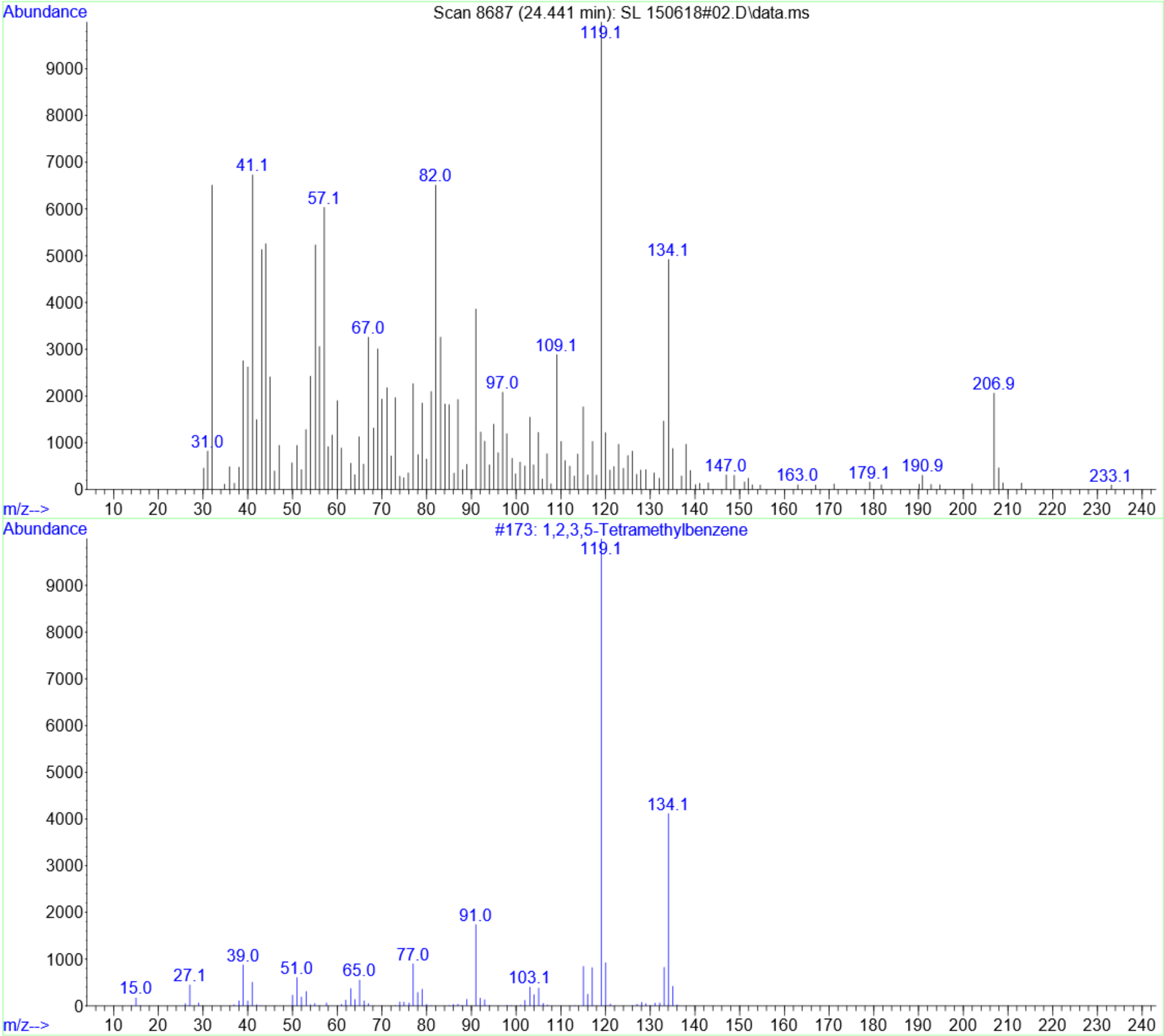

104. Veratrole

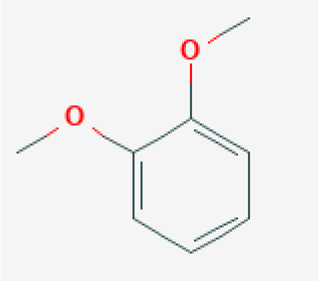

Library Searched : C:\Database\Adams.L
Quality : 87
ID : 13.28 Veratrole

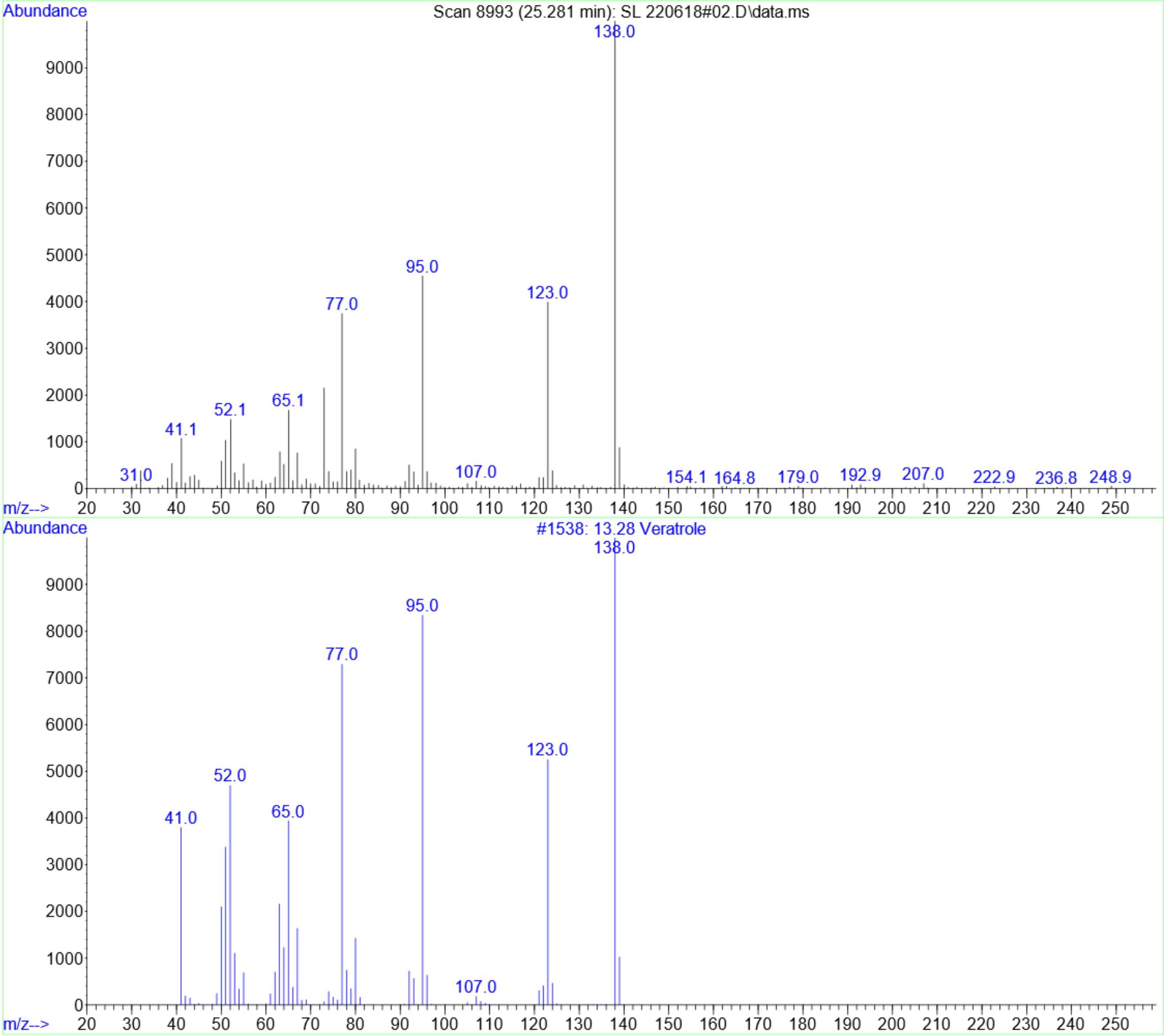

105. Octanoic acid

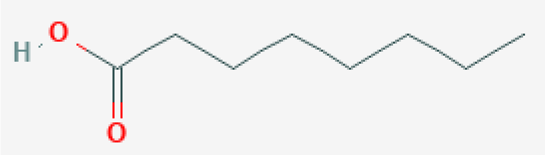

Library Searched : C:\Database\NIST11.L
Quality : 64
ID : Octanoic acid

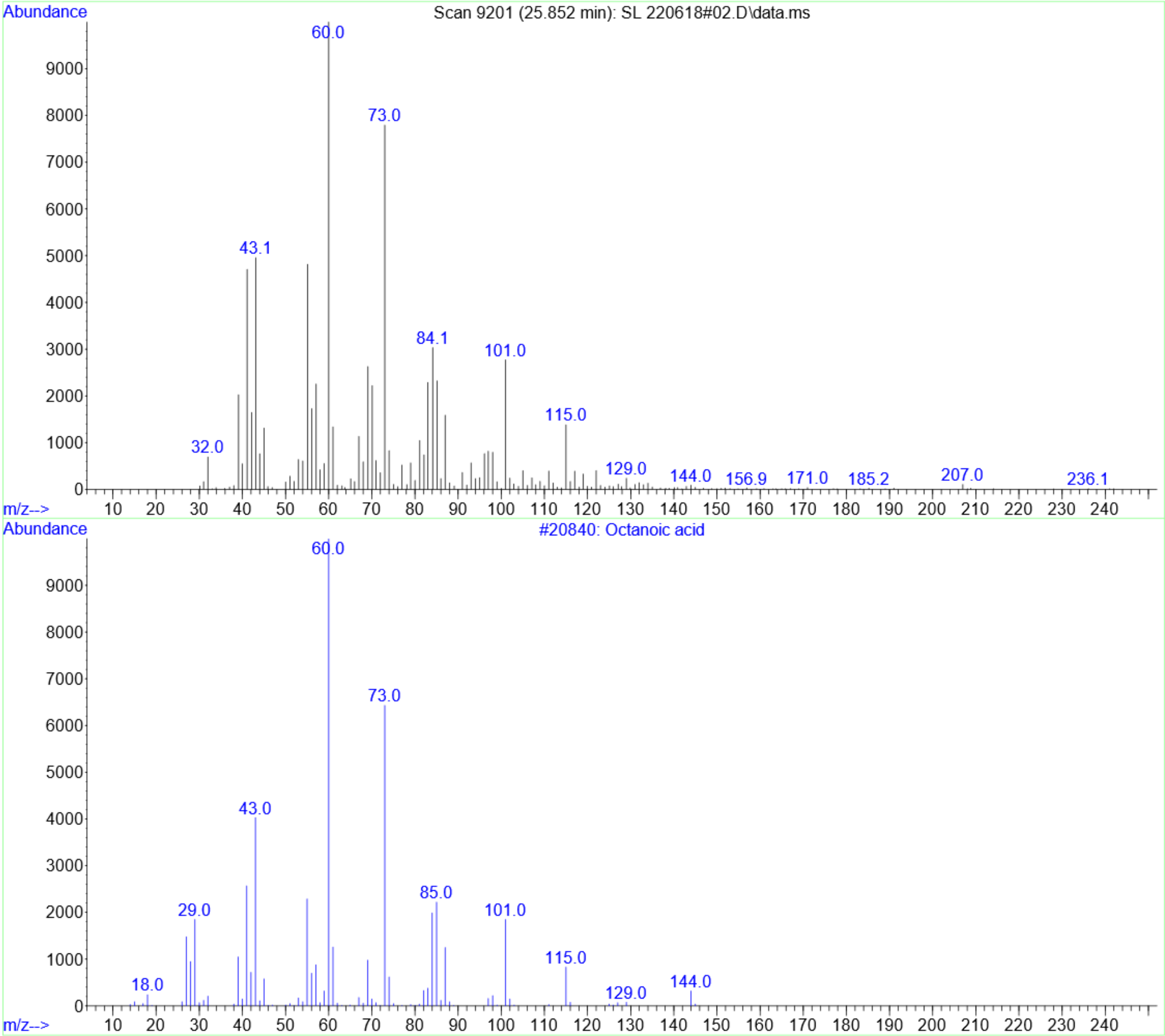

106. 2-*sec*-butyl-3-methoxypyrazine

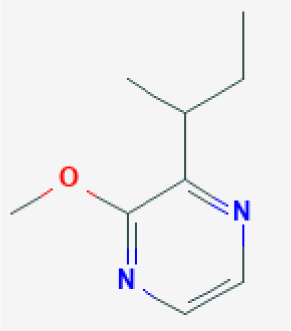

Library Searched : C:\Database\NIST11.L
Quality : 97
ID : Pyrazine, 2-methoxy-3-(1-methylpropyl)-

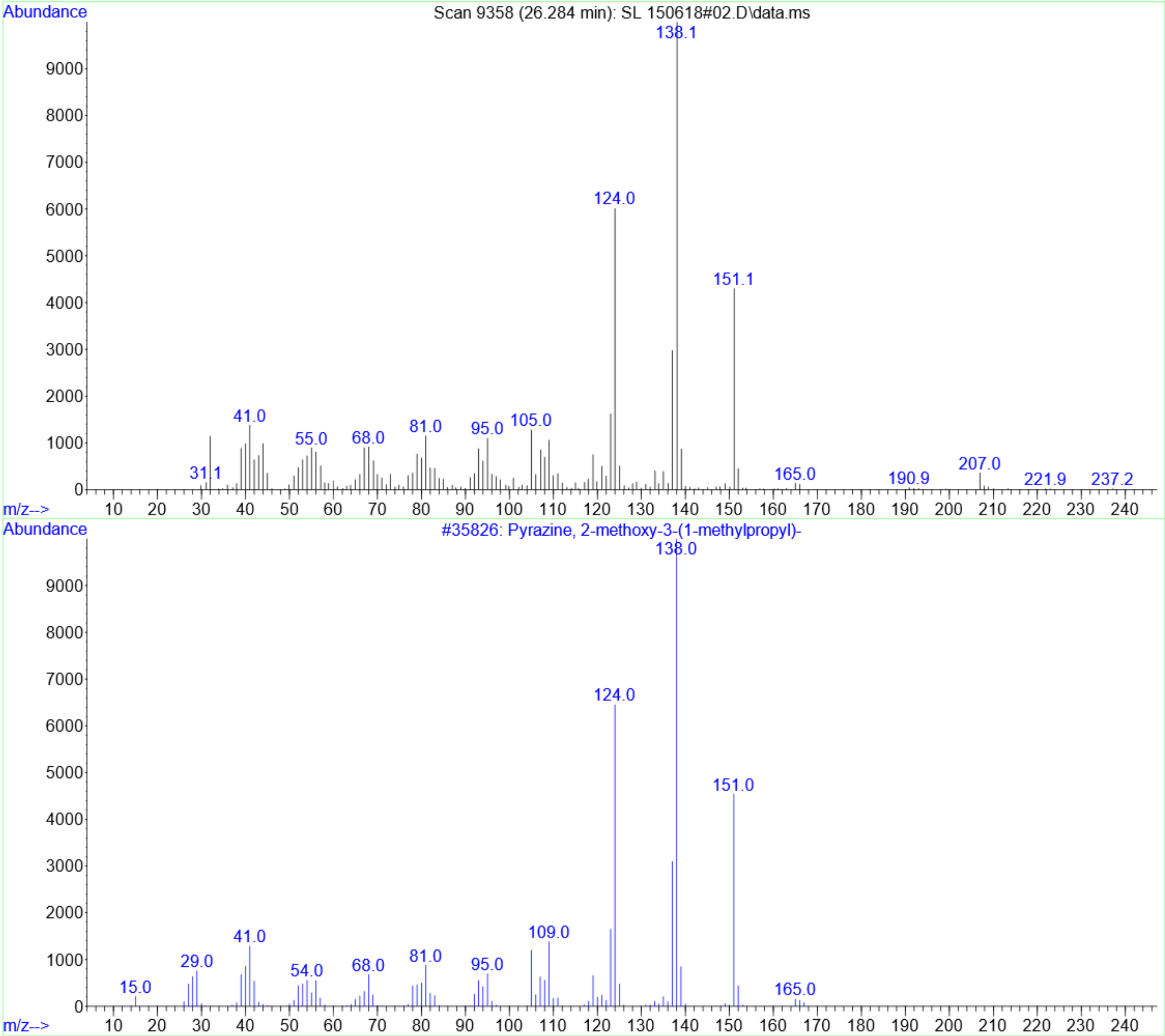

107. Phenethyl isocyanate

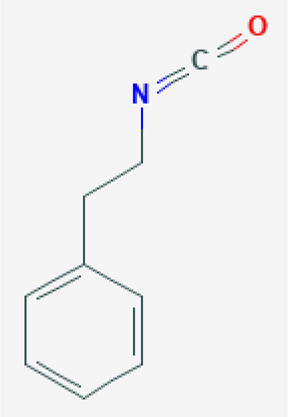

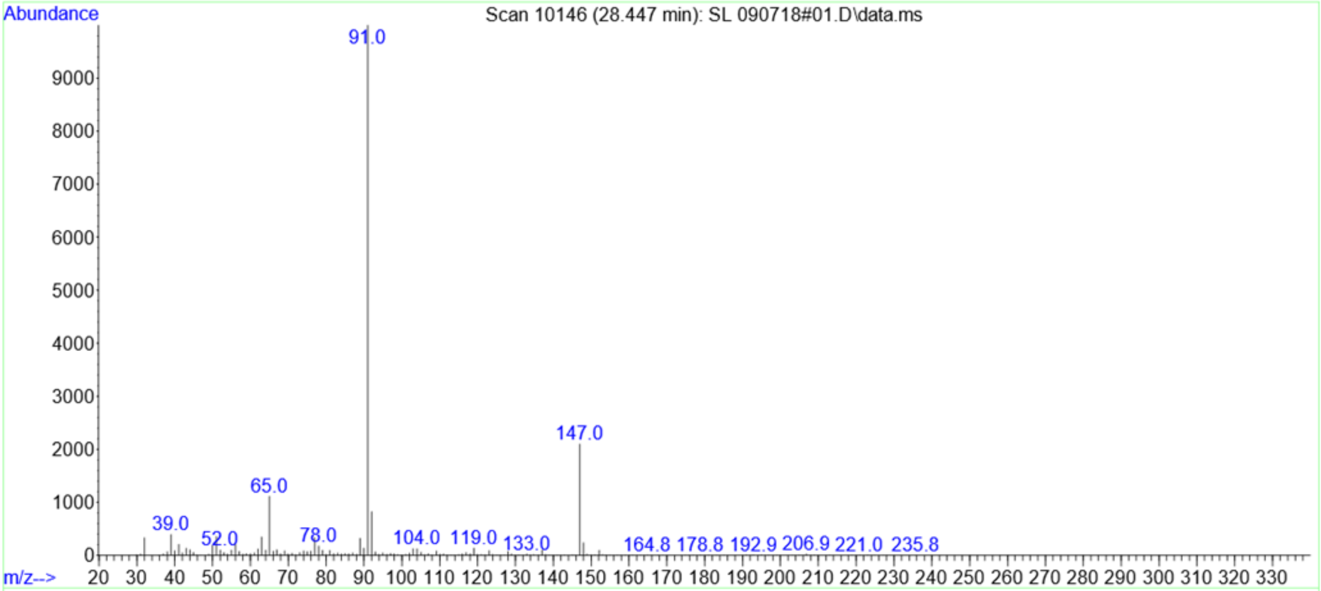

108. Quinoline

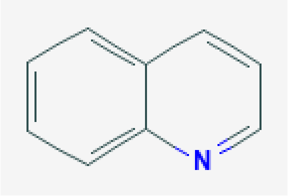

Library Searched : C:\Database\NIST11.L
Quality : 95
ID : Quinoline

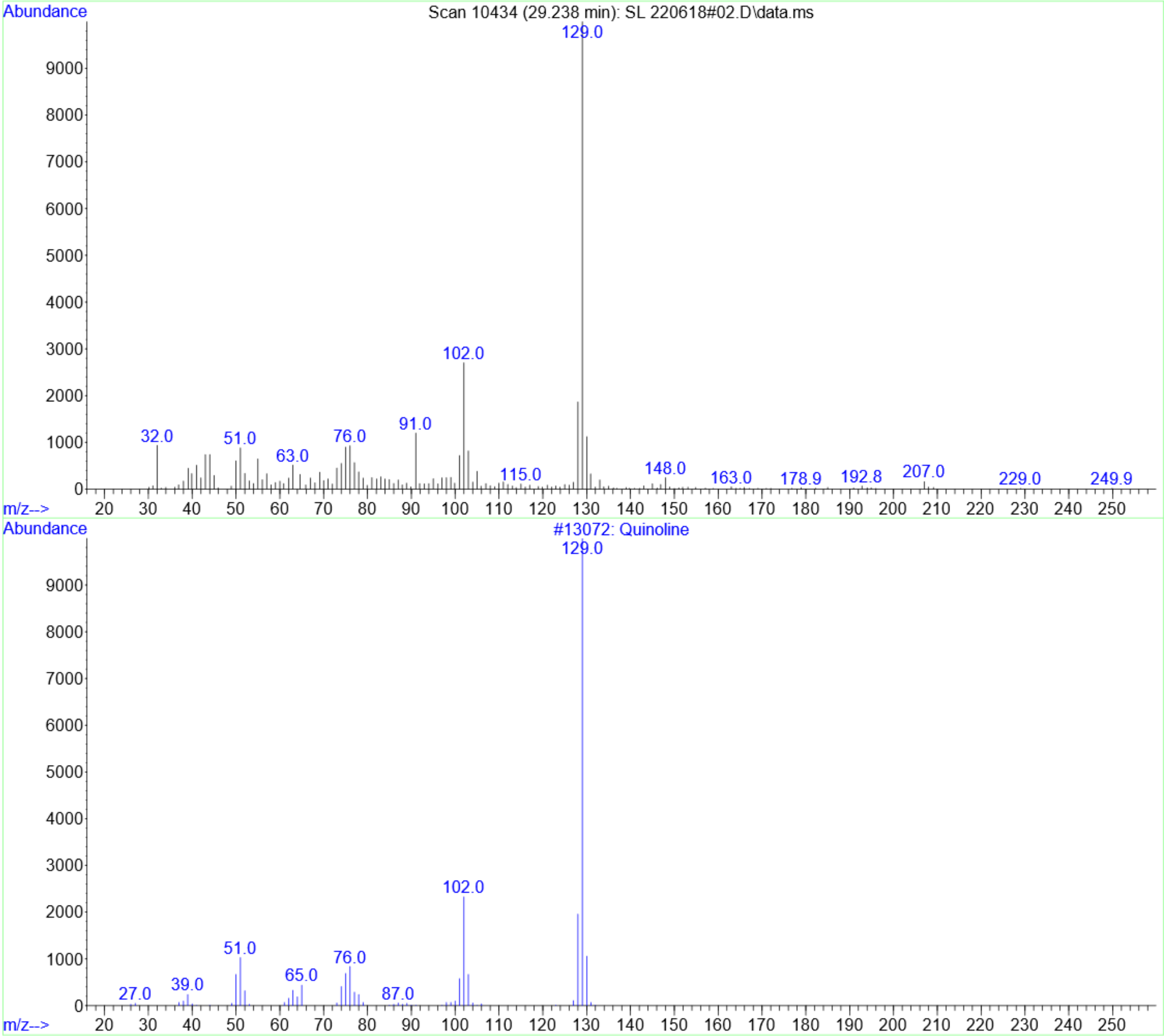

109. Caprolactam

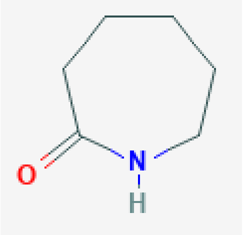

Library Searched : C:\Database\NIST11.L
Quality : 46
ID : Caprolactam

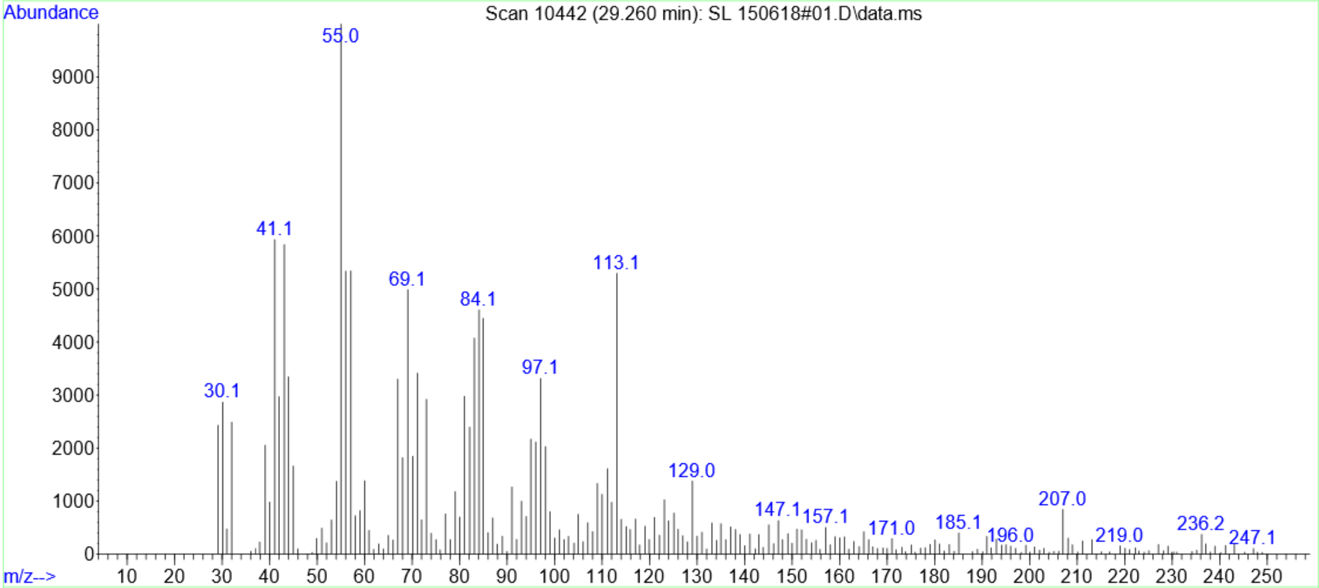

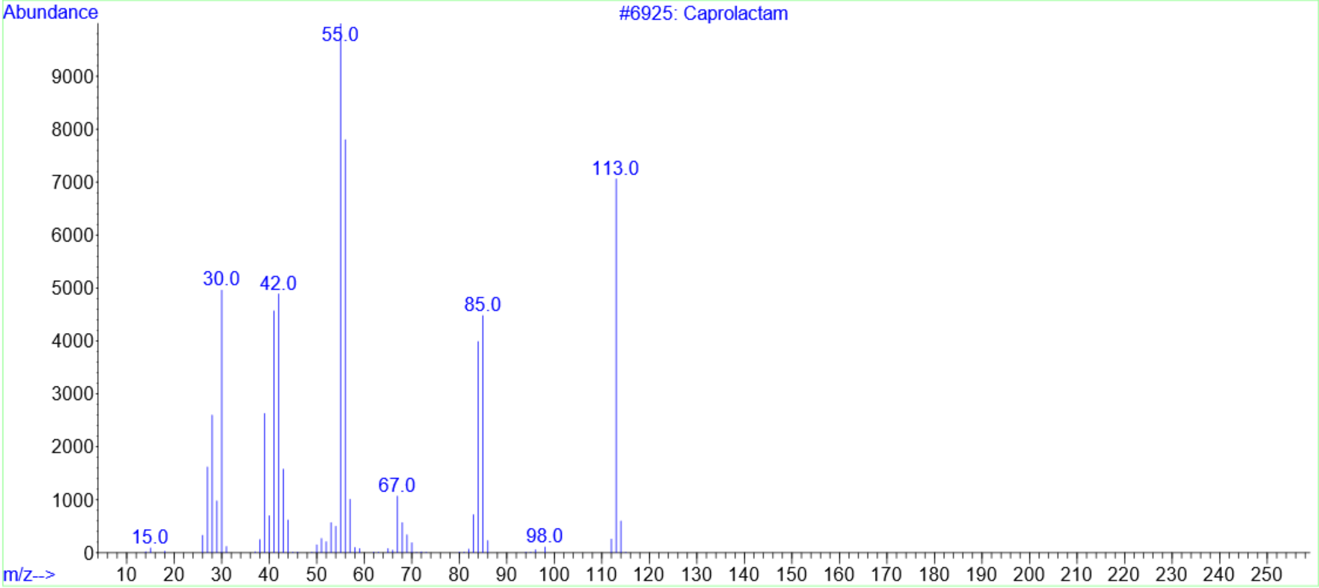

110. 4-bromophenol

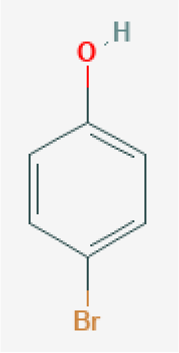

Library Searched : C:\Database\NIST11.L
Quality : 97
ID : Phenol, 4-bromo-

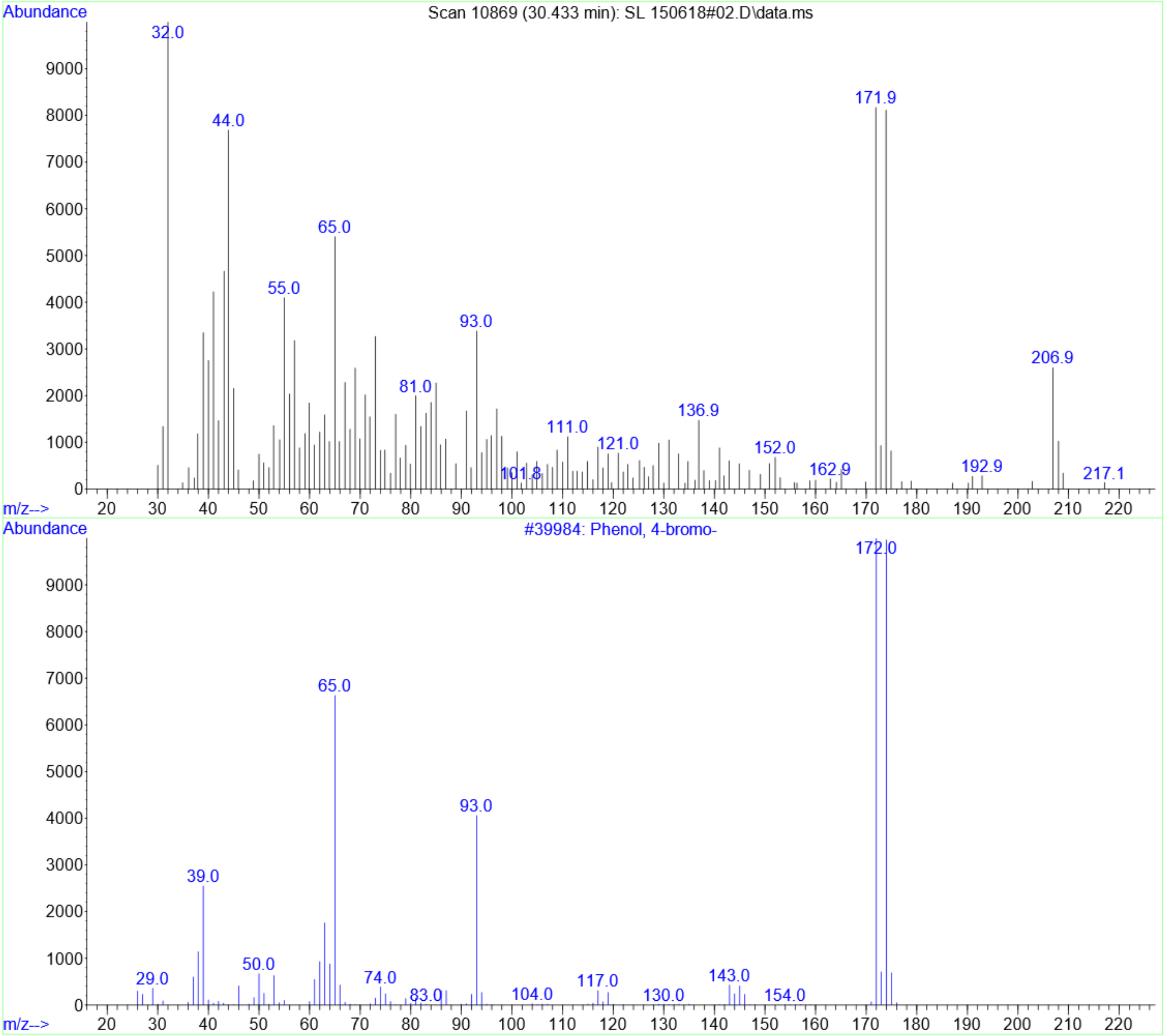

111. 6-methylquinoline

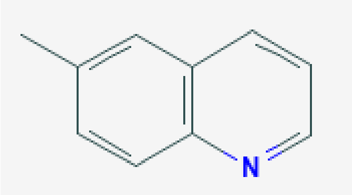

Library Searched : C:\Database\NIST11.L
Quality : 94
ID : Quinoline, 6-methyl-

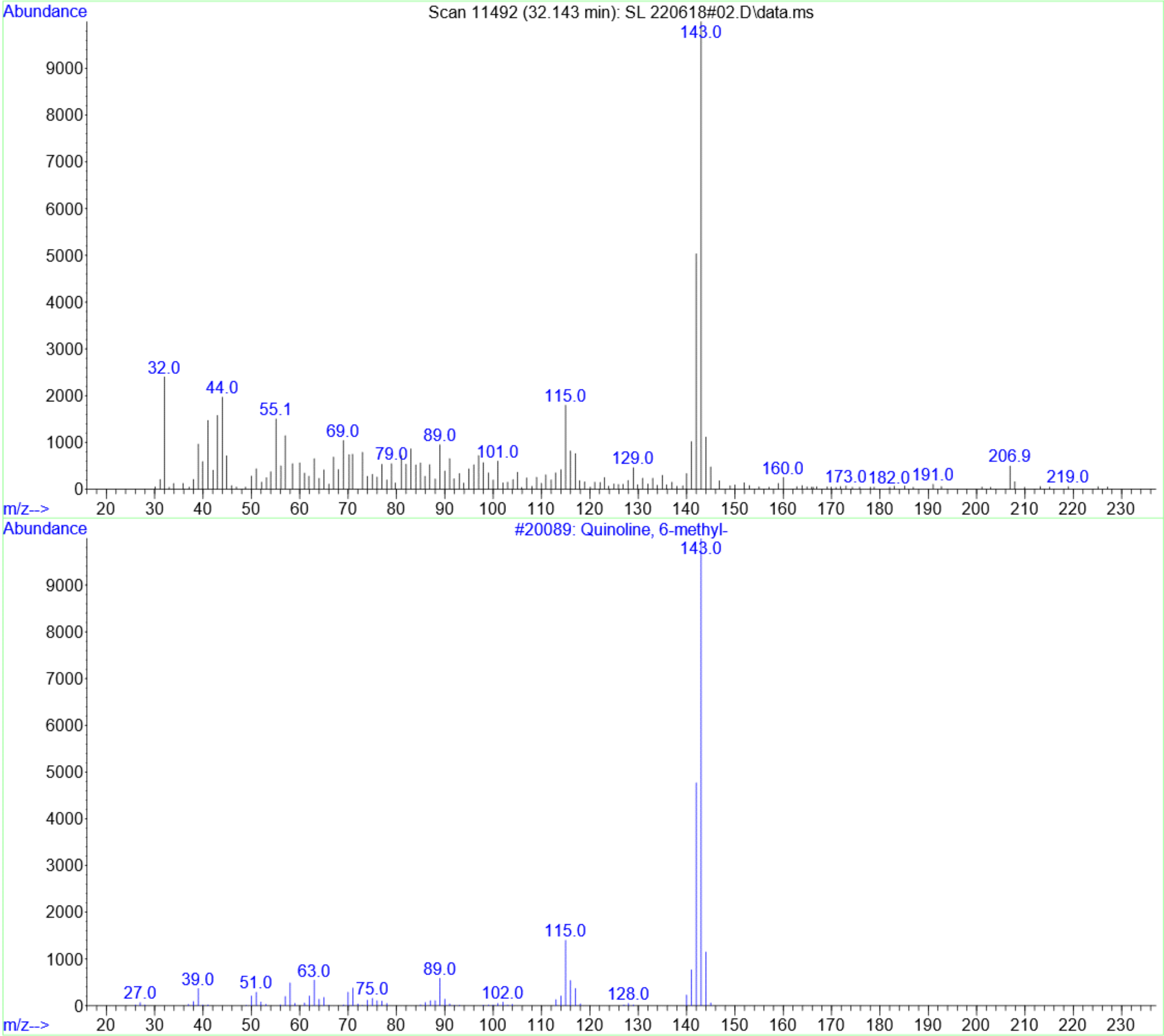

112. Methyleugenol

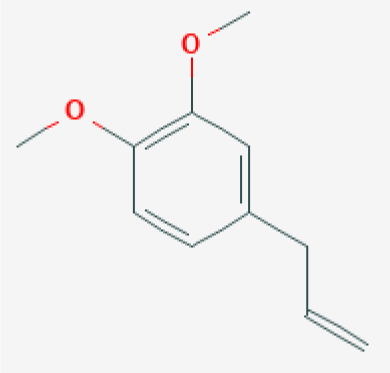

Library Searched : C:\Database\NIST11.L
Quality : 64
ID : Methyleugenol

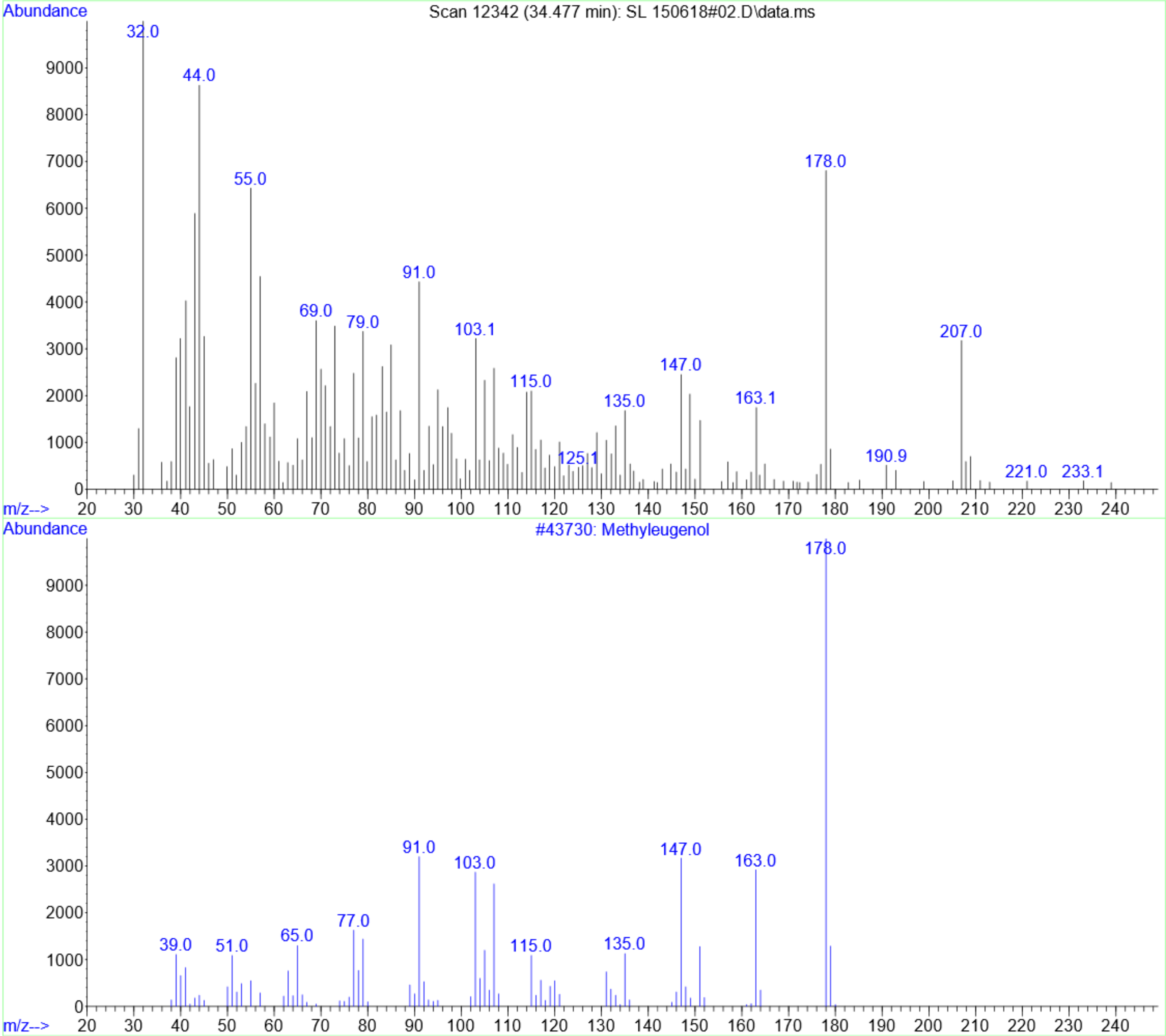

113. Benzyl tiglate

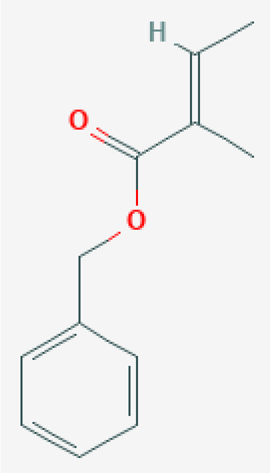

Library Searched : C:\Database\Adams.L
Quality : 64
ID : 28.71 Benzyl tiglate

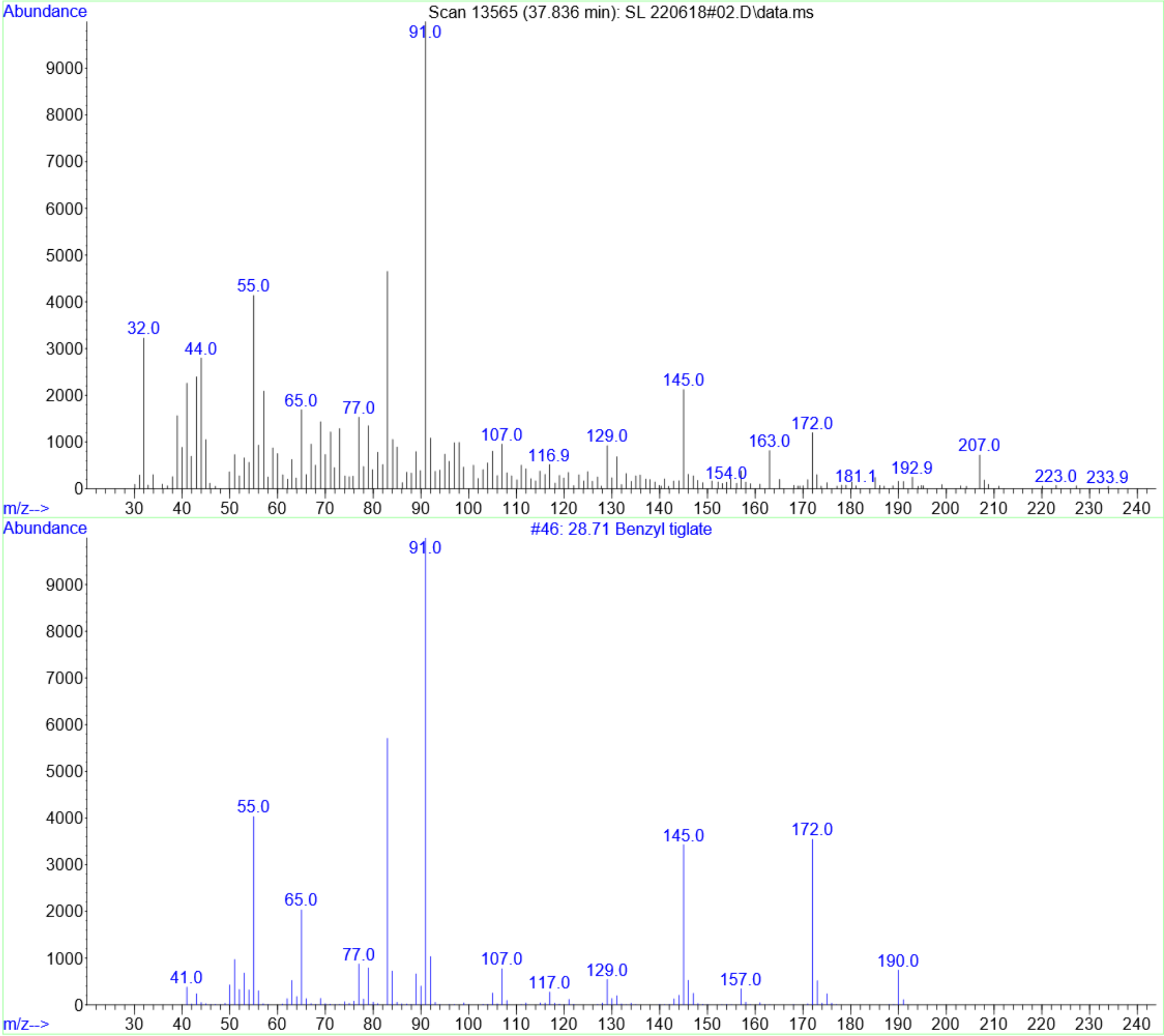

GC-MS chromatograms

Horseradish

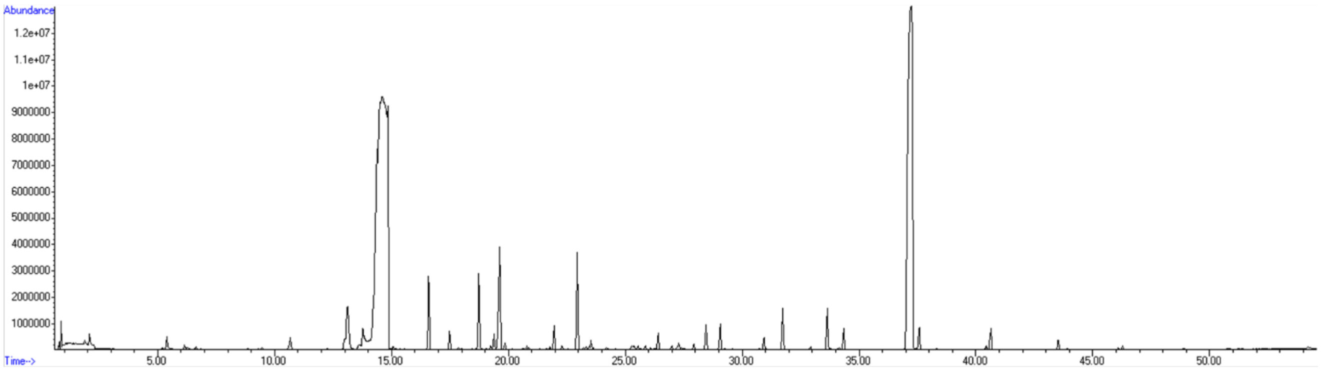

Rocket

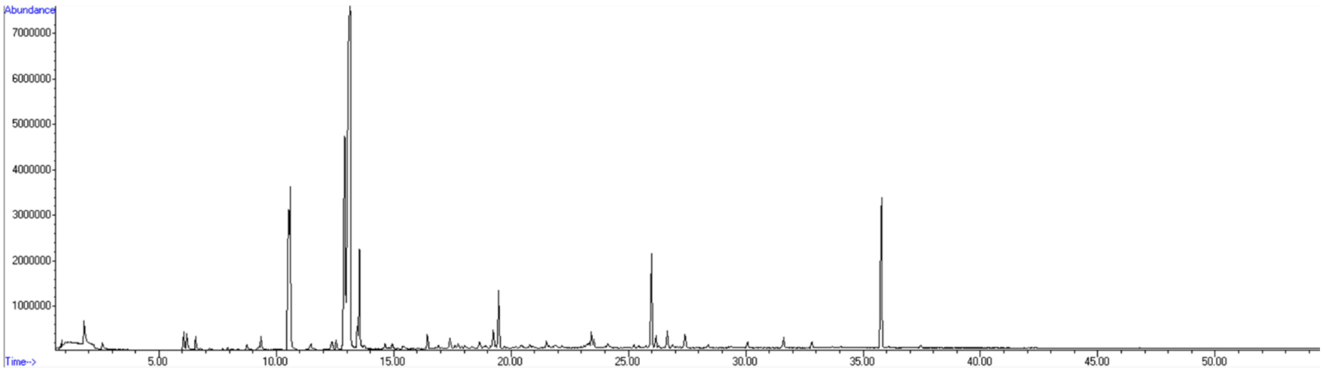

Wasabi

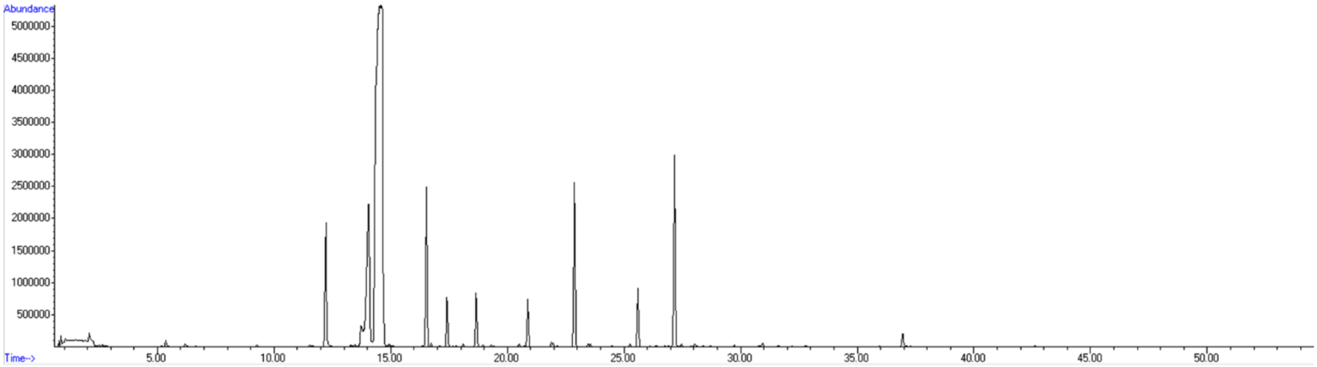

Watercress

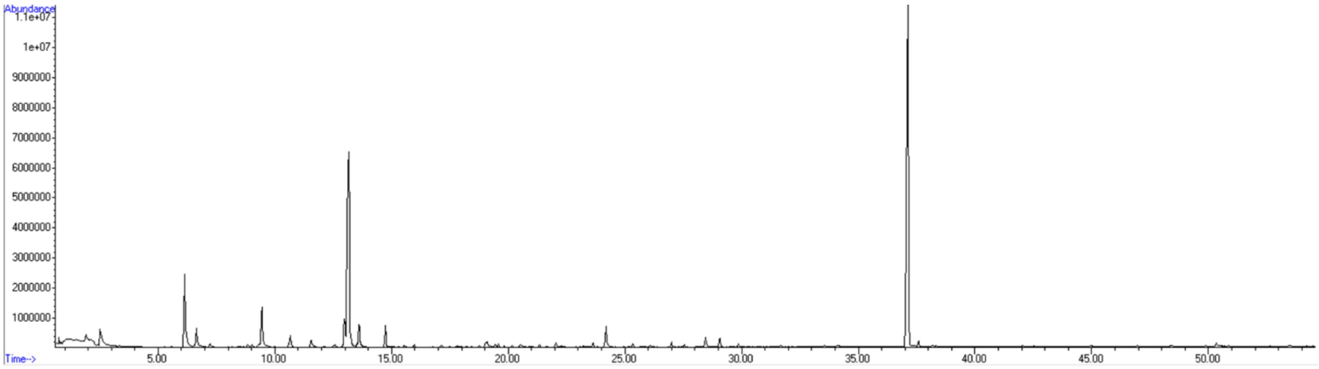

Supplementary data S2. Total Ion Chromatograms (TICs) of glucosinolates identified by UPLC-MS/MS.

Glucoiberin

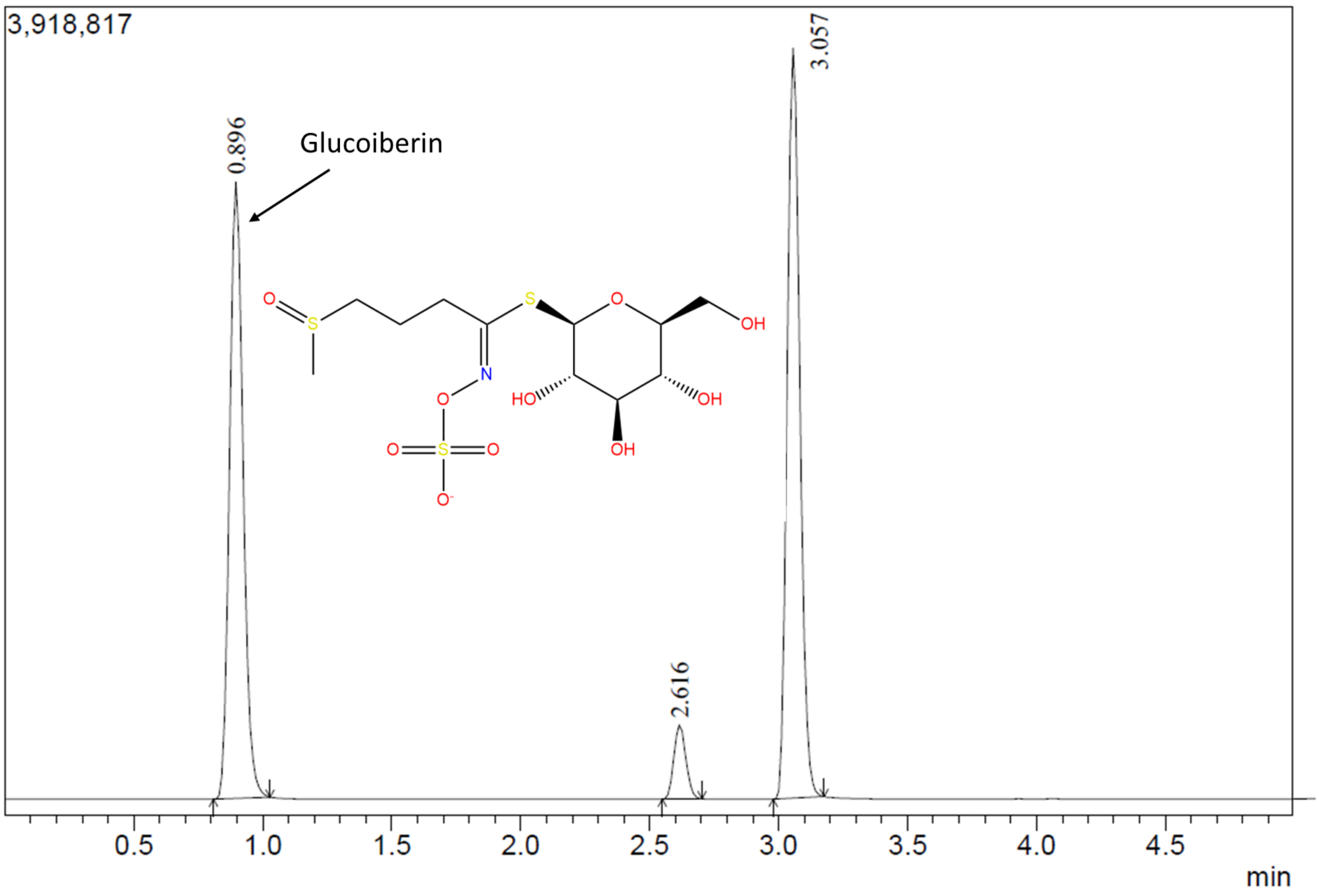

Pentyl GSL

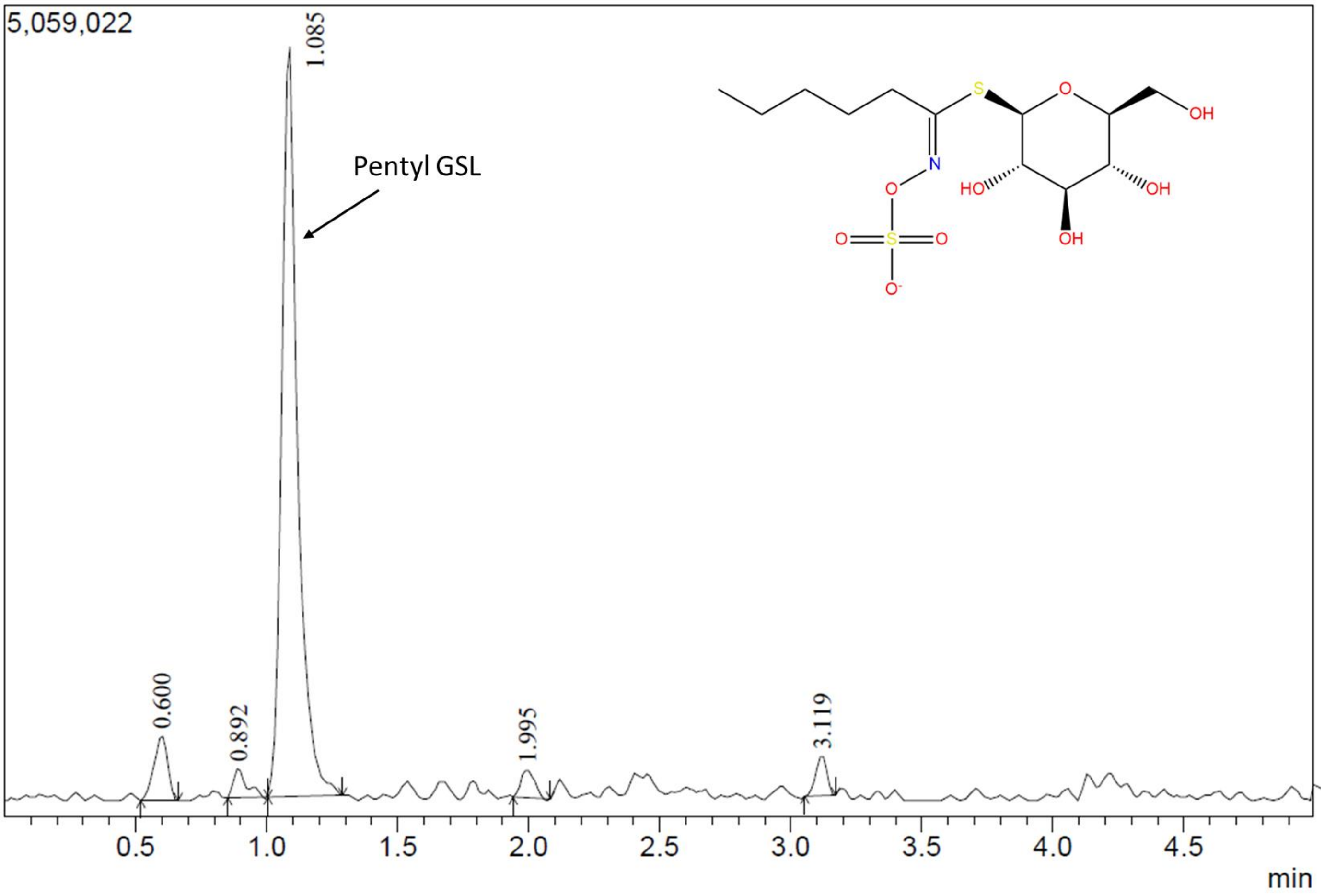

5,059,022
1.085
Pentyl GSL
0.600
0.892
1.995
3.119
0.5
1.0
1.5
2.0
2.5
3.0
3.5
4.0
4.5
min
S
O
OH
N
O
HO
OH
OH
O
S
O
O

Progoitrin

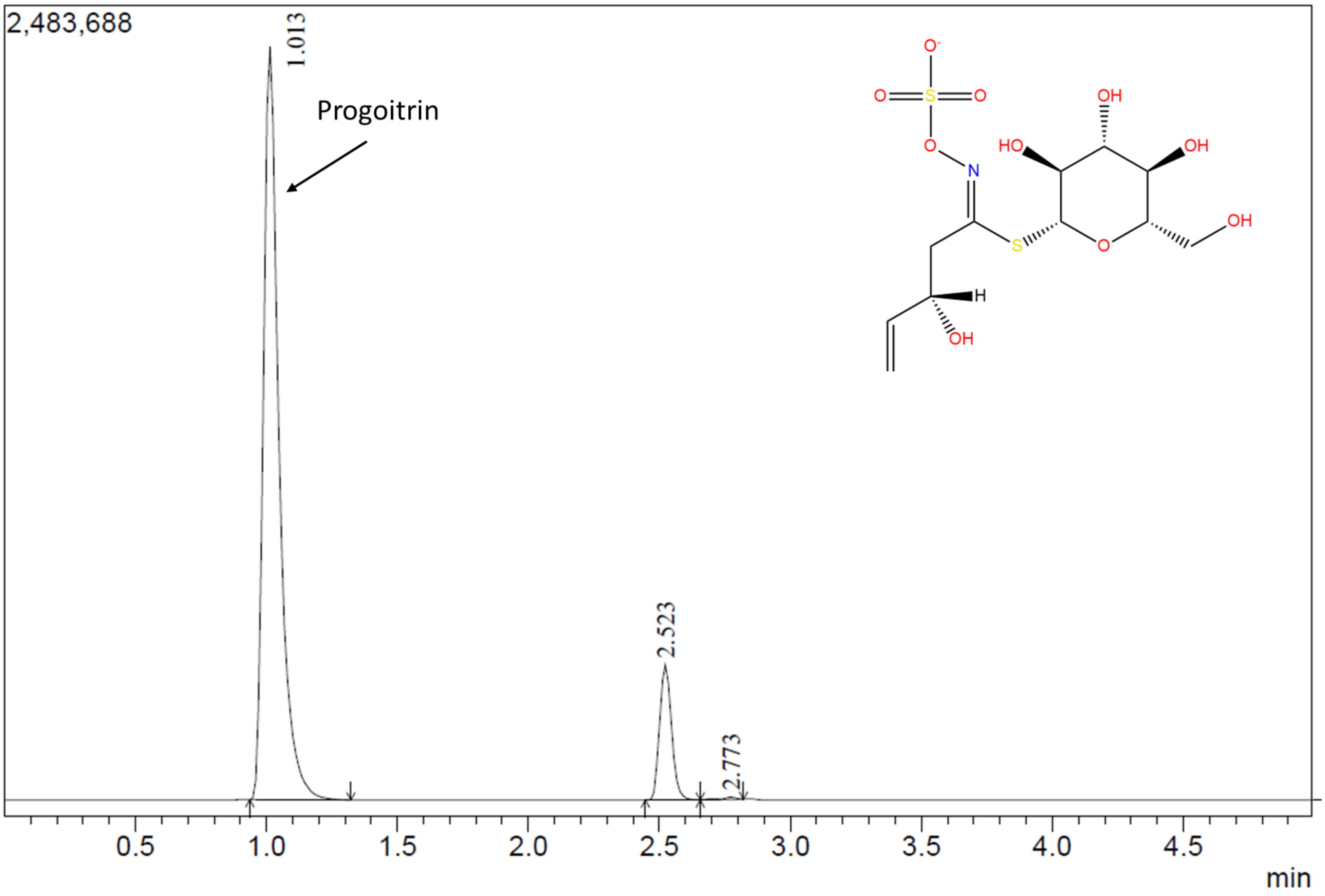
2,483,688
1.013
Progoitrin
2.523
2.773
0.5
1.0
1.5
2.0
2.5
3.0
3.5
4.0
4.5
min
O-
O
S
O
O
N
OH
HO
OH
OH
S
O
H
OH

Sinigrin

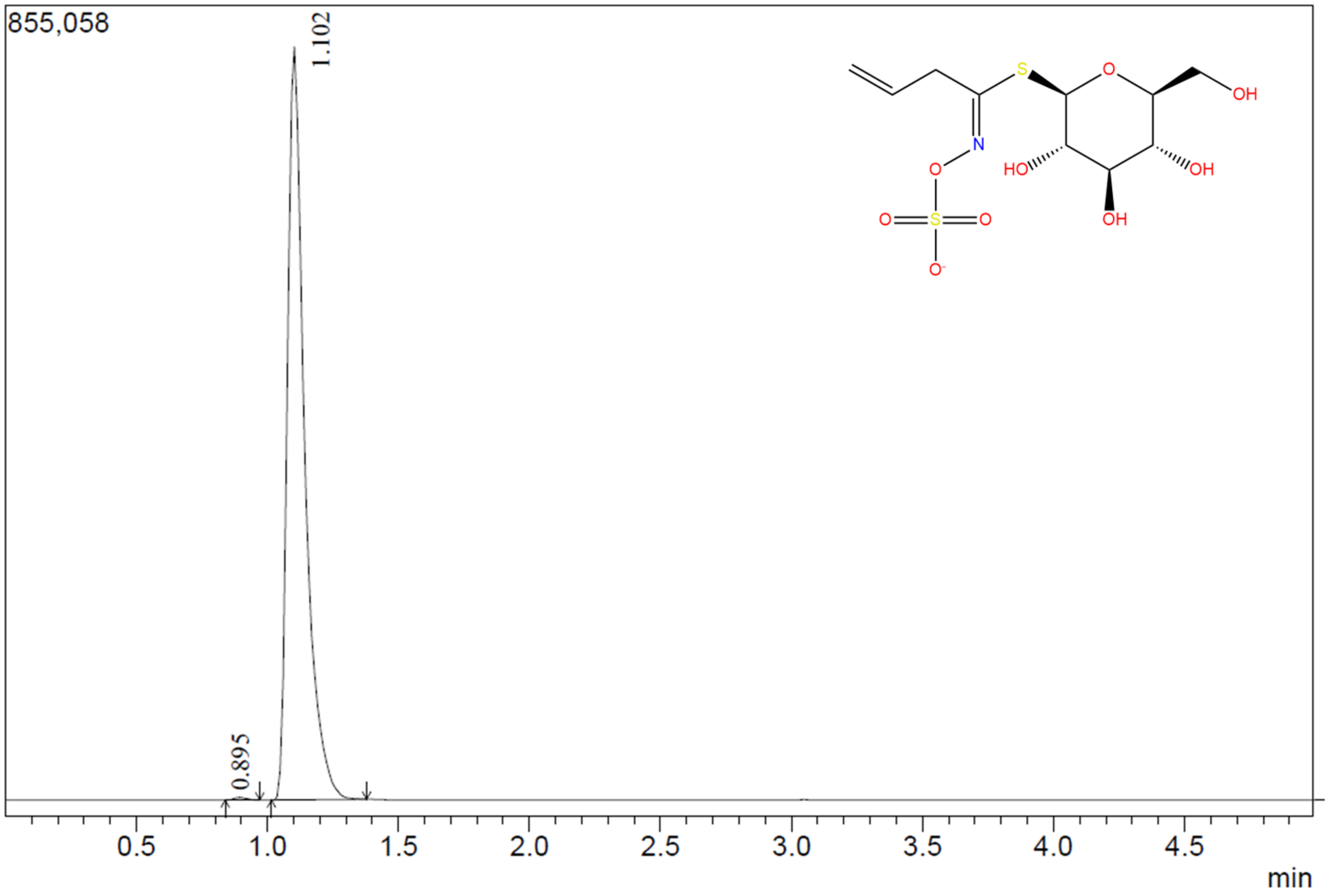

855,058
1.102
0.895
0.5
1.0
1.5
2.0
2.5
3.0
3.5
4.0
4.5
min
S
O
OH
N
HO
OH
O
OH
O
S
O
O

Isobutyl GSL

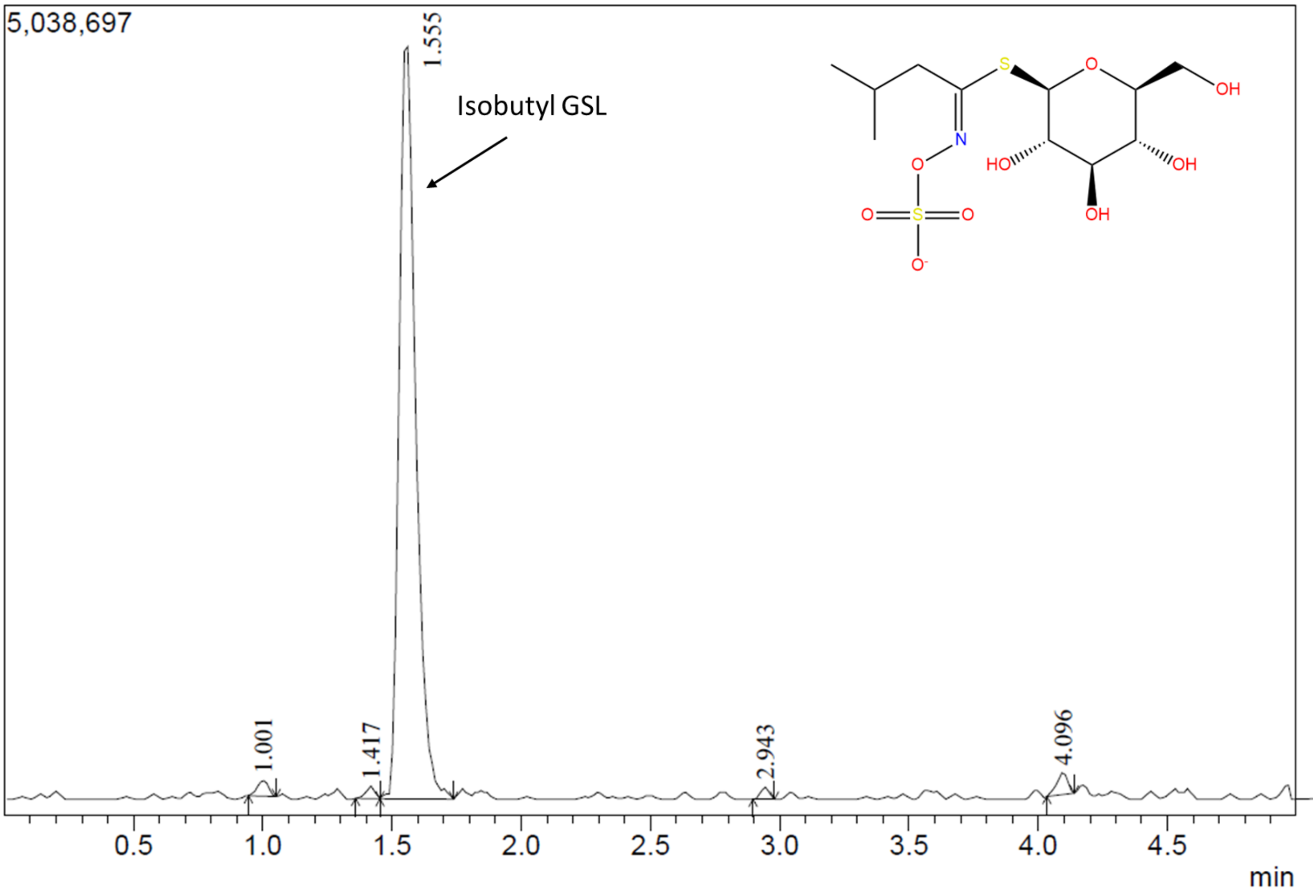

Glucoraphanin

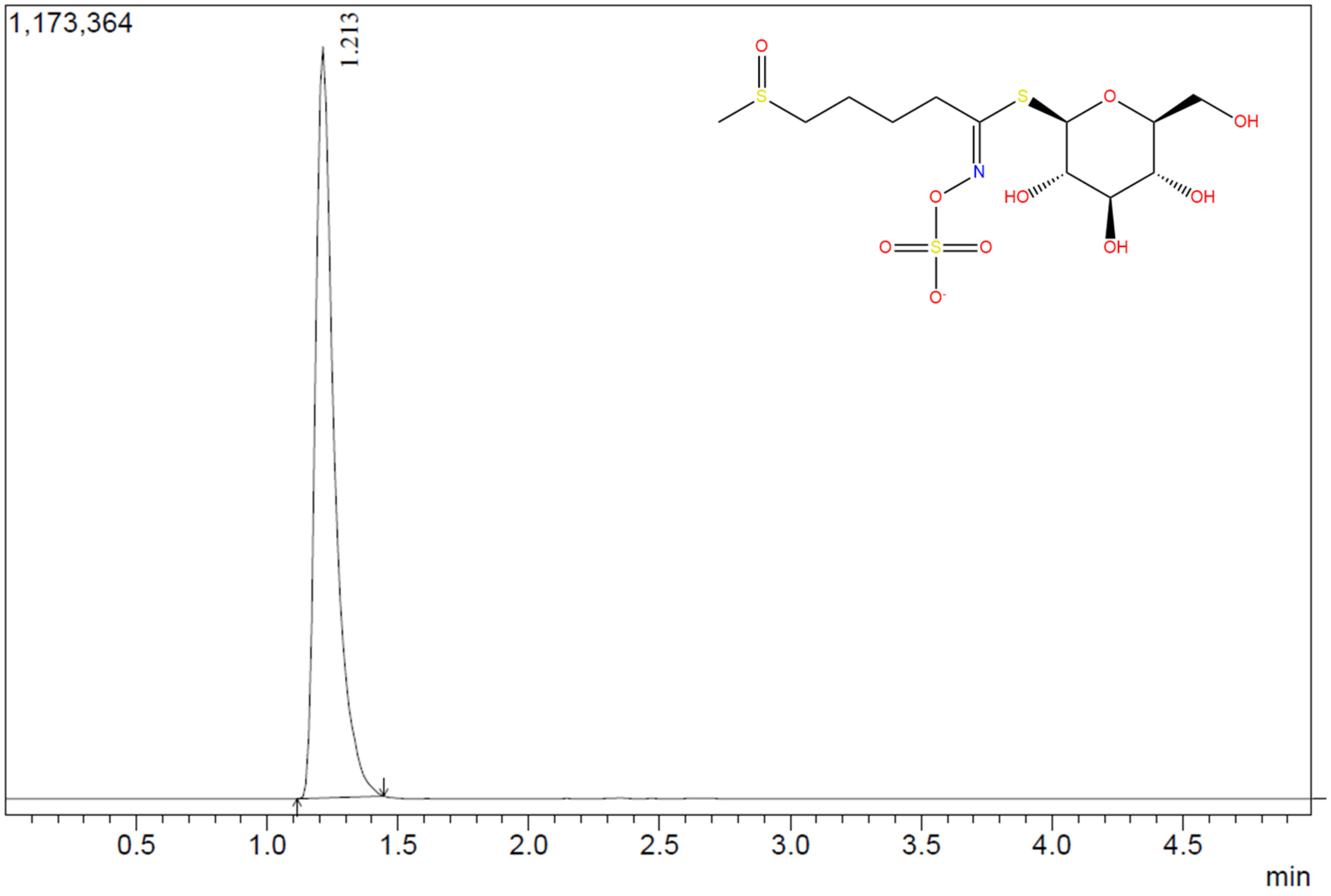

Glucorucolamine

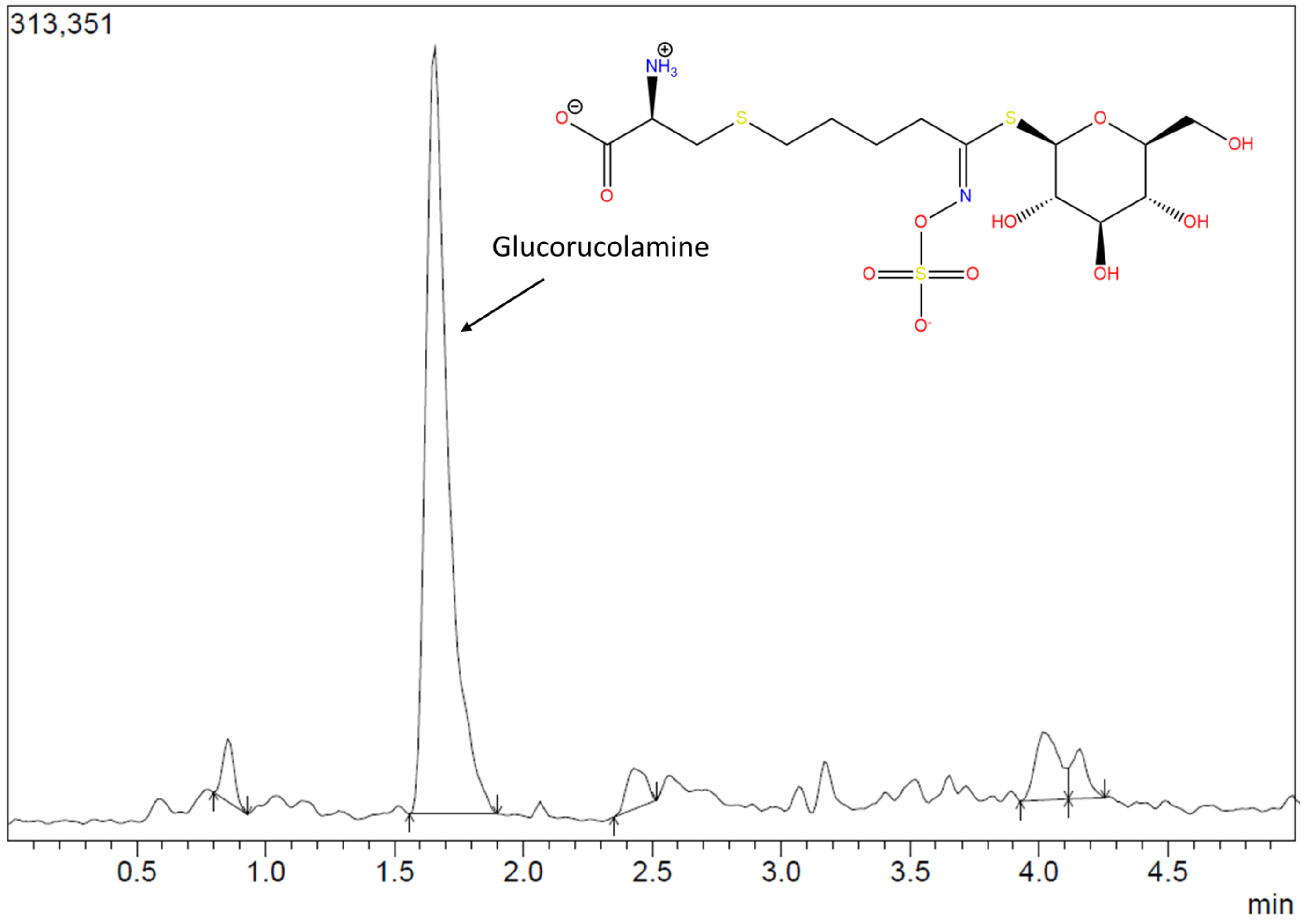

Glucoalyssin

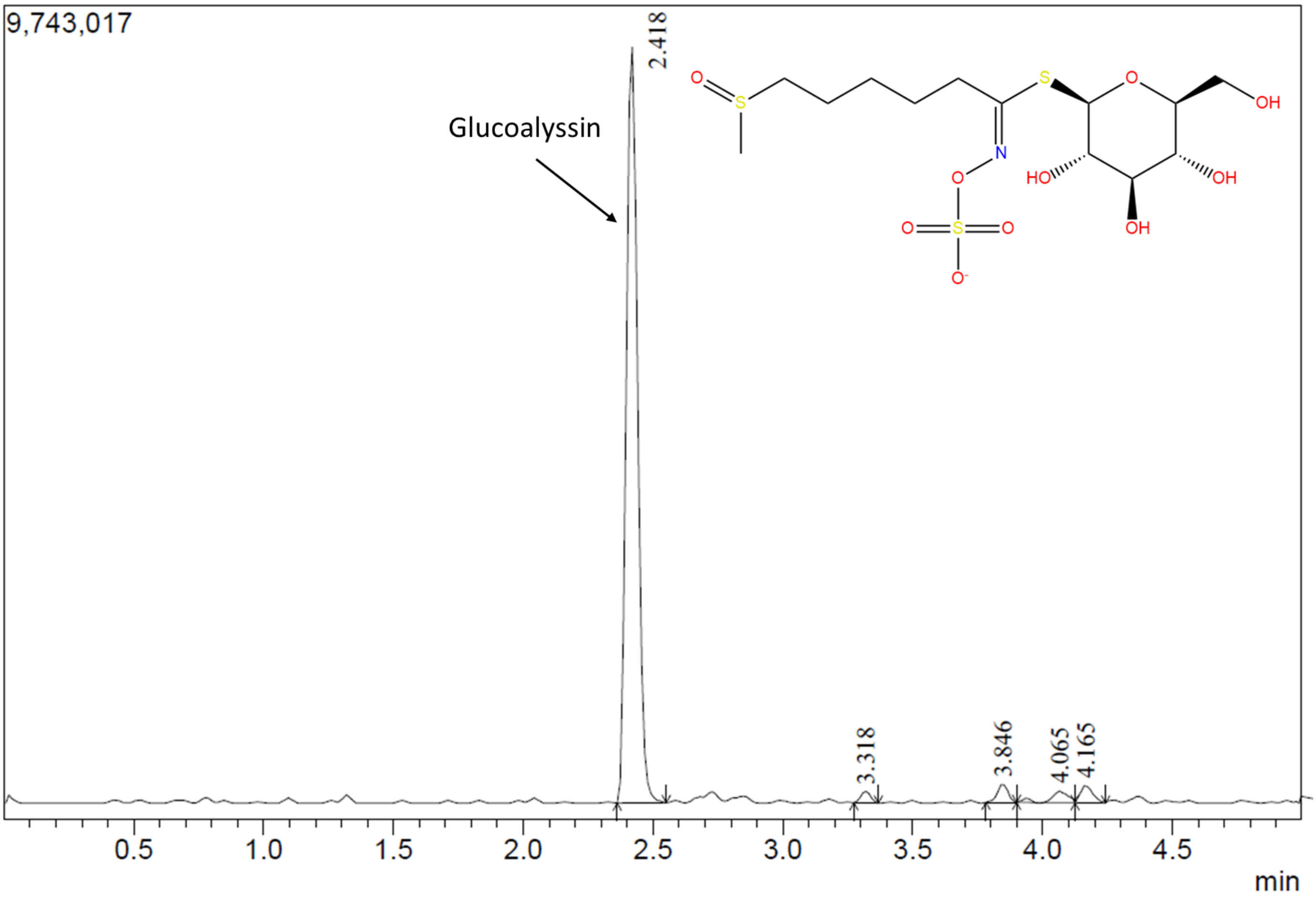

Glucoputranjivin

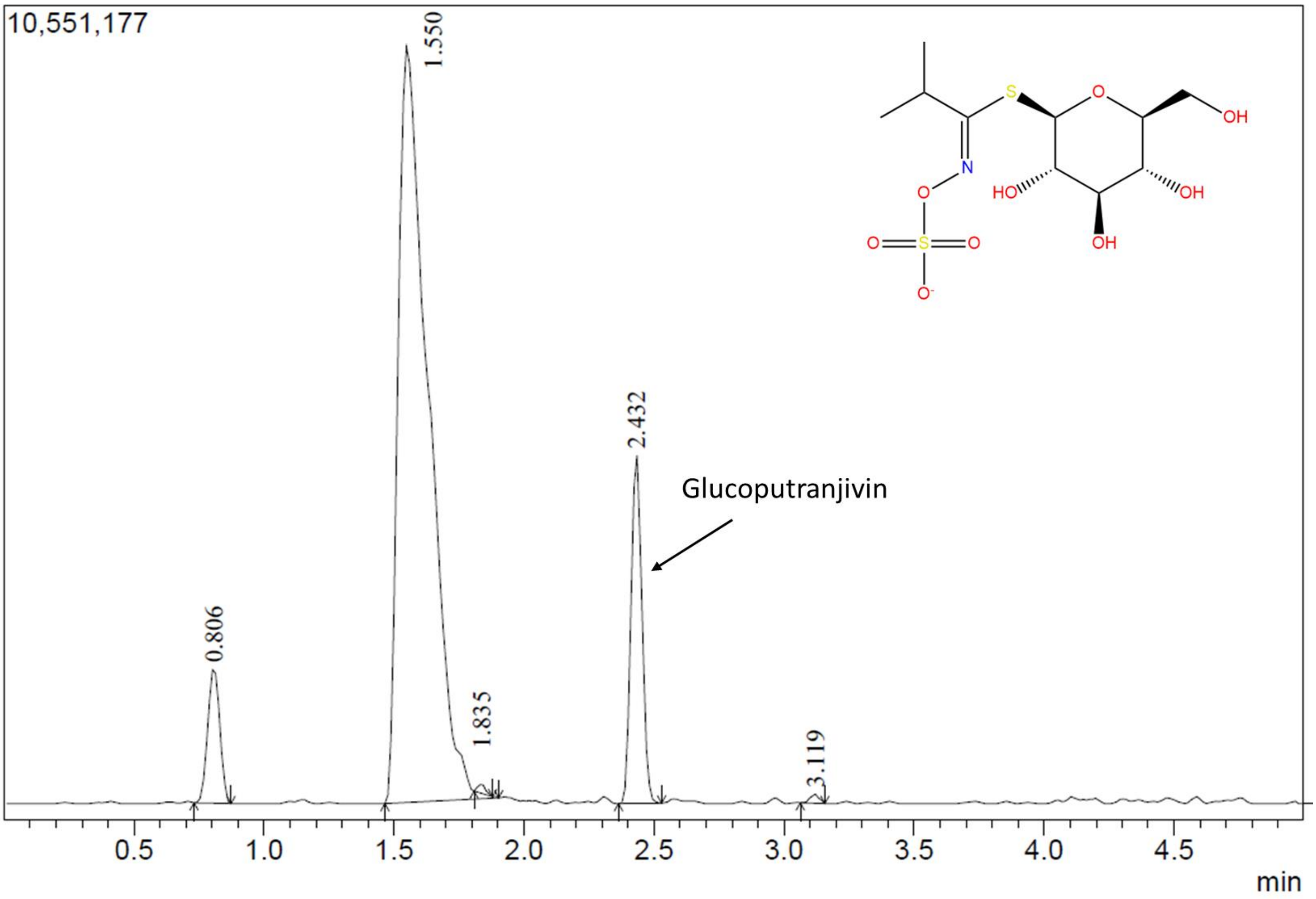

Gluconapin

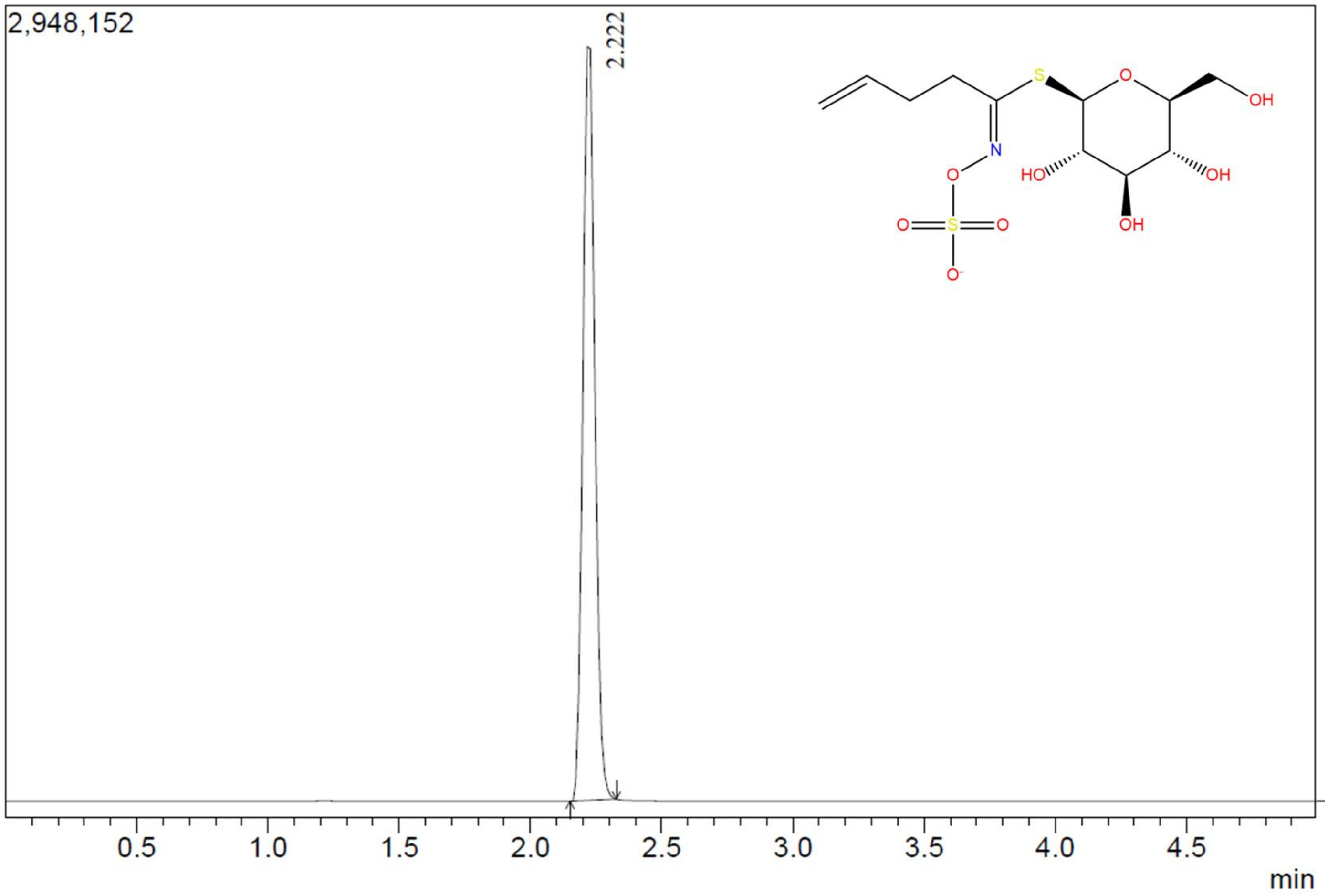

Diglucothiobeinin

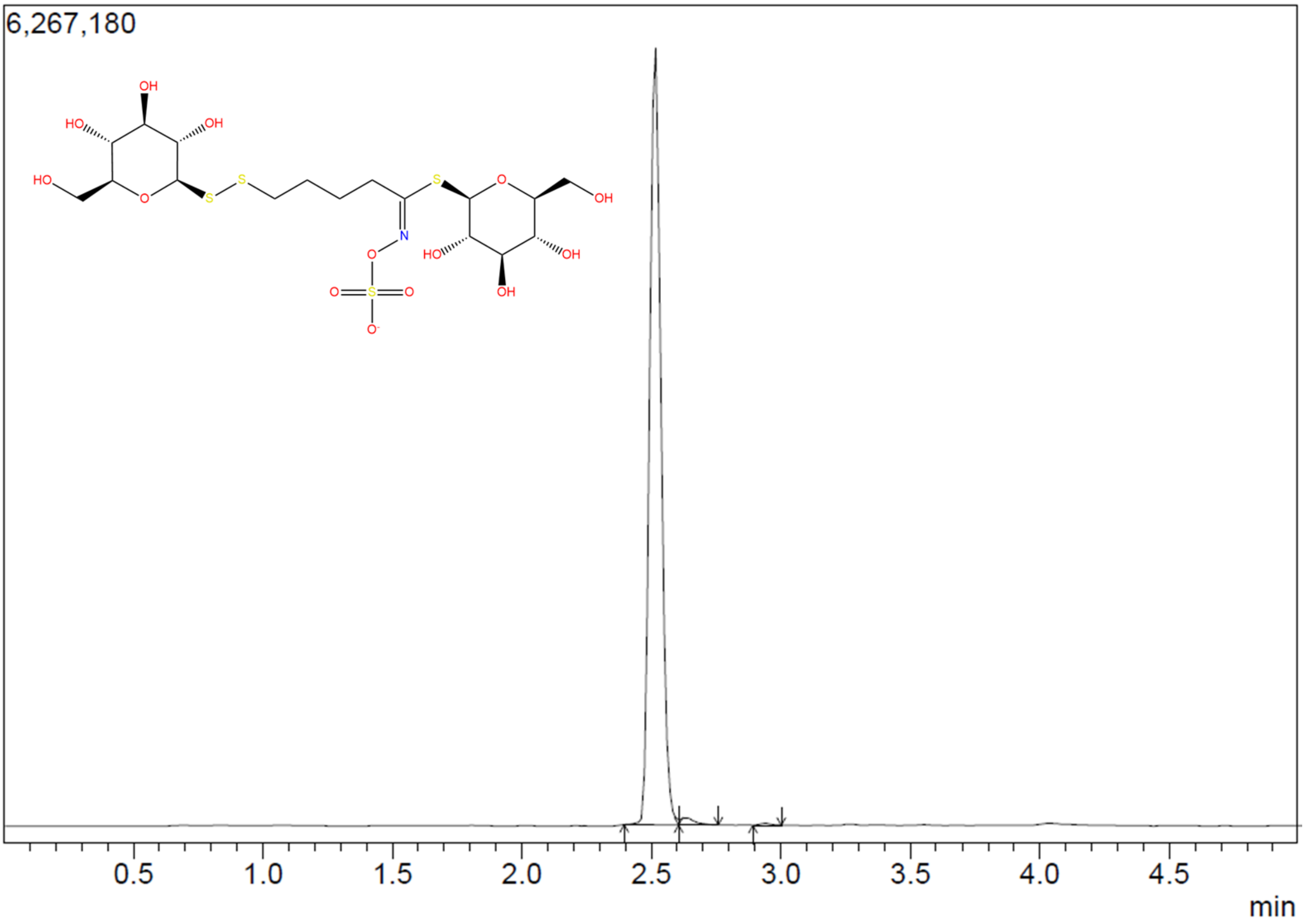

Glucoberteroin

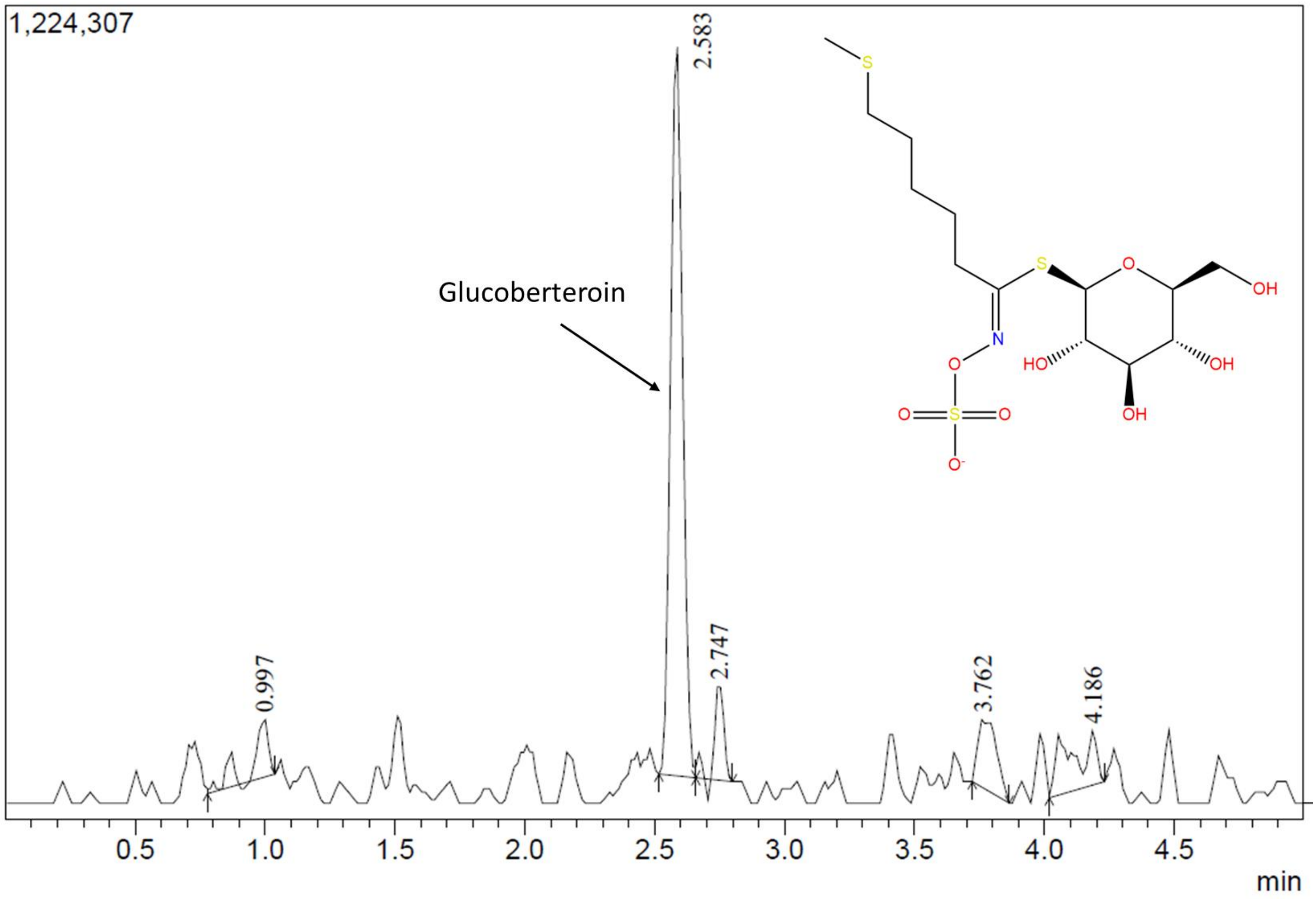

4-hydroxyglucobrassicin

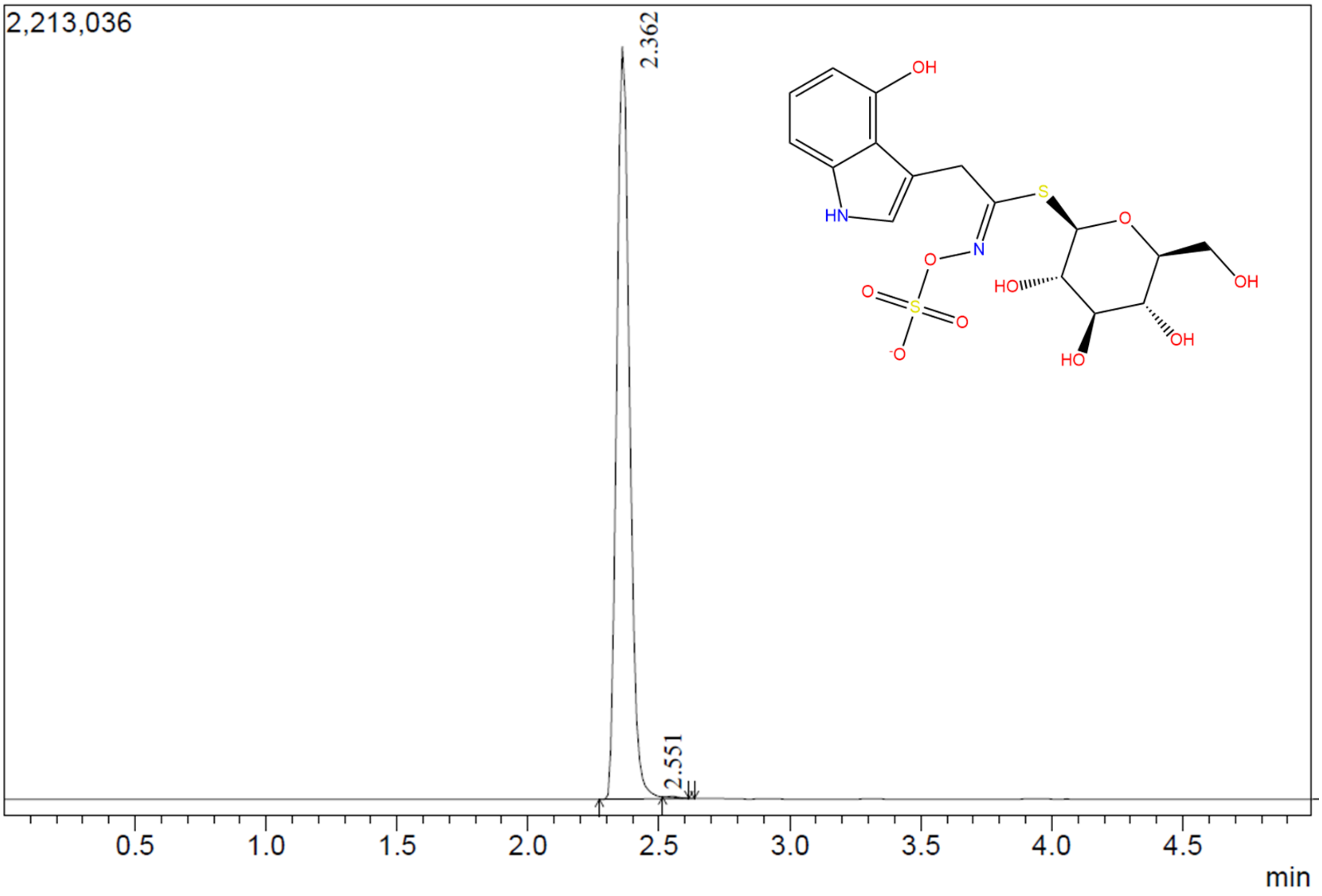

Glucocochlearin

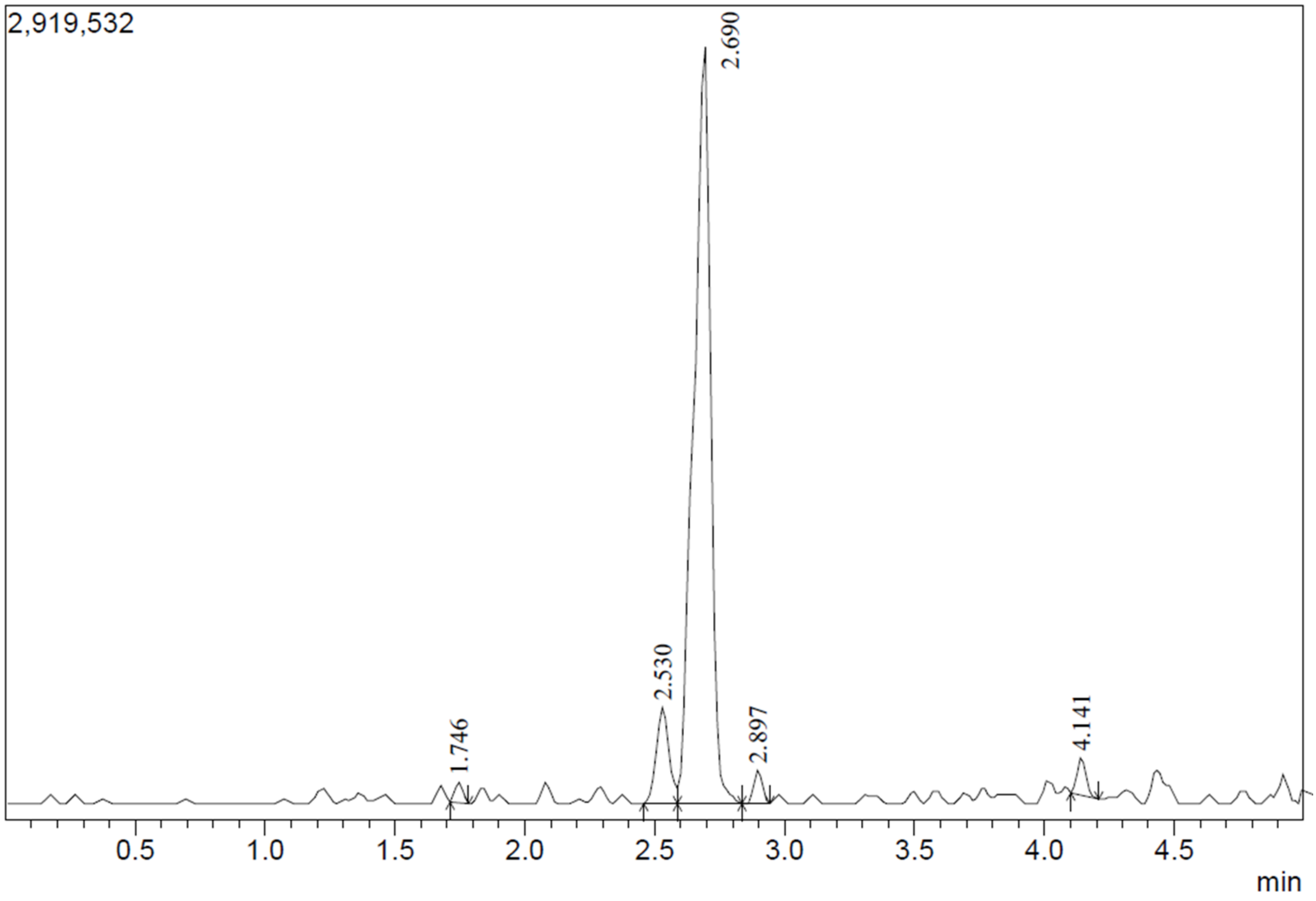

Glucosativin & Dimeric 4-mercaptobutyl GSL

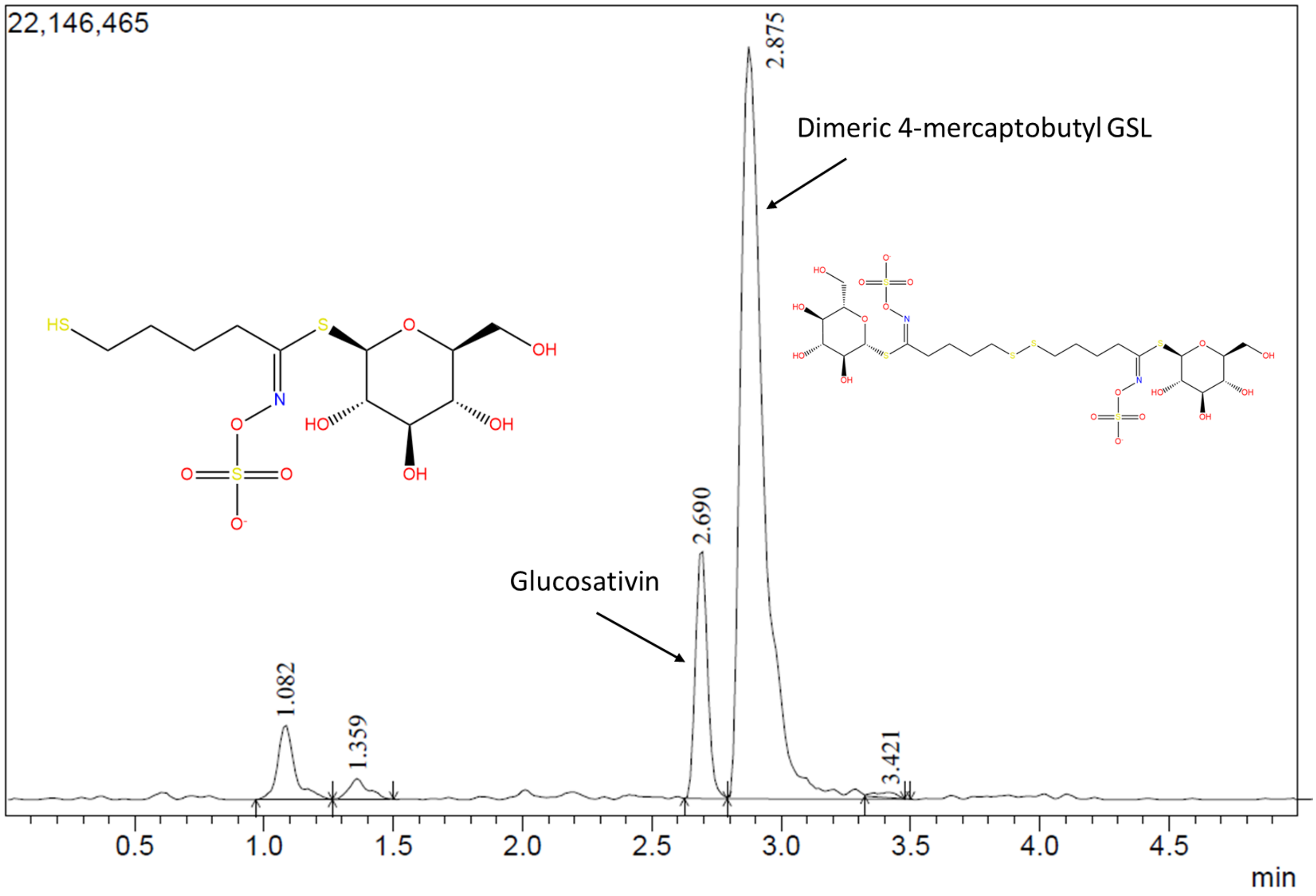

7-methylsulfinylheptyl GSL

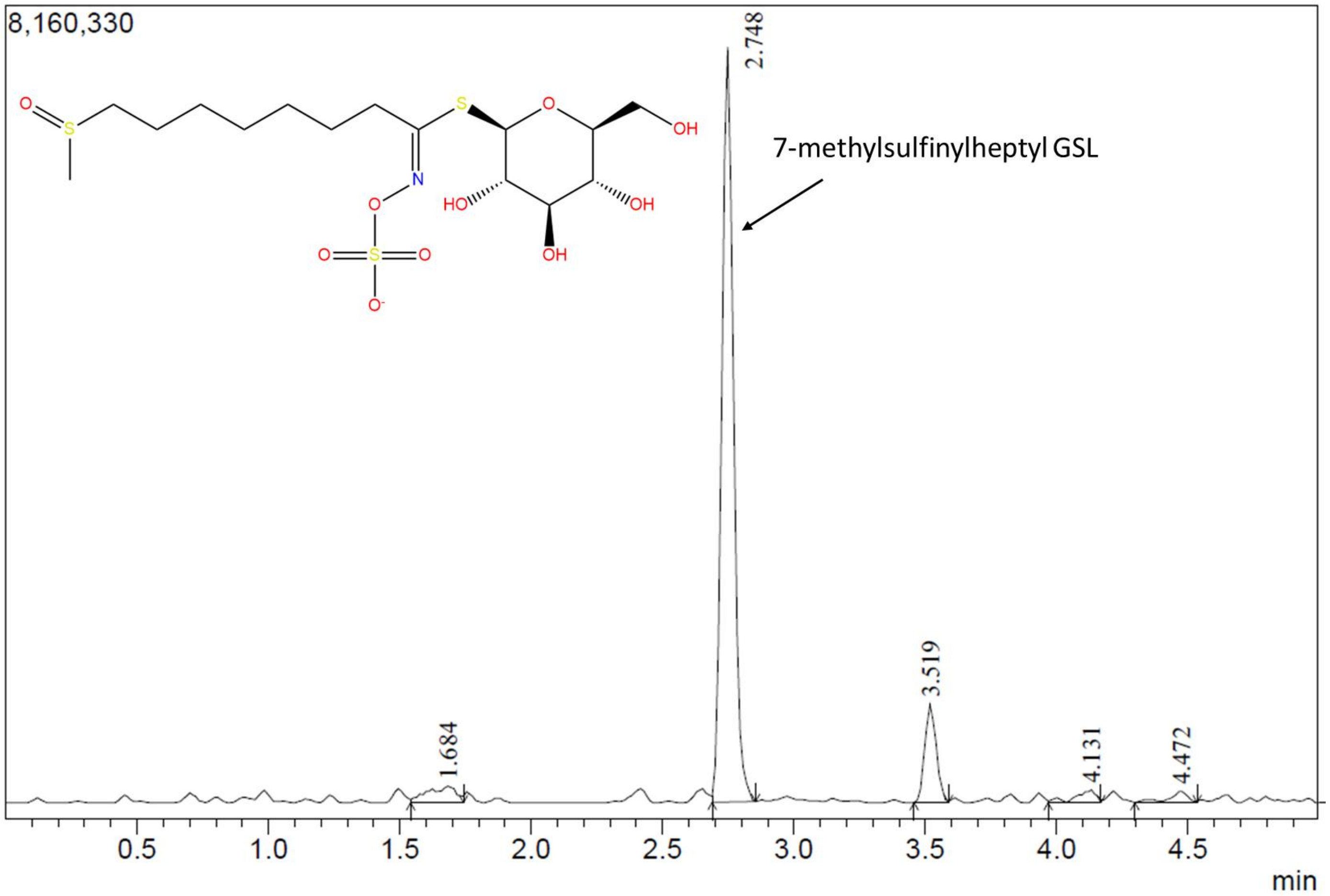

Glucobrassicanapin

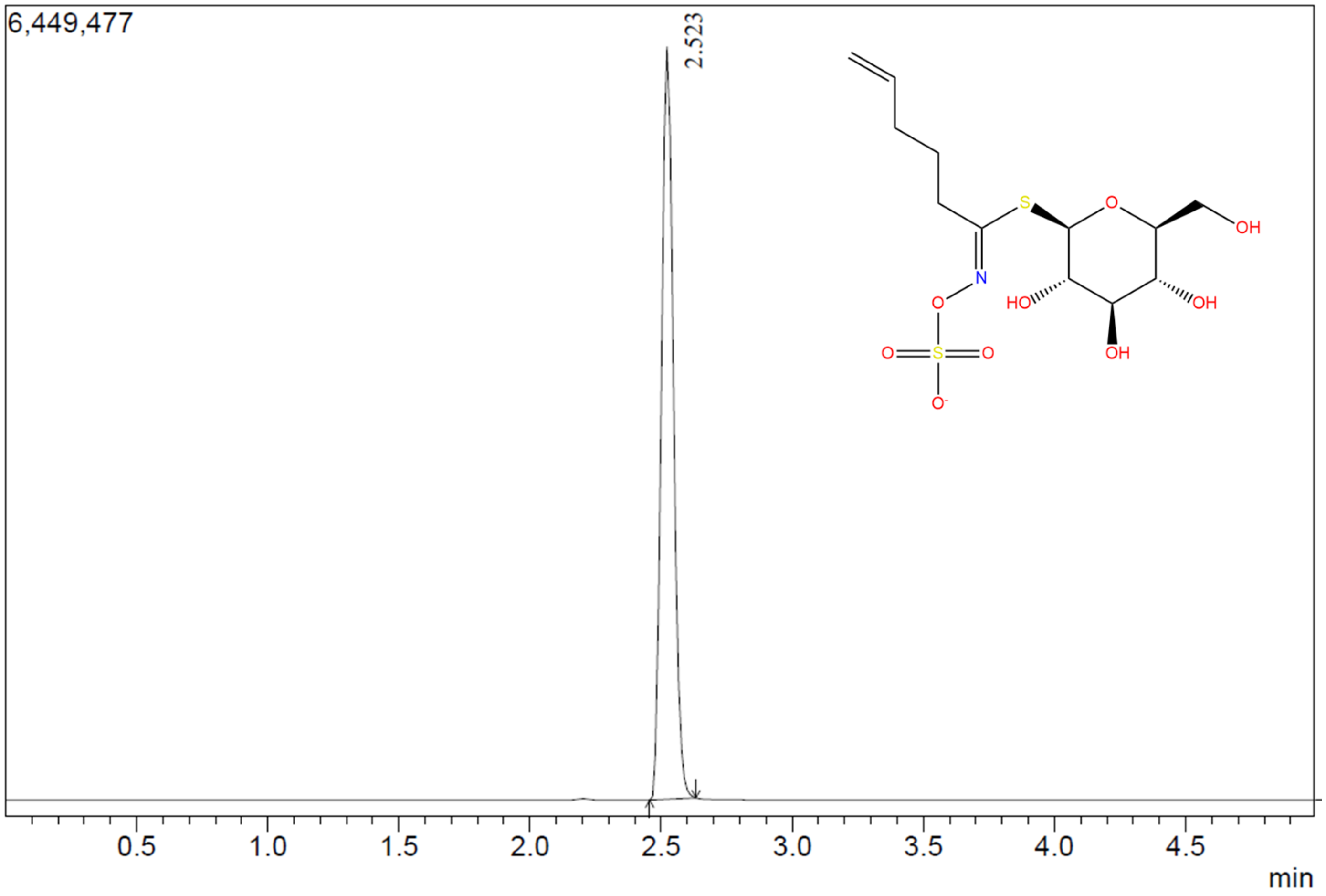

Glucobarbarin

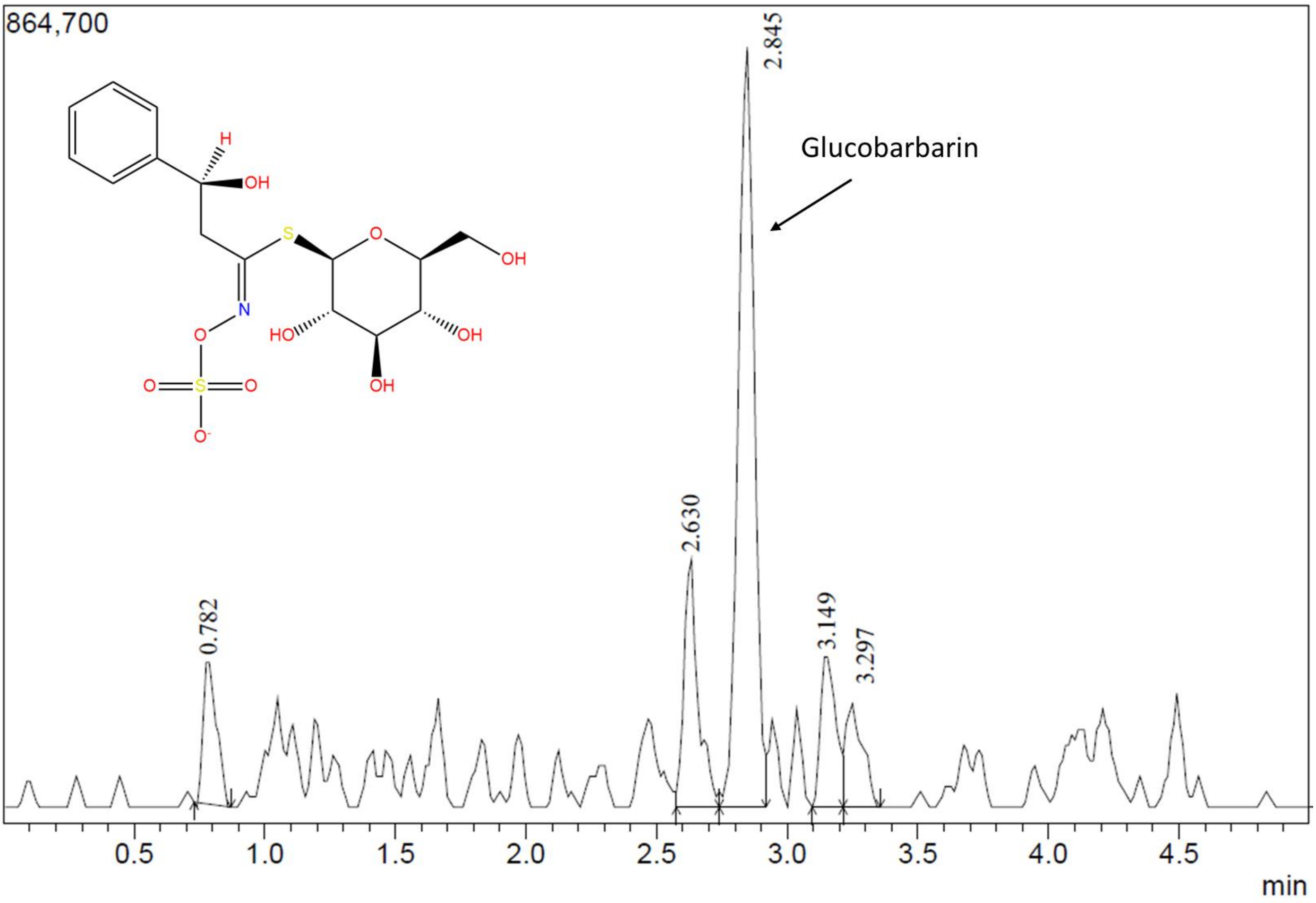

Glucotropaeolin

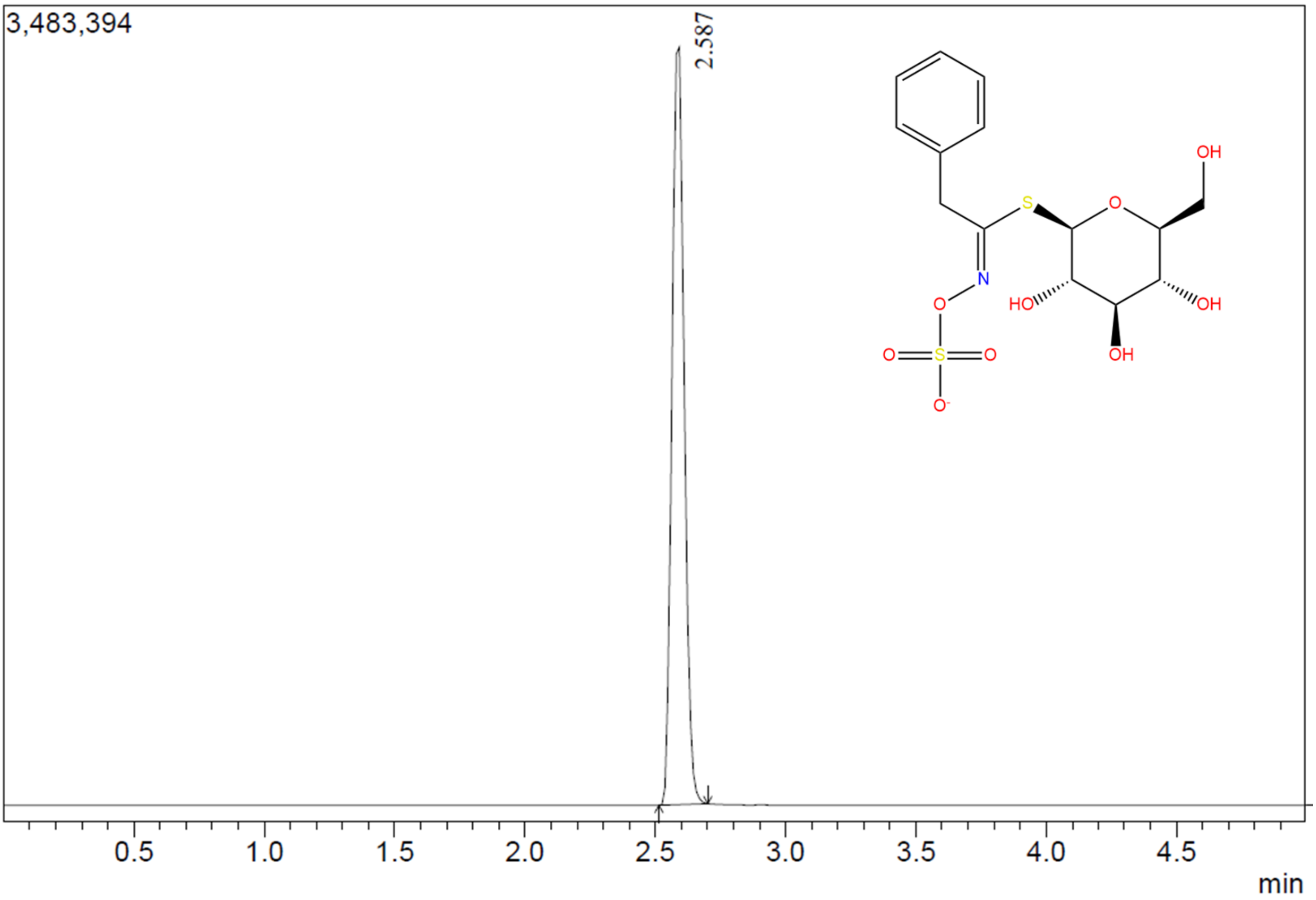

Glucoerucin

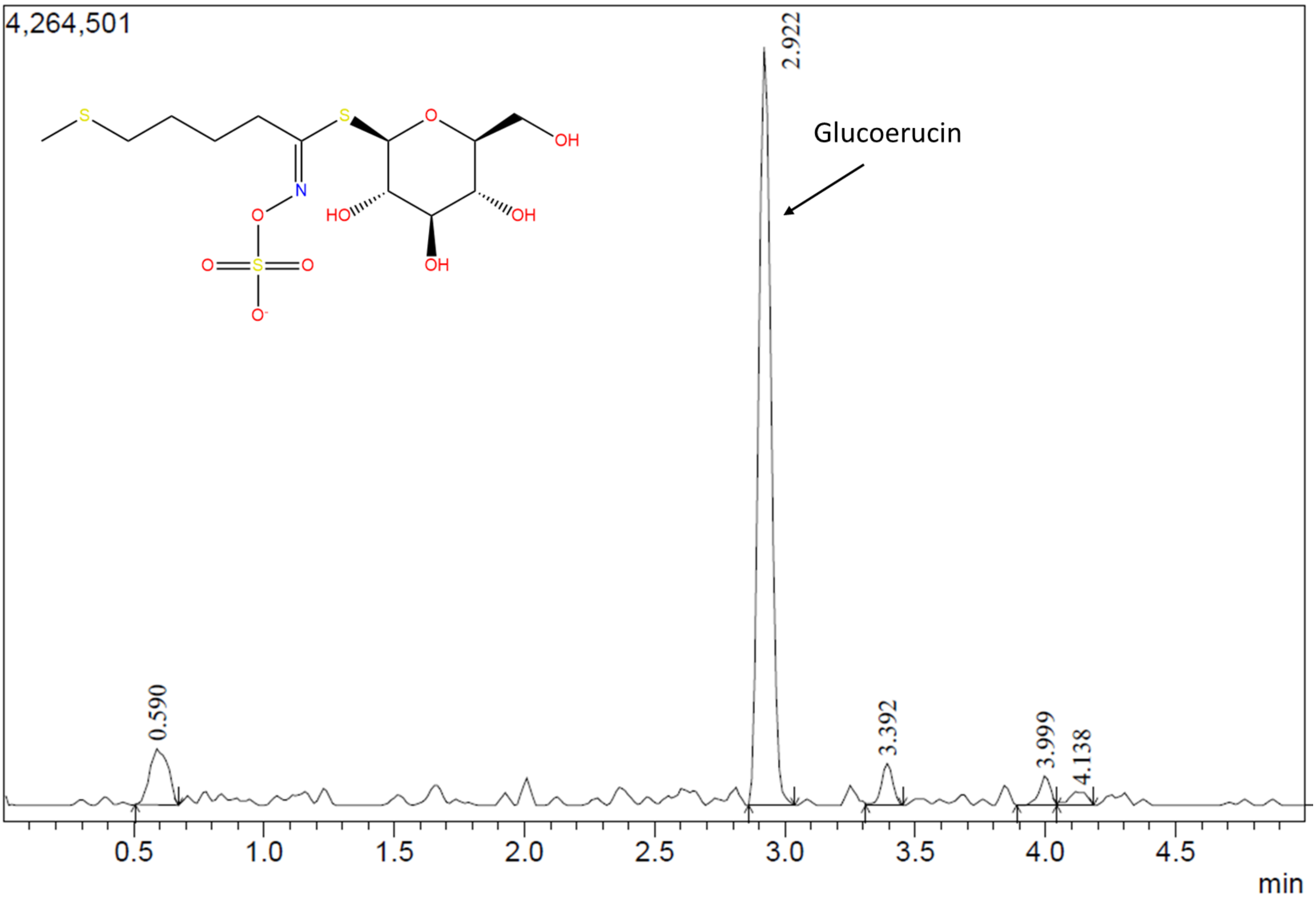

Glucobrassicin

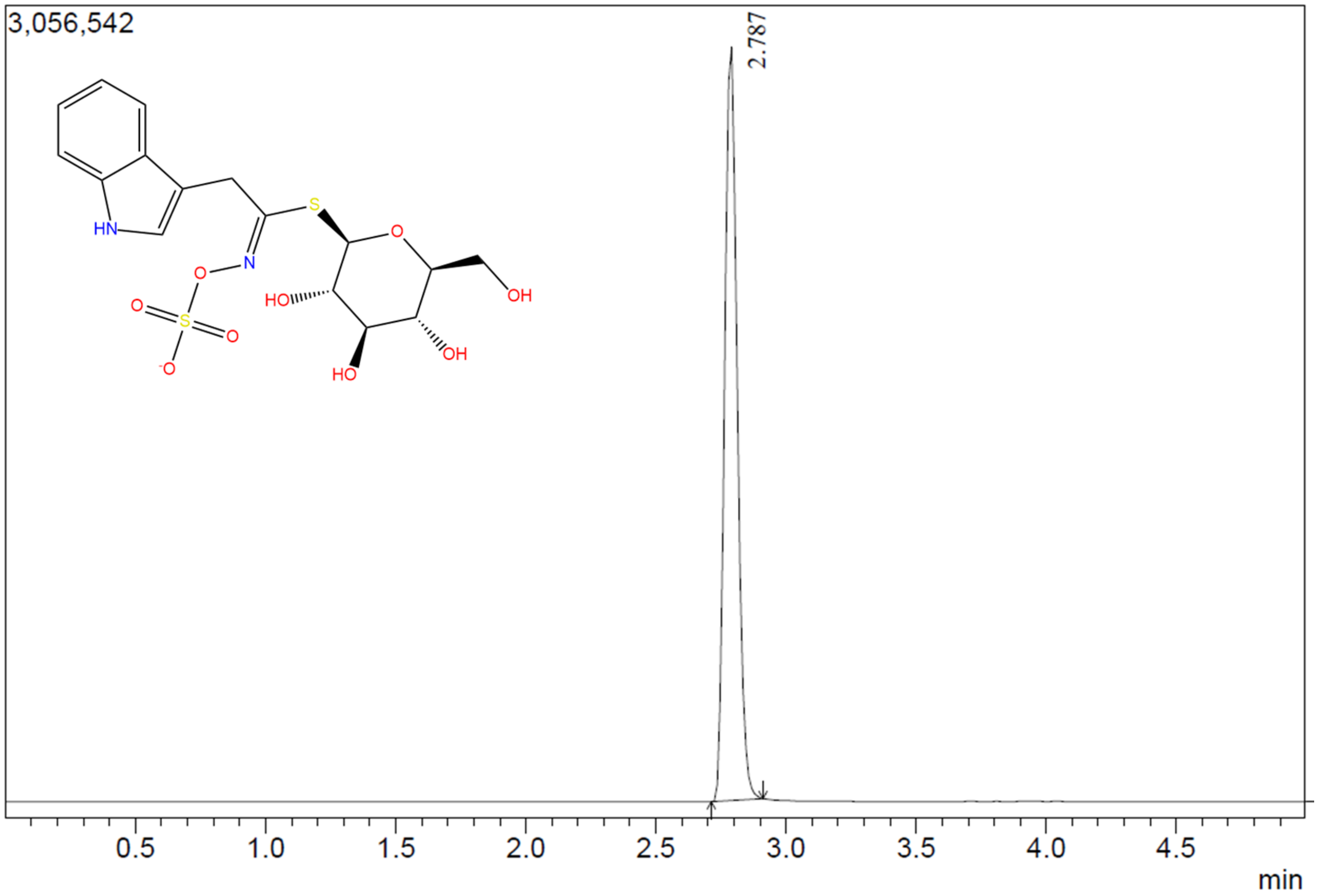

4-methoxyglucobrassicin

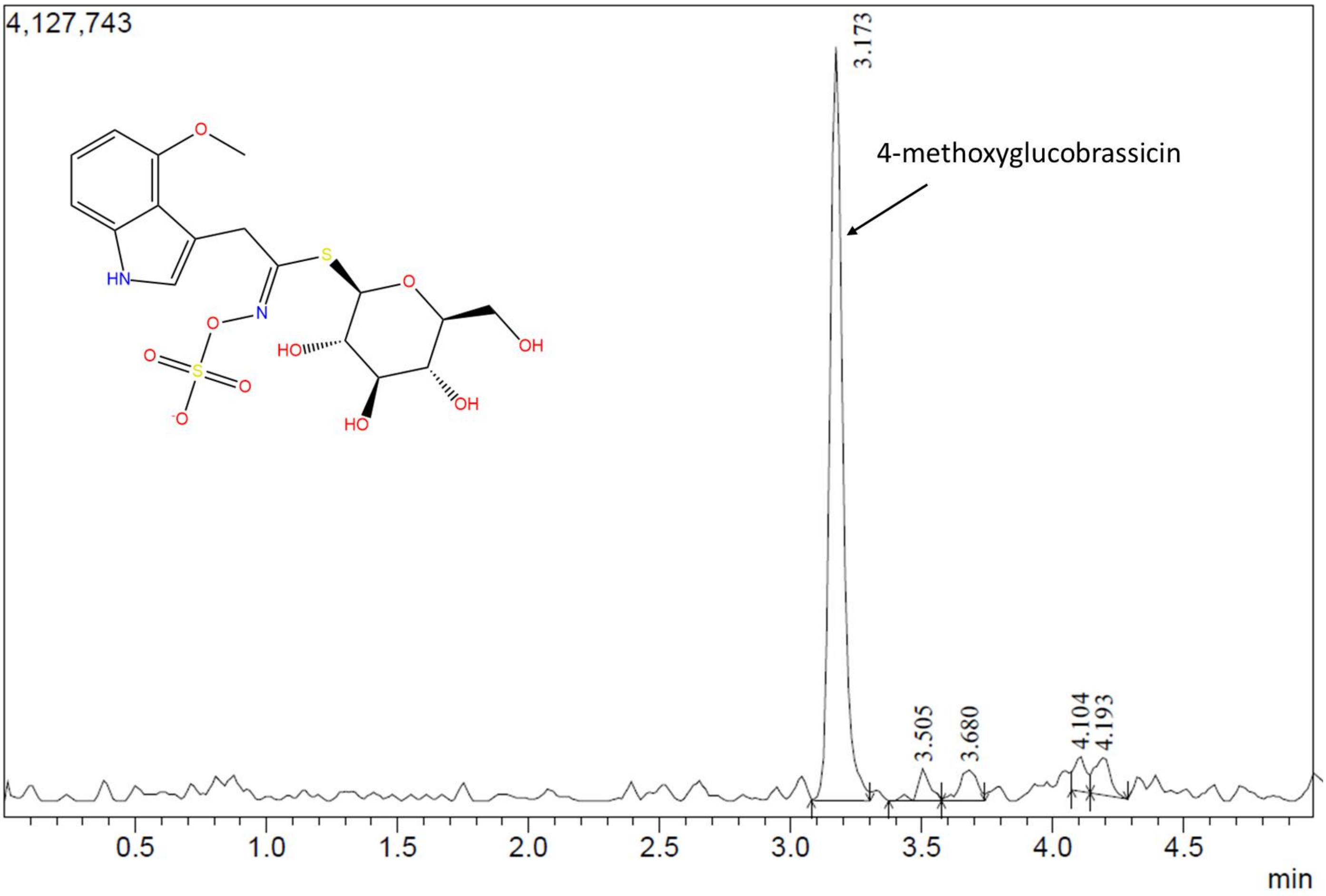

Gluconasturtiin

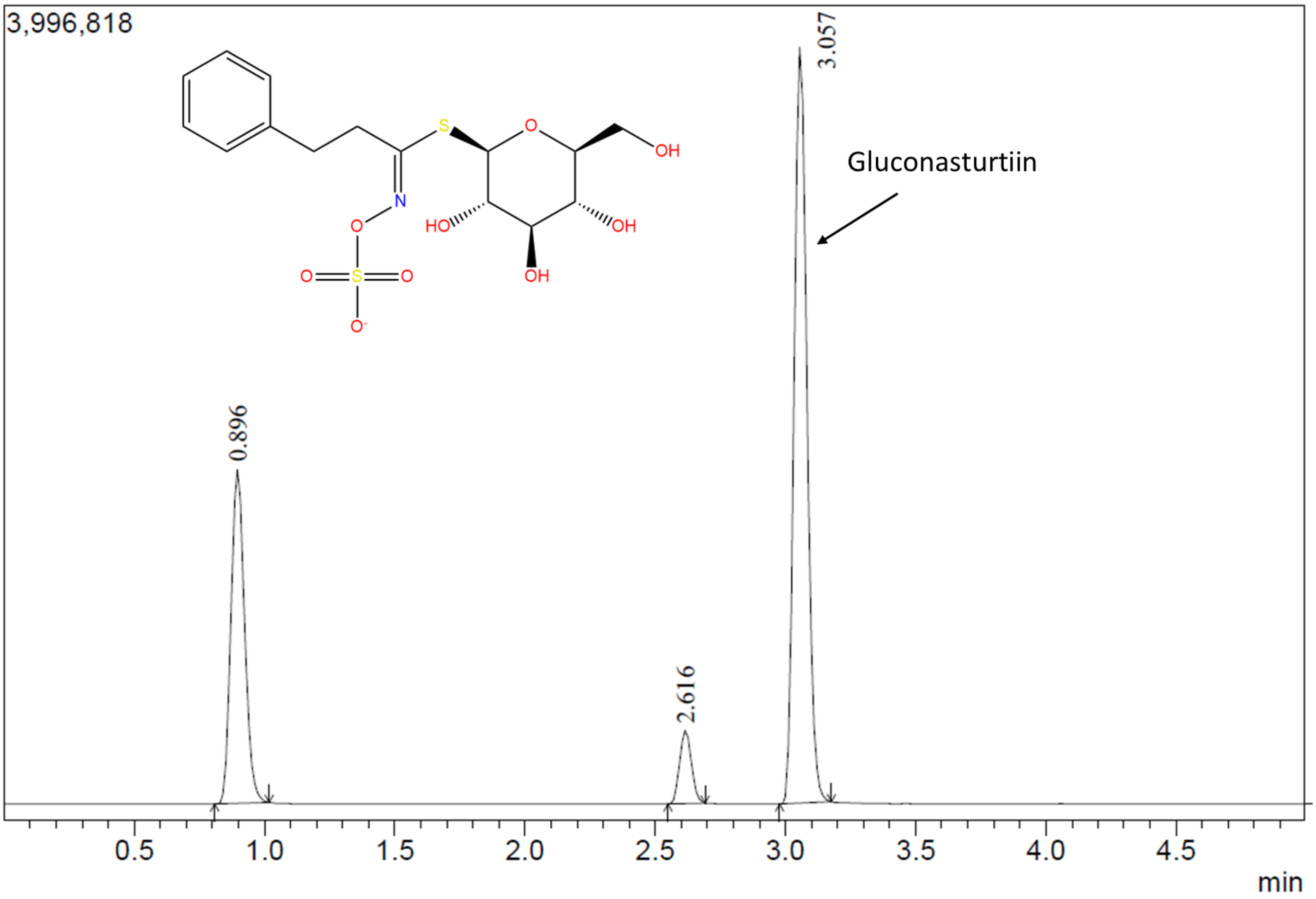

Neoglucobrassicin

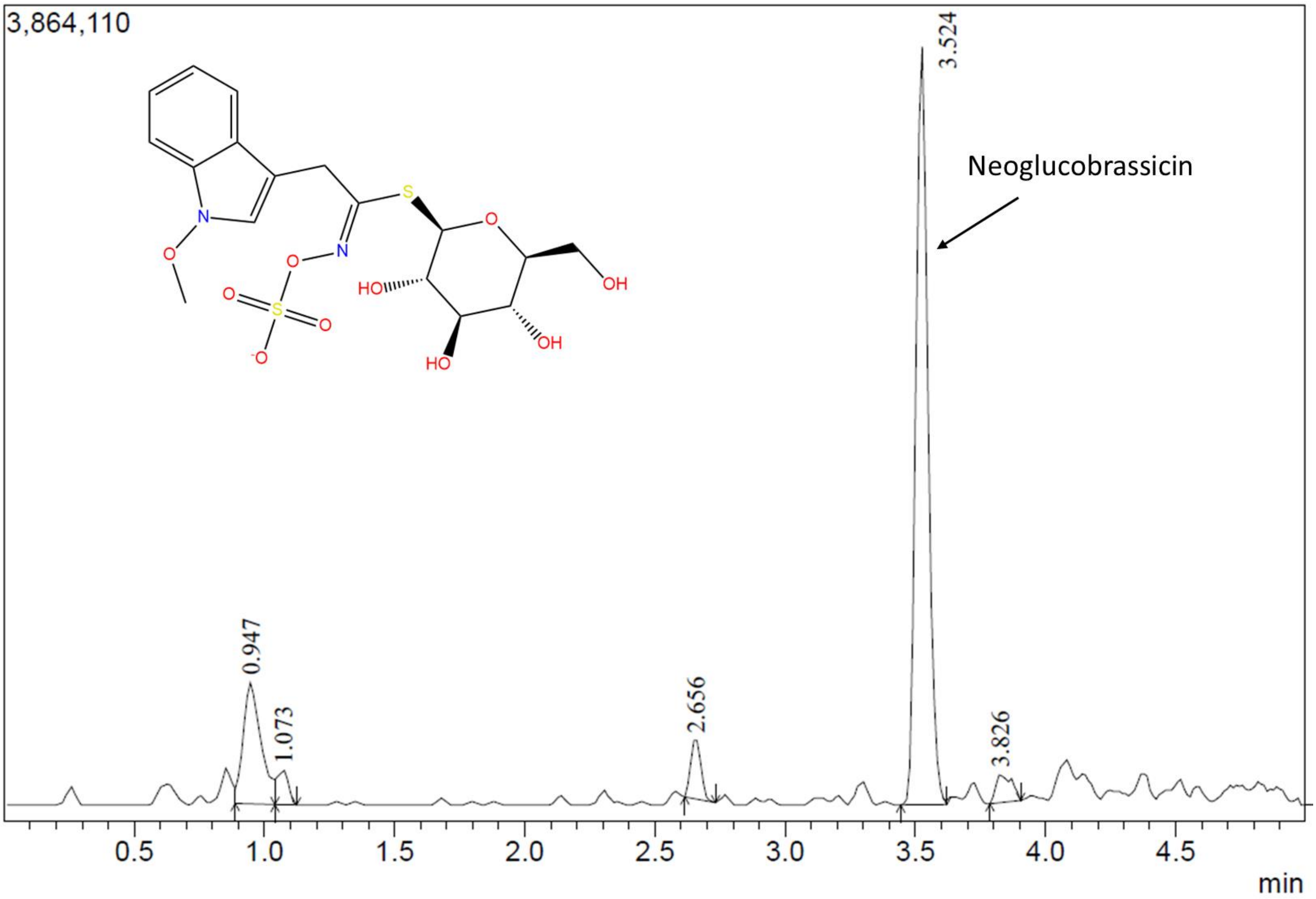

4-methylpentyl GSL

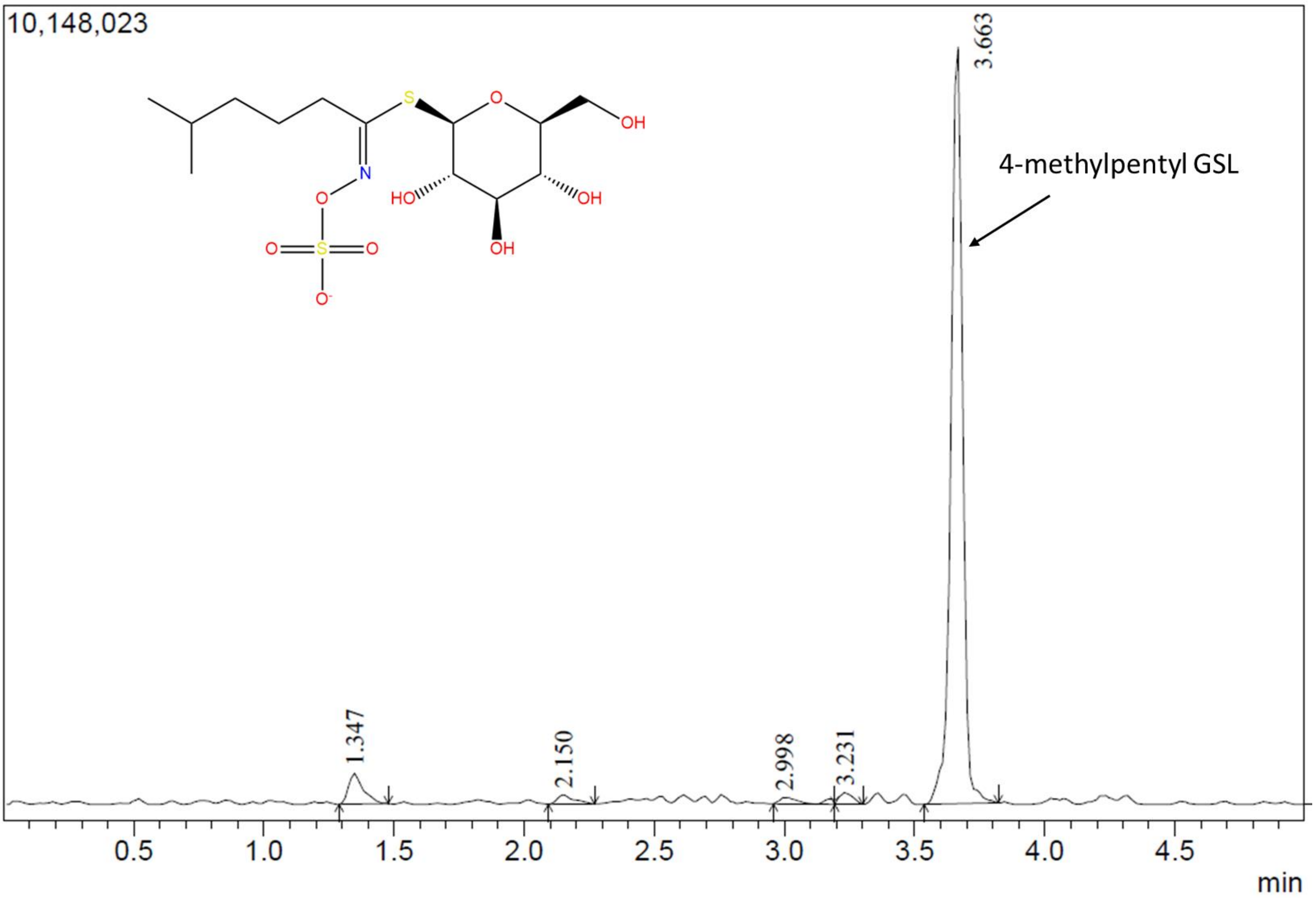

Hexyl GSL

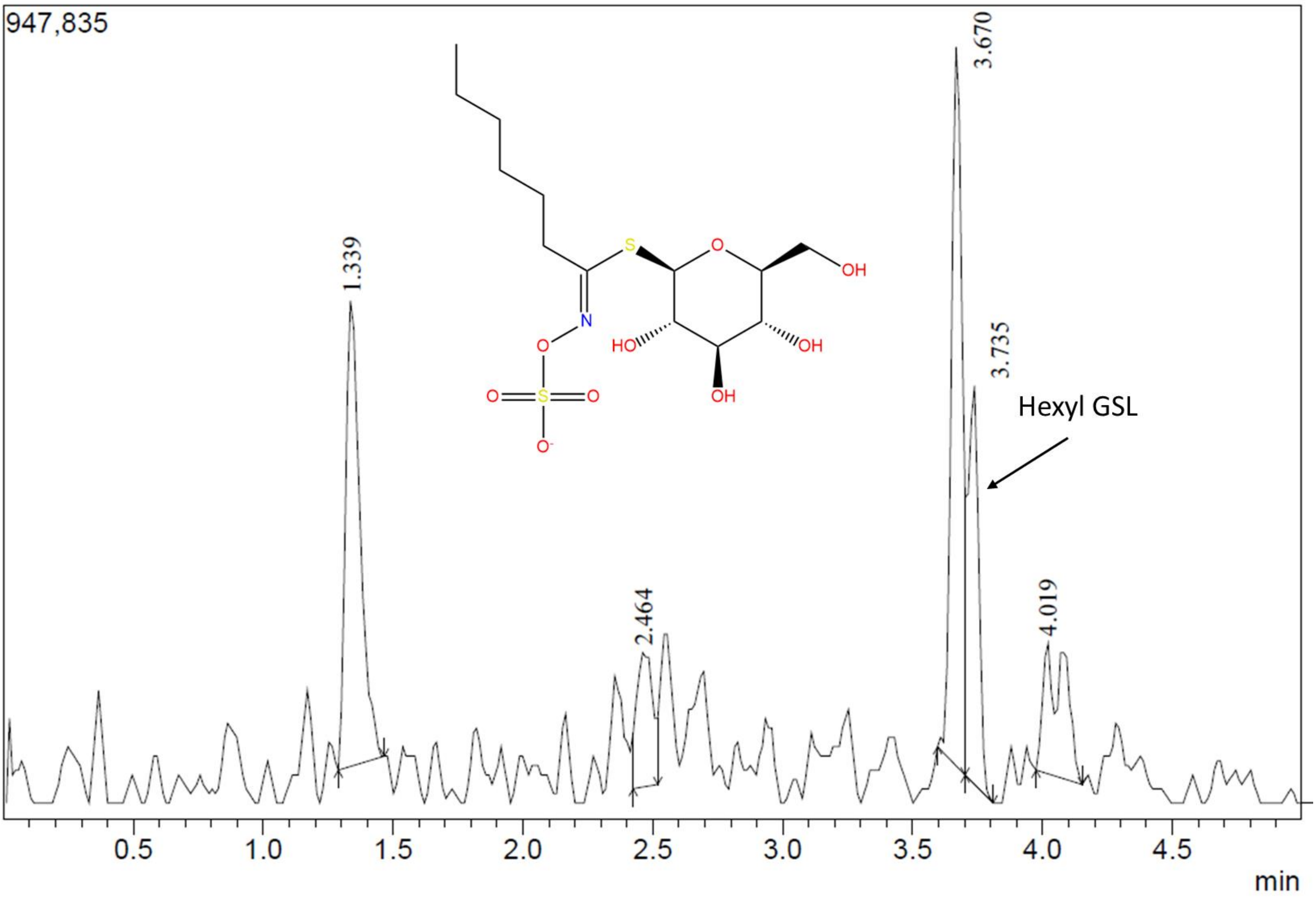

947,835
1.339
2.464
3.670
3.735
4.019
Hexyl GSL
S
O
OH
N
O
HO
OH
OH
O
S
O
O⁻
0.5
1.0
1.5
2.0
2.5
3.0
3.5
4.0
4.5
min

7-(methylthio)heptyl GSL

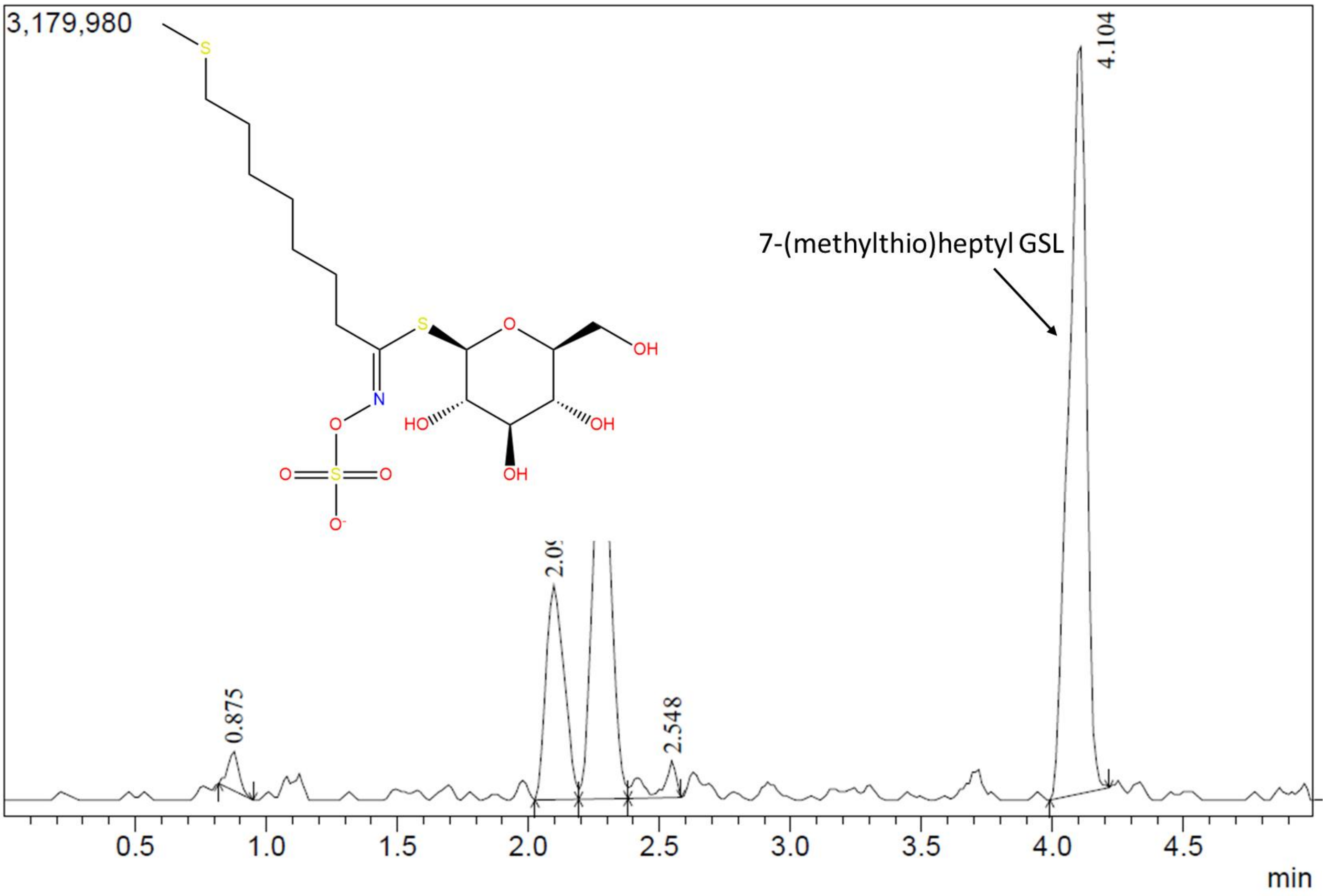

Butyl GSL

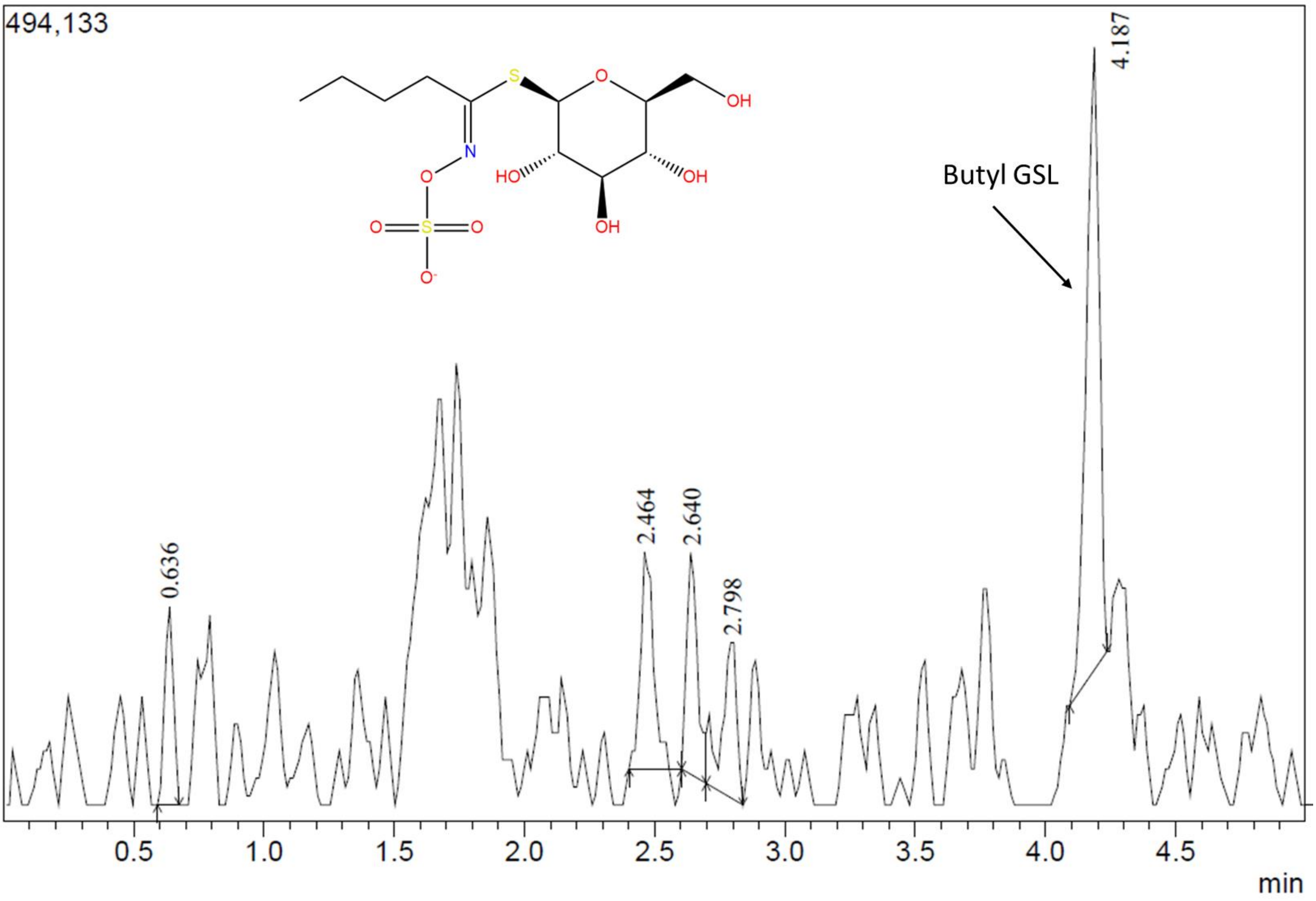

494,133
S
O
OH
N
O
HO
OH
O
S
O
OH
O-
Butyl GSL
4.187
0.636
2.464
2.640
2.798
0.5
1.0
1.5
2.0
2.5
3.0
3.5
4.0
4.5
min
